# Author Correction: Results of the phase IIa RADICAL trial of the FGFR inhibitor AZD4547 in endocrine resistant breast cancer

**DOI:** 10.1038/s41467-023-35969-4

**Published:** 2023-01-17

**Authors:** R. C. Coombes, P. D. Badman, J. P. Lozano-Kuehne, X. Liu, I. R. Macpherson, I. Zubairi, R. D. Baird, N. Rosenfeld, J. Garcia-Corbacho, N. Cresti, R. Plummer, A. Armstrong, R. Allerton, D. Landers, H. Nicholas, L. McLellan, A. Lim, F. Mouliere, O. E. Pardo, V. Ferguson, M. J. Seckl

**Affiliations:** 1 Department of Surgery and Cancer, Imperial College London, London, UK; 2 Cancer Research UK Clinical Trials Unit, Beatson West of Scotland Cancer Centre, Glasgow, UK; 3 Medical Oncology, Addenbrooke’s Hospital, Breast Cancer Research Unit, Cancer Research UK Cambridge Centre, Cambridge, UK; 4 Sir Bobby Robson Cancer Trials Research Centre, Northern Centre for Cancer Care, Freeman Hospital, Newcastle, UK; 5 Breast Research Office, The Christie NHS Foundation Trust, Christie Hospital, Manchester, UK; 6 C8 Admin Offices, Russell’s Hall Hospital, Russells Hall, UK; 7 Astrazeneca, Cheshire, UK; 8 ECMC Programme Office, Research and Innovation, Cancer Research UK, London, UK; 9 Department of Metabolism, Digestion and Reproduction, Imperial College London, London, UK; 10 Amsterdam UMC, Vrije Universiteit Amsterdam, Department of Pathology, Cancer Centre Amsterdam, Amsterdam, The Netherlands

**Keywords:** Breast cancer, Cancer models, Breast cancer

Correction to: *Nature Communications* 10.1038/s41467-022-30666-0

In the original version of this article, the following author (Veronica Ferguson) was omitted from the author list.

Author list, Author contributions and competing interests statements have now been updated in both the PDF and HTML versions of the article.

