# Supplementary material for: Results of the phase IIa RADICAL trial of the FGFR inhibitor AZD4547 in endocrine resistant breast cancer
Source: Nat Commun. 2022 Jun 10;13:3246. doi: 10.1038/s41467-022-30666-0 (PMC9187670; doi:10.1038/s41467-022-30666-0)
Supplement: Supplementary file 2 — Supplementary Information [file 41467_2022_30666_MOESM2_ESM.pdf]

Supplementary tables

Supplementary Table 1a. Recruiting Centre

Centre ID	Location	(R&D) Department	Principal Investigators
RAD 01	Imperial College London, London, UK	Imperial College Healthcare NHS Trust Joint Research Compliance Office	Michael Seckl and Charles Coombes
RAD03	Northern Centre for Cancer Care, Freeman Hospital, Newcastle, UK	Newcastle upon Tyne Hospitals NHS Foundation Trust Joint Research Office	Ruth Plummer (SRI) and Nic Cresti (PIIa)
RAD04	Addenbrooke's Hospital, Cambridge, UK	Cambridge University Hospitals NHS Foundation Trust R&D Department	Richard Baird and Co-Investigator Javier Garcia-Corbacho
RAD05	Beatson West of Scotland Cancer Centre, Glasgow, UK	NHS Greater Glasgow and Clyde R&D Central Office	Iain Macpherson and Co-Investigator Ishtiaq Zubairi
RAD07	Christie Hospital, Manchester, UK	The Christie NHS Foundation Trust, The R&D Division	Anne Armstrong
RAD14	Russells Hall Hospital, Russells Hall, UK	The Dudley Group NHS Foundation Trust R&D Directorate	Rozenn Allerton
RAD16	Queen's Hospital, Burton, UK	Burton Hospitals NHS Foundation Trust R&D Department	Mojca Persic

Supplementary Table 1b: Study Plan (Safety run-in).

Study Period	Screening	NSAI[1] monotherapy		AZD4547 + NSAI[1] combination								AZD4547 / NSAI[1] Discont.	28-day follow -up
**Cycle**	**n/a**	**n/a**		**Cycle 1**				**Cycle 2**		**Cycle 3**	**Cycle 4 (onwards)**		
**Activity / Day**	**-28 to -1**	**1**	**7**	**1**	**7**	**15**	**21**	**1**	**15**	**1**	**1**		
Inclusion/exclusion criteria & informed consent	X												
Demographics	X												
Medical history / Concomitant medical conditions	X	X	X	X	X	X	X	X		X	X	X	
Radiotherapy (previous/current) [2]	X	X	X	X	X	X	X	X		X	X	X	X
Chemotherapy (previous/current)	X												X
Surgical History	X												
Disease Extent/Tumour Characteristics	X												
Concomitant medication	X	X	X	X	X	X	X	X		X	X	X	X
Prohibited medication	X												
Physical examination	X	X	X					X		X	X	X	
ECOG performance status	X	X	X		X	X	X	X		X	X	X	X
Vital signs	X		X					X		X	X	X	X
ECG	X				X			X		X	X	X	
Echo / MUGA scan	X							X[3]				X	
Laboratory evaluations (blood/urine)	X		X		X	X	X	X		X	X	X	X
Ophthalmic assessment	X							X		X	X[4]	X	
Treatment compliance			X	X	X	X	X	X		X	X	X	
PI disease status assessments	X								X		X		

Study Period	Screening	NSAI[1] monotherapy		AZD4547 + NSAI[1] combination								AZD4547 / NSAI[1] Discont.	28-day follow -up
**Cycle**	**n/a**	**n/a**		**Cycle 1**				**Cycle 2**		**Cycle 3**	**Cycle 4 (onwards)**		
**Activity / Day**	**-28 to -1**	**1**	**7**	**1**	**7**	**15**	**21**	**1**	**15**	**1**	**1**		
Pharmacokinetic sampling			X		X								
Blood sample (pharmacodynamics) biomarkers	X		X		X	X	X					X	
Treatment dosing		Treatment will be administered according to details in Section 6.2.3.											
DLT assessment				X	X	X	X	X					
Adverse events		X	X	X	X	X	X	X		X	X	X	X
Archival tumour (optional)	X												
Paired tumour biopsy (optional)	X							X[5]					
Pharmacogenetic sample (optional)		X											

[1] Either anastrozole or letrozole

[2] **ONLY** radiation for palliation at focal sites is permitted whilst the patient is on study medication

[3] After the cycle 2, day 1 assessment, MUGA/ECHO should be performed every three months i.e. at the end of cycles 4, 7 etc. and at discontinuation of AZD4547.

[4] After first 3 months of AZD4547, ophthalmological review should be every 8 weeks (+/- 1 week) until permanent discontinuation of AZD4547

[5] The second biopsy should be collected during cycle 2, but as close to completion of 1st cycle of study treatment as possible

Notes: Activities in italic are unique to Safety run-in study.

Supplementary Table 1c: Study Plan (Phase IIa)

Study period	Screening	AZD4547 + NSAI[1] combination									AZD4547 / NSAI[1] Discont.	28-day follow-up
Cycle	n/a	Cycle 1				Cycle 2		Cycle 3	Cycle 4	Cycle 5 (onwards)		
Activity / Day	-28 to -1	1	8	15	22	1	15	1	1	1		
Inclusion/exclusion criteria & informed consent	X											
Archival tumour[2] sample (mandatory) / exploratory (optional)	X											
Demographics	X											
Medical history / Concomitant medical conditions	X	X	X	X	X	X	X	X	X	X	X	
Radiotherapy (previous/current) [3]	X	X	X	X	X	X	X	X	X	X	X	X
Chemotherapy (previous/current)	X											X
Endocrine Therapy (previous)	X											
Targeted Therapy (previous)	X											
Surgical History	X											
Disease Extent/Tumour Characteristics	X											
Concomitant medication	X	X	X	X	X	X	X	X	X	X	X	X
Prohibited medication	X											
Physical examination	X	X		X		X		X	X	X	X	
ECOG performance status	X	X	X	X	X	X	X	X	X	X	X	X
Vital signs	X	X		X		X		X	X	X	X	X
ECG	X		X			X		X	X	X	X	
Echo / MUGA scan	X							X[4]			X	
Laboratory evaluations (blood/urine)	X	X	X	X	X	X	X	X	X	X	X	X
Ophthalmic assessment	X					X		X	X[5]		X	
Treatment compliance			X	X	X	X		X	X	X	X	

Study period	Screening	AZD4547 + NSAI[1] combination									AZD4547 / NSAI[1] Discont.	28-day follow-up
Cycle	n/a	Cycle 1				Cycle 2		Cycle 3	Cycle 4	Cycle 5 (onwards)		
**Activity / Day**	**-28 to -1**	**1**	**8**	**15**	**22**	**1**	**15**	**1**	**1**	**1**		
Tumour assessments as per RECIST 1.1	X						X		X[6]		X	
Blood sample (pharmacodynamics) biomarkers[7]	X		X	X	X	X		X	X	X	X	
Treatment dosing		Treatment will be administered according to details in Section 6.2.3.										
Adverse events	X[8]	X	X	X	X	X	X	X	X	X	X	X
Paired tumour biopsy (optional)	X[9]				X[10]							
Pharmacogenetics sample (optional)		X										
Circulating tumour specific DNA (ctDNA) (optional)	X[11]	X	X	X	X	X	X	X	X	X	X	X

[1] Either anastrozole or letrozole

[2] If an archival sample is not available, a fresh tumour biopsy sample must be taken.

[3] **ONLY** radiation for palliation at focal sites is permitted whilst the patient is on study medication

[4] After the cycle 3, day 1 assessment, MUGA/ECHO should be performed every three months i.e. at the end of cycles 5, 8 etc. and at discontinuation of AZD4547.

[5] After first 3 months of AZD4547, ophthalmological review should be every 8 weeks (+/- 1 week) i.e. at the end of cycles 5, 7 etc. and at discontinuation of AZD4547

[6] Tumour assessments to be carried out at baseline, week 6, week 12, then every 8 weeks until disease progression or permanent discontinuation of study treatment and finally at AZD4547 discontinuation visit.

[7] If a patient has AZD4547 treatment delays, please contact the Study Team for advice regarding appropriate timing of pharmacodynamics biomarker assessments

[8] AEs collected from the point the patient has been confirmed to be eligible by the RADICAL study team

[9] The first biopsy must only be taken once the patient has been confirmed to be eligible by the RADICAL study team.

[10] The second biopsy **must be taken within 18 hours** of administration of previous AZD4547 dose

[11] Sample to be taken within 1 week of Cycle 1 day 1

Supplementary Table 1c RADICAL was governed by the following committees:

- **Trial Management Group (TMG):** Appointed to oversee the day to day running and progress of the study. The day-to-day management of the study was co-ordinated through ICTU-Ca. The TMG met formally 10 times during the duration of the study and also made decisions by email when appropriate.

- **Safety Review Committee (SRC) - (Safety run-in only):** Appointed to evaluate safety data acquired during the SRI part of the study only and made recommendations on dose de-escalation / modification decisions in the SRI phase and confirmed the dose to take forward into the phase IIa part of the study. The SRC met formally 5 times during the duration of the SRI and also made decisions by email when appropriate.

- **Independent Data Monitoring Committee (IDMC) – (PIIa only):** Appointed to monitor data collected during the phase IIa part of the study only and make recommendations to the Trial Steering Committee (TSC) on whether there are any ethical or safety reasons as to why the trial should not continue. The IDMC met twice during the duration of the study.

  Trial Steering Committee (TSC): Appointed to provide overall supervision for the study including monitoring progress, adherence to the protocol and patient safety. It also considered new information relevant to the research question as it became available and as necessary, advised the TMG on operational issues. The TSC reviewed the recommendations from the IDMC and decided on continuing or stopping the study or modifying the protocol. The TSC met five times during the duration of the study.

Supplementary Table 2: Permanent Discontinuation of Study Treatment*

	Number
Total number of subjects who received treatment	52
Number of subjects who permanently discontinued *prior to week 28***	37
Number of subjects who permanently discontinued *at week 28*	5
Number of subjects who were on treatment *at week 28 and beyond*	10
Reason for permanent discontinuation	
Patient Decision	2
Adverse Event	14
Disease Progression	35
Investigator Decision	1
Number of subjects by tumour response who permanently discontinued *at week 28****	(5)
Stable disease	2
Progressive disease	3
Number of subjects by tumour response who were still on treatment *at week 28 or beyond****	(10)
Partial response	3
Stable disease	6
Progressive disease	1

**Discontinuation time periods include +/- 3 weeks of the scheduled visit. Subjects who withdrew or stopped the treatment before the scheduled tumour assessment were assigned the last known response. Not all subjects who discontinued the study treatment were due to disease progression.*

*** Two subjects were not evaluated in the Central Review.*

**** Of the 15 subjects observed at week 28, five permanently discontinued treatment while 10 continued with the treatment. Among the 15 subjects, 3 had partial response, 8 had stable disease and 4 had progressive disease at week 28 based on Central Review. Source data are provided as a Source Data file.*

Supplementary Table 3a.

Summary of AEs by Grade*

	Grade 1	Grade 2	Missing
	No. of Aes (No. of patients having Aes)		
**Blood and lymphatic system disorders**			
Anaemia	1(1)	1(1)	0(0)
**Eye disorders**			
Retinal pigment epithelium detachment (RPED)	4(2)	0(0)	0(0)
Dry eyes	3(2)	0(0)	0(0)
Eye discharge	1(1)	0(0)	0(0)
Foreign body sensation in eyes	1(1)	0(0)	0(0)
Lacrimation increased	0(0)	0(0)	1(1)
Retinal disorder	2(1)	0(0)	0(0)
**Gastrointestinal disorders**			
Abdomen distention	1(1)	0(0)	0(0)
Abdominal distension	1(1)	0(0)	0(0)
Abdominal mass	2(1)	0(0)	0(0)
Abdominal tenderness	2(1)	0(0)	0(0)
Constipation	5(3)	0(0)	0(0)
Diarrhoea	3(3)	2(1)	0(0)
Dry mouth	3(3)	1(1)	1(1)
Dyspepsia	2(2)	0(0)	0(0)
Gastrointestinal pain	2(1)	0(0)	0(0)
Intestinal obstruction	3(2)	0(0)	0(0)
Mouth ulceration	5(4)	0(0)	0(0)
Nausea	4(3)	0(0)	0(0)
Vomiting	1(1)	0(0)	0(0)
**General disorders and administration site conditions**			
Asthenia	1(1)	0(0)	0(0)
Chest discomfort	1(1)	0(0)	0(0)
Chest pain	1(1)	0(0)	0(0)
Fatigue	4(3)	1(1)	0(0)
Flank pain	1(1)	0(0)	0(0)
Hot flush	1(1)	0(0)	0(0)
Influenza like illness	1(1)	0(0)	0(0)
Metastatic pain	2(1)	0(0)	0(0)
Pain	1(1)	0(0)	0(0)
Procedural pain	1(1)	0(0)	0(0)
Pyrexia	2(1)	0(0)	0(0)
Suprapubic pain	1(1)	0(0)	0(0)
**Immune system disorders**			
Seasonal allergy	2(2)	0(0)	0(0)
**Infections and infestations**			
Oral candidiasis	1(1)	0(0)	0(0)
**Injury, poisoning and procedural complication**			
Postoperative wound complication	1(1)	0(0)	0(0)
**Investigations**			
Alanine aminotransferase increased	6(4)	0(0)	0(0)
Aspartate aminotransferase increased	4(3)	0(0)	0(0)
Blood albumin decreased	4(2)	0(0)	0(0)
Blood alkaline phosphatase increased	3(3)	0(0)	0(0)
Blood calcium decreased	2(2)	0(0)	0(0)

	Grade 1	Grade 2	Missing
	No. of Aes (No. of patients having Aes)		
Blood cholesterol increased	3(3)	0(0)	0(0)
Blood creatinine decreased	5(2)	0(0)	0(0)
Blood creatinine increased	1(1)	0(0)	0(0)
Blood glucose increased	3(2)	0(0)	0(0)
Blood magnesium decreased	4(1)	0(0)	0(0)
Blood magnesium increased	3(2)	0(0)	0(0)
Blood phosphorus decreased	1(1)	0(0)	0(0)
Blood phosphorus increased	8(4)	1(1)	0(0)
Blood potassium decreased	1(1)	0(0)	0(0)
Blood potassium increased	1(1)	0(0)	0(0)
Blood triglycerides increased	1(1)	0(0)	0(0)
Blood urea decreased	1(1)	0(0)	0(0)
Blood urea increased	3(2)	0(0)	0(0)
Blood urine present	1(1)	0(0)	0(0)
Calcium phosphate product increased	0(0)	0(0)	1(1)
Carbohydrate antigen 15-3 increased	1(1)	0(0)	0(0)
Eastern cooperative oncology group performance status worsened	1(1)	1(1)	0(0)
Fundoscopy abnormal	2(1)	0(0)	0(0)
Globulins increased	1(1)	0(0)	0(0)
Haemoglobin decreased	4(3)	0(0)	0(0)
High density lipoprotein decreased	1(1)	0(0)	0(0)
Lymphocyte count decreased	3(2)	0(0)	0(0)
Mean platelet volume increased	1(1)	0(0)	0(0)
Neutrophil count increased	4(2)	0(0)	0(0)
Platelet count increased	2(1)	0(0)	0(0)
Quality of life decreased	0(0)	1(1)	0(0)
Urine ketone body positive	1(1)	0(0)	0(0)
Vitamin d decreased	1(1)	0(0)	0(0)
Weight decreased	1(1)	0(0)	0(0)
Weight increased	1(1)	0(0)	0(0)
White blood cell count decreased	1(1)	0(0)	0(0)
White blood cell count increased	1(1)	0(0)	0(0)
White blood cells urine positive	2(1)	0(0)	0(0)
**Metabolism and nutrition disorders**			
Decreased appetite	2(2)	0(0)	0(0)
Hyponatraemia	1(1)	0(0)	0(0)
**Musculoskeletal and connective tissue disorders**			
Arthralgia	2(2)	0(0)	0(0)
Arthritis	1(1)	0(0)	0(0)
Back pain	2(2)	1(1)	0(0)
Joint swelling	1(1)	1(1)	0(0)
Muscle spasm	2(1)	0(0)	0(0)
Musculoskeletal pain	1(1)	3(1)	0(0)
Musculoskeletal stiffness	1(1)	0(0)	0(0)
Pain in extremity	2(2)	1(1)	0(0)
**Nervous system disorders**			
Burning sensation	3(1)	0(0)	0(0)
Dizziness	1(1)	0(0)	0(0)
Headache	3(3)	0(0)	0(0)
Hypoaesthesia	1(1)	0(0)	0(0)
Neuralgia	1(1)	0(0)	0(0)

	Grade 1	Grade 2	Missing
	No. of Aes (No. of patients having Aes)		
**Renal and urinary disorders**			
Cystitis escherichia	1(1)	0(0)	0(0)
Proteinuria	1(1)	0(0)	0(0)
**Reproductive system and breast disorders**			
Vulvovaginal pain	1(1)	0(0)	0(0)
**Respiratory, thoracic and mediastinal disorders**			
Cough	0(0)	1(1)	0(0)
Dyspnoea	1(1)	0(0)	0(0)
Lower respiratory tract infection	1(1)	1(1)	0(0)
Rales	1(1)	0(0)	0(0)
**Skin and subcutaneous tissue disorders**			
Alopecia	5(3)	0(0)	0(0)
Dry skin	3(2)	0(0)	0(0)
Onychoclasis	2(1)	0(0)	0(0)
Onycholysis	3(2)	0(0)	0(0)
Rash	2(1)	1(1)	0(0)
Rash follicular	4(1)	0(0)	0(0)
Rash pruritic	1(1)	0(0)	0(0)
Skin lesion	2(2)	0(0)	0(0)
**Vascular disorders**			
Epistaxis	5(3)	0(0)	0(0)
Hypertension	1(1)	0(0)	0(0)
**Total†**	**207 (6)**	**17 (4)**	**3(1)**

*****A patient may have more than one AE in any category and a patient is only shown once in each category, but may have Aes in more than one category
†Each patient is counted only once in the total for the number of patients

Supplementary Table 3b.

Summary of AEs by Relation to Study Treatment *

	**Definitely**	**Probably/ Possibly**	**Unlikely/ Not related**	**Not assessable**	**Missing**
	**No. of AEs (No. of patients having AEs)**				
**Blood and lymphatic system disorders**					
Anaemia	0(0)	0(0)	2(2)	0(0)	0(0)
**Eye disorders**					
Detachment of retinal pigment epithelium	2(1)	2(1)	0(0)	0(0)	0(0)
Dry eyes	1(1)	2(1)	0(0)	0(0)	0(0)
Eye discharge	0(0)	1(1)	0(0)	0(0)	0(0)
Foreign body sensation in eyes	0(0)	0(0)	1(1)	0(0)	0(0)
Lacrimation increased	0(0)	0(0)	0(0)	0(0)	1(1)
Retinal disorder	1(1)	1(1)	0(0)	0(0)	0(0)
**Gastrointestinal disorders**					
Abdomen distention	0(0)	0(0)	1(1)	0(0)	0(0)
Abdominal distension	0(0)	0(0)	1(1)	0(0)	0(0)
Abdominal mass	0(0)	0(0)	2(1)	0(0)	0(0)
Abdominal tenderness	0(0)	0(0)	2(1)	0(0)	0(0)
Constipation	0(0)	1(1)	3(3)	1(1)	0(0)
Diarrhoea	0(0)	3(2)	1(1)	0(0)	1(1)
Dry mouth	2(1)	2(2)	0(0)	0(0)	1(1)
Dyspepsia	0(0)	0(0)	2(2)	0(0)	0(0)
Gastrointestinal pain	0(0)	0(0)	2(1)	0(0)	0(0)
Intestinal obstruction	0(0)	0(0)	1(1)	0(0)	0(0)
Mouth ulceration	0(0)	3(2)	0(0)	0(0)	0(0)
Nausea	0(0)	1(1)	4(3)	0(0)	0(0)
Vomiting	0(0)	1(1)	3(2)	0(0)	0(0)
**General disorders and administration site conditions**					
Asthenia	0(0)	0(0)	1(1)	0(0)	0(0)
Chest discomfort	0(0)	0(0)	1(1)	0(0)	0(0)
Chest pain	0(0)	0(0)	1(1)	0(0)	0(0)
Fatigue	0(0)	0(0)	5(4)	0(0)	0(0)
Flank pain	0(0)	0(0)	1(1)	0(0)	0(0)
Hot flush	0(0)	1(1)	0(0)	0(0)	0(0)
Influenza like illness	0(0)	0(0)	0(0)	0(0)	1(1)
Metastatic pain	0(0)	0(0)	2(1)	0(0)	0(0)
Pain	0(0)	0(0)	1(1)	0(0)	0(0)
Procedural pain	0(0)	0(0)	1(1)	0(0)	0(0)
Pyrexia	0(0)	0(0)	2(1)	0(0)	0(0)
Suprapubic pain	0(0)	0(0)	1(1)	0(0)	0(0)
**Immune system disorders**					
Seasonal allergy	0(0)	0(0)	2(2)	0(0)	0(0)
**Infections and infestations**					
Oral candidiasis	0(0)	0(0)	1(1)	0(0)	0(0)
**Injury, poisoning and procedural complication**					
Postoperative wound complication	0(0)	0(0)	1(1)	0(0)	0(0)

	Definitely	Probably/ Possibly	Unlikely/ Not related	Not assessable	Missing
	No. of AEs (No. of patients having AEs)				
**Investigations**					
Alanine aminotransferase increased	0(0)	4(2)	2(2)	0(0)	0(0)
Aspartate aminotransferase increased	0(0)	3(2)	1(1)	0(0)	0(0)
Blood albumin decreased	0(0)	0(0)	4(2)	0(0)	0(0)
Blood alkaline phosphatase increased	0(0)	2(2)	1(1)	0(0)	0(0)
Blood calcium decreased	0(0)	0(0)	2(2)	0(0)	0(0)
Blood cholesterol increased	0(0)	1(1)	2(2)	0(0)	0(0)
Blood creatinine decreased	0(0)	0(0)	5(2)	0(0)	0(0)
Blood creatinine increased	0(0)	0(0)	1(1)	0(0)	0(0)
Blood glucose increased	0(0)	0(0)	3(2)	0(0)	0(0)
Blood magnesium decreased	0(0)	0(0)	4(1)	0(0)	0(0)
Blood magnesium increased	0(0)	0(0)	3(2)	0(0)	0(0)
Blood phosphorus decreased	0(0)	0(0)	1(1)	0(0)	0(0)
Blood phosphorus increased	1(1)	8(4)	0(0)	0(0)	0(0)
Blood potassium decreased	0(0)	0(0)	1(1)	0(0)	0(0)
Blood potassium increased	0(0)	0(0)	1(1)	0(0)	0(0)
Blood triglycerides increased	0(0)	0(0)	1(1)	0(0)	0(0)
Blood urea decreased	0(0)	0(0)	1(1)	0(0)	0(0)
Blood urea increased	0(0)	0(0)	3(2)	0(0)	0(0)
Blood urine present	0(0)	0(0)	1(1)	0(0)	0(0)
Calcium phosphate product increased	0(0)	0(0)	0(0)	0(0)	1(1)
Carbohydrate antigen 15-3 increased	0(0)	0(0)	1(1)	0(0)	0(0)
Eastern cooperative oncology group performance status worsened	0(0)	0(0)	2(2)	0(0)	0(0)
Fundoscopy abnormal	0(0)	2(1)	0(0)	0(0)	0(0)
Globulins increased	0(0)	0(0)	1(1)	0(0)	0(0)
Haemoglobin decreased	0(0)	0(0)	4(3)	0(0)	0(0)
High density lipoprotein decreased	0(0)	0(0)	1(1)	0(0)	0(0)
Lymphocyte count decreased	0(0)	2(1)	1(1)	0(0)	0(0)
Mean platelet volume increased	0(0)	0(0)	1(1)	0(0)	0(0)
Neutrophil count increased	0(0)	2(1)	2(2)	0(0)	0(0)
Platelet count increased	0(0)	0(0)	2(1)	0(0)	0(0)
Quality of life decreased	1(1)**	0(0)	0(0)	0(0)	0(0)
Urine ketone body positive	0(0)	0(0)	1(1)	0(0)	0(0)
Vitamin d decreased	0(0)	0(0)	1(1)	0(0)	0(0)
Weight decreased	0(0)	0(0)	1(1)	0(0)	0(0)

	Definitely	Probably/ Possibly	Unlikely/ Not related	Not assessable	Missing
	No. of AEs (No. of patients having AEs)				
Weight increased	0(0)	0(0)	0(0)	1(1)	0(0)
White blood cell count decreased	0(0)	1(1)	0(0)	0(0)	0(0)
White blood cell count increased	0(0)	0(0)	1(1)	0(0)	0(0)
White blood cells urine positive	0(0)	0(0)	2(1)	0(0)	0(0)
**Metabolism and nutrition disorders**					
Decreased appetite	0(0)	0(0)	2(2)	0(0)	0(0)
Hyponatraemia	0(0)	0(0)	1(1)	0(0)	0(0)
**Musculoskeletal and connective tissue disorders**					
Arthralgia	0(0)	0(0)	2(2)	0(0)	0(0)
Arthritis	0(0)	0(0)	1(1)	0(0)	0(0)
Back pain	0(0)	0(0)	2(2)	1(1)	0(0)
Joint swelling	0(0)	0(0)	2(1)	0(0)	0(0)
Muscle spasm	0(0)	0(0)	2(1)	0(0)	0(0)
Musculoskeletal pain	0(0)	0(0)	2(2)	0(0)	3(1)
Musculoskeletal stiffness	0(0)	0(0)	1(1)	0(0)	0(0)
Pain in extremity	0(0)	0(0)	2(2)	0(0)	1(1)
**Nervous system disorders**					
Burning sensation	0(0)	0(0)	3(1)	0(0)	0(0)
Dizziness	0(0)	0(0)	1(1)	0(0)	0(0)
Headache	0(0)	0(0)	3(3)	0(0)	0(0)
Hypoaesthesia	1(1)**	0(0)	0(0)	0(0)	0(0)
Neuralgia	0(0)	0(0)	1(1)	0(0)	0(0)
**Renal and urinary disorders**					
Cystitis escherichia	0(0)	0(0)	1(1)	0(0)	0(0)
Proteinuria	0(0)	0(0)	1(1)	0(0)	0(0)
**Reproductive system and breast disorders**					
Vulvovaginal pain	0(0)	0(0)	1(1)	0(0)	0(0)
**Respiratory, thoracic and mediastinal disorders**					
Cough	0(0)	0(0)	1(1)	0(0)	0(0)
Dyspnoea	0(0)	0(0)	1(1)	0(0)	0(0)
Lower respiratory tract infection	0(0)	0(0)	2(2)	0(0)	0(0)
Rales	0(0)	0(0)	1(1)	0(0)	0(0)
**Skin and subcutaneous tissue disorders**					
Alopecia	4(3)	1(1)	0(0)	0(0)	0(0)
Dry skin	0(0)	3(2)	0(0)	0(0)	0(0)
Onychoclasis	2(1)	0(0)	0(0)	0(0)	0(0)
Onycholysis	3(2)	0(0)	0(0)	0(0)	0(0)
Rash	0(0)	3(1)	0(0)	0(0)	0(0)
Rash follicular	0(0)	4(1)	0(0)	0(0)	0(0)
Rash pruritic	0(0)	1(1)	0(0)	0(0)	0(0)
Skin lesion	0(0)	0(0)	2(2)	0(0)	0(0)
**Vascular disorders**					
Epistaxis	2(2)**	0(0)	3(1)	0(0)	0(0)
Hypertension	0(0)	0(0)	1(1)	0(0)	0(0)
**Total†**	**20(4)**	**55(6)**	**142(6)**	**3(2)**	**9(1)**

*****Data presented as number of AEs (number of patients having AEs); a patient may have more than one AE in any category and a patient is only shown once in each category, but may have AEs in more than one category
******The classification of Causality of AE is currently being queried with the site
†Each patient is counted only once in the total for the number of patients

Supplementary Figures

Supplementary Figure 1

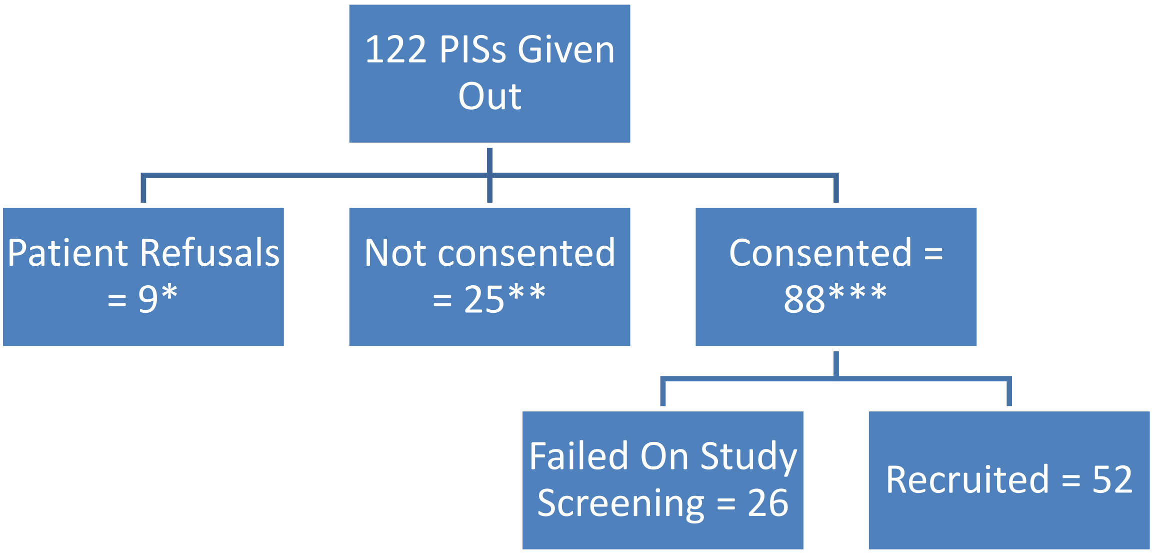

Patient Consort Diagram

** 2 Patients thought there were too many study visits; 2 patients gave no reason; 1 patient had preconceived ideas about research; 1 patient wanted a complete change of treatment; 1 patient wanted 3rd line endocrine treatment and 2 patients were concerned about potential study treatment toxicities.*

***11 patients went onto other treatments; 4 patients had sclerotic and non lytic bone lesions, therefore not eligible; 3 patients had ongoing eye issues; 2 patients had poor ECOG; 1 patient had history of malignancy within 5 years, therefore not eligible; 1 patient found not to be eligible due to result of CT scan; 1 patient was not truly menopausal; 1 patient decided to leave U.K. for a few months and 1 patient had ongoing cardiac problems.*

****10 patients did not enter study screening because of the following reasons: 8 patients were withdrawn from screening as too unwell to continue; 1 patient's medical team decided alternative treatment would better suit the patient and there was 1 mistake with patient eligibility.*

Supplementary Figure 2

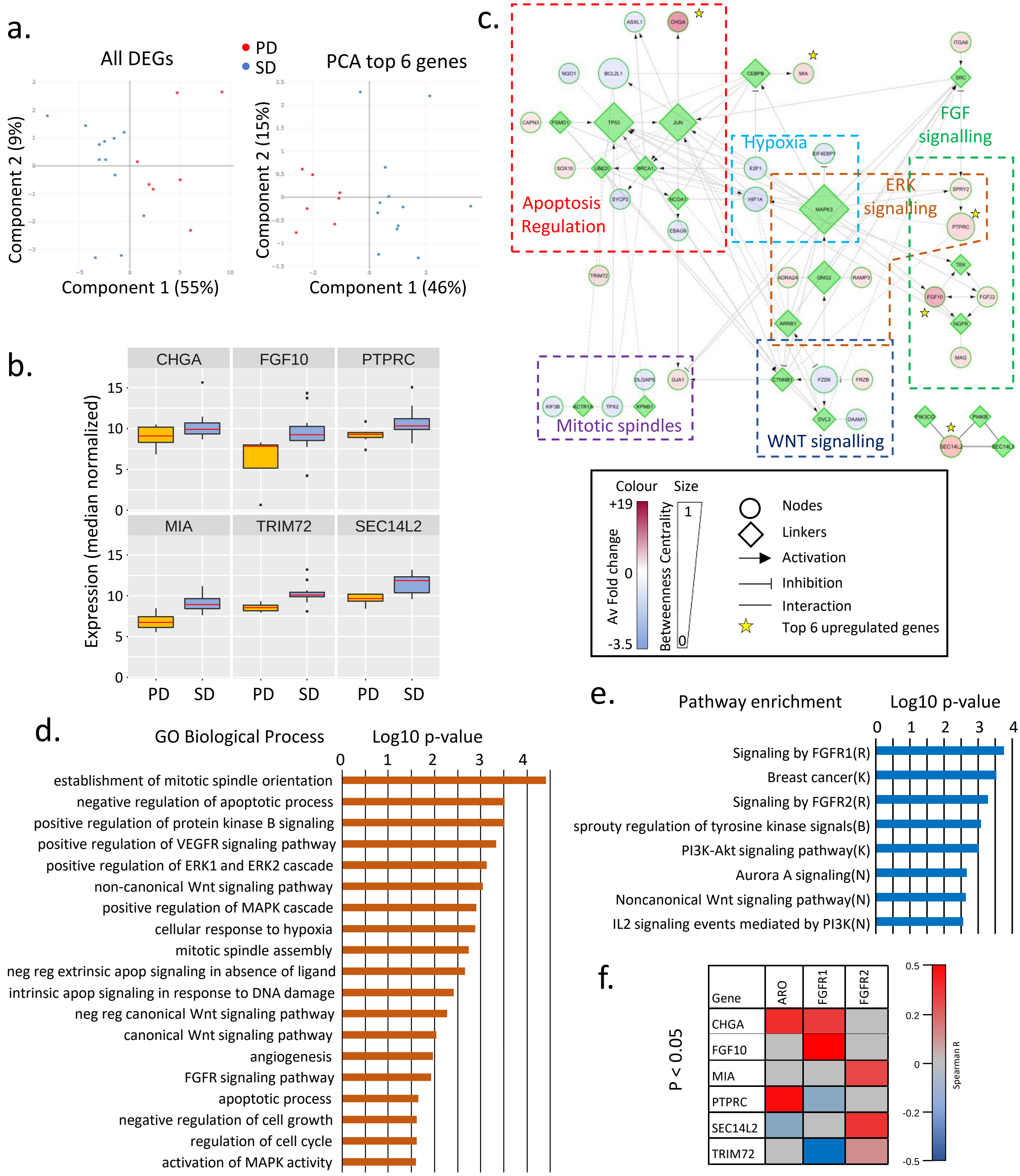

A gene signature made of the top 6 upregulated differentially expressed genes enables discrimination between responders and non-responders to AI/FGFR inhibitor combination. N= 12 SD and 6 PD patient samples. Supplementary Figure 2b: boxes represent 75% and side-bars 12.5%. Red bar represents the median. N= 12 SD and 6 PD patient samples. Supplementary Figure 2d and e: p-values are the result of a Fisher's exact test. Supplementary Figure 2f: the p-value is the result of a t-test performed as part of the cor() function in R using the ranked correlation option (Spearman); Source data are provided as a Source Data file.

Supplementary Figure 3

a.

Genes	DESEq2	edgeR	Av FC
CHGA	3.25	33.33	18.29
FGF10	3.6	21.73	12.665
PTPRC	3.47	6.34	4.905
MIA	3.71	4.9	4.305
TRIM72	3.54	4.91	4.225
SEC14L2	3.34	4.35	3.845
GJA1	2.96	4.36	3.66
MAG	3.04	4.15	3.595
ITGA8	3.15	4.01	3.58
RAMP3	2.73	3.6	3.165
ADRA2A	2.6	3.14	2.87
TMEFF1	2.53	3.13	2.83
FGF22	2.52	2.92	2.72
SOX10	2.39	2.73	2.56
FRZB	2.07	2.3	2.185
SPRY2	1.93	2.08	2.005
CAPN3	1.83	1.93	1.88
ASXL1	-1.68	-1.7	-1.69
DLGAP5	-1.79	-1.81	-1.8
DAAM1	-1.81	-1.84	-1.825
KIF3B	-1.81	-1.84	-1.825
EBAG9	-1.87	-1.92	-1.895
BCL2L1	-2.05	-2.1	-2.075
EIF4EBP1	-2.11	-2.25	-2.18
E2F1	-2.2	-2.33	-2.265
FZD6	-2.24	-2.42	-2.33
TPX2	-2.32	-2.53	-2.425
TSPYL5	-2.41	-2.72	-2.565
PRR15L	-2.57	-2.92	-2.745
NQO1	-2.54	-3.07	-2.805
SYCP2	-2.87	-3.82	-3.345
HIF1A	-2.87	-4.03	-3.45

b.

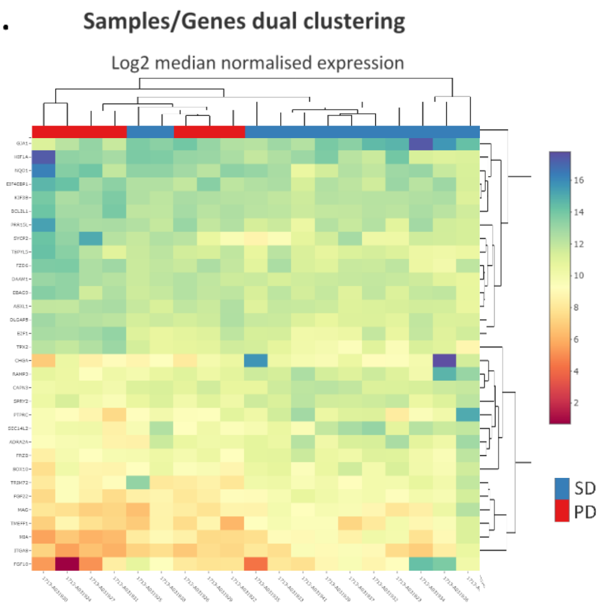

c.

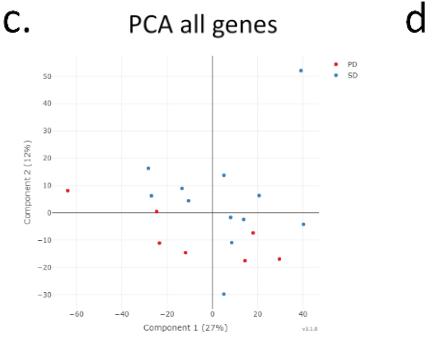

d.

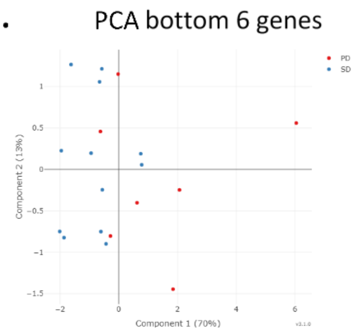

The entire list of differentially expressed genes (DEGs) or the top 6 downregulated DEGs do not enable discrimination between responders and non-responders to AI/FGFR inhibitor combination. Supplementary Figure 3a: the p-values were adjusted using the Benjamini-Hochberg method and a cut-off of 10% was used to select the hits; Source data are provided as a Source Data file. (a-d) N= 12 SD and 6 PD patient samples.

Supplementary Figure 4

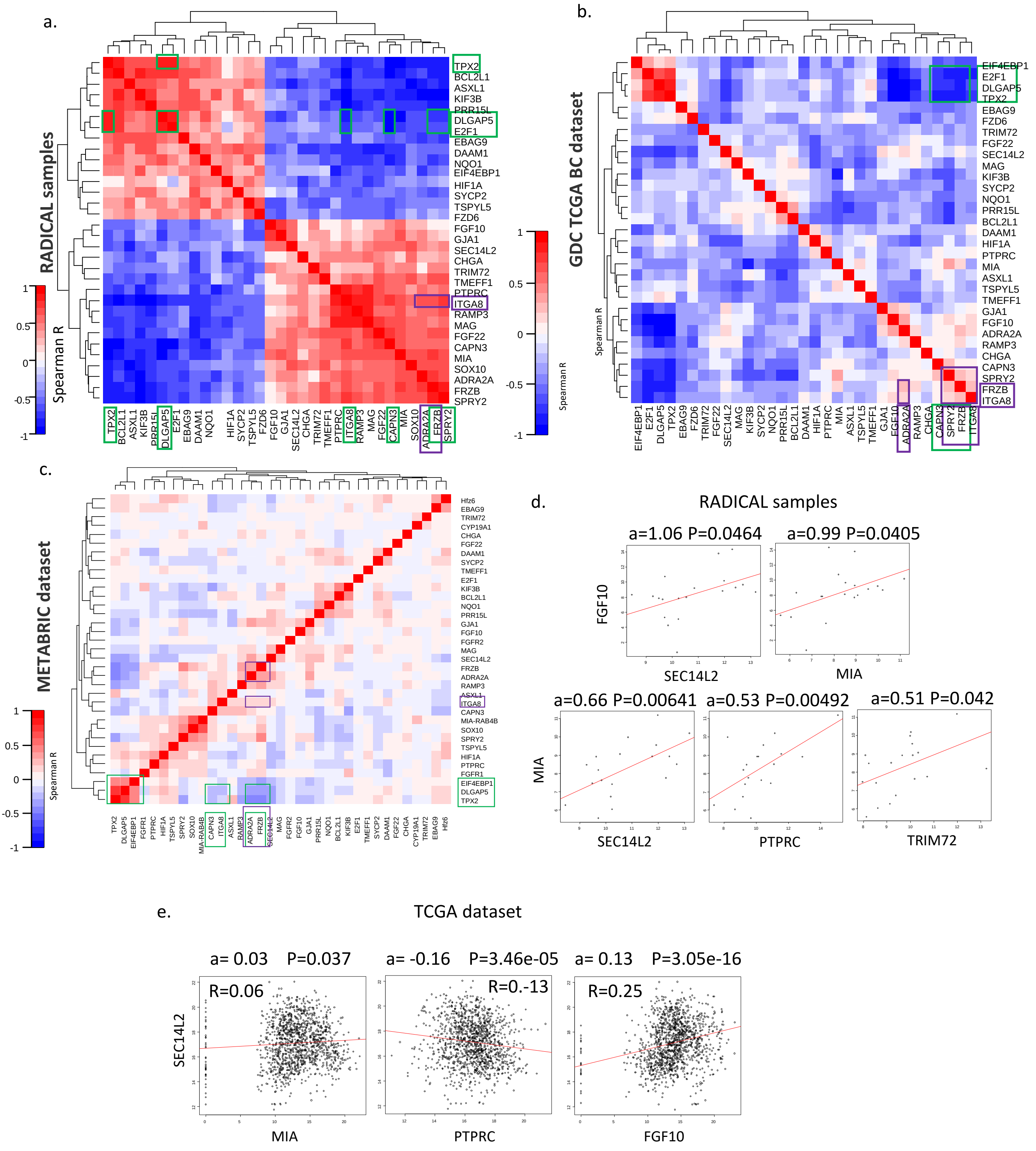

Correlation between the expression of our differentially expressed genes in our and two publicly-available datasets. Supplementary Figure 4d and e: the p-value is the result of a t-test performed as part of the lm() function in R. "a" represents the correlation coefficients; Source data are provided as a Source Data file. (a) N= 12 SD and 6 PD patients. (b) N=1055 patient samples (c) N=2509 patient samples. (d) N= 19. (e) N=1055 patient samples.

Supplementary Figure 5

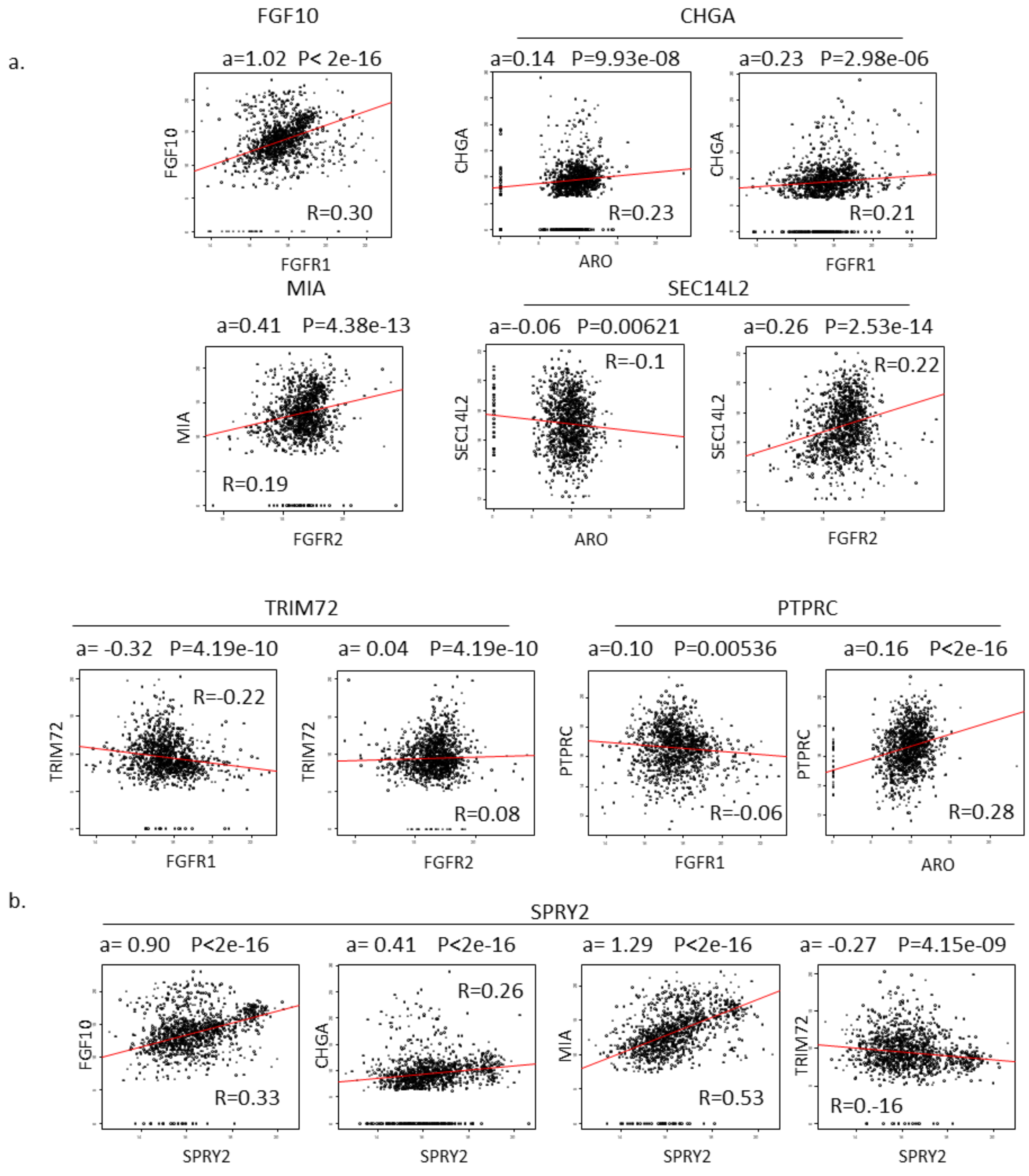

Linear correlation between the expression of our 6 top upregulated DEGs and that of FGFR1/2, SPRY2 and ARO in the <u>Breast Cancer GDC TCGA dataset.</u> N=1055 patient samples. The p value is the result of a t-test performed as part of the lm() function in R. "a" represents the correlation coefficients.

Supplementary Note 1

Final Clinical Bioanalysis Report

Project Code:	D2610
Clinical Study Number:	D2610C00011
CRO Reference Number:	8264324
Date:	07 January 2014

8264324: The Determination of Letrozole and Anastrozole in PK Samples from D2610C00011 (RADICAL)

Contributing Scientist: Gordon Swan, BSc

Covance Laboratories Ltd
Otley Road,
Harrogate, North Yorkshire,
HG3 1PY, England

TABLE OF CONTENTS PAGE

	TITLE PAGE	1
	TABLE OF CONTENTS	2
	LIST OF TABLES	4
	LIST OF FIGURES	5
	LIST OF FIGURES	5
	LIST OF APPENDICES	7
	BIOANALYTICAL SITE	8
	CLINICAL BIOANALYSIS ALLIANCE REPRESENTATIVE	8
	ARCHIVING	8
	EXPERIMENTAL DATES	9
	ADDITIONAL RESPONSIBLE PERSONS	9
	AUTHENTICATION STATEMENT	10
	QA STATEMENT	11
SUMMARY		12
Summary information		12
1.	INTRODUCTION	13
2.	MATERIALS AND METHODS	13
2.1	Analytical Method for Analysis of Letrozole in Human Plasma	13
2.2	Analytical Method for Analysis of Anastrozole in Human Plasma	13
2.3	Reference Standards for Letrozole	14
	Internal Standard	14
2.4	Reference Standards for Anastrozole	15
	Internal Standard	15
	Co-administered Drug	15
2.5	Computer systems	16
2.6	Calibration Standards for Letrozole	16
2.7	QC Samples for Letrozole	16
2.8	Calibration Standards for Anastrozole	16
2.9	QC Samples for Anastrozole	17
2.10	Study samples	17

2.11 Acceptance criteria 17
3. PROTOCOL DEVIATIONS 18
4. ANALYTICAL PROCEDURE DEVIATION 18
5. RESULTS AND DISCUSSION FOR LETROZOLE ANALYSIS 19
5.1 Selectivity 19
5.2 Calibration standards 19
5.3 QC samples 20
5.4 Carryover assessment 20
5.5 Study samples 20
5.6 Repeat analysis 21
5.7 Incurred sample reproducibility (ISR) 21
5.8 Selectivity in the Presence of a Co-Administered Drug (AZD4547) 21
6. RESULTS AND DISCUSSION FOR ANASTROZOLE ANALYSIS 21
6.1 Selectivity 22
6.2 Calibration standards 22
6.3 QC samples 22
6.4 Carryover assessment 23
6.5 Study samples 23
6.6 Repeat analysis 23
6.7 Incurred sample reproducibility (ISR) 23
6.8 Selectivity in the Presence of a Co-Administered Drug (AZD4547) 24
7. CONCLUSION 24
8. REFERENCES 25
9. TABLES 26
9.1 Letrozole 28
9.2 Anastrazole 35
10. LETROZOLE FIGURES 42
11. ANASTROZOLE FIGURES 57
12. CERTIFICATES OF ANALYSIS 72

LIST OF TABLES

Table 1 Run Analysis Summary, Clinical Study Number D2610C00011 27

Table 2 Back-Calculated Concentrations of Letrozole in Calibration Standards in Human Plasma 29

Table 3 Calibration Curve Parameters of Letrozole in Human Plasma............30

Table 4 Determined Concentrations of Letrozole in QC Samples in Human Plasma............31

Table 5 Incurred Sample Reproducibility Results for Letrozole in Human Plasma (ng/mL) 32

Table 6 Quantification of Letrozole in the Presence of AZD4547 (Run 1) 34

Table 7 Back-Calculated Concentrations of Anastrozole in Calibration Standards in Human Plasma 36

Table 8 Calibration Curve Parameters of Anastrozole in Human Plasma............37

Table 9 Determined Concentrations of Anastrozole in QC Samples in Human Plasma 38

Table 10 Incurred Sample Reproducibility Results for Anastrozole in Human Plasma (ng/mL) 39

Table 11 Quantification of Anastrozole in the Presence of AZD4547 (Run 6)............41

Table A1 Concentration of Letrozole in Human Plasma from AstraZeneca Study D2610C00011 80

Table B1 Concentration of Anastrozole in Human Plasma from AstraZeneca Study D2610C00011 86

LIST OF FIGURES

Figure 1 Plasma Blank with Added Internal Standard Chromatogram (Run 2) 43
Figure 2 LLOQ Plasma Calibration Standard (1 ng/mL) Chromatogram (Run 2) 44
Figure 3 ULOQ Plasma Calibration Standard (1000 ng/mL) Chromatogram (Run 2) 45
Figure 4 LoQC Plasma Sample (3 ng/mL) Chromatogram (Run 2) 46
Figure 5 MeQC Plasma Sample (50 ng/mL) Chromatogram (Run 2) 47
Figure 6 HiQC Plasma Sample (750 ng/mL) Chromatogram (Run 2) 48
Figure 7 Plasma Sample Collected From Subject RAD040001, Monotherapy Day 7, 0.5 Hour Sample Chromatogram (Run 4) 49
Figure 8 Plasma Sample Collected From Subject RAD040001, Monotherapy Day 7, 8 Hour Sample Chromatogram (Run 4) 50
Figure 9 Plasma Sample Collected From Subject RAD040001, Cycle 1 Day 7, 1 Hour Sample Chromatogram (Run 4) 51
Figure 10 Plasma Sample Collected From Subject RAD040001, Cycle 1 Day 7, 6 Hour Sample Chromatogram (Run 4) 52
Figure 11 Plasma Sample Collected From Subject RAD010001, Monotherapy Day 7, 3 Hour Sample Chromatogram (Run 1) 53
Figure 12 Plasma Sample Collected From Subject RAD010001, Monotherapy Day 7, 5 Hour Sample Chromatogram (Run 1) 54
Figure 13 Plasma Sample Collected From Subject RAD010001, Cycle 1 Day 7, 0 Hour Sample Chromatogram (Run 1) 55
Figure 14 Plasma Sample Collected From Subject RAD010001, Cycle 1 Day 7, 4 Hour Sample Chromatogram (Run 1) 56
Figure 15 Plasma Blank with Added Internal Standard Chromatogram (Run 10) 58
Figure 16 LLOQ Plasma Calibration Standard (1 ng/mL) Chromatogram (Run 10) 59
Figure 17 ULOQ Plasma Calibration Standard (100 ng/mL) Chromatogram (Run 10) 60
Figure 18 LoQC Plasma Sample (3ng/mL) Chromatogram (Run 10) 61
Figure 19 MeQC Plasma Sample (40 ng/mL) Chromatogram (Run 10) 62
Figure 20 HiQC Plasma Sample (70 ng/mL) Chromatogram (Run 10) 63

Figure 21 Plasma Sample Collected From Subject RAD010002, Monotherapy Day 7, 3 Hour Sample Chromatogram (Run 7) 64

Figure 22 Plasma Sample Collected From Subject RAD010002, Monotherapy Day 7, 8 Hour Sample Chromatogram (Run 7) 65

Figure 23 Plasma Sample Collected From Subject RAD010002, Cycle 1 Day 7, 2 Hour Sample Chromatogram (Run 7) 66

Figure 24 Plasma Sample Collected From Subject RAD010002, Cycle 1 Day 7, 5 Hour Sample Chromatogram (Run 7) 67

Figure 25 Plasma Sample Collected From Subject RAD010005, Monotherapy Day 7, 0.5 Hour Sample Chromatogram (Run 7) 68

Figure 26 Plasma Sample Collected From Subject RAD010005, Monotherapy Day 7, 4 Hour Sample Chromatogram (Run 7) 69

Figure 27 Plasma Sample Collected From Subject RAD010005, Cycle 1 Day 7, 0 Hour Sample Chromatogram (Run 7) 70

Figure 28 Plasma Sample Collected From Subject RAD010005, Cycle 1 Day 7, 6 Hour Sample Chromatogram (Run 7) 71

LIST OF APPENDICES

Appendix A Bioanalytical Results for Letrozole in Human Plasma............................79
Appendix B Bioanalytical Results for Anastrozole in Human Plasma.........................85

BIOANALYTICAL SITE

Contributing Scientist	Katherine Catton (from 11 October 2012 until 10 October 2013)<br><br>Gordon Swan (from 11 October up to report finalisation)<br><br>Covance Laboratories Limited<br>Otley Road, Harrogate<br>North Yorkshire, HG3 1PY<br>England

CLINICAL BIOANALYSIS ALLIANCE REPRESENTATIVE

Clinical Bioanalysis Alliance (CBioA) Program Manager on behalf of AstraZeneca R&D	Nina Gaw (from 11 October 2012 to 06 September 2013)<br><br>Tammy Harter (from 06 September 2013)<br><br>Covance Laboratories Limited<br>Otley Road, Harrogate<br>North Yorkshire, HG3 1PY<br>England

ARCHIVING

All primary data (including electronic data), or authenticated copies thereof, the protocol and the final report will be retained using appropriate storage media in the Covance Laboratories Limited archives for at least five years after issue of the final report. At this time the Sponsor will be contacted to determine whether the data should be returned or destroyed on their behalf. Sponsors will be notified of the financial implications of each of these options at that time.

Specimens or samples requiring frozen storage at Covance are specifically excluded from the above. These will be retained for as long as the material permits further evaluation or up to a maximum of three months after issue of the draft report. At this time, the Sponsor will be contacted to determine whether samples should be returned, retained or destroyed on their behalf. Any financial implications of these options will also be notified at this time. Samples will not be destroyed without prior approval of the Contributing Scientist.

EXPERIMENTAL DATES

Dates of sample receipt:	05 November 2012 to 21 October 2013 (11 sample shipments in total)
Bioanalytical work started:	09 May 2013 (first extracted run)
Bioanalytical work completed:	05 Nov 2013 (last reported run)

ADDITIONAL RESPONSIBLE PERSONS

The following personnel besides the Contributing Scientist were also significantly involved in the conduct and evaluation of this part of the study:

Bioanalysis:	Melissa Abel, Paul Snelling, Ian Dawson and Anna Rozej
	Responsible for sample analysis
	Philip Brain, Ashfaq Ali and Daniel Lloyd
	Responsible for LC-MS/MS operation

AUTHENTICATION STATEMENT

Clinical Study Number: D2610C00011

Study Title: 8264324: The Determination of Letrozole and Anastrozole in PK Samples from D2610C00011 (RADICAL)

Study Part: Bioanalysis

I, the undersigned, hereby declare that the data generated are scientifically acceptable and valid and the report provides a true and accurate record of the results obtained.

The study was performed in accordance with the agreed protocol and with Covance Laboratories Limited Standard Operating Procedures, unless otherwise stated, and the study objectives were achieved.

There were no deviations from the protocol, Standard Operating Procedures or applicable regulations which had an impact on the rights, well-being or confidentiality of the patient or on the validity and interpretation of the data.

This study involved the analysis of human samples generated as part of a clinical trial, and as such was not within the scope of Good Laboratory Practice (GLP) regulations. No formal claim of compliance with GLP was therefore made, although all work was conducted in a facility that is a member of the UK MHRA GLP monitoring program following laboratory procedures commensurate with International Standards of GLP.

As part of a clinical study, the work was conducted in accordance with the following:

- UK Statutory Instrument 2004 No. 1031: The Medicines for Human Clinical Use (Clinical Trials) Regulations 2004 plus subsequent amendments
- Good Clinical Practice: Consolidated Guideline ICH Topic E6, adopted by CPMP, July 1996, issued as CPMP/ICH/135/95

....

Gordon Swan, BSc
Contributing Scientist

Date: 07 JANUARY 2014

QA STATEMENT

This study has been subject to the independent Quality Assurance programmes defined in Covance QA Standard Operating Procedures.

Penelope J Wilkes | 7th January 2014.

Penelope J Wilkes
QA Representative
Covance QA

Date

SUMMARY

Information regarding this study part is detailed below:

Summary information

Test compound	Letrozole<br>Anastrozole
Analytical matrix	Human plasma containing sodium heparin
Analyte	Letrozole<br>Anastrozole
Internal standard (IS)	Letrozole: Letrozole-D4<br>Anastrozole: Anastrozole-d12
Validated method	Letrozole: HB-12-076<br>Anastrozole: HB-09-098
Lower limit of quantification	1 ng/mL for both Letrozole and Anastrozole
Validated range	Letrozole: 1 -1000 ng/mL<br>Anastrozole: 1 -100 ng/mL
Quality control (QC) levels	Letrozole: 3 ng/mL (LoQC), 50 ng/mL (MeQC), 750 ng/mL (HiQC) and 5000 ng/mL (DiQC)<br>Anastrozole: 3 ng/mL (LoQC), 40 ng/mL (MeQC), 70 ng/mL (HiQC) and 250 ng/mL (DiQC)
Analytical technique/method of detection	Letrozole: Liquid-liquid extraction followed by liquid chromatography (LC) with tandem mass spectrometric (MS/MS) detection<br>Anastrozole: Automated protein precipitation followed by liquid chromatography (LC) with tandem mass spectrometric (MS/MS) detection
Total number of samples analysed	Letrozole: 96 (including 42 samples that were analysed in error. See section 3 for further discussion)<br>Anastrozole: 42
Sample storage conditions	Nominal -20°C (for both Letrozole and Anastrozole)

In summary, the calibration standards, QC sample data and the ISR assessment met the acceptance criteria. The overall maximum precision (%RSD) for Letrozole plasma QC samples at the three concentrations assessed was 3.4%, with a % bias of between -2.4% to 2.2%. The overall maximum precision (RSD%) for Anastrozole plasma QC samples at the three concentrations assessed was 11.7%, with a % bias of between -4.7% to 0.1%.

The data indicated that the method performed reliably during the analysis of Letrozole and Anastrozole plasma study samples. All study samples were analysed within their established stability period. Analytical results for the determination of Letrozole and Anastrozole concentrations in human plasma samples collected in Clinical Study Number D2610C00011 [1] are presented in this report.

1. INTRODUCTION

The objective of this study was to determine the concentration of Letrozole and Anastrozole in human plasma using liquid chromatography (LC) with tandem mass spectrometric (MS/MS) detection.

2. MATERIALS AND METHODS

2.1 Analytical Method for Analysis of Letrozole in Human Plasma

The concentrations of Letrozole in human plasma were determined by supported liquid-liquid extraction and liquid chromatography with tandem mass spectrometric detection (LC-MS/MS) following method number HB-12-076. The analytical method has a calibration range of 1 -1000 ng/mL, utilizing a 50 µL sample aliquot, with a validated dilution of 10-fold with human plasma

The method has been validated prior to sample analysis and reported in Covance Study Number 8264611 [2] .

2.2 Analytical Method for Analysis of Anastrozole in Human Plasma

The concentrations of Anastrozole in human plasma were determined by automated protein precipitation and liquid chromatography with tandem mass spectrometric detection (LC-MS/MS) following method number HB-09-098. The analytical method for Anastrozole has a calibration range of 1 -100 ng/mL, utilizing a 25 µL sample aliquot, with a validated dilution of 10-fold with human plasma.

The method has been previously validated prior to sample analysis and reported in Covance Study Number and 8216406 [3] .

2.3 Reference Standards for Letrozole

The following reference materials were provided and used to prepare the calibration and QC samples:

Compound code:	Letrozole
Batch number:	2-EOD-12-1
Covance material ID:	87510
Purity:	98.0%
Correction factor:	0.980
Received on:	15 October 2012
Storage conditions:	Frozen (nominal -20°C)
Retest date:	12 April 2015

The Certificate of Analysis for Letrozole can be found in the raw data.

Internal Standard

The following internal standard was added to calibration standards, quality control (QC) samples and study samples:

Compound code:	Letrozole-d_4
Batch number:	CR4-44
Purity:	100%
Correction factor:	1.00
Received on:	22 June 2012
Storage conditions:	Frozen (nominal -20°C)
Expiry date:	06 June 2014

The Certificate of Analysis for Letrozole-d_4 can be found in the raw data.

2.4 Reference Standards for Anastrozole

The following reference materials were provided and used to prepare the calibration and QC samples:

Compound code:	Anastrozole
Lot number:	5-XJZ-121-1
Covance material ID:	132043
Purity:	98.0%
Correction factor:	0.980
Received on:	01 Feb 2013
Storage conditions:	Frozen (nominal -20°C)
Expiry date:	07 Sep 2015

The Certificate of Analysis for Anastrozole can be found in the raw data.

Internal Standard

The following internal standard was added to calibration standards, quality control (QC) samples and study samples:

Compound code:	Anastrozole-d_{12}
Lot number:	5-GBH-105-1
Purity:	98% (Chemical Purity)
Correction factor:	0.970
Received on:	15 Oct 2012
Storage conditions:	Frozen (nominal -20°C)
Expiry date:	07 Oct 2014

The Certificate of Analysis for Anastrozole-d_{12} can be found in the raw data.

Co-administered Drug

The following co-administered drug was used in this study:

Compound code:	AZD4547
Batch number:	C510/2
Purity:	99.8%
Correction factor:	0.998
Received on:	23 Oct 2012
Storage conditions:	15-25°C
Expiry date:	31 May 2014

The Isotope Chemistry Data Sheet (Certificate of Analysis) for AZD4547 can be found in the raw data.

2.5 Computer systems

The following computer systems were used in this study. The version numbers of the systems are held on file at Covance:

System	Use
Nautilus	Sample Tracking
Watson	Laboratory Information Management System (LIMS)
Analyst	Data collection and processing for all Sciex MS/MS instruments

2.6 Calibration Standards for Letrozole

Plasma calibration standards were prepared on 07 May 2013 prior to sample analysis. Calibration standards were stored at nominal -20°C and were prepared at the following concentrations:

1, 2, 7.5, 25, 100, 400, 800 and 1000 ng/mL

2.7 QC Samples for Letrozole

Plasma QC samples were prepared on 07 May 2013 prior to sample analysis. QC Samples were stored at nominal -20°C and were prepared at the following concentrations:

3 ng/mL (LoQC), 50 ng/mL (MeQC), 750 ng/mL (HiQC) and 5000 ng/mL (DiQC)

DiQC samples were prepared however they were not required to be used.

2.8 Calibration Standards for Anastrozole

Plasma calibration standards were prepared on 28 Oct 2013 prior to sample analysis. Calibration standards were stored at nominal -20°C and prepared at the following concentrations:

1, 2, 5, 7, 10, 30, 50, 80 and 100 ng/mL

2.9 QC Samples for Anastrozole

Plasma QC samples were prepared on 28 October 2013, prior to sample analysis and subsequently on 31 October 2013 and 04 November 2013. QC Samples were stored at nominal -20°C and prepared at the following concentrations:

3 ng/mL (LoQC), 40 ng/mL (MeQC), 70 ng/mL (HiQC) and 250 ng/mL (DiQC)

DiQC samples were prepared however they were not required to be used.

2.10 Study samples

The first subject was dosed on 29 October 2012 with sodium heparin used as the anticoagulant for the human plasma samples. A total of 96 Letrozole samples and 96 Anastrozole samples were received at the bioanalytical site. The samples arrived on 11 occasions between 05 November 2012 and 21 October 2013, from Quintiles Laboratories Europe, Livingston, EH54 7EG. A total of 96 Letrozole samples (including 42 samples that were analysed in error. See section 3 for further discussion) and 42 Anastrozole samples were analysed. All samples were received frozen and in good condition and stored in a freezer set at nominal -20°C prior to analysis (with the exception of the samples from run 2 which are discussed in section 3).

2.11 Acceptance criteria

The acceptance criteria for the calibration curves were as follows:

All calibration standards utilized within the regression had back-calculated values within 85 - 115% (80-120% for the LLOQ) of the nominal concentration.

No more than 25% of the calibration standard levels were excluded from the calibration curves. For Letrozole, at least six calibration standard levels were to remain in the calibration curve, for a run to be acceptable. For Anastrozole, at least seven calibration standard levels were to remain in the calibration curve, for a run to be acceptable

Calibration standards at the lower or upper limit of quantification could be excluded and a revised calibration range used, providing that QC samples were all within the revised calibration range. The reason for exclusion of any calibration standards was documented in the raw data.

The acceptance criteria for QC samples were as follows:

Two undiluted QC samples at each of three concentrations (LoQC, MeQC and HiQC), were analysed in each run. At least four of the six QC samples were within ±15% of their respective nominal values; with a minimum of one QC sample within limit at each concentration.

Dilution of samples and therefore dilute QC samples (DiQC) were not required throughout this study.

3. PROTOCOL DEVIATIONS

The following deviations from the protocol were noted during the course of the study. These deviations were considered not to have compromised the validity of the study or the results:

- Samples from analytical run 2 were stored at nominal -80°C between 24 January 2013 and 23 May 2013. As 197 days storage stability has been established at both nominal -20°C and nominal -80°C, the integrity of the samples is confirmed to be unaffected
- Samples from subjects RAD01002, RAD01004 and, RAD01005 (a total of 42 samples) were analysed for Letrozole in error. Letrozole was not administered to these three subjects; therefore samples from these subjects should not have been analysed using the Letrozole method. The data for all samples from these subjects were confirmed as below the LLOQ
- In the ISR assessment for Anastrozole, high internal standard response was observed in four of repeat results. As all repeat results confirmed the original values and as acceptable calibration standards and QC samples were observed with comparable internal standard response, the internal standard was considered to be performing acceptably. The data were therefore considered to be acceptable and further reanalysis was not performed

In the opinion of the Contributing Scientist these deviations have no impact on the integrity of the results reported in this bioanalytical study part.

4. ANALYTICAL PROCEDURE DEVIATION

The following deviation from the analytical procedure was noted during the course of the study. This deviation was considered not to have compromised the validity of the study or the results obtained:

- During the Anastrozole ISR analysis, in run 10, the samples were submitted to a fourth freeze thaw cycle, which was outside the validated three cycles, as documented in the analytical procedure. The initial, reported analysis was performed within three freeze/thaw cycle and as the repeat results from all samples confirmed the original results, ISR data were considered to be acceptable and to confirm the performance of the assay

5. RESULTS AND DISCUSSION FOR LETROZOLE ANALYSIS

Numerical data may have been rounded for presentation. Therefore, manual recalculation may give slightly different values to those shown.

A summary of the analytical runs performed in this study are present in Table 1.

5.1 Selectivity

In each analytical run, a blank plasma sample and a blank plasma sample spiked with internal standard were included to test the specificity of the procedure to interferences (endogenous compounds and possible impurities). A reagent blank sample was also analysed to test for possible impurities. A plasma blank containing internal standard is presented in Figure 1.

The selectivity blanks should demonstrate a lack of significant interference in the chromatographic regions of interest for the analyte, peak area, ≤20.0% of the utilised LLOQ calibration standard peak area.

Additionally, the selectivity blanks without internal standard should also demonstrate a lack of significant interference in the chromatographic regions of interest for the internal standard, peak area ≤5% of the internal standard peak area for the matrix blank with internal standard.

There were no interfering peaks (> 20% of the LLOQ peak area) detected in reagent and matrix blanks in the retention window of Letrozole. There were no interfering peaks (> 5% of the matrix blank with internal standard peak area) detected in selectivity blanks with no internal standard in the retention window of the internal standard. Selectivity was considered acceptable in all accepted runs.

5.2 Calibration standards

Calibration curves for Letrozole in human plasma were calculated using a linear regression curve employing a $1/x^2$ weighting. Single calibration standards were analysed. The individual and mean back-calculated concentrations, precision (%RSD) and accuracy (% bias) of the plasma calibration samples from accepted runs are presented in Table 2. The regression parameters (slope, intercept and correlation coefficient) for all accepted calibration curves of Letrozole are presented in Table 3.

Typical lower limit of quantification (LLOQ) and upper limit of quantification (ULOQ) chromatograms are presented in Figure 2 and Figure 3, respectively.

5.3 QC samples

The precision (%RSD) and accuracy (% bias) of the bioanalytical method were assured over the course of the assays based on the results of the plasma QC samples, which were assayed on each day of the sample analysis. The precision and accuracy of the plasma QC samples from accepted runs are presented in Table 4.

Typical chromatograms from each QC level are presented in Figure 4 to Figure 6.

No plasma dilution QC samples were analysed throughout the course of this study.

5.4 Carryover assessment

One matrix blank was analysed after the two highest calibration standards in each analytical run (carryover assessment blanks), one near the beginning and one near the end of the run. The carryover assessment blanks should demonstrate a lack of significant interference in the chromatographic regions of interest for the analyte, peak area, ≤20.0% of the utilised LLOQ calibration standard peak area.

Additionally, the carryover assessment blanks should also demonstrate a lack of significant interference in the chromatographic regions of interest for the internal standard, peak area ≤5% of the internal standard peak area for the matrix blank with internal standard.

There were no interfering peaks (> 20% of the LLOQ peak area) detected in carryover assessment blanks in the retention window of Letrozole. There were no interfering peaks (> 5% of the matrix blank with internal standard peak area) detected in carryover assessment blanks in the retention window of the internal standard.

Carry-over was considered acceptable in all accepted runs.

5.5 Study samples

Samples were analysed in a total of four reported runs. All four analytical runs met the acceptance criteria.

A total of 54 samples (excluding the 42 samples which were analysed in error) were received and analysed for concentrations of Letrozole in human plasma samples, from Clinical Study Number D2610C00011. All study samples were analysed within the (197 days) established stability period for Letrozole in human plasma. QC samples represented the range of the samples analysed.

Individual plasma concentrations of Letrozole are presented in Appendix A.

Representative chromatograms from plasma samples are presented in Figure 7 through Figure 14.

5.6 Repeat analysis

No samples required repeat analysis.

5.7 Incurred sample reproducibility (ISR)

ISR in human plasma was investigated in this study. A set of 25 study samples for Letrozole, with concentrations higher than the LLOQ, were reassayed for the purpose of demonstrating the reproducibility of the method when applied to incurred samples. The difference between the reanalysis result and the original result, relative to the original result was calculated for each sample as a percentage. At least two-thirds of the samples in the evaluation demonstrated a percentage difference within ±20.0%, thus meeting the acceptance criteria. The results are shown in Table 5.

5.8 Selectivity in the Presence of a Co-Administered Drug (AZD4547)

Testing was required to assess the potential for AZD4547 to interfere with the HB-12-076 analytical method. This was performed in analytical run 1.

Blank matrix (three replicates) was spiked with AZD4547 at approximately 2000 ng/mL. In addition, AZD4547 (2000 ng/mL) was spiked into a LoQC sample (six replicates). The blank samples did not contain any significant peaks at the retention time of Letrozole or internal standard.

The accuracy and precision values of the LoQC samples spiked with AZD4547 (2000 ng/mL) were within the acceptance criteria as stated in the protocol. The data confirm that the presence of AZD4547 does not affect the quantification of Letrozole. The data are presented in Table 6.

6. RESULTS AND DISCUSSION FOR ANASTROZOLE ANALYSIS

Numerical data may have been rounded for presentation. Therefore, manual recalculation may give slightly different values to those shown.

A summary of the analytical runs performed in this study are present in Table 1.

Low internal standard response was observed within the run, due to suspected sample processing error during extraction and therefore run 5 was not reported. The samples were successfully re-analysed in run 7 and the data reported.

Run 8 was not reported, as the LoQC samples did not meet the acceptance criteria. QC samples were re-prepared. A test analytical run (run 9) was performed in order to confirm the performance of the QC samples. The performance of the QC samples was acceptable however as this is a trial run the data have not been reported. The samples from run 8 were re-analyzed in run 10 and the data reported.

6.1 Selectivity

In each analytical run, a blank plasma sample and a blank plasma sample spiked with internal standard were included to test the specificity of the procedure to interferences (endogenous compounds and possible impurities). A reagent blank sample was also analysed to test for possible impurities. A plasma blank containing internal standard is presented in Figure 15.

The selectivity blanks should demonstrate a lack of significant interference in the chromatographic regions of interest for the analyte, peak area, ≤20.0% of the utilised LLOQ calibration standard peak area.

Additionally, the selectivity blanks without internal standard should also demonstrate a lack of significant interference in the chromatographic regions of interest for the internal standard, peak area ≤5% of the internal standard peak area for the matrix blank with internal standard.

There were no interfering peaks (> 20% of the LLOQ peak area) detected in reagent and matrix blanks in the retention window of Anastrozole. There were no interfering peaks (> 5% of the matrix blank with internal standard peak area) detected in selectivity blanks with no internal standard in the retention window of the internal standard. Selectivity was considered acceptable in all accepted runs.

6.2 Calibration standards

Calibration curves for Anastrozole in human plasma were calculated using a linear regression curve employing a 1/x weighting. Single calibration standards were analysed, with duplicate standards at the LLOQ and ULOQ. The individual and mean back-calculated concentrations, precision (%RSD) and accuracy (% bias) of the plasma calibration samples from accepted runs are presented in Table 7. The regression parameters (slope, intercept and correlation coefficient) for all accepted calibration curves of Anastrozole are presented in Table 8.

Typical lower limit of quantification (LLOQ) and upper limit of quantification (ULOQ) chromatograms are presented in Figure 16 and Figure 17, respectively.

6.3 QC samples

The precision (%RSD) and accuracy (% bias) of the bioanalytical method were assured over the course of the assays based on the results of the plasma QC samples, which were analysed on each day of the sample analysis. The precision and accuracy of the plasma QC samples from accepted runs are presented in Table 9.

One LoQC sample in run 7 was confirmed as a statistical outlier. Data are presented both including and excluding the outlier.

Typical chromatograms from each QC level are presented in Figure 18 to Figure 20.

No plasma dilution QC samples were analysed throughout the course of this study.

6.4 Carryover assessment

One matrix blank was analysed after the two highest calibration standards in each analytical run (carryover assessment blanks), one near the beginning and one near the end of the run. The carryover assessment blanks should demonstrate a lack of significant interference in the chromatographic regions of interest for the analyte, peak area, ≤20.0% of the utilised LLOQ calibration standard peak area.

Additionally, the carryover assessment blanks should also demonstrate a lack of significant interference in the chromatographic regions of interest for the internal standard, peak area ≤5% of the internal standard peak area for the matrix blank with internal standard.

There were no interfering peaks (> 20% of the LLOQ peak area) detected in carryover assessment blanks in the retention window of Anastrozole. There were no interfering peaks (> 5% of the matrix blank with internal standard peak area) detected in carryover assessment blanks in the retention window of the internal standard.

Carry-over was considered acceptable in all accepted runs.

6.5 Study samples

Samples were analysed in a total of two reported runs.

A total of 42 samples were received to be analysed for Anastrozole from Clinical Study Number D2610C00011. All active study samples were analysed within the (883 days) established stability for Anastrozole in human plasma. QC samples represented the range of the samples analysed.

Individual plasma concentrations of Anastrozole are presented in Appendix B.

Representative chromatograms from plasma samples are presented in Figure 21 to Figure 28.

6.6 Repeat analysis

No samples required repeat analysis.

6.7 Incurred sample reproducibility (ISR)

ISR in human plasma was investigated in this study. A set of 25 study samples for Anastrozole, with concentrations higher than the LLOQ, were reassayed for the purpose of demonstrating the reproducibility of the method when applied to incurred samples. The difference between the reanalysis result and the original result, relative to the original result was calculated for each sample as a percentage. At least two-thirds of the samples in the evaluation demonstrated a percentage difference within ±20.0%, thus meeting the acceptance criteria. The results are shown in Table 10.

ISR samples were analysed in run 10. The repeat and original results from all 24 samples selected were within ISR acceptance criteria. High internal standard response was observed in four of the repeat results, however as all results confirmed the original values and as acceptable calibration standards and QC samples were observed with comparable high internal standard response, the internal standard was considered to be performing acceptably. The data were therefore considered to be acceptable and further reanalysis was not performed.

6.8 Selectivity in the Presence of a Co-Administered Drug (AZD4547)

Testing was required to assess the potential for AZD4547 to interfere with the HB-09-098 analytical method. This was performed in analytical run 6.

Blank matrix (three replicates) was spiked with AZD4547 at approximately 2000 ng/mL. In addition, AZD4547 (2000 ng/mL) was spiked into LoQC samples (six replicates). The blank samples did not contain any significant peaks at the retention time of Anastrozole or internal standard.

The accuracy and precision values of the LoQC samples spiked with AZD4547 (2000 ng/mL) were within the acceptance criteria as stated in the protocol. The data confirm that the presence of AZD4547 does not affect the quantification of Anastrozole. The data are presented in Table 11.

7. CONCLUSION

In summary, the calibration standards, QC sample data and the ISR assessment met the acceptance criteria. The overall maximum precision (%RSD) for Letrozole plasma QC samples at the three concentrations assessed was 3.4%, with a % bias of between -2.4% to 2.2%. The overall maximum precision (RSD%) for Anastrozole plasma QC samples at the three concentrations assessed was 11.7%, with a % bias of between -4.7% to 0.1%.

The data indicated that the method performed reliably during the analysis of Letrozole and Anastrozole plasma study samples. All study samples were analysed within their established stability period. Analytical results for the determination of Letrozole and Anastrozole concentrations in human plasma samples collected in Clinical Study Number D2610C00011 [1] are presented in this report.

8. REFERENCES

[1] AstraZeneca Clinical Protocol Study No D2610C00011
A Randomised Phase IIa Study (with Combination Safety Run-in) to Assess the Safety and Efficacy of AZD4547 in Combination with either Anastrozole or Letrozole versus Exemestane alone in ER Positive Breast Cancer Patients who are Progressing on Current Treatment with Anastrozole or Letrozole – RADICAL

[2] Covance Harrogate Study Number 8264611
Method Transfer for the Determination of Letrozole in Human Plasma, Covance Laboratories Harrogate, final report issued 03 July 2013

[3] Covance Harrogate Study Number 8216406
Validation of an Analytical Procedure for the Determination of Anastrozole in Human Plasma using Protein Precipitation and Liquid Chromatography with Tandem Mass Spectrometric Detection (LC-MS/MS), Covance Laboratories Harrogate, final report issued 25 March 2010

9. TABLES

Table 1 Run Analysis Summary, Clinical Study Number D2610C00011

Run ID	Extraction Date	Assay Date	Run Type	Analyte Name	Assay Name	Regression Status	LLOQ (ng/mL)	ULOQ (ng/mL)	Regression Type	Weighting Factor
1	09-May-2013	09-May-2013	Study samples and Co-administered drug assessment	Letrozole	HB-12-076	Accepted	1	1000	Linear	1/X**2
2	10-May-2013	12-May-2013	ISR run	Letrozole	HB-12-076	Accepted	1	1000	Linear	1/X**2
3	27-Aug-2013	27-Aug-2013	Study samples	Letrozole	HB-12-076	Accepted	1	1000	Linear	1/X**2
4	29-Oct-2013	29-Oct-2013	Study samples	Letrozole	HB-12-076	Accepted	1	1000	Linear	1/X**2
5	29-Oct-2013	29-Oct-2013	Study samples	Anastrozole	HB-09-098	Rejected	1	100	Linear	1/X
6	30-Oct-2013	30-Oct-2013	Co-administered drug assessment	Anastrozole	HB-09-098	Accepted	1	100	Linear	1/X
7	31-Oct-2013	31-Oct-2013	Study samples	Anastrozole	HB-09-098	Accepted	1	100	Linear	1/X
8	01-Nov-2013	03-Nov-2013	ISR run	Anastrozole	HB-09-098	Rejected	1	100	Linear	1/X
9	04-Nov-2013	04-Nov-2013	Trial line	Anastrozole	HB-09-098	Rejected	1	100	Linear	1/X
10	05-Nov-2013	05-Nov-2013	Re-extraction of Run 8	Anastrozole	HB-09-098	Accepted	1	100	Linear	1/X

9.1 Letrozole

Table 2 **Back-Calculated Concentrations of Letrozole in Calibration Standards in Human Plasma**

Assay Date	Run ID	1.00 (ng/mL)	2.00 (ng/mL)	7.50 (ng/mL)	25.0 (ng/mL)	100 (ng/mL)	400 (ng/mL)	800 (ng/mL)	1000 (ng/mL)
09-May-2013	1	0.978	2.07	7.69	25.4	102	397	764	976
12-May-2013	2	0.996	2.00	7.66	25.4	97.1	407	792	987
27-Aug-2013	3	1.03	1.82	8.06	25.7	100	406	801	935
29-Oct-2013	4	0.993	2.05	7.24	25.4	98.9	397	793	1030
Mean		0.999	1.99	7.66	25.5	99.5	402	788	982
S.D.		0.0220	0.114	0.335	0.150	2.05	5.50	16.2	39.0
RSD (%)		2.2	5.7	4.4	0.6	2.1	1.4	2.1	4.0
%Bias		-0.1	-0.5	2.1	2.0	-0.5	0.5	-1.5	-1.8
n		4	4	4	4	4	4	4	4

Table 3 Calibration Curve Parameters of Letrozole in Human Plasma

Assay Date	Run ID	Slope	Intercept	Coefficient of Determination	LLOQ (ng/mL)	ULOQ (ng/mL)	Regression Footnote(s)
09-May-2013	1	0.00687	0.000598	0.9990	1.00	1000	1
12-May-2013	2	0.00684	0.000466	0.9996	1.00	1000	1
27-Aug-2013	3	0.00732	0.000751	0.9965	1.00	1000	1
29-Oct-2013	4	0.0162	0.000758	0.9994	1.00	1000	1

Regression Footnote(s):

1) Resp. = Slope * Conc. + Intercept

Table 4 Determined Concentrations of Letrozole in QC Samples in Human Plasma

Assay Date	Run ID	LoQC 3.00 ng/mL	MeQC 50.0 ng/mL	HiQC 750 ng/mL
09-May-2013	1	2.96	52.8	722
		3.04	52.0	738
12-May-2013	2	2.88	49.5	723
		3.02	50.5	738
27-Aug-2013	3	2.92	51.2	717
		2.76	52.8	723
29-Oct-2013	4	2.99	50.4	737
		3.08	49.7	761
Mean		2.96	51.1	732
S.D.		0.102	1.31	14.3
RSD (%)		3.4	2.6	2.0
Accuracy (%)		98.7	102.2	97.6
%Bias		-1.3	2.2	-2.4
n		8	8	8

Table 5 Incurred Sample Reproducibility Results for Letrozole in Human Plasma (ng/mL)

Sample ID	Analyte ID	Group	Subject	Treatment	Day	Hour	Final Analysis Run ID	Final Original Result	ISR Run ID	ISR Result	%Bias
05116910000011 RAD010001 LET C1D7 Plasma-1 Day 7	Letrozole	1	RAD010001	LET C1D7	7	0.5	1	87.0	2	87.4	0.5
05116910000010 RAD010001 LET C1D7 Plasma-1 Day 7 0h	Letrozole	1	RAD010001	LET C1D7	7	0	1	57.0	2	56.6	-0.7
05116910000012 RAD010001 LET C1D7 Plasma-1 Day 7 1h	Letrozole	1	RAD010001	LET C1D7	7	1	1	79.4	2	77.6	-2.3
05116910000013 RAD010001 LET C1D7 Plasma-1 Day 7 2h	Letrozole	1	RAD010001	LET C1D7	7	2	1	72.1	2	73.1	1.4
05116910000014 RAD010001 LET C1D7 Plasma-1 Day 7 3h	Letrozole	1	RAD010001	LET C1D7	7	3	1	69.9	2	70.0	0.1
05116910000015 RAD010001 LET C1D7 Plasma-1 Day 7 4h	Letrozole	1	RAD010001	LET C1D7	7	4	1	64.7	2	61.2	-5.6
05116910000016 RAD010001 LET C1D7 Plasma-1 Day 7 5h	Letrozole	1	RAD010001	LET C1D7	7	5	1	64.5	2	64.7	0.3
05116910000017 RAD010001 LET C1D7 Plasma-1 Day 7 6h	Letrozole	1	RAD010001	LET C1D7	7	6	1	66.1	2	65.1	-1.5
05116910000018 RAD010001 LET C1D7 Plasma-1 Day 7 8h	Letrozole	1	RAD010001	LET C1D7	7	8	1	62.7	2	63.0	0.5
05116910000029 RAD010001 LET MD7 Plasma-1 Day 7 0.5h	Letrozole	1	RAD010001	LET MD7	7	0.5	1	90.8	2	89.6	-1.3
05116910000028 RAD010001 LET MD7 Plasma-1 Day 7 0h	Letrozole	1	RAD010001	LET MD7	7	0	1	66.8	2	65.4	-2.1
05116910000030 RAD010001 LET MD7 Plasma-1 Day 7 1h	Letrozole	1	RAD010001	LET MD7	7	1	1	80.3	2	79.8	-0.6
05116910000031 RAD010001 LET MD7 Plasma-1 Day 7 2h	Letrozole	1	RAD010001	LET MD7	7	2	1	83.7	2	82.8	-1.1

Table 5 Continued: Incurred Sample Reproducibility Results for Letrozole in Human Plasma (ng/mL)

Sample ID	Analyte ID	Group	Subject	Treatment	Day	Hour	Final Analysis Run ID	Final Original Result	ISR Run ID	ISR Result	%Bias
05116910000032 RAD010001 LET MD7 Plasma-1 Day 7 3h	Letrozole	1	RAD010001	LET MD7	7	3	1	80.9	2	82.6	2.1
05116910000033 RAD010001 LET MD7 Plasma-1 Day 7 4h	Letrozole	1	RAD010001	LET MD7	7	4	1	75.5	2	72.2	-4.5
05116910000034 RAD010001 LET MD7 Plasma-1 Day 7 5h	Letrozole	1	RAD010001	LET MD7	7	5	1	71.3	2	72.5	1.7
05116910000035 RAD010001 LET MD7 Plasma-1 Day 7 6h	Letrozole	1	RAD010001	LET MD7	7	6	1	74.0	2	75.7	2.3
05116910000036 RAD010001 LET MD7 Plasma-1 Day 7 8h	Letrozole	1	RAD010001	LET MD7	7	8	1	85.9	2	88.0	2.4
05116910000102 RAD010003 LET MD7 Plasma-1 Day 7 1h	Letrozole	1	RAD010003	LET MD7	7	1	1	58.5	2	60.0	2.5
05116910000103 RAD010003 LET MD7 Plasma-1 Day 7 2h	Letrozole	1	RAD010003	LET MD7	7	2	1	55.8	2	56.0	0.4
05116910000104 RAD010003 LET MD7 Plasma-1 Day 7 3h	Letrozole	1	RAD010003	LET MD7	7	3	1	51.8	2	51.0	-1.6
05116910000105 RAD010003 LET MD7 Plasma-1 Day 7 4h	Letrozole	1	RAD010003	LET MD7	7	4	1	52.4	2	52.2	-0.4
05116910000106 RAD010003 LET MD7 Plasma-1 Day 7 5h	Letrozole	1	RAD010003	LET MD7	7	5	1	51.2	2	50.3	-1.8
05116910000107 RAD010003 LET MD7 Plasma-1 Day 7 6h	Letrozole	1	RAD010003	LET MD7	7	6	1	48.2	2	49.9	3.5
05116910000108 RAD010003 LET MD7 Plasma-1 Day 7 8h	Letrozole	1	RAD010003	LET MD7	7	8	1	47.9	2	47.1	-1.7

Table 6 Quantification of Letrozole in the Presence of AZD4547 (Run 1)

Quality control level (ng/mL)	Co-administered drug	Spiked concentration of co-adminstered drug (ng/mL)	Replicate	Observed concentration (ng/mL)
3	AZD4547	2000	1	3.22
			2	3.08
			3	3.01
			4	3.06
			5	2.97
			6	2.99
Mean (ng/mL)				3.06
Standard deviation (n-1)				0.0909
RSD (%)				3.0
Accuracy (%)				102.0

9.2 Anastrazole

Table 7 Back-Calculated Concentrations of Anastrozole in Calibration Standards in Human Plasma

Assay Date	Run ID	1.00 (ng/mL)	2.00 (ng/mL)	5.00 (ng/mL)	7.00 (ng/mL)	10.0 (ng/mL)	30.0 (ng/mL)	50.0 (ng/mL)	80.0 (ng/mL)	100 (ng/mL)
30-Oct-2013	6	1.10	2.02	4.96	6.78	9.74	31.4	51.5	83.5	98.4
		0.899								95.8
31-Oct-2013	7	0.913	*2.45	5.23	7.57	10.4	29.0	49.4	80.8	102
		0.955								97.7
05-Nov-2013	10	0.996	2.09	4.97	7.43	10.1	31.8	50.5	80.0	103
		0.854								94.4
Mean		0.953	2.06	5.05	7.26	10.1	30.7	50.5	81.4	98.6
S.D.		0.0869		0.153	0.422	0.330	1.51	1.05	1.83	3.38
RSD (%)		9.1		3.0	5.8	3.3	4.9	2.1	2.2	3.4
%Bias		-4.7	3.0	1.0	3.7	1.0	2.3	1.0	1.8	-1.4
n		6	2	3	3	3	3	3	3	6

Reason Deactivated

* Failed Acceptance Criteria

Table 8 Calibration Curve Parameters of Anastrozole in Human Plasma

Assay Date	Run ID	Slope	Intercept	Coefficient of Determination	LLOQ (ng/mL)	ULOQ (ng/mL)	Regression Footnote(s)
30-Oct-2013	6	0.0214	0.000979	0.9986	1.00	100	1
31-Oct-2013	7	0.0251	0.00379	0.9993	1.00	100	1
05-Nov-2013	10	0.0248	0.00285	0.9984	1.00	100	1

Regression Footnote(s):

1) Resp. = Slope * Conc. + Intercept

Table 9 **Determined Concentrations of Anastrozole in QC Samples in Human Plasma**

Assay Date	Run ID	LoQC 3.00 ng/mL	MeQC 40.0 ng/mL	HiQC 70.0 ng/mL
30-Oct-2013	6	2.70	35.8	65.2
		&2.39	&32.4	65.0
31-Oct-2013	7	2.99	41.3	71.6
		&7.40#	40.2	79.1
05-Nov-2013	10	3.28	39.8	70.8
		2.96	40.2	68.6
Mean		3.62 [2.86]	38.3	70.1
S.D.		1.88 [0.335]	3.45	5.22
RSD (%)		51.9 [11.7]	9.0	7.4
Accuracy (%)		120.7 [95.3]	95.8	100.1
%Bias		20.7 [-4.7]	-4.3	0.1
n		6 [5]	6	6

& > 15% Bias

Excluded as an outlier according to Grubbs test. Values in parentheses presented excluding the outlier

Table 10 Incurred Sample Reproducibility Results for Anastrozole in Human Plasma (ng/mL)

Sample ID	Analyte ID	Group	Subject	Treatment	Day	Hour	Final Analysis Run ID	Final Original Result	ISR Run ID	ISR Result	%Bias
05116910000059 RAD010002 ANA MD7 Plasma-1 Day 7 3h	Anastrozole	1	RAD010002	ANA MD7	7	3	7	53.1	10	46.7	-12.8
05116910000060 RAD010002 ANA MD7 Plasma-1 Day 7 4h	Anastrozole	1	RAD010002	ANA MD7	7	4	7	43.4	10	43.2	-0.5
05116910000062 RAD010002 ANA MD7 Plasma-1 Day 7 6h	Anastrozole	1	RAD010002	ANA MD7	7	6	7	35.0	10	32.3	-8.0
05116910000063 RAD010002 ANA MD7 Plasma-1 Day 7 8h	Anastrozole	1	RAD010002	ANA MD7	7	8	7	36.4	10	36.8	1.1
05116910000112 RAD010004 ANA C1D7 Plasma-1 Day 7 2h	Anastrozole	1	RAD010004	ANA C1D7	7	2	7	41.7	10	39.3	-5.9
05116910000113 RAD010004 ANA C1D7 Plasma-1 Day 7 3h	Anastrozole	1	RAD010004	ANA C1D7	7	3	7	39.4	10	38.1	-3.4
05116910000114 RAD010004 ANA C1D7 Plasma-1 Day 7 4h	Anastrozole	1	RAD010004	ANA C1D7	7	4	7	34.9	10	35.9	2.8
05116910000115 RAD010004 ANA C1D7 Plasma-1 Day 7 5h	Anastrozole	1	RAD010004	ANA C1D7	7	5	7	34.1	10	34.0	-0.3
05116910000117 RAD010004 ANA C1D7 Plasma-1 Day 7 8h	Anastrozole	1	RAD010004	ANA C1D7	7	8	7	35.6	10	37.3	4.7
05116910000128 RAD010004 ANA MD7 Plasma-1 Day 7 0.5h	Anastrozole	1	RAD010004	ANA MD7	7	0.5	7	33.0	10	31.0	-6.3
E8705568-1 RAD010004 ANA MD7 Plasma-1 Day 7 1h	Anastrozole	1	RAD010004	ANA MD7	7	1	7	42.1	10	40.8	-3.1
05116910000130 RAD010004 ANA MD7 Plasma-1 Day 7 2h	Anastrozole	1	RAD010004	ANA MD7	7	2	7	38.5	10	39.9	3.6

Table 10 Continued Incurred Sample Reproducibility Results for Anastrozole in Human Plasma (ng/mL)

Sample ID	Analyte ID	Group	Subject	Treatment	Day	Hour	Final Analysis Run ID	Final Original Result	ISR Run ID	ISR Result	%Bias
05116910000131 RAD010004 ANA MD7 Plasma-1 Day 7 3h	Anastrozole	1	RAD010004	ANA MD7	7	3	7	39.4	10	39.4	0.0
05116910000135 RAD010004 ANA MD7 Plasma-1 Day 7 8h	Anastrozole	1	RAD010004	ANA MD7	7	8	7	34.2	10	35.5	3.7
E8706799-4 RAD010005 ANA C1D7 Plasma-1 Day 7 0.5h	Anastrozole	1	RAD010005	ANA C1D7	7	0.5	7	35.8	10	36.2	1.1
E8706800-4 RAD010005 ANA C1D7 Plasma-1 Day 7 1h	Anastrozole	1	RAD010005	ANA C1D7	7	1	7	45.6	10	41.9	-8.5
E8706801-4 RAD010005 ANA C1D7 Plasma-1 Day 7 2h	Anastrozole	1	RAD010005	ANA C1D7	7	2	7	41.5	10	40.8	-1.7
E8706802-5 RAD010005 ANA C1D7 Plasma-1 Day 7 3h	Anastrozole	1	RAD010005	ANA C1D7	7	3	7	42.5	10	38.8	-9.1
E8706806-2 RAD010005 ANA C1D7 Plasma-1 Day 7 8h	Anastrozole	1	RAD010005	ANA C1D7	7	8	7	38.2	10	34.0	-11.6
05116910000164 RAD010005 ANA MD7 Plasma-1 Day 7 0.5h	Anastrozole	1	RAD010005	ANA MD7	7	0.5	7	45.9	10	42.7	-7.2
05116910000165 RAD010005 ANA MD7 Plasma-1 Day 7 1h	Anastrozole	1	RAD010005	ANA MD7	7	1	7	46.3	10	39.7	-15.3
05116910000166 RAD010005 ANA MD7 Plasma-1 Day 7 2h	Anastrozole	1	RAD010005	ANA MD7	7	2	7	35.6	10	38.8	8.6
05116910000167 RAD010005 ANA MD7 Plasma-1 Day 7 3h	Anastrozole	1	RAD010005	ANA MD7	7	3	7	39.8	10	36.7	-8.1
05116910000171 RAD010005 ANA MD7 Plasma-1 Day 7 8h	Anastrozole	1	RAD010005	ANA MD7	7	8	7	36.1	10	37.6	4.1

Table 11 Quantification of Anastrozole in the Presence of AZD4547 (Run 6)

Quality control level (ng/mL)	Co-administered drug	Spiked concentration of co-adminstered drug (ng/mL)	Replicate	Observed concentration (ng/mL)
3	AZD4547	2000	1	2.37
			2	2.76
			3	2.8
			4	2.49
			5	2.8
			6	3.16
Mean (ng/mL)				2.73
Standard deviation (n-1)				0.277
RSD (%)				10.1
Accuracy (%)				91.0

10. LETROZOLE FIGURES

Figure 1 Plasma Blank with Added Internal Standard Chromatogram (Run 2)

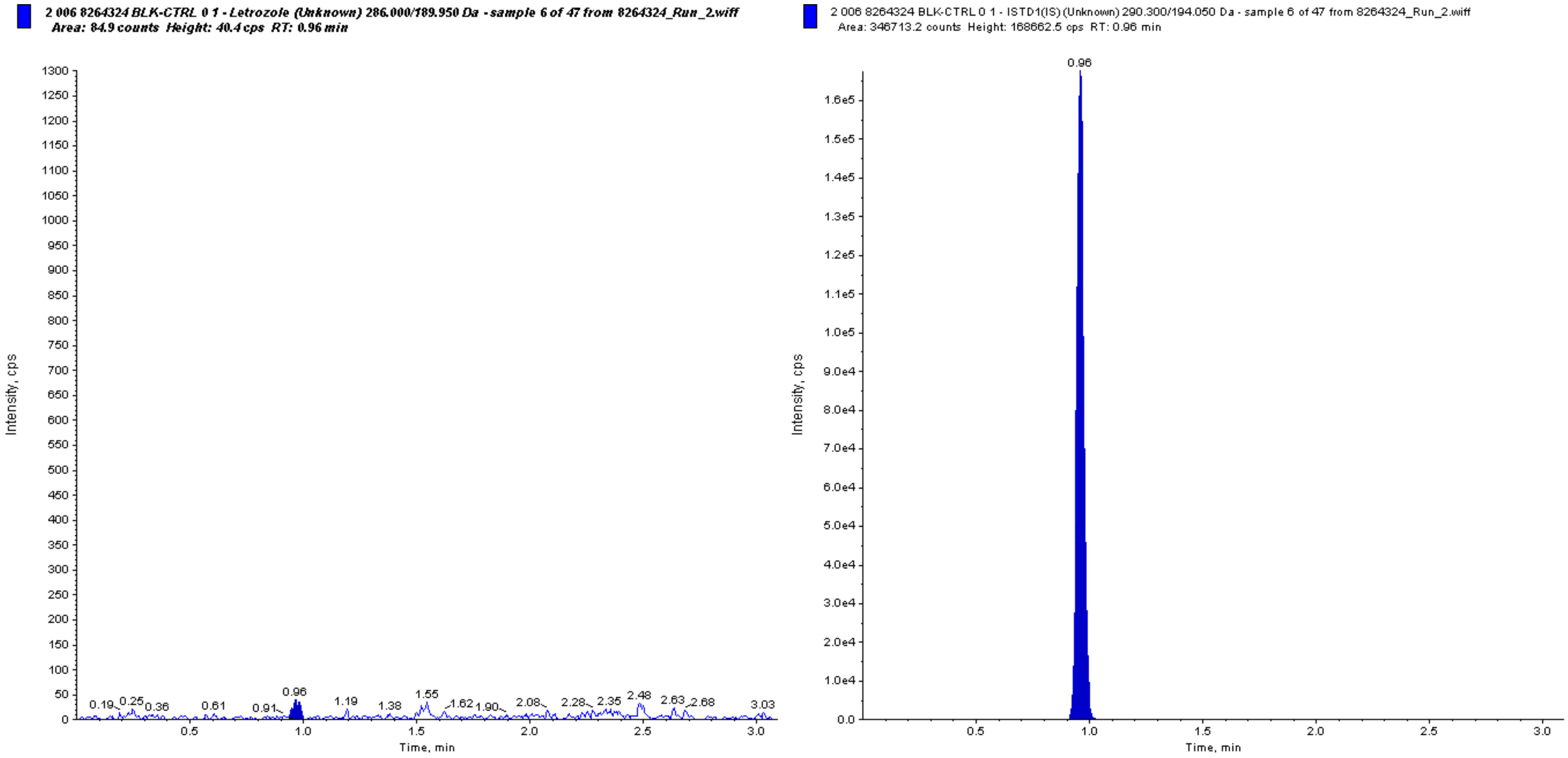

Figure 2 LLOQ Plasma Calibration Standard (1 ng/mL) Chromatogram (Run 2)

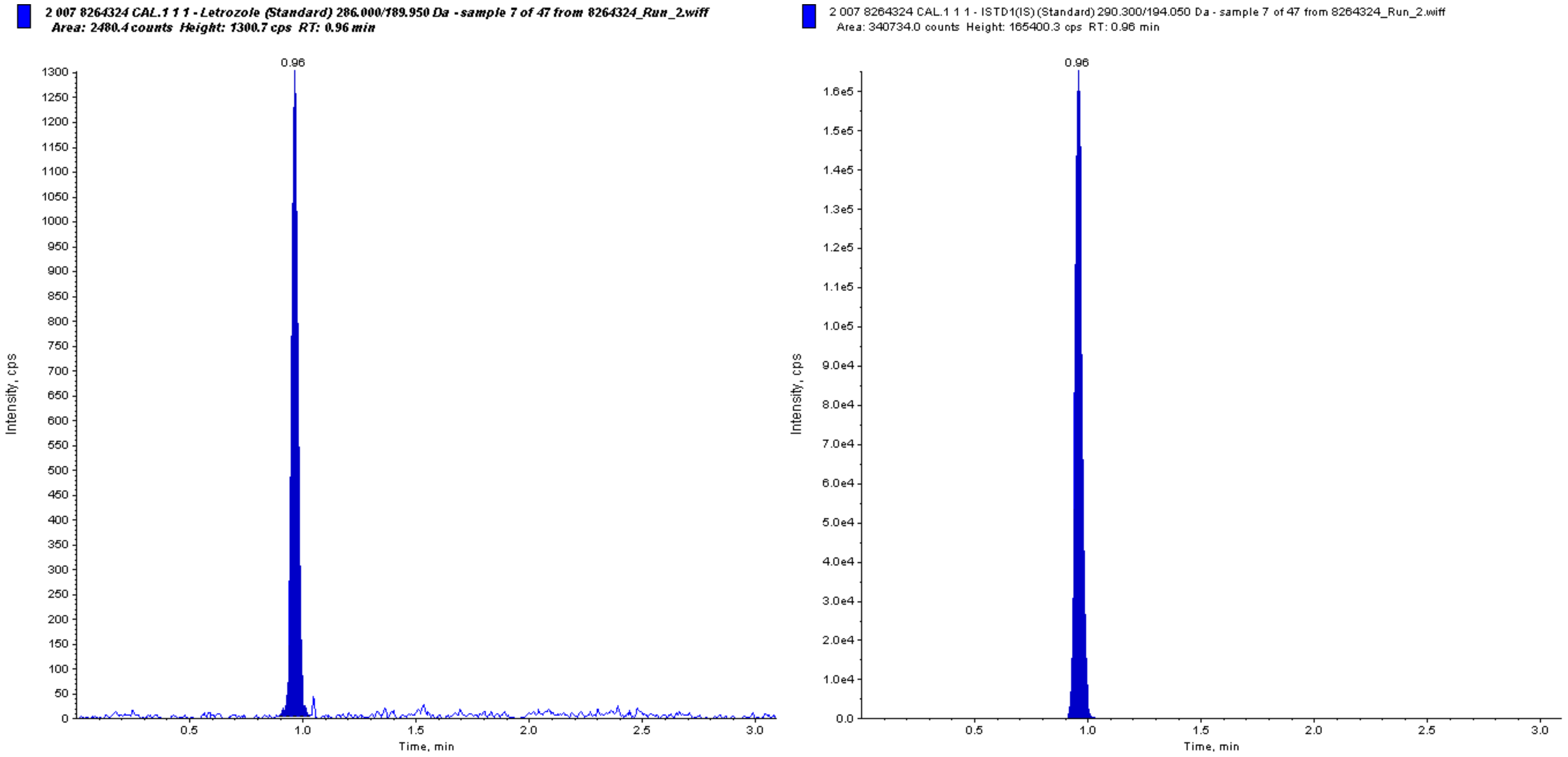

Figure 3 ULOQ Plasma Calibration Standard (1000 ng/mL) Chromatogram (Run 2)

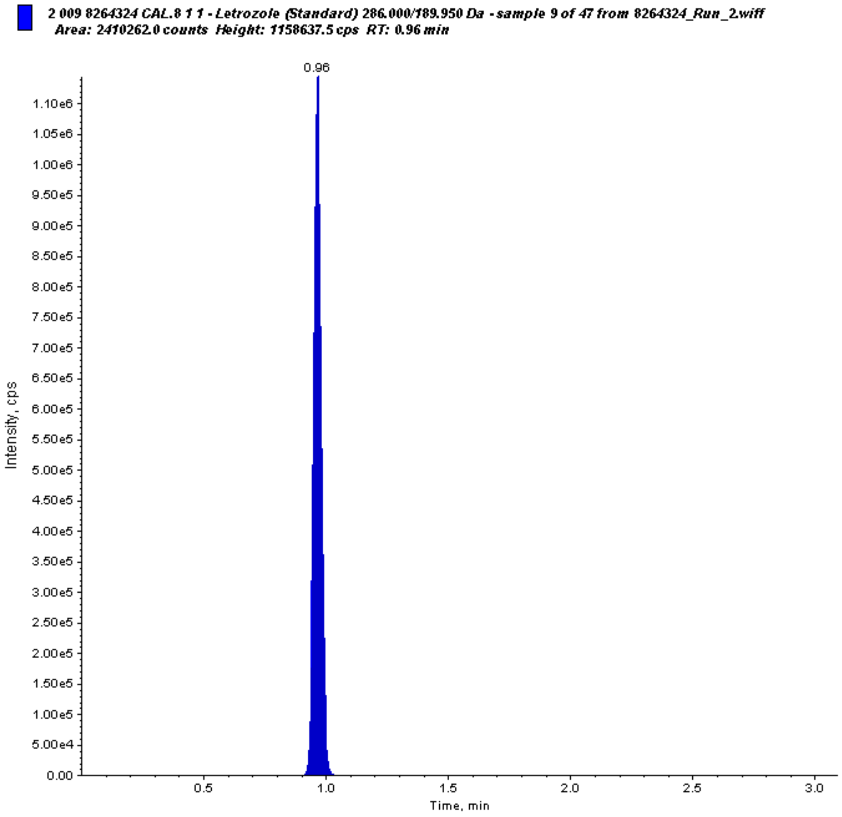

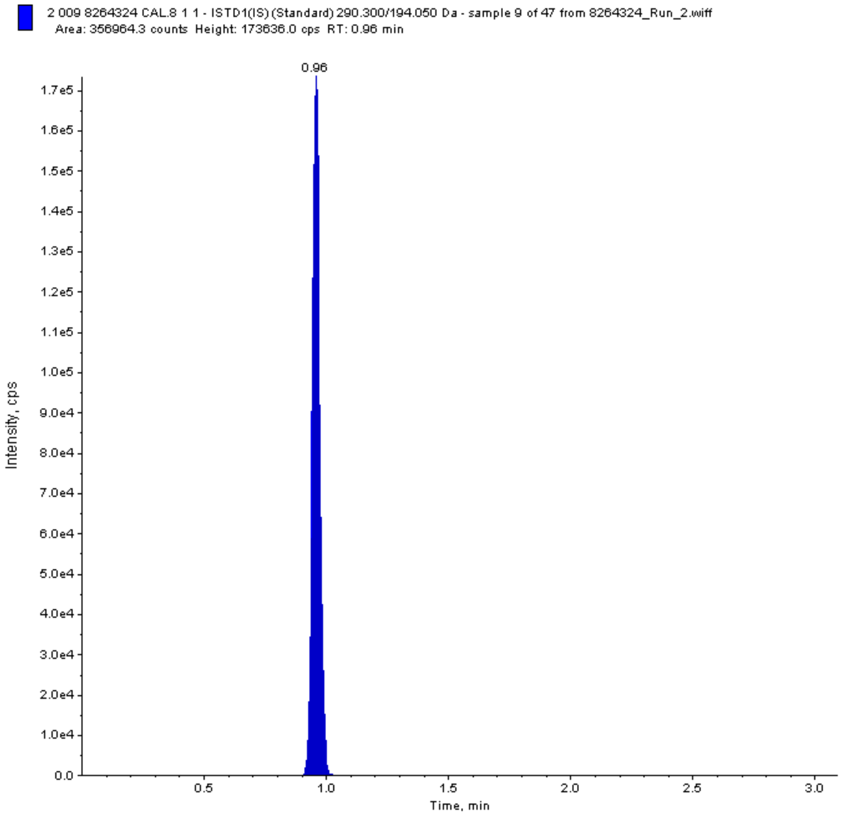

Figure 4 LoQC Plasma Sample (3 ng/mL) Chromatogram (Run 2)

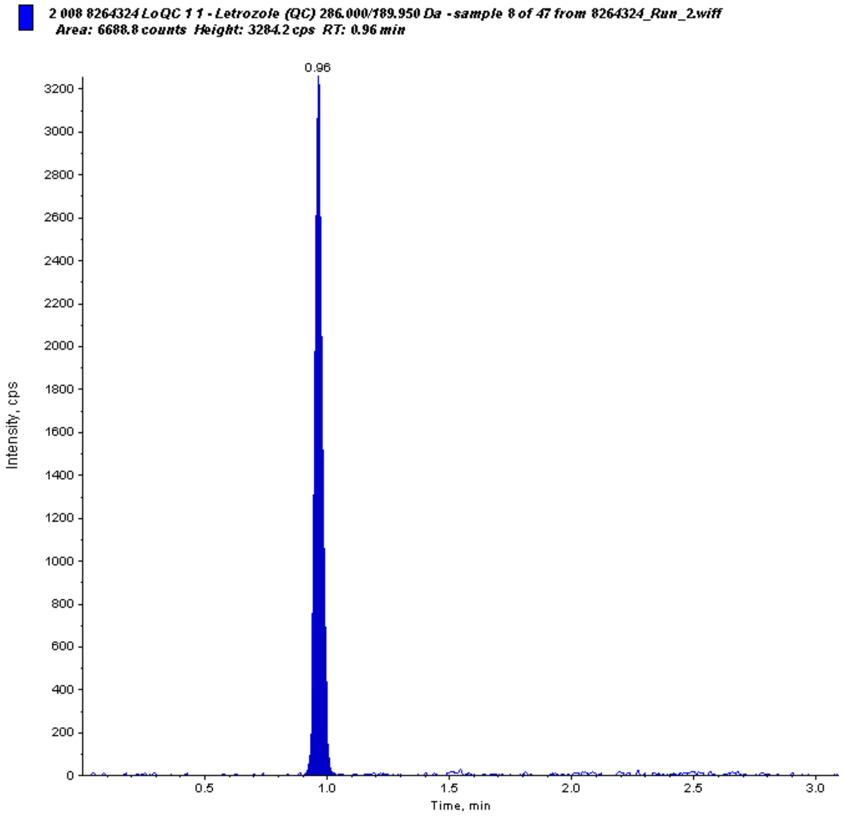

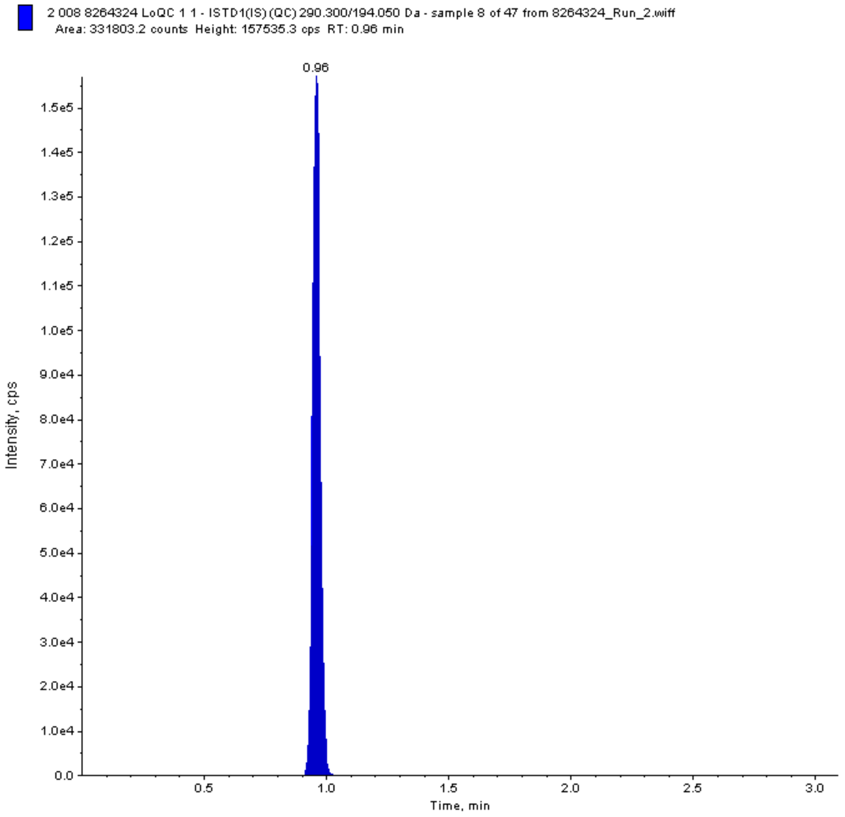

Figure 5 MeQC Plasma Sample (50 ng/mL) Chromatogram (Run 2)

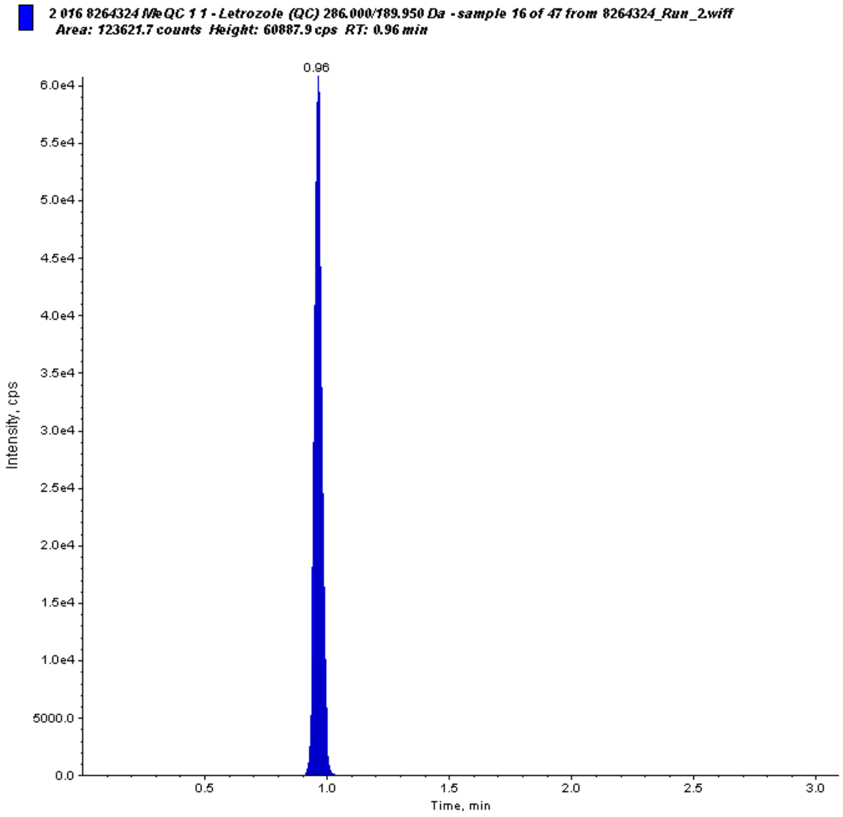

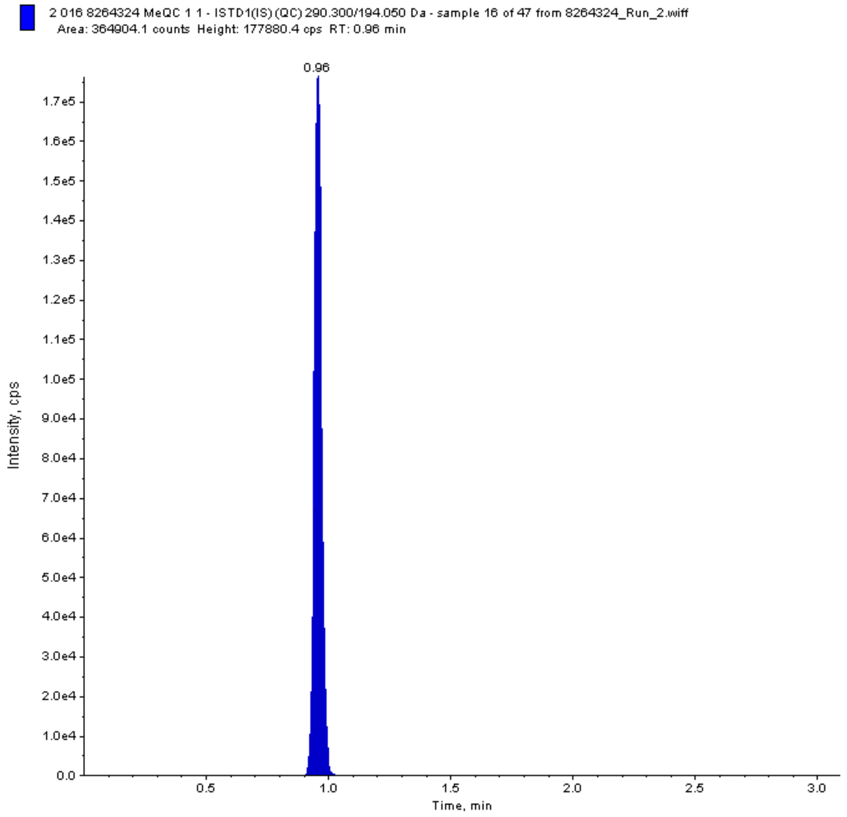

Figure 6 HiQC Plasma Sample (750 ng/mL) Chromatogram (Run 2)

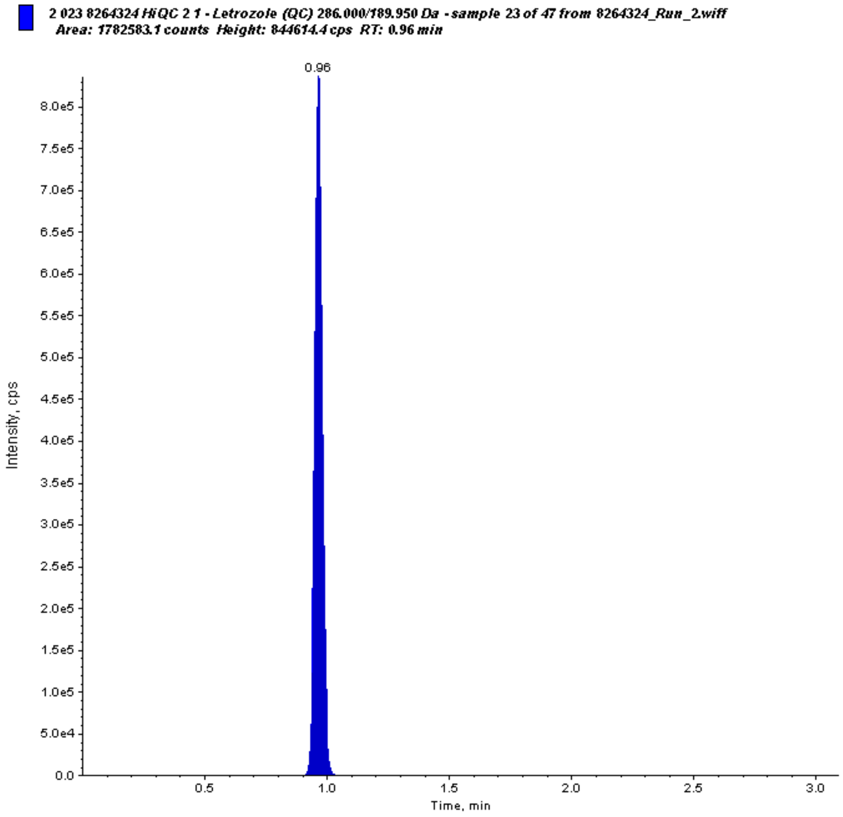

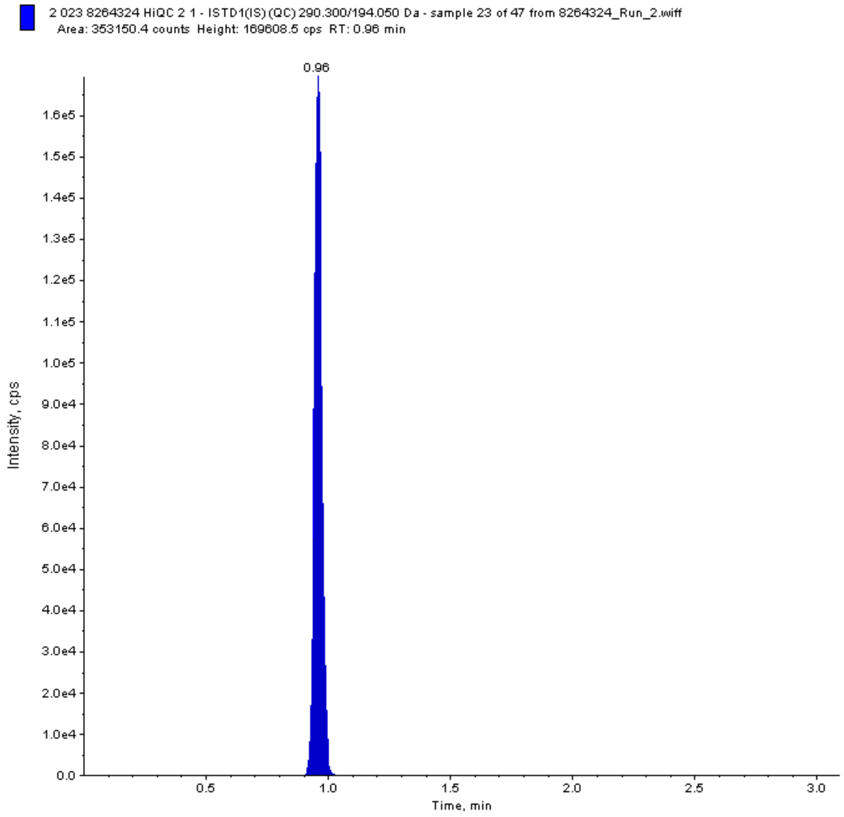

Figure 7 **Plasma Sample Collected From Subject RAD040001, Monotherapy Day 7, 0.5 Hour Sample Chromatogram (Run 4)**

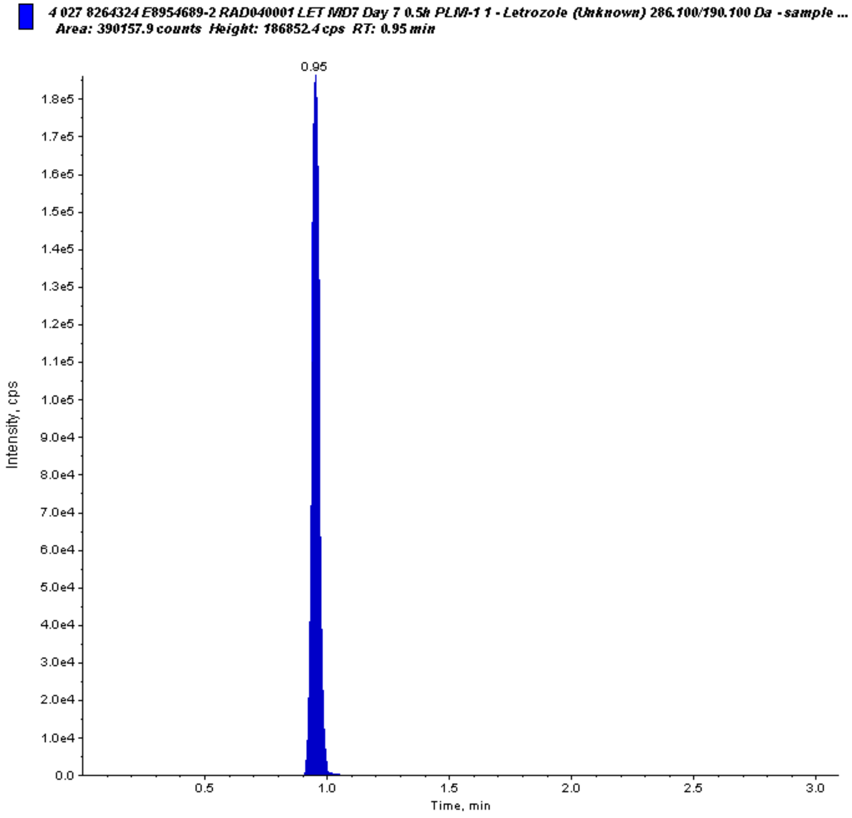

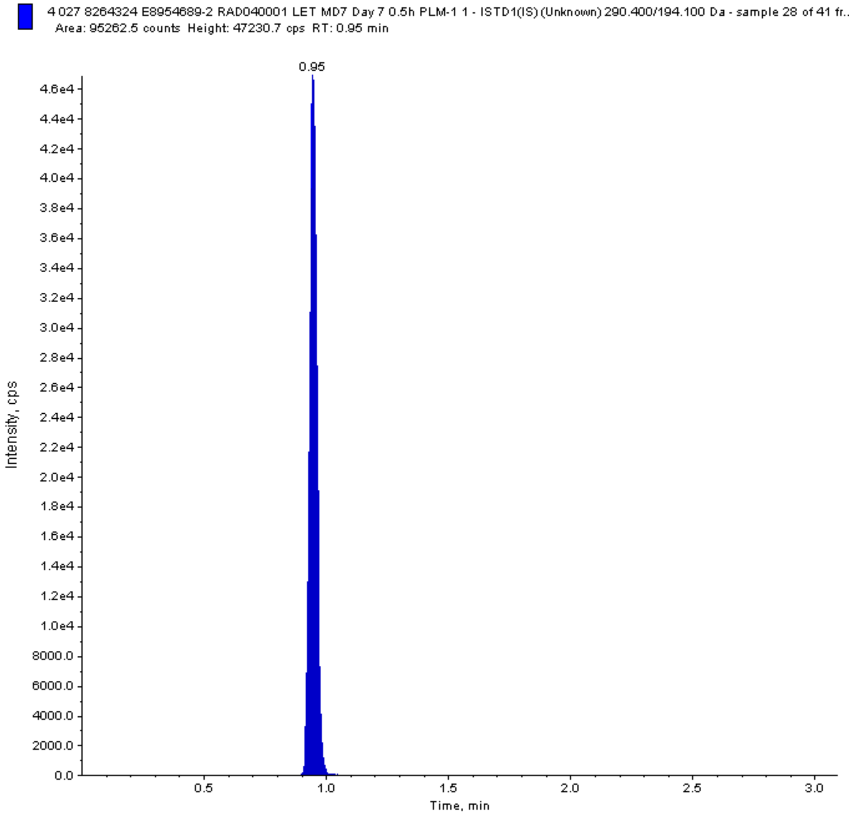

Figure 8 Plasma Sample Collected From Subject RAD040001, Monotherapy Day 7, 8 Hour Sample Chromatogram (Run 4)

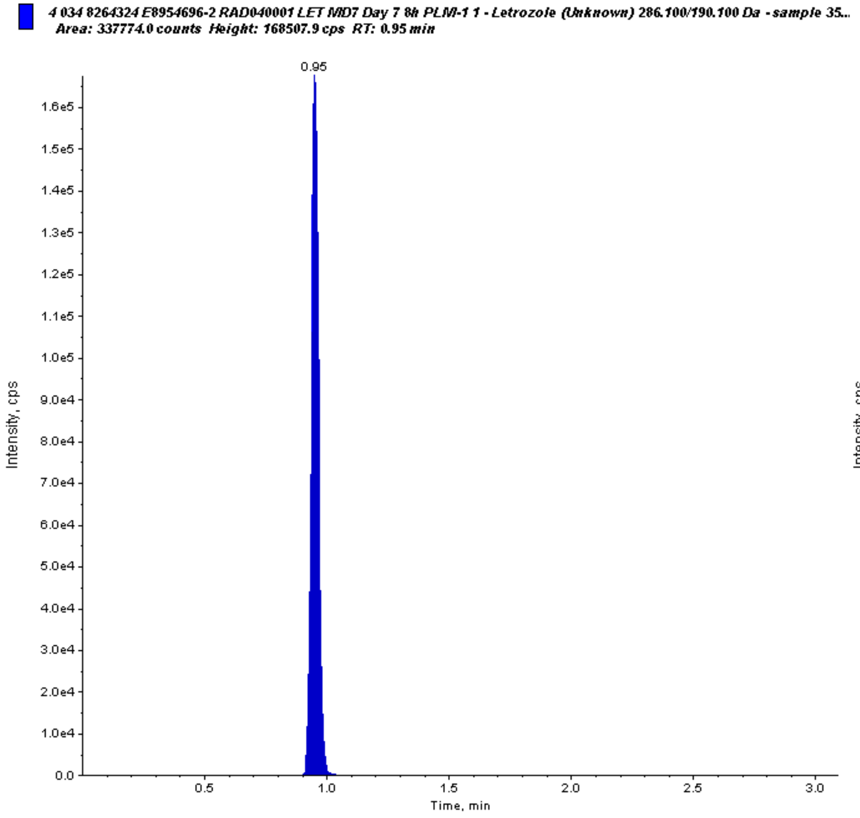

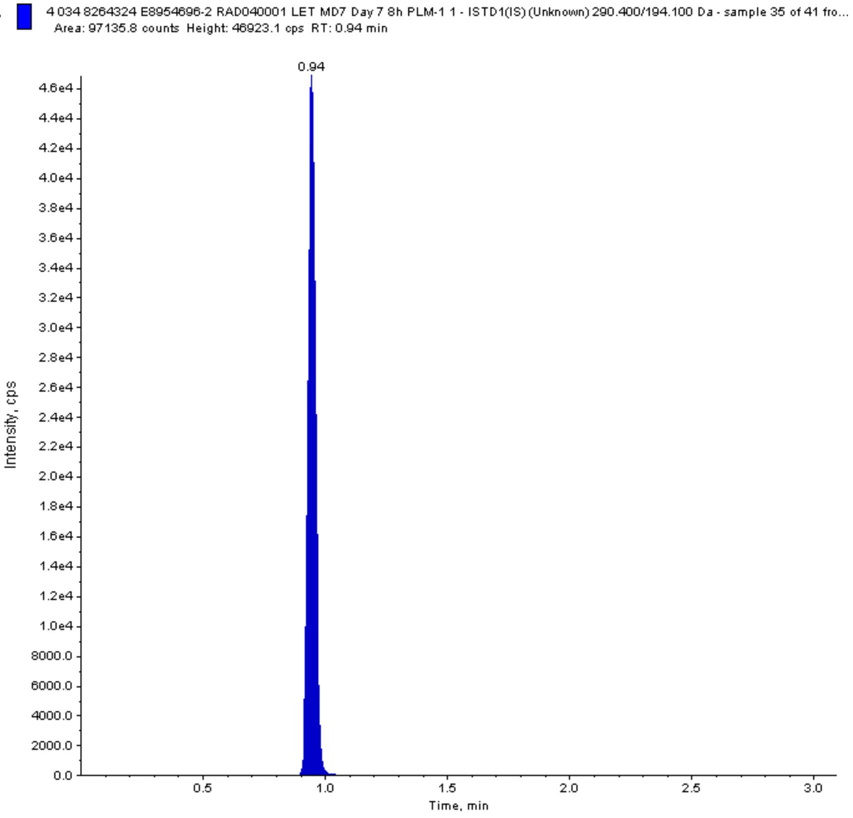

Figure 9 **Plasma Sample Collected From Subject RAD040001, Cycle 1 Day 7, 1 Hour Sample Chromatogram (Run 4)**

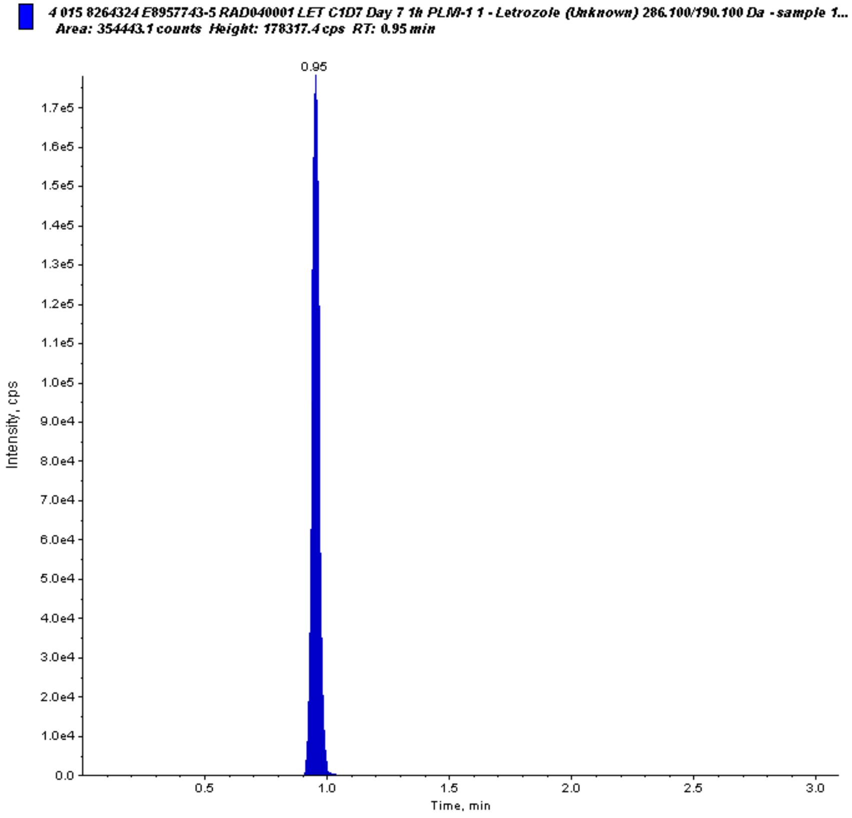

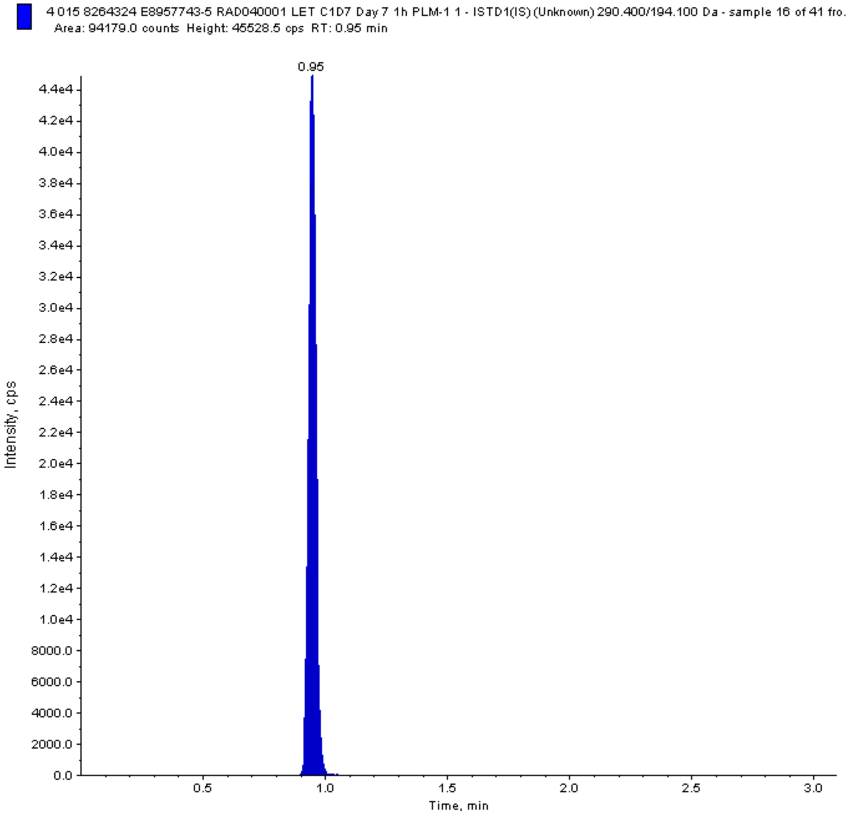

Figure 10 **Plasma Sample Collected From Subject RAD040001, Cycle 1 Day 7, 6 Hour Sample Chromatogram (Run 4)**

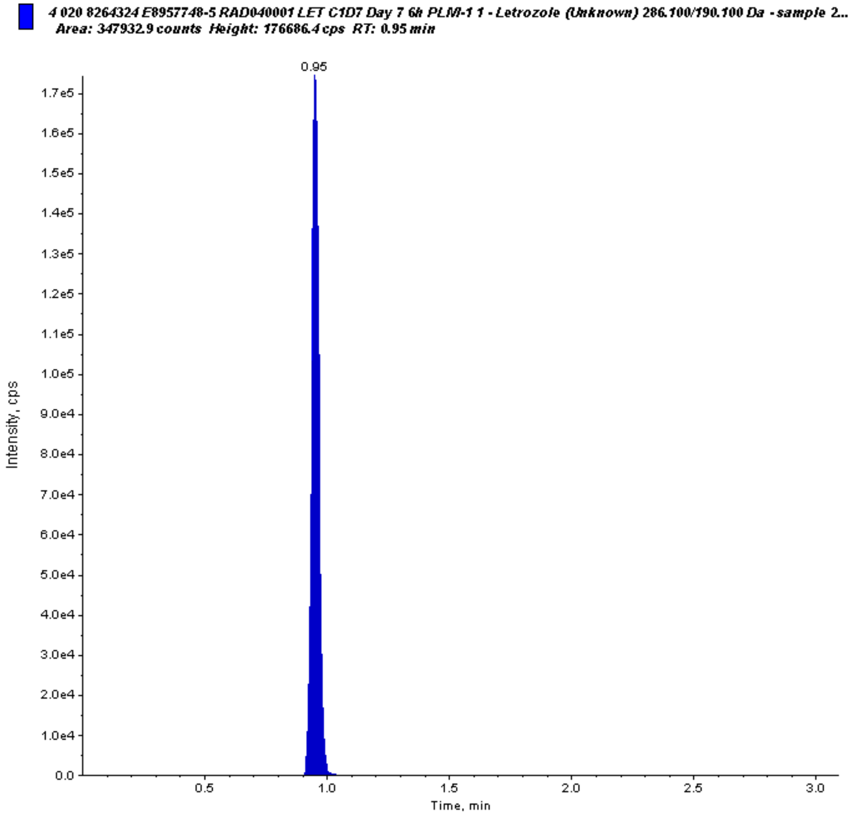

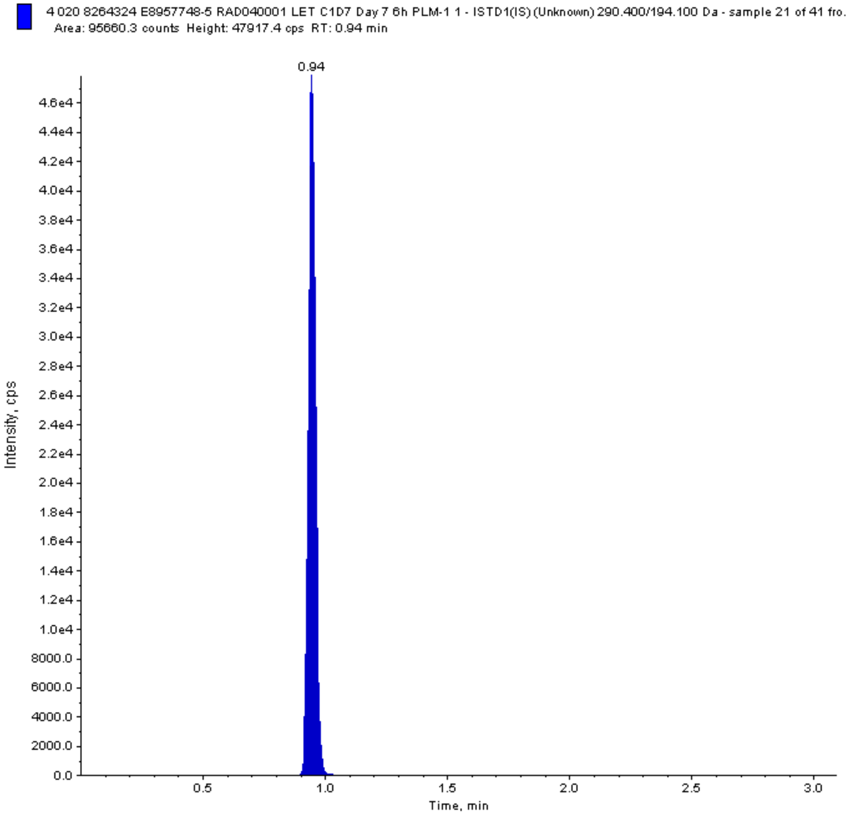

Figure 11 Plasma Sample Collected From Subject RAD010001, Monotherapy Day 7, 3 Hour Sample Chromatogram (Run 1)

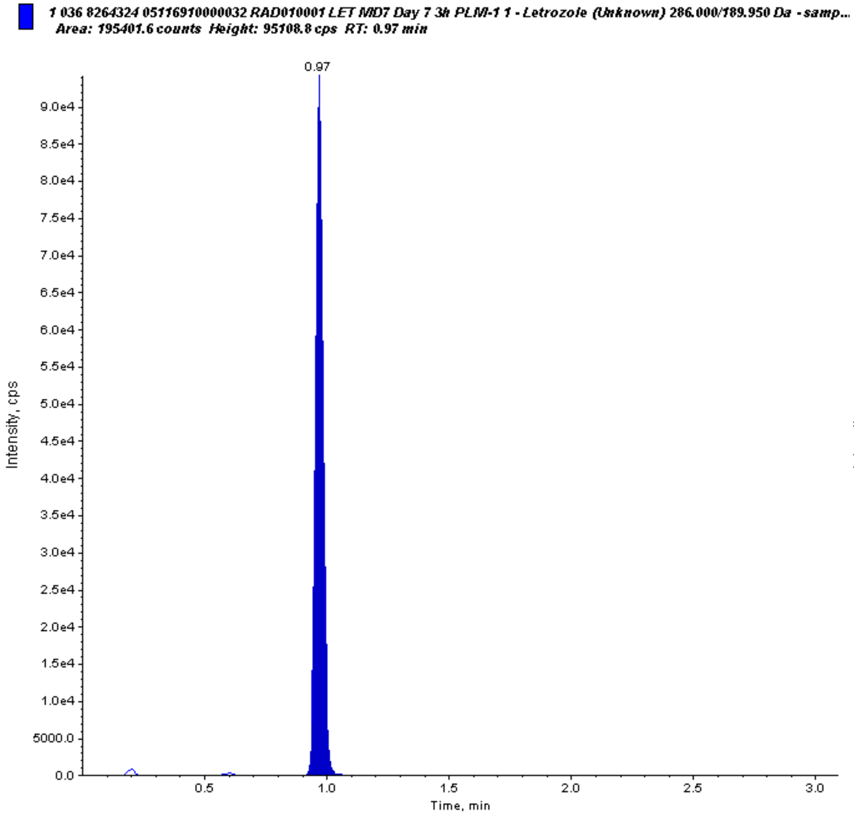

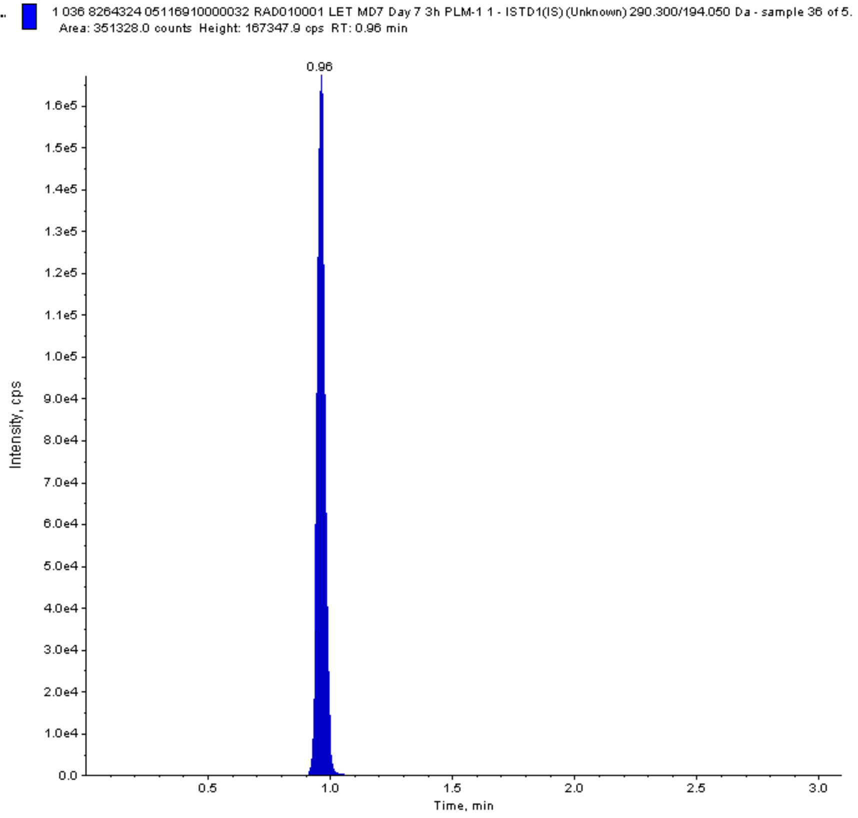

Figure 12 Plasma Sample Collected From Subject RAD010001, Monotherapy Day 7, 5 Hour Sample Chromatogram (Run 1)

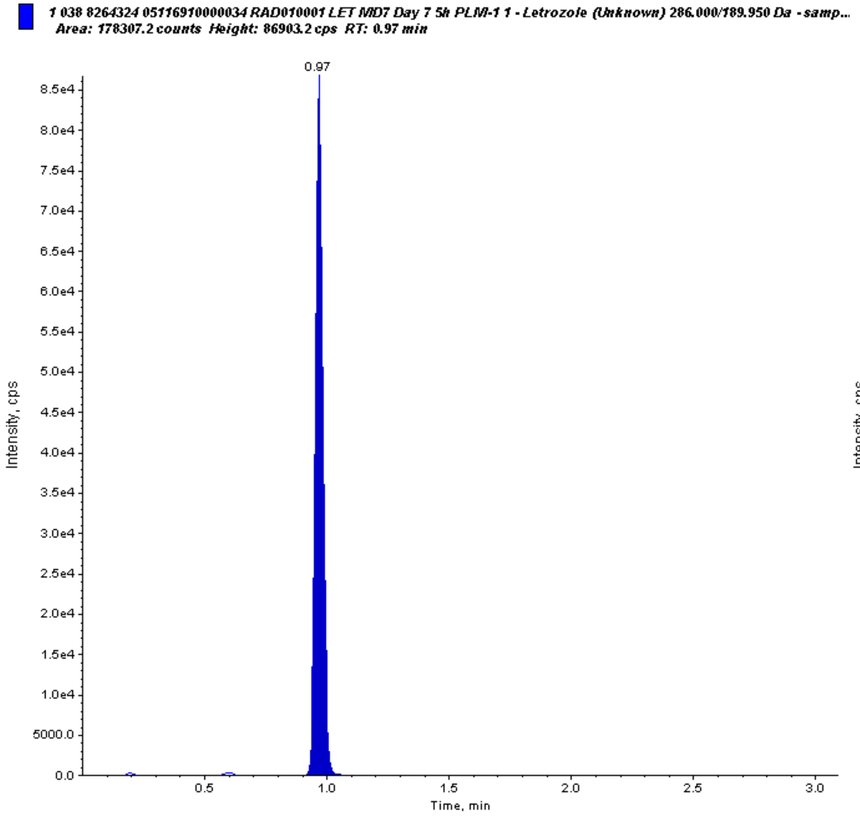

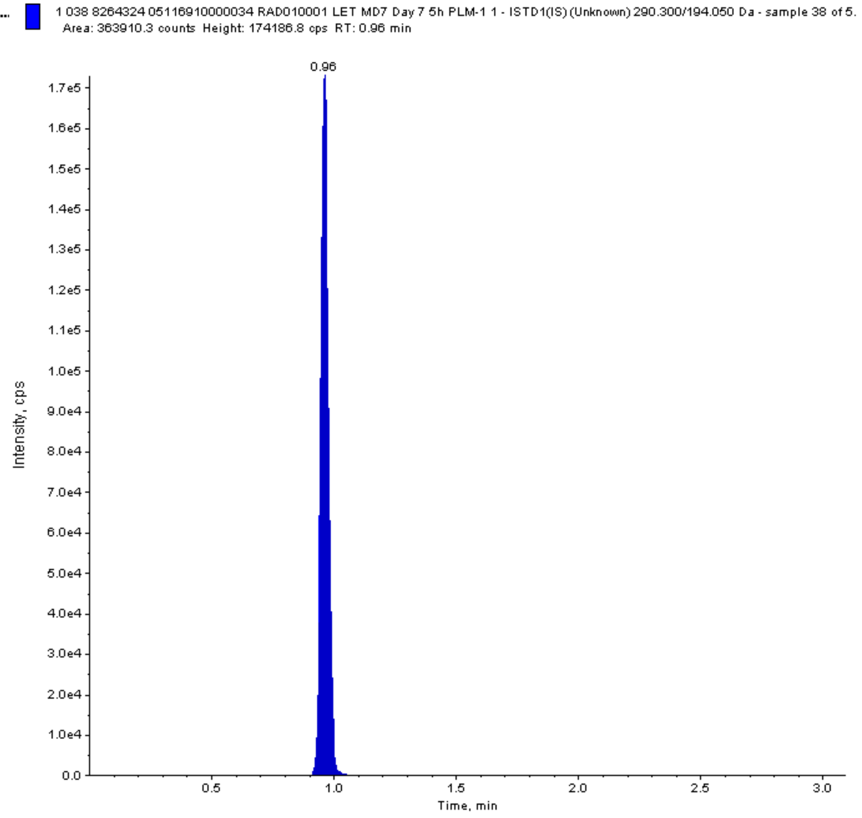

Figure 13 **Plasma Sample Collected From Subject RAD010001, Cycle 1 Day 7, 0 Hour Sample Chromatogram (Run 1)**

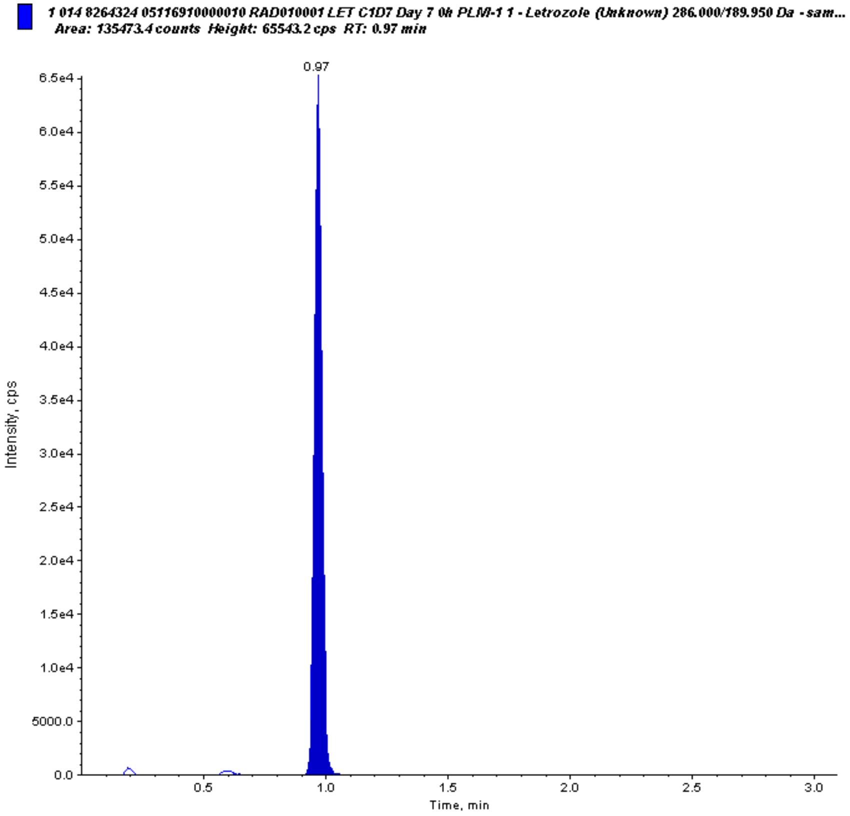

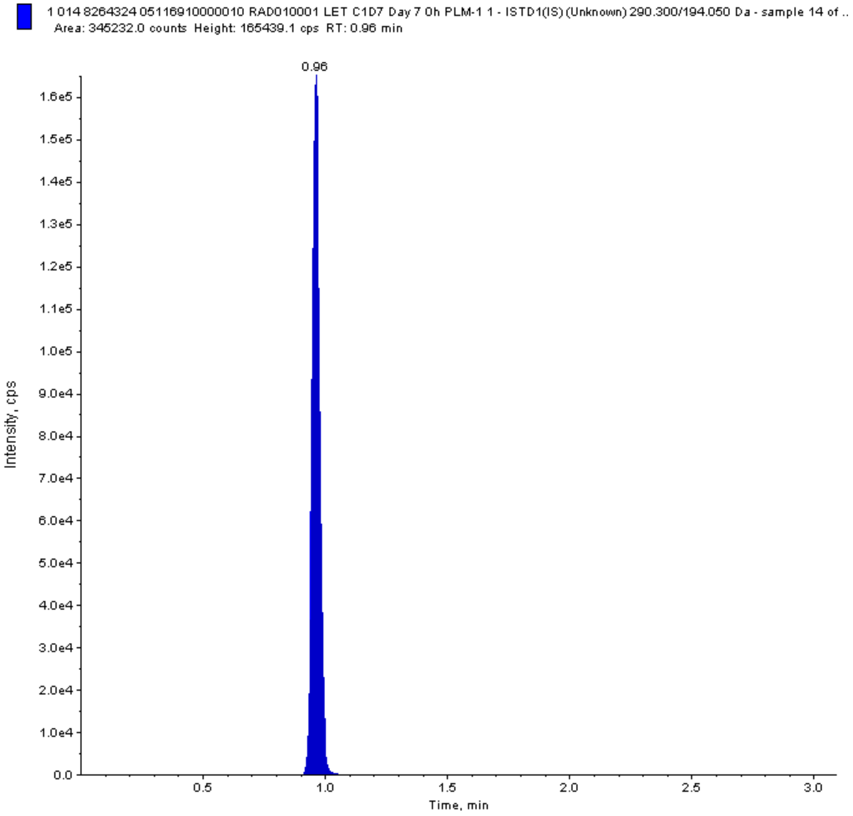

Figure 14 Plasma Sample Collected From Subject RAD010001, Cycle 1 Day 7, 4 Hour Sample Chromatogram (Run 1)

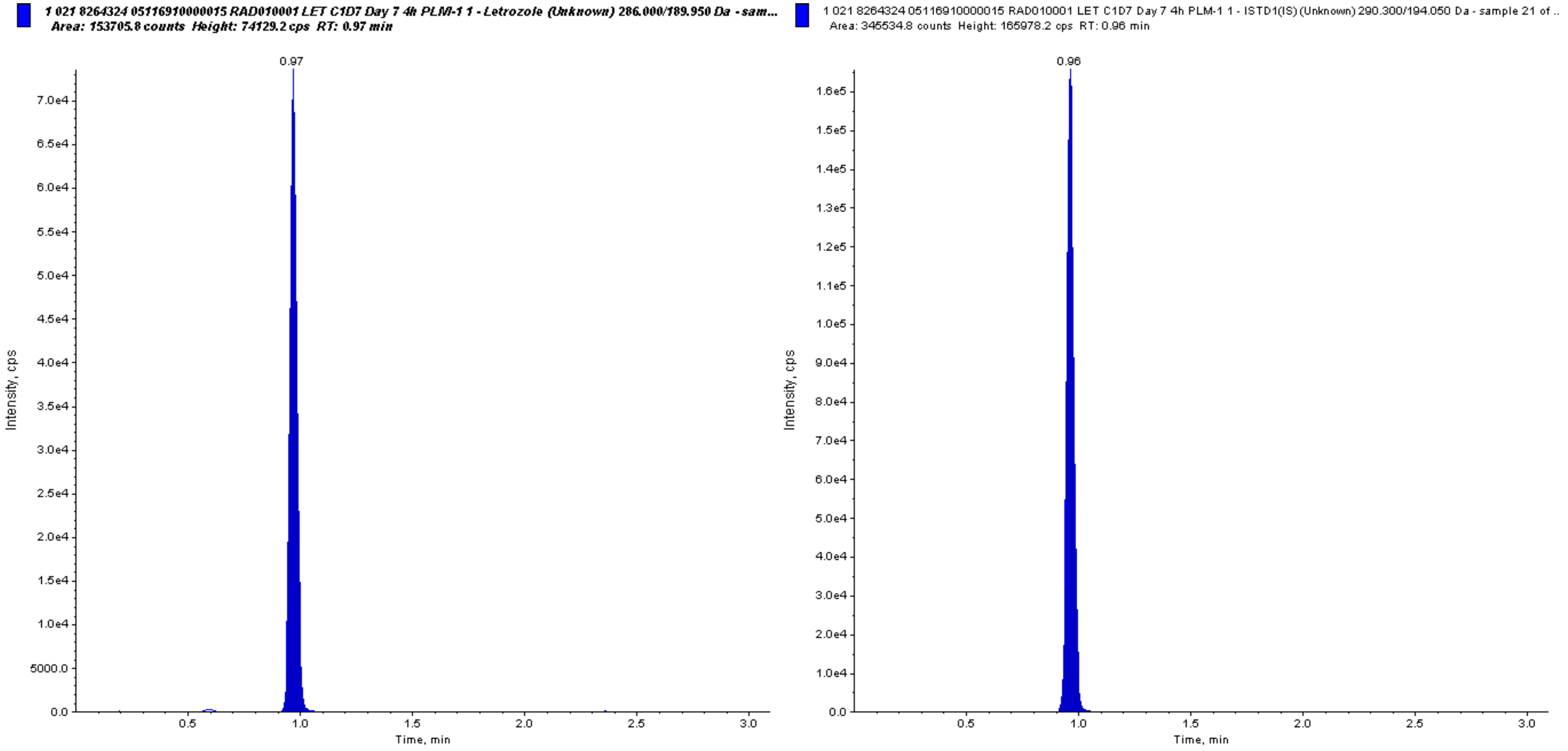

11. ANASTROZOLE FIGURES

Figure 15 Plasma Blank with Added Internal Standard Chromatogram (Run 10)

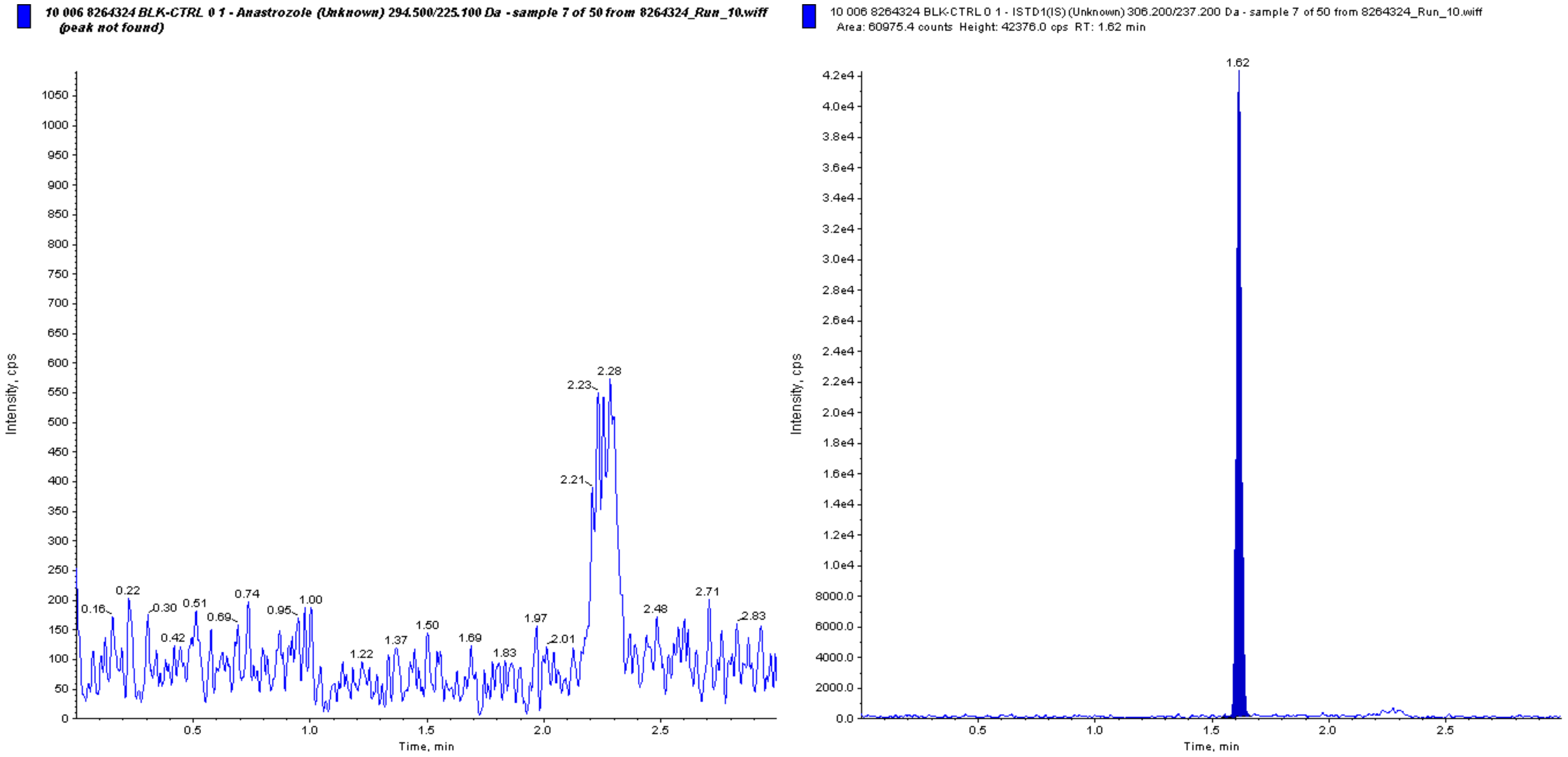

Figure 16 LLOQ Plasma Calibration Standard (1 ng/mL) Chromatogram (Run 10)

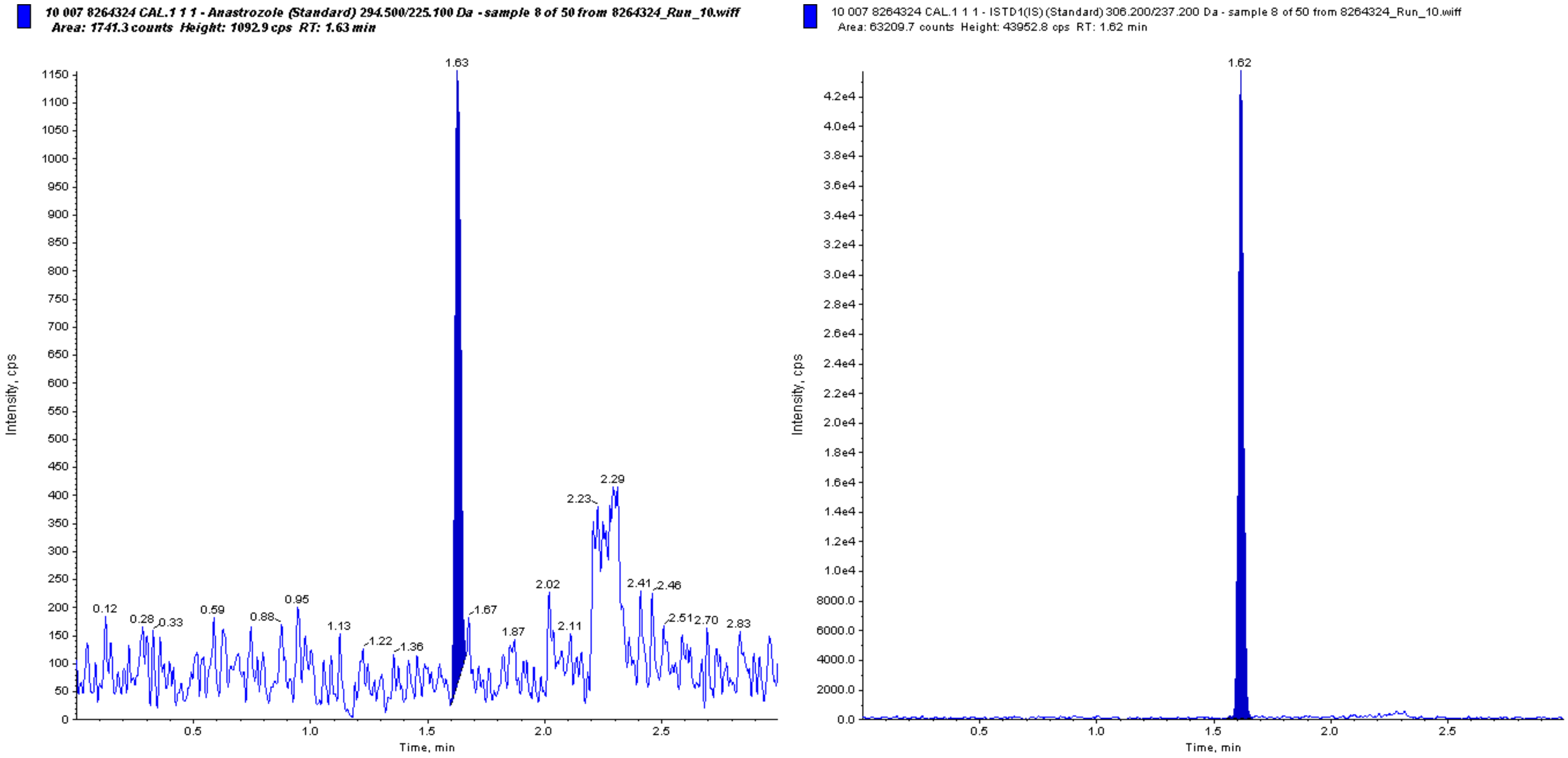

Figure 17 ULOQ Plasma Calibration Standard (100 ng/mL) Chromatogram (Run 10)

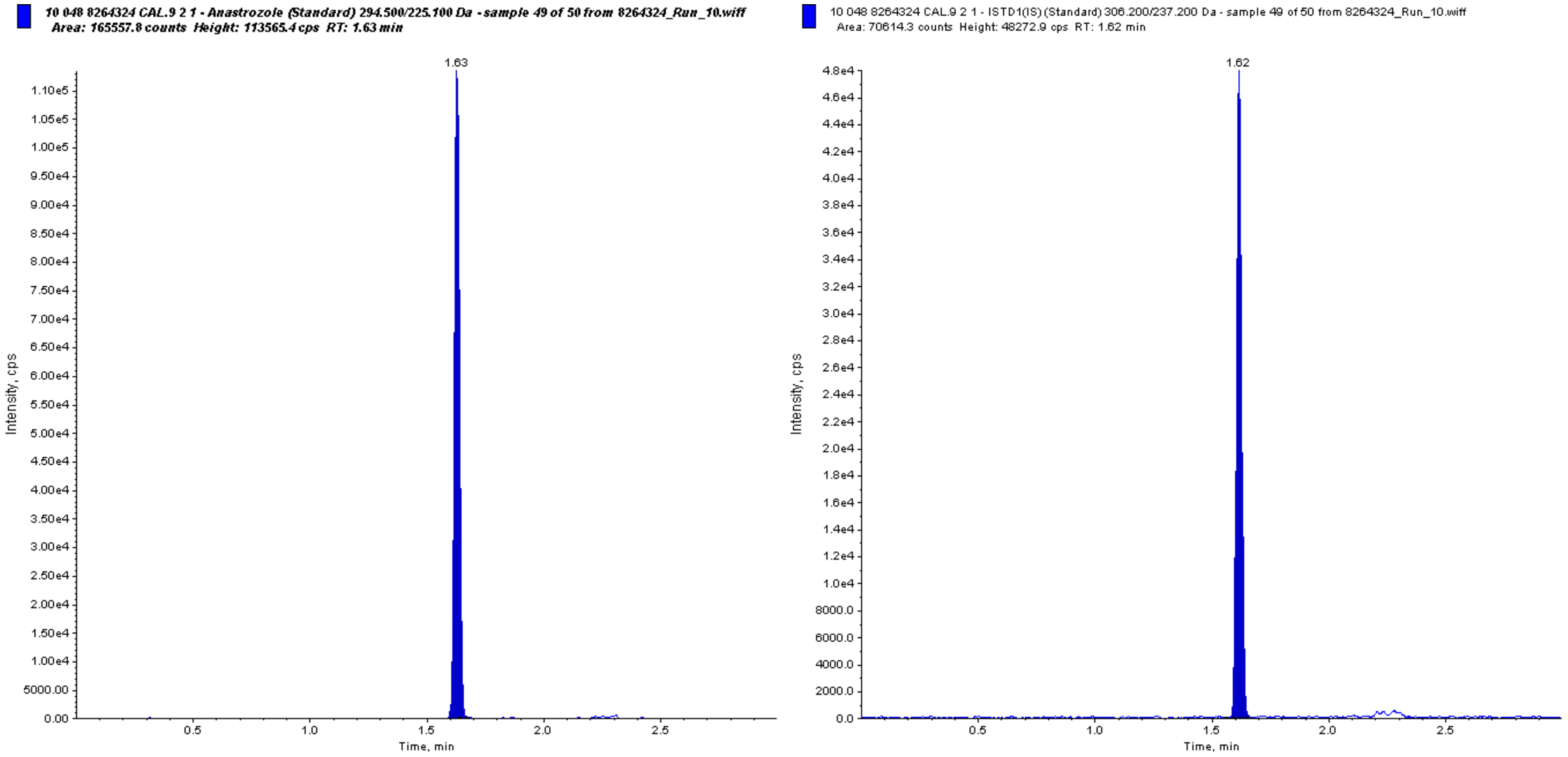

Figure 18 LoQC Plasma Sample (3ng/mL) Chromatogram (Run 10)

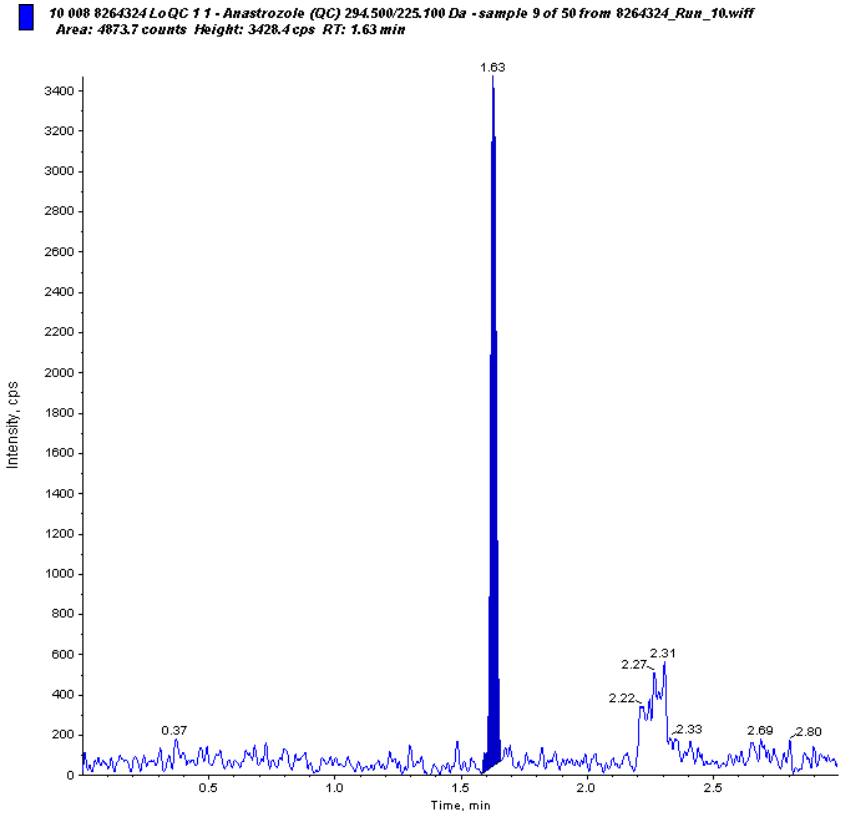

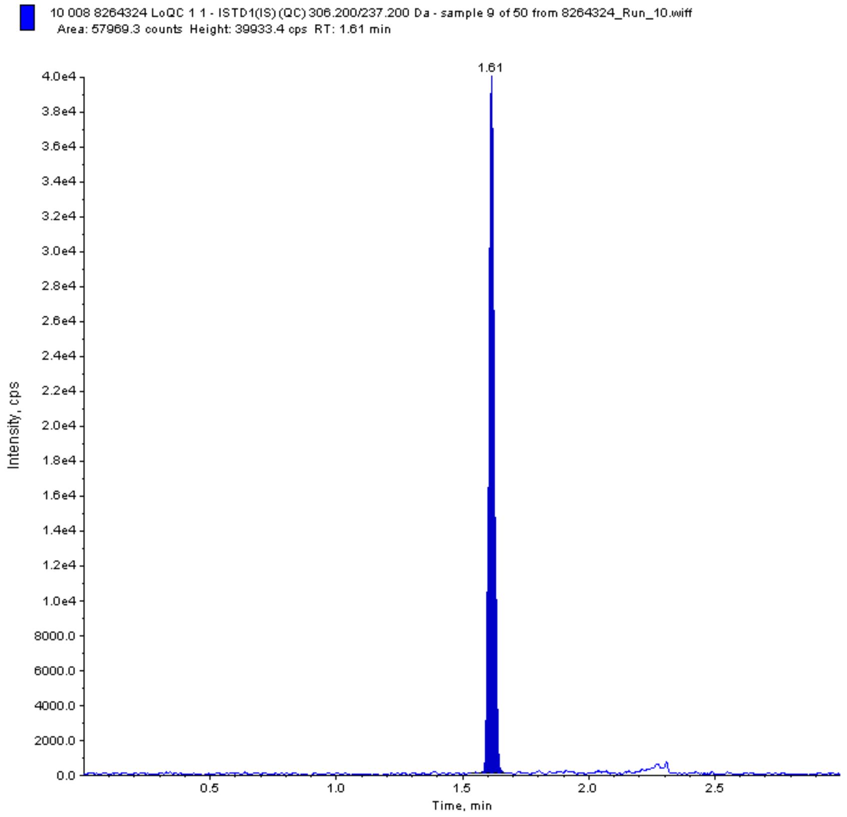

Figure 19 MeQC Plasma Sample (40 ng/mL) Chromatogram (Run 10)

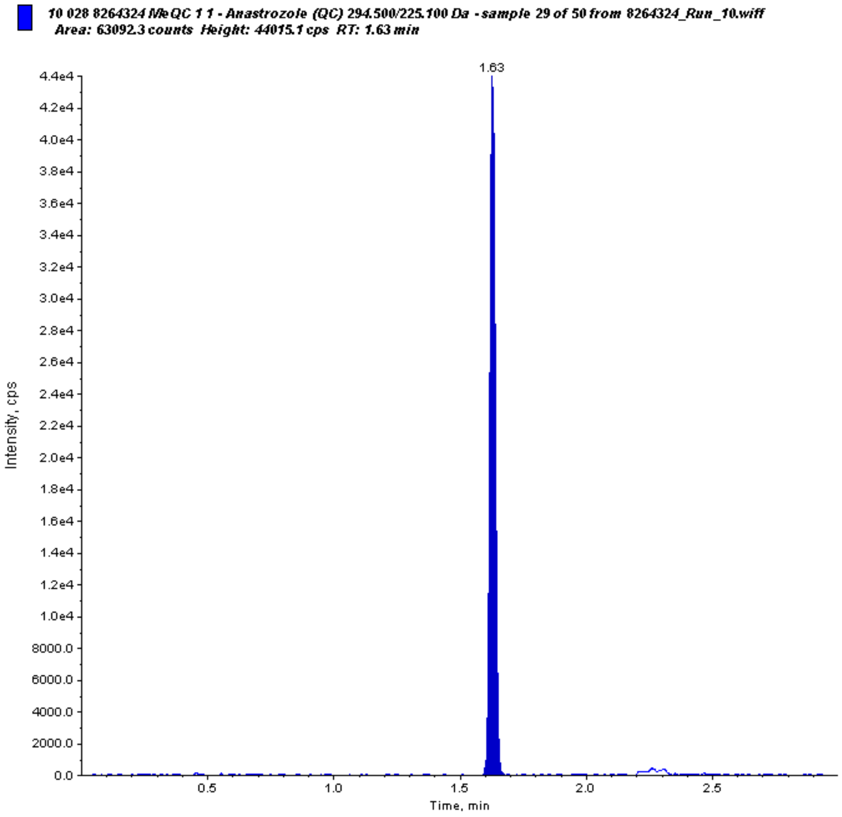

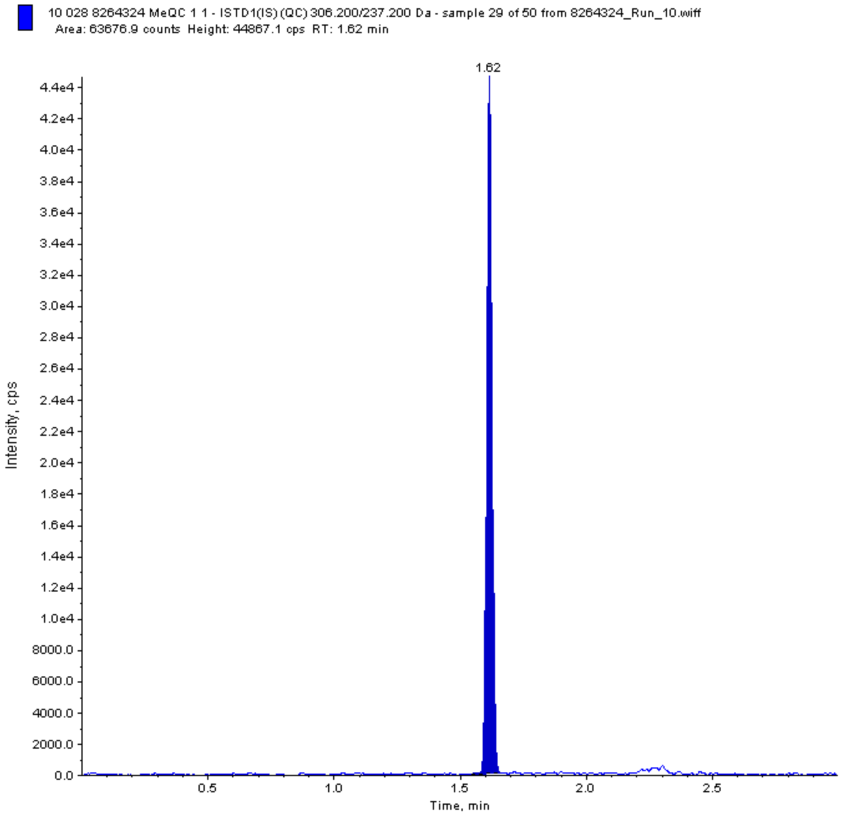

Figure 20 HiQC Plasma Sample (70 ng/mL) Chromatogram (Run 10)

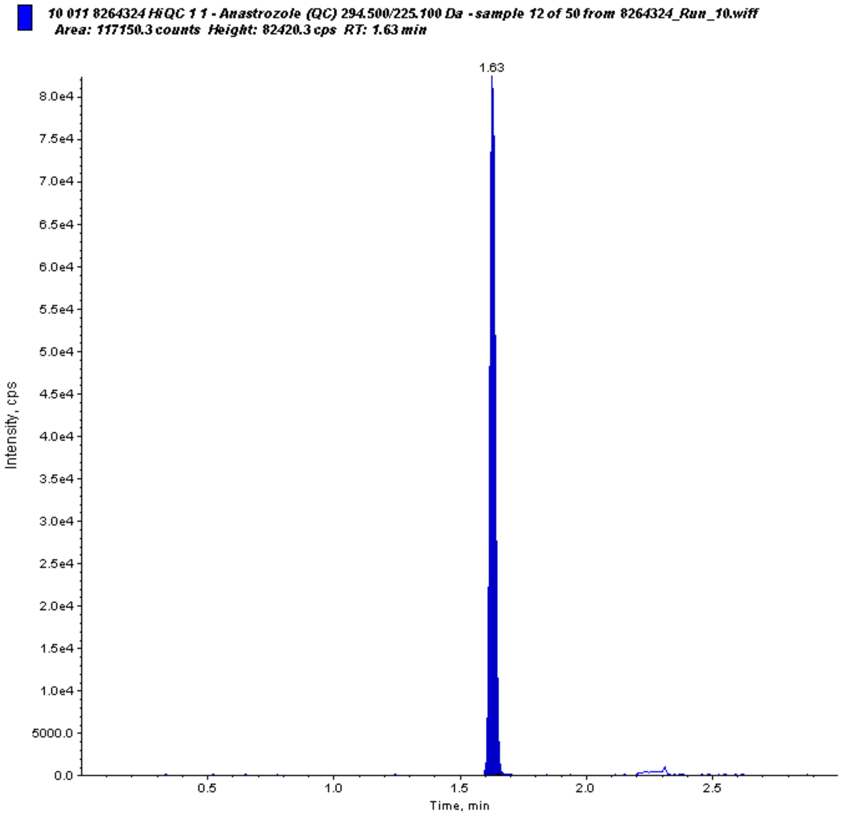

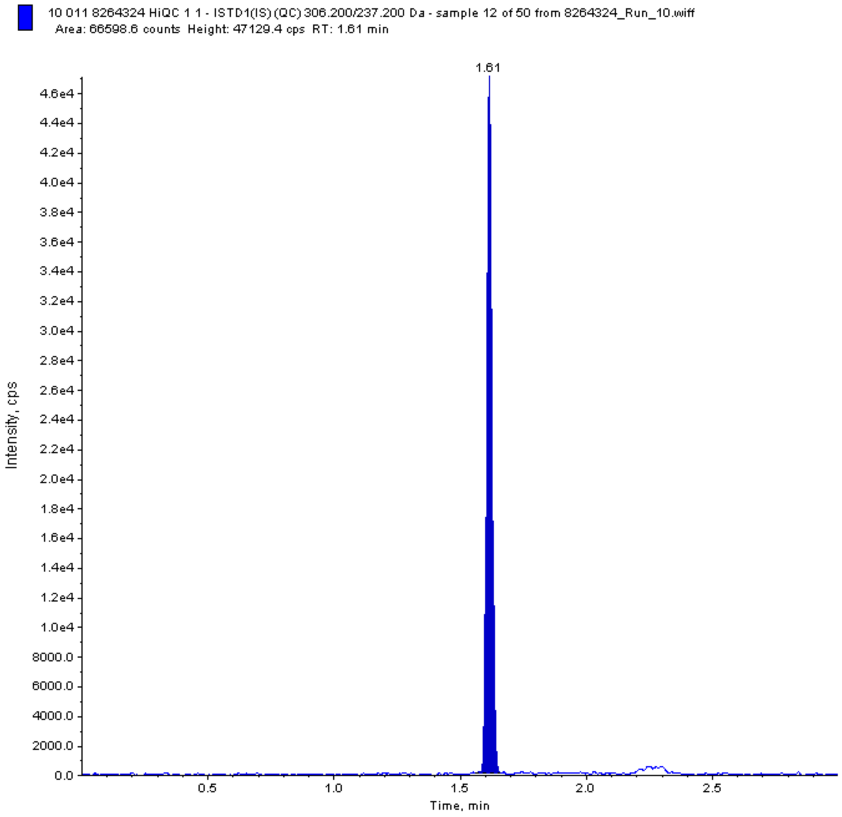

Figure 21 **Plasma Sample Collected From Subject RAD010002, Monotherapy Day 7, 3 Hour Sample Chromatogram (Run 7)**

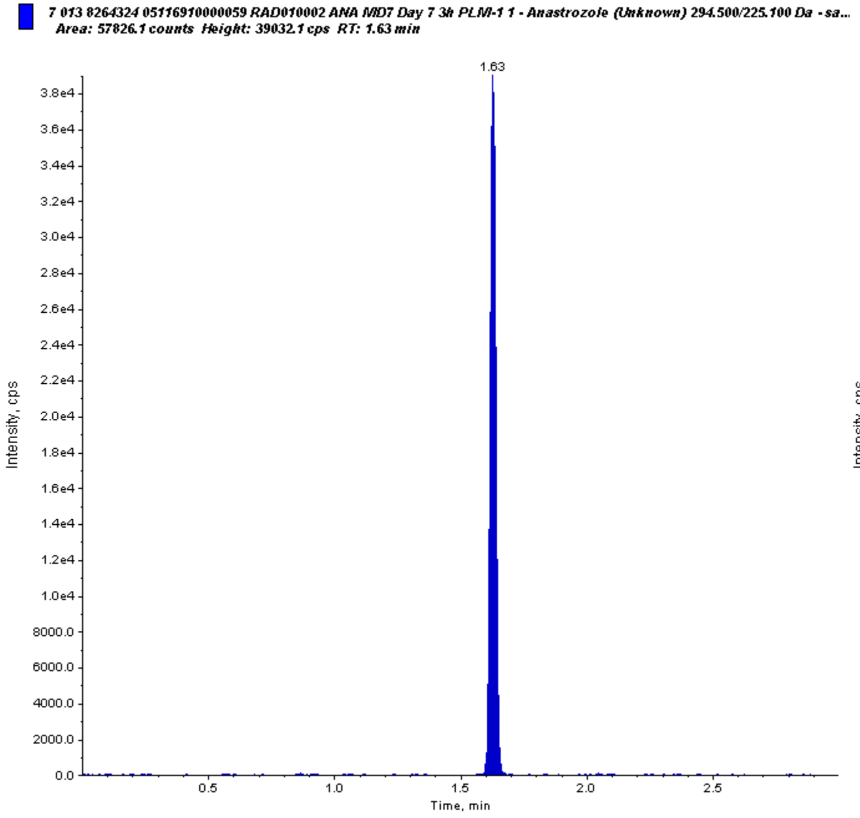

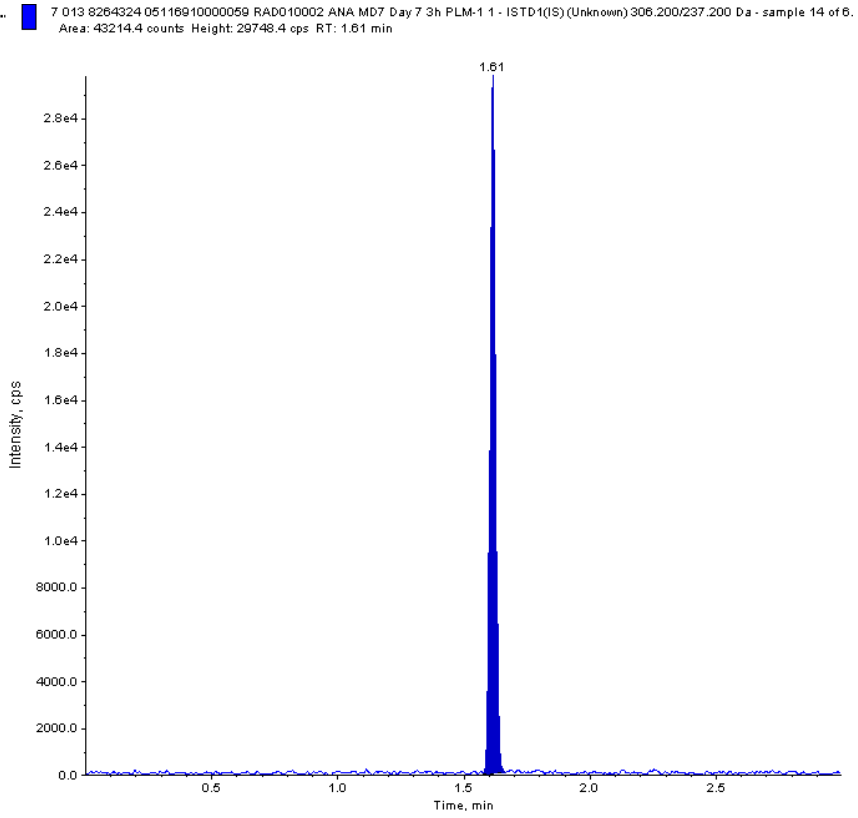

Figure 22 Plasma Sample Collected From Subject RAD010002, Monotherapy Day 7, 8 Hour Sample Chromatogram (Run 7)

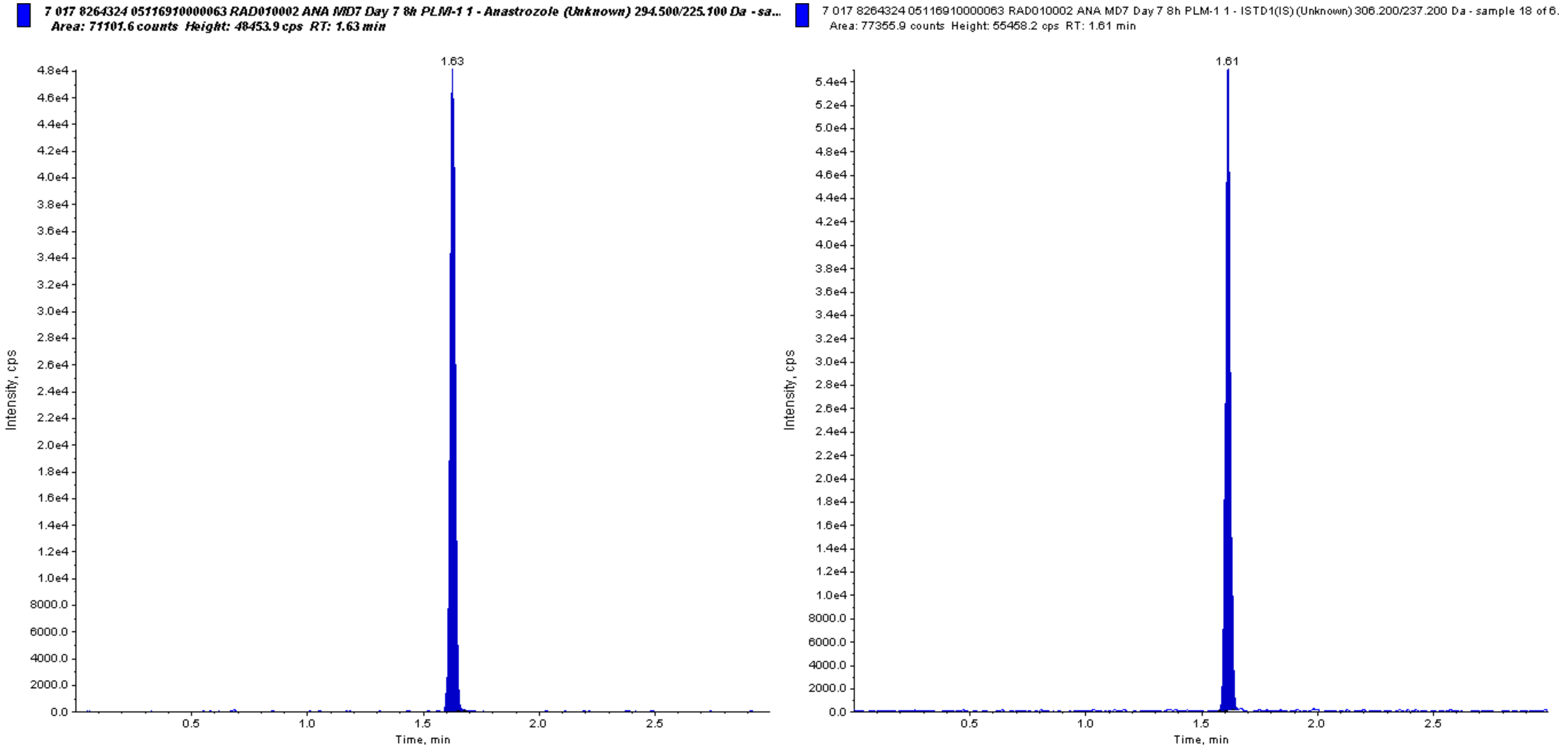

Figure 23 Plasma Sample Collected From Subject RAD010002, Cycle 1 Day 7, 2 Hour Sample Chromatogram (Run 7)

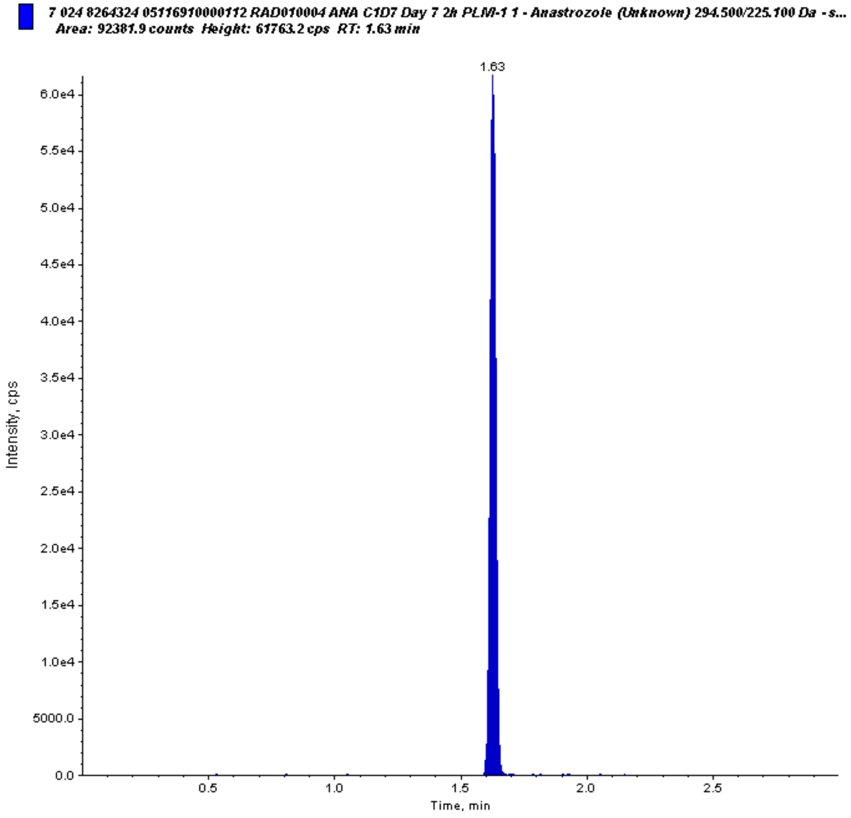

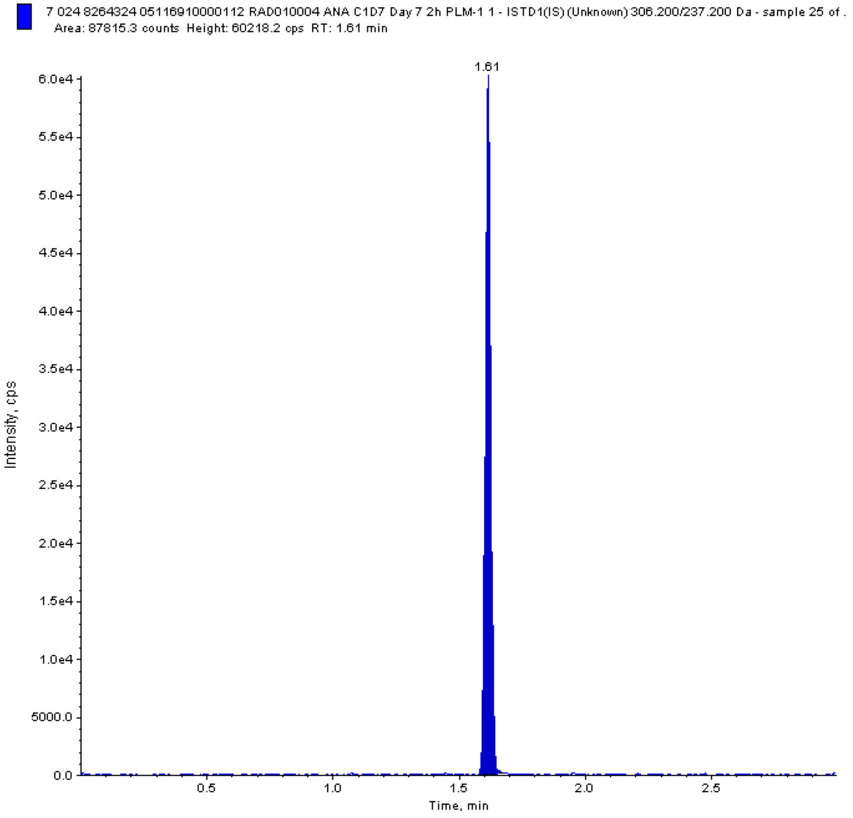

Figure 24 Plasma Sample Collected From Subject RAD010002, Cycle 1 Day 7, 5 Hour Sample Chromatogram (Run 7)

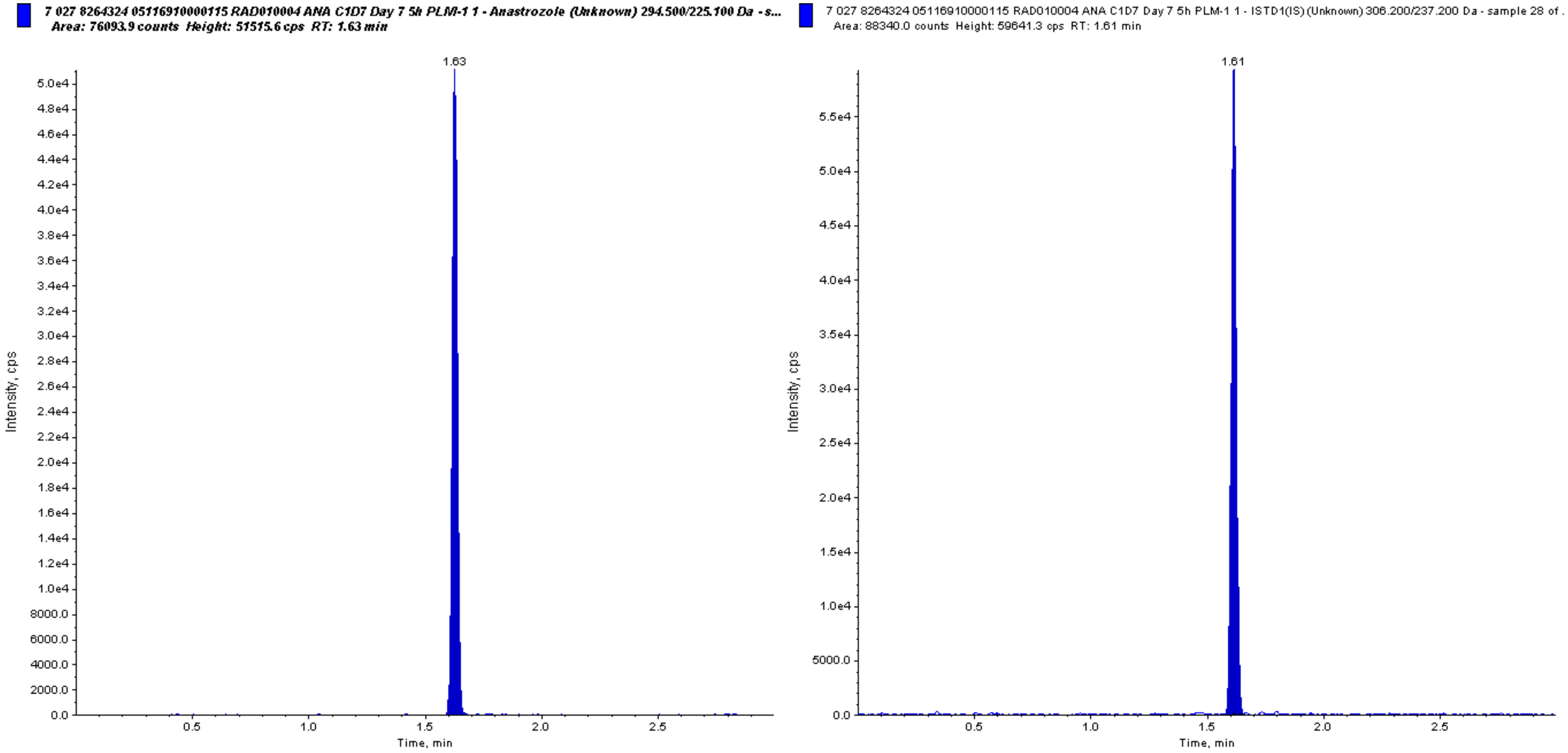

Figure 25 Plasma Sample Collected From Subject RAD010005, Monotherapy Day 7, 0.5 Hour Sample Chromatogram (Run 7)

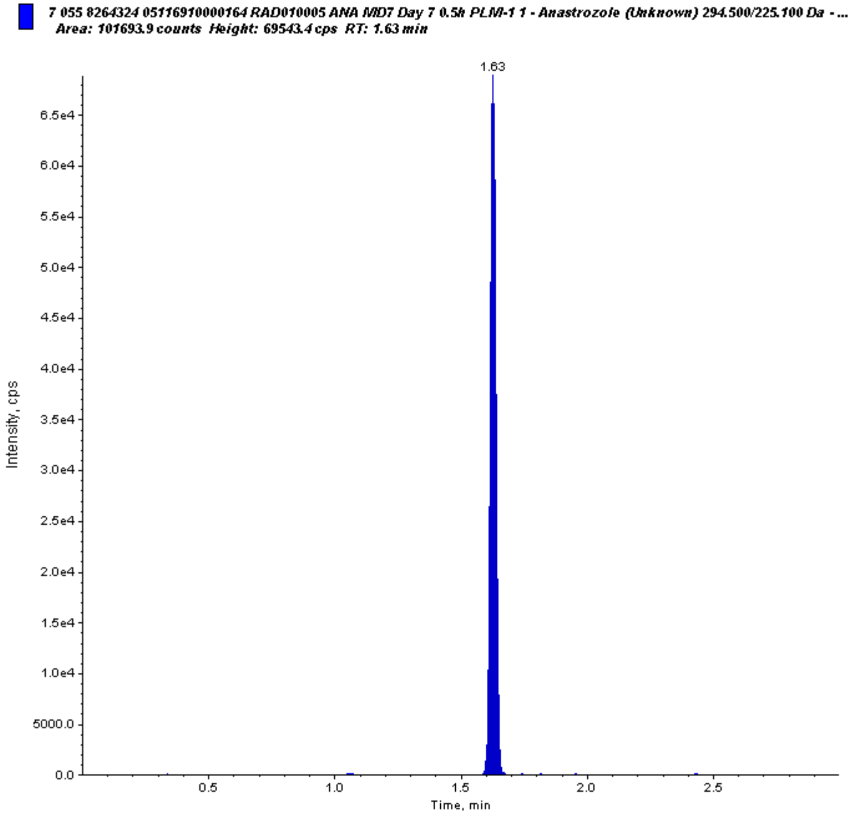

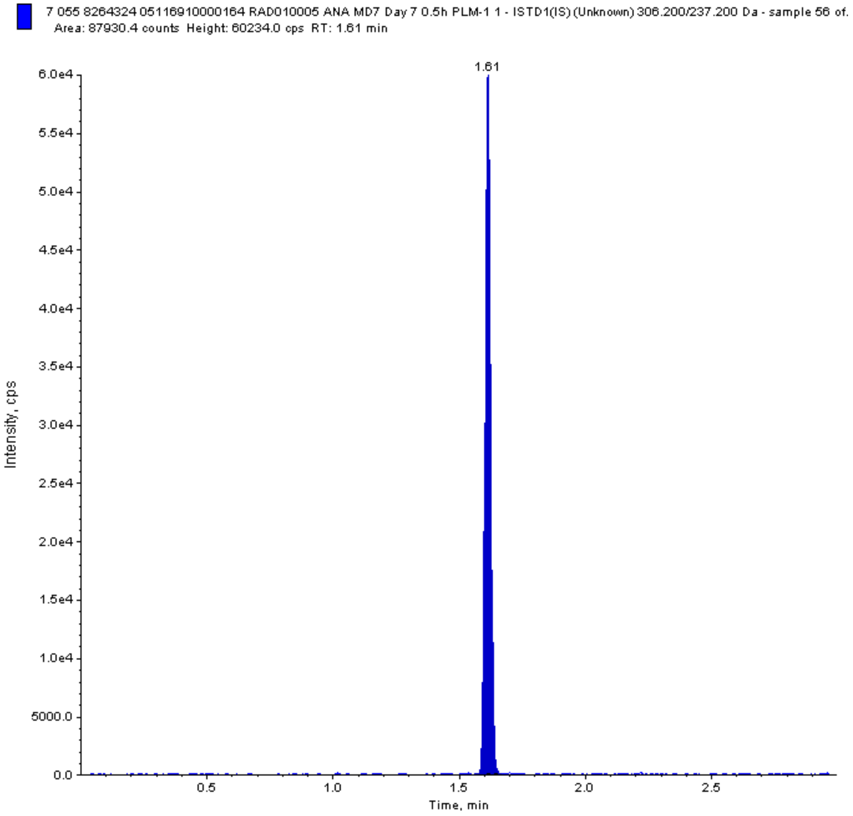

Figure 26 Plasma Sample Collected From Subject RAD010005, Monotherapy Day 7, 4 Hour Sample Chromatogram (Run 7)

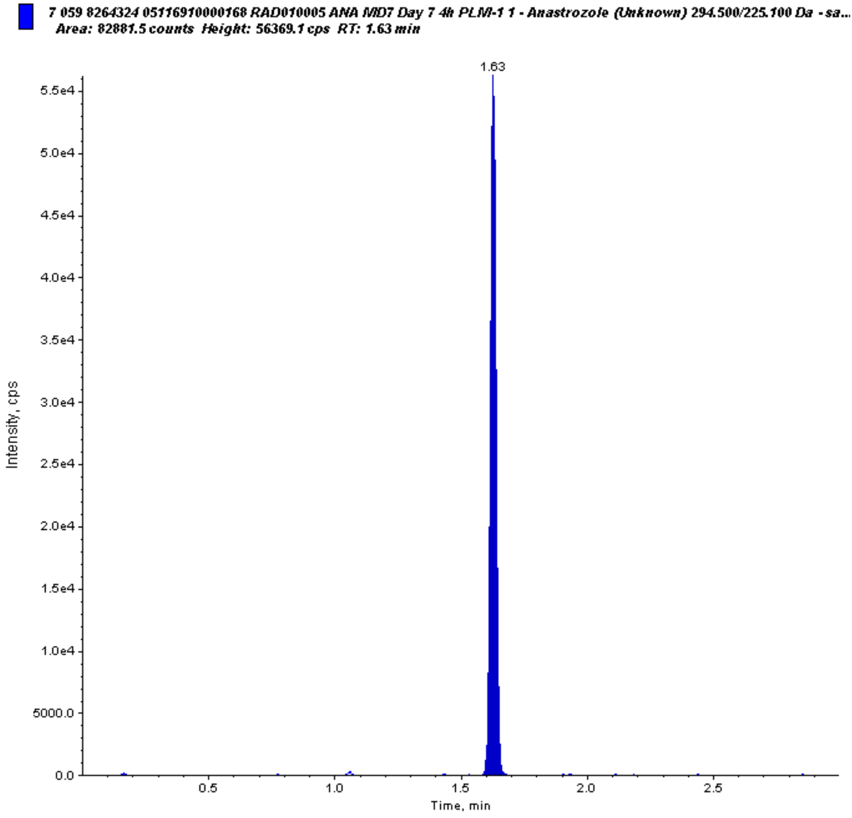

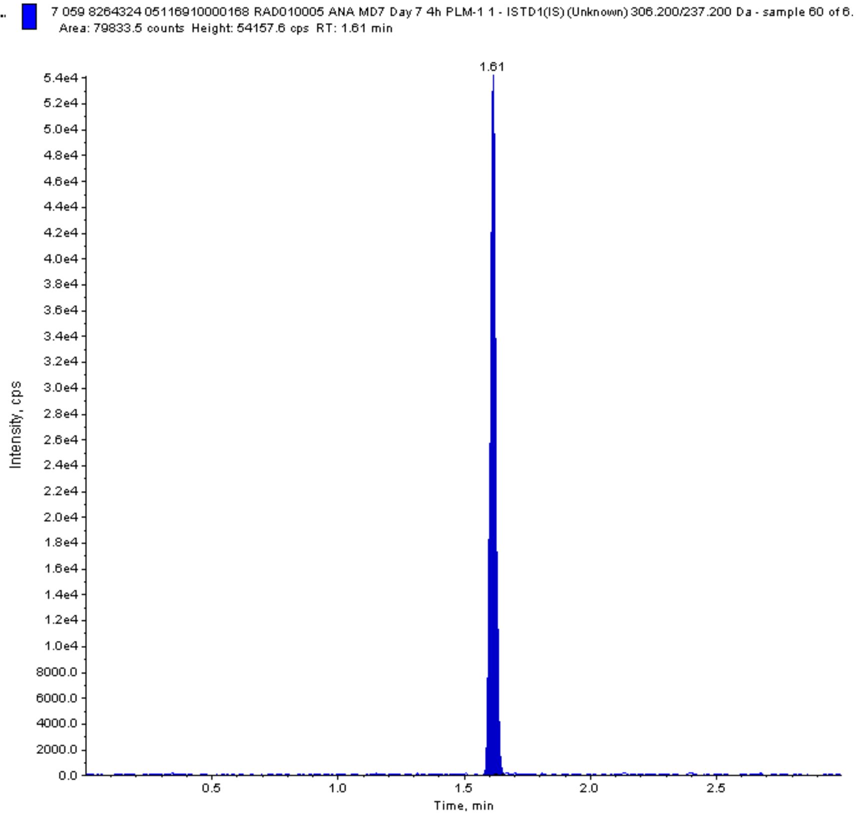

Figure 27 Plasma Sample Collected From Subject RAD010005, Cycle 1 Day 7, 0 Hour Sample Chromatogram (Run 7)

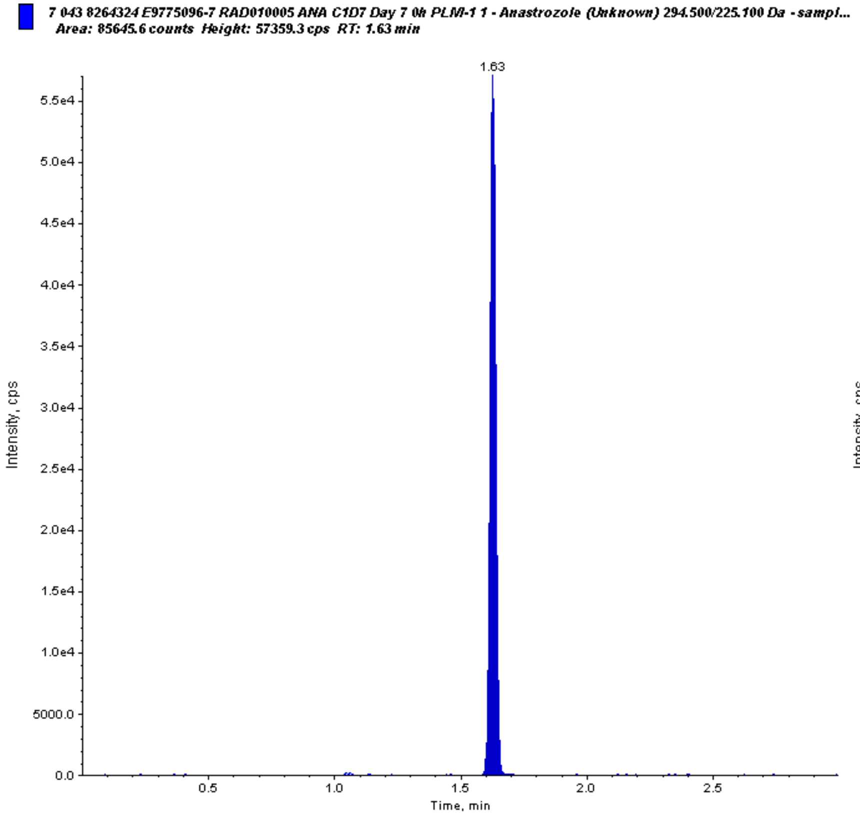

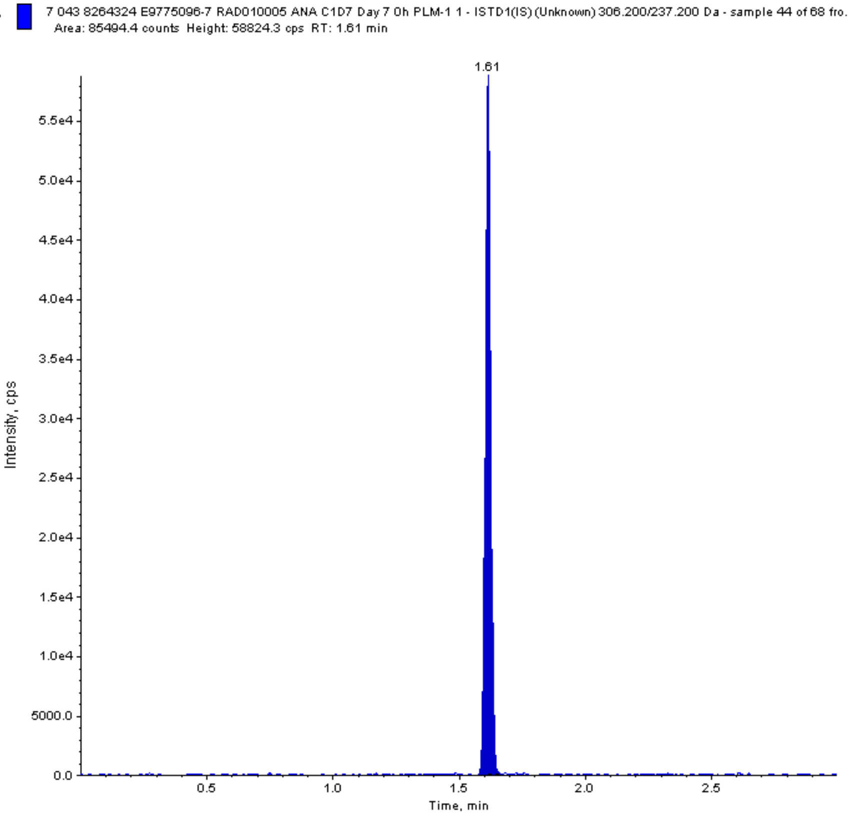

Figure 28 **Plasma Sample Collected From Subject RAD010005, Cycle 1 Day 7, 6 Hour Sample Chromatogram (Run 7)**

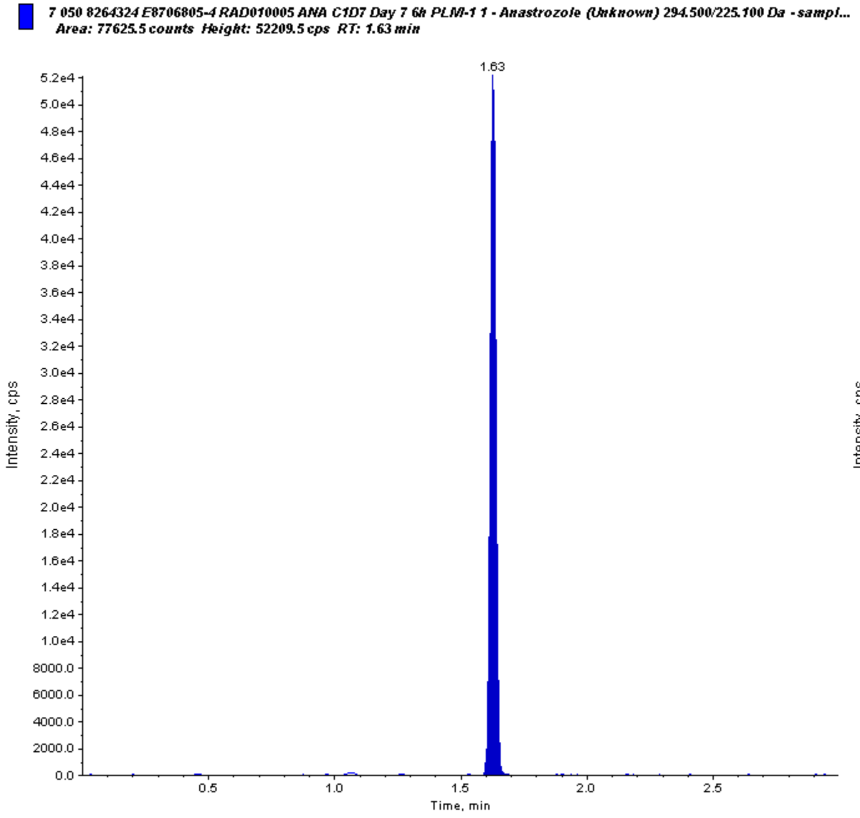

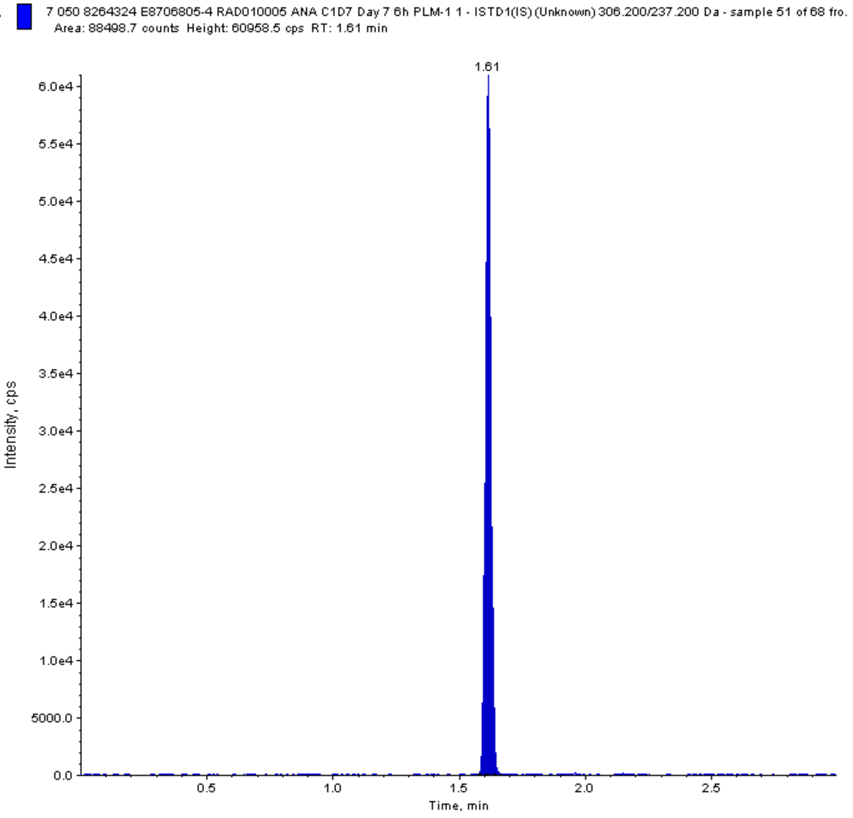

12. CERTIFICATES OF ANALYSIS

CERTIFICATE OF ANALYSIS

Toronto Research Chemicals Inc.
2 Brisbane Road, North York, ON. M3J 2J8 Canada Tel: (416) 665-9696 Fax: (416) 665-4439
E-mail: orders@trc-canada.com Website: www.trc-canada.com

1. Identification

CAS Number:
112809-51-5

Catalogue Number:
L330100

Product:
Letrozole

Synonyms:
4,4'-(1H-1,2,4-Triazol-1-ylmethylene)bisbenzonitrile; 4-[1-(4-Cyanophenyl)-1-(1,2,4-triazol-1-yl)methyl]benzonitrile; CGS 20267; Femara; Lerozole; Letrazole;

Structure:

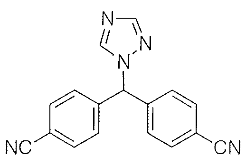

Molecular Formula:
$C_{17}H_{11}N_5$

Molecular Weight:
285.30

Source of Product:

2. Analytical Information

Lot Number:
2-EOD-12-1

Melting Point:
183-185°C

Boiling Point:
N/A

Atmosphere:
Air

Appearance of Product:
White Solid

Solubility
Chloroform, DMSO

Method for Determining Identity:
^1H NMR (DMSO-d_6) Spectroscopic and Mass Spectrometric Analysis

Stability
Not determined

Purity:
98%

Long Term Storage Condition:
-20°C Freezer

Additional Information:
TLC Conditions: SiO_2; Dichloromethane : Methanol = 9 : 1; Visualized with UV and $KMnO_4$; Single spot, Rf=0.5.
^1H NMR and Mass spectra conform to structure.

Philip Chan, Head of Quality Assurance

QC Test Date
April 12, 2012

Retest Date
April 12, 2015

COVANCE.
THE DEVELOPMENT SERVICES COMPANY

Covance
Laboratories Inc.

3301 Kinsman Blvd.
Madison, WI 53704
Tel: 608/242-2712
Fax: 608/241-7227

Certificate of Analysis

Vial-M 22002

**Compound Name:**	Letrozole-d_4		
**Other Name:**			
**CAS Number:**			
**Molecular Formula:**	$C_{17}H_7D_4N_5$	**Molecular Weight:**	289.33
**Lot Number:**	CR4-44	**Date manufactured:**	01/27/06
**Structure:**			

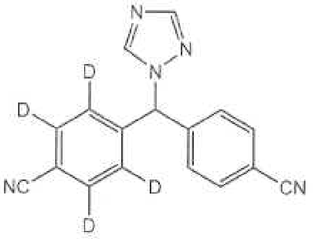

Note: 2 vials (vial 1 of 2 contains 140mg and vial 2 of 2 contains 115mg) received at ambient temperature from H. Junga (in-house synthesis) on 9 Feb. 2006. Initial storage location at BioA-Madison is -20 Freezer, rack 86A
[illegible] 9 Feb'06

Tests performed:

Test	Reference	Result
**Nuclear Magnetic Resonance:**	N/A	Conforms
**Mass Spectrometry:**	N/A	Conforms
**HPLC:**	N/A	100%
**Melting point:**	N/A	182-183°C
**Appearance:**	N/A	White solid
**Chemical Purity:**	100% by HPLC/UV	
**Storage Conditions:**	Freezer, protected from moisture and light.	
**Review/Expiration Date:**	02/08/07	

Comments:

Reviewed by, date: Heiko Junga 2/8/06
Heiko Junga

Approved by, date: [illegible] 2/8/06
Xiangyu Jiang

CERTIFICATE OF ANALYSIS

Toronto Research Chemicals Inc.
2 Brisbane Road, North York, ON. M3J 2J8 Canada Tel: (416) 665-9696 Fax: (416) 665-4439
E-mail: orders@trc-canada.com Website: www.trc-canada.com

1. Identification

CAS Number:
120511-73-1

Catalogue Number:
A637425

Product:
Anastrozole

Synonyms:
α1,α1,α3,α3-Tetramethyl-5-(1H-1,2,4-triazol-1-ylmethyl)-1,3-benzenediacetonitrile; 2-[3-(2-Cyano-2-propyl)-5-(1,2,4-triazol-1-ylmethyl)phenyl]-2-methylpropiononitrile; Anastrazole; Anastrol; Arimidex; ICI-D 1033; ZD 1033;

Structure:

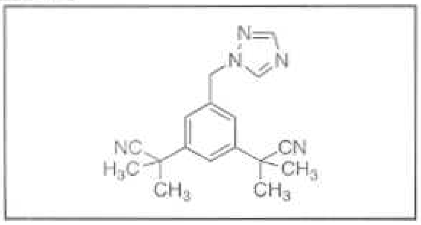

Molecular Formula:
$C_{17}H_{19}N_5$

Molecular Weight:
293.37

Source of Product:

2. Analytical Information

Lot Number:
5-XJZ-121-1

Melting Point:
83-85°C

Boiling Point:
N/A

Atmosphere:
Air

Appearance of Product:
White Solid

Solubility
Chloroform, Methanol

Method for Determining Identity:
^1H NMR ($CDCl_3$) Spectroscopic and Mass Spectrometric Analysis

Stability
Not determined

Purity:
98%

Long Term Storage Condition:
-20°C Freezer

Additional Information:
TLC Conditions: SiO_2; Dichloromethane : Methanol = 9 : 1; Visualized with UV and $KMnO_4$; Single spot, Rf=0.55.
^1H NMR and Mass spectra conform to structure.

Philip Chan, Head of Quality Assurance

QC Test Date
September 7, 2012

Retest Date
September 7, 2015

CERTIFICATE OF ANALYSIS

Toronto Research Chemicals Inc.
2 Brisbane Road, North York, ON. M3J 2J8 Canada Tel: (416) 665-9696 Fax: (416) 665-4439
E-mail: orders@trc-canada.com Website: www.trc-canada.com

1. Identification

CAS Number:

Catalogue Number:
A637427

Product:
Anastrozole-d12

Synonyms:
α1,α1,α3,α3-(Tetramethyl-d12)-5-(1H-1,2,4-triazol-1-ylmethyl)-1,3-benzenediacetonitrile; 2-[3-(2-Cyano-2-propyl)-5-(1,2,4-triazol-1-ylmethyl)phenyl]-2-methylpropiononitrile-d12; Anastrazole-d12; Anastrol-d12; Arimidex-d12; ICI-D 1033-d12; ZD 1033-d12;

Structure:

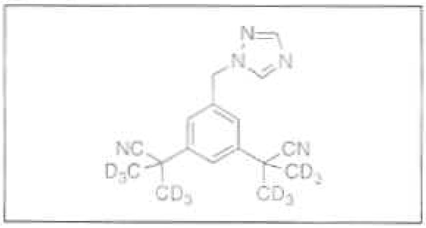

Molecular Formula:
C[illegible]H[illegible]D$_{12}$N[illegible]

Molecular Weight:
305.44

Source of Product:
Synthetic

2. Analytical Information

Lot Number:
5-GBH-105-1

Melting Point:
83-85°C

Boiling Point:
N/A

Atmosphere:
Air

Appearance of Product:
Pale Yellow Solid

Solubility
Chloroform, Ethyl Acetate

Method for Determining Identity:
^{1}H NMR ($CDCl_3$) Spectroscopic and Mass Spectrometric Analysis

Stability
Not determined

Purity:
Chemical purity: 98%
Isotopic purity: 99%

Long Term Storage Condition:
-20°C Freezer

Additional Information:
TLC Conditions: SiO_2; Dichloromethane: Methanol = 9: 1; Visualized with UV and KMnO4; Single spot, Rf=0.45.
^{1}H NMR and mass spectra conform to structure.
Normalized intensity: d12=98.20%, d11=0.42%, d10=0.18%, d9=0.01%, d8=0.01%, d7=0.09%, d6=0.05%, d5=0.32%, d4=0.05%, d3=0.08%, d2=0.23%, d1=0.00%, d0=0.035%,

Philip Chan, Head of Quality Assurance

QC Test Date
October 7, 2011

Retest Date
October 7, 2014

GEL Version ID:

TEST ITEM (COMPOUND) CHARACTERISATION SUMMARY

THIS IS A PRINTED COPY OF AN ELECTRONIC DOCUMENT. PLEASE CHECK ITS VALIDITY BEFORE USE.

**Compound name/Code:**	AZD4547	**Batch no:**	C510/2
**Alternative Ref. No:**	10-005551AZ / ADM 82589F10		
**Site of manufacture:**	Pharmaceutical Development, AstraZeneca, Macclesfield. SK10 2NA		
**Date of manufacture:**	04 May 2010		
**Site of characterisation:**	Analytical Sciences, Pharmaceutical Development, AstraZeneca, Macclesfield. SK10 2NA		
**Molecular weight:** (parent)	463.57 g/mol	**Molecular weight:** (salt or solvate)	N/A
**Purity[a]:**	99.8% w/w	**Identity confirmed:**	Yes
**Radiochem. purity:**	N/A	**Spec. radioact.:**	N/A
**Content parent[a]:**	N/A	***Note: To be used by formulators in manufacturing***	
**Impurities[a]:**	Total organic impurities: ND<br>Sulphated Ash <0.05% w/w		
**Solvents[a]:**	Water <0.05% w/w<br>Organic Solvents: ND		
**Appearance:**	A white powder		

a see calculations in PASSA Templates Guidance Document in GEL (FNC.000-070-178).

ND=none detected

**Endotoxins tested:**	No	**Microbial quality tested:**	No
**Archive sample taken:**	Yes	**Stored:**	Analytical Sciences, Pharmaceutical Development, Macclesfield
**Expiry date:**	31st May 2014		
**Storage conditions:**	Store below 30°C		
**Hazard category:** (TH Group - AZ Internal System)	TH5*		
**Reference:**	ONC.000-510-041		

GEL Version ID:

TEST ITEM (COMPOUND) CHARACTERISATION SUMMARY

**Recommended for:**	Non GLP studies	Yes
	Preclinical studies (GLP studies)	Yes
	Other specific studies:	
**Additional information:**	Document updated May 2013 - extension of shelf-life (refer to ADM: 82589F10-P6 for retest results).<br><br>Purity value was calculated by:<br><br>Purity % = (100% - (Solvents % + Water % + Inorganic Impurities % + Organic Impurities %)<br><br>At re-analysis the purity changed by <0.20%. Therefore, in accordance with LDMS_001_00007907, there is no change in assigned purity.	

THIS IS A PRINTED COPY OF AN ELECTRONIC DOCUMENT. PLEASE CHECK ITS VALIDITY BEFORE USE.

Electronic signatures are located on the last page of the pdf.

GEL Version ID: ONC.000-575-461.3.0 Approved Date Printed: 31 May 2013
Electronically Signed on 30 May 2013 19:30:39. Refer to appended signature page for details.

Appendix A Bioanalytical Results for Letrozole in Human Plasma

Table A1 Concentration of Letrozole in Human Plasma from AstraZeneca Study D2610C00011

Study ID	Subject	Treatment ID	Analyte	Day Nominal	Hour Nominal	Concentration (ng/mL)	Result Comment	Run ID
C/23/2011	RAD010001	LET C1D7	Letrozole	7	0	57	-	1
C/23/2011	RAD010001	LET C1D7	Letrozole	7	0.5	87	-	1
C/23/2011	RAD010001	LET C1D7	Letrozole	7	1	79.4	-	1
C/23/2011	RAD010001	LET C1D7	Letrozole	7	2	72.1	-	1
C/23/2011	RAD010001	LET C1D7	Letrozole	7	3	69.9	-	1
C/23/2011	RAD010001	LET C1D7	Letrozole	7	4	64.7	-	1
C/23/2011	RAD010001	LET C1D7	Letrozole	7	5	64.5	-	1
C/23/2011	RAD010001	LET C1D7	Letrozole	7	6	66.1	-	1
C/23/2011	RAD010001	LET C1D7	Letrozole	7	8	62.7	-	1
C/23/2011	RAD010001	LET MD7	Letrozole	7	0	66.8	-	1
C/23/2011	RAD010001	LET MD7	Letrozole	7	0.5	90.8	-	1
C/23/2011	RAD010001	LET MD7	Letrozole	7	1	80.3	-	1
C/23/2011	RAD010001	LET MD7	Letrozole	7	2	83.7	-	1
C/23/2011	RAD010001	LET MD7	Letrozole	7	3	80.9	-	1
C/23/2011	RAD010001	LET MD7	Letrozole	7	4	75.5	-	1
C/23/2011	RAD010001	LET MD7	Letrozole	7	5	71.3	-	1
C/23/2011	RAD010001	LET MD7	Letrozole	7	6	74	-	1
C/23/2011	RAD010001	LET MD7	Letrozole	7	8	85.9	-	1
C/23/2011	RAD010002	LET C1D7	Letrozole	7	0	< 1.00	Analysed in error	3
C/23/2011	RAD010002	LET MD7	Letrozole	7	3	< 1.00	Analysed in error	3
C/23/2011	RAD010002	LET MD7	Letrozole	7	4	< 1.00	Analysed in error	3

Study ID	Subject	Treatment ID	Analyte	Day Nominal	Hour Nominal	Concentration (ng/mL)	Result Comment	Run ID
C/23/2011	RAD010002	LET MD7	Letrozole	7	5	< 1.00	Analysed in error	3
C/23/2011	RAD010002	LET MD7	Letrozole	7	6	< 1.00	Analysed in error	3
C/23/2011	RAD010002	LET MD7	Letrozole	7	8	< 1.00	Analysed in error	3
C/23/2011	RAD010003	LET C1D7	Letrozole	7	0	48.2	-	3
C/23/2011	RAD010003	LET C1D7	Letrozole	7	0.5	53.6	-	3
C/23/2011	RAD010003	LET C1D7	Letrozole	7	1	53.9	-	3
C/23/2011	RAD010003	LET C1D7	Letrozole	7	2	53.6	-	3
C/23/2011	RAD010003	LET C1D7	Letrozole	7	3	54.4	-	3
C/23/2011	RAD010003	LET C1D7	Letrozole	7	4	55.9	-	3
C/23/2011	RAD010003	LET C1D7	Letrozole	7	5	50.2	-	3
C/23/2011	RAD010003	LET C1D7	Letrozole	7	6	50.1	-	3
C/23/2011	RAD010003	LET C1D7	Letrozole	7	8	47.4	-	3
C/23/2011	RAD010003	LET MD7	Letrozole	7	0	45.6	-	3
C/23/2011	RAD010003	LET MD7	Letrozole	7	0.5	68.9	-	3
C/23/2011	RAD010003	LET MD7	Letrozole	7	1	58.5	-	1
C/23/2011	RAD010003	LET MD7	Letrozole	7	2	55.8	-	1
C/23/2011	RAD010003	LET MD7	Letrozole	7	3	51.8	-	1
C/23/2011	RAD010003	LET MD7	Letrozole	7	4	52.4	-	1
C/23/2011	RAD010003	LET MD7	Letrozole	7	5	51.2	-	1
C/23/2011	RAD010003	LET MD7	Letrozole	7	6	48.2	-	1
C/23/2011	RAD010003	LET MD7	Letrozole	7	8	47.9	-	1
C/23/2011	RAD010004	LET C1D7	Letrozole	7	0	< 1.00	Analysed in error	3

Study ID	Subject	Treatment ID	Analyte	Day Nominal	Hour Nominal	Concentration (ng/mL)	Result Comment	Run ID
C/23/2011	RAD010004	LET C1D7	Letrozole	7	0.5	< 1.00	Analysed in error	3
C/23/2011	RAD010004	LET C1D7	Letrozole	7	1	< 1.00	Analysed in error	3
C/23/2011	RAD010004	LET C1D7	Letrozole	7	2	< 1.00	Analysed in error	3
C/23/2011	RAD010004	LET C1D7	Letrozole	7	3	< 1.00	Analysed in error	3
C/23/2011	RAD010004	LET C1D7	Letrozole	7	4	< 1.00	Analysed in error	3
C/23/2011	RAD010004	LET C1D7	Letrozole	7	5	< 1.00	Analysed in error	3
C/23/2011	RAD010004	LET C1D7	Letrozole	7	6	< 1.00	Analysed in error	3
C/23/2011	RAD010004	LET C1D7	Letrozole	7	8	< 1.00	Analysed in error	3
C/23/2011	RAD010004	LET MD7	Letrozole	7	0	< 1.00	Analysed in error	3
C/23/2011	RAD010004	LET MD7	Letrozole	7	0.5	< 1.00	Analysed in error	3
C/23/2011	RAD010004	LET MD7	Letrozole	7	1	< 1.00	Analysed in error	3
C/23/2011	RAD010004	LET MD7	Letrozole	7	2	< 1.00	Analysed in error	3
C/23/2011	RAD010004	LET MD7	Letrozole	7	3	< 1.00	Analysed in error	3
C/23/2011	RAD010004	LET MD7	Letrozole	7	4	< 1.00	Analysed in error	3
C/23/2011	RAD010004	LET MD7	Letrozole	7	5	< 1.00	Analysed in error	3
C/23/2011	RAD010004	LET MD7	Letrozole	7	6	< 1.00	Analysed in error	3
C/23/2011	RAD010004	LET MD7	Letrozole	7	8	< 1.00	Analysed in error	3
C/23/2011	RAD010005	LET C1D7	Letrozole	7	0	< 1.00	Analysed in error	3
C/23/2011	RAD010005	LET C1D7	Letrozole	7	0.5	< 1.00	Analysed in error	3
C/23/2011	RAD010005	LET C1D7	Letrozole	7	1	< 1.00	Analysed in error	3
C/23/2011	RAD010005	LET C1D7	Letrozole	7	2	< 1.00	Analysed in error	3
C/23/2011	RAD010005	LET C1D7	Letrozole	7	3	< 1.00	Analysed in error	3

Study ID	Subject	Treatment ID	Analyte	Day Nominal	Hour Nominal	Concentration (ng/mL)	Result Comment	Run ID
C/23/2011	RAD010005	LET C1D7	Letrozole	7	4	< 1.00	Analysed in error	3
C/23/2011	RAD010005	LET C1D7	Letrozole	7	5	< 1.00	Analysed in error	3
C/23/2011	RAD010005	LET C1D7	Letrozole	7	6	< 1.00	Analysed in error	3
C/23/2011	RAD010005	LET C1D7	Letrozole	7	8	< 1.00	Analysed in error	3
C/23/2011	RAD010005	LET MD7	Letrozole	7	0	< 1.00	Analysed in error	3
C/23/2011	RAD010005	LET MD7	Letrozole	7	0.5	< 1.00	Analysed in error	3
C/23/2011	RAD010005	LET MD7	Letrozole	7	1	< 1.00	Analysed in error	3
C/23/2011	RAD010005	LET MD7	Letrozole	7	2	< 1.00	Analysed in error	3
C/23/2011	RAD010005	LET MD7	Letrozole	7	3	< 1.00	Analysed in error	3
C/23/2011	RAD010005	LET MD7	Letrozole	7	4	< 1.00	Analysed in error	3
C/23/2011	RAD010005	LET MD7	Letrozole	7	5	< 1.00	Analysed in error	3
C/23/2011	RAD010005	LET MD7	Letrozole	7	6	< 1.00	Analysed in error	3
C/23/2011	RAD010005	LET MD7	Letrozole	7	8	< 1.00	Analysed in error	3
C/23/2011	RAD040001	LET C1D7	Letrozole	7	0	221	-	4
C/23/2011	RAD040001	LET C1D7	Letrozole	7	0.5	220	-	4
C/23/2011	RAD040001	LET C1D7	Letrozole	7	1	232	-	4
C/23/2011	RAD040001	LET C1D7	Letrozole	7	2	224	-	4
C/23/2011	RAD040001	LET C1D7	Letrozole	7	3	240	-	4
C/23/2011	RAD040001	LET C1D7	Letrozole	7	4	239	-	4
C/23/2011	RAD040001	LET C1D7	Letrozole	7	5	223	-	4
C/23/2011	RAD040001	LET C1D7	Letrozole	7	6	224	-	4
C/23/2011	RAD040001	LET C1D7	Letrozole	7	8	212	-	4

Study ID	Subject	Treatment ID	Analyte	Day Nominal	Hour Nominal	Concentration (ng/mL)	Result Comment	Run ID
C/23/2011	RAD040001	LET MD7	Letrozole	7	0	202	-	4
C/23/2011	RAD040001	LET MD7	Letrozole	7	0.5	252	-	4
C/23/2011	RAD040001	LET MD7	Letrozole	7	1	255	-	4
C/23/2011	RAD040001	LET MD7	Letrozole	7	2	236	-	4
C/23/2011	RAD040001	LET MD7	Letrozole	7	3	224	-	4
C/23/2011	RAD040001	LET MD7	Letrozole	7	4	153	-	4
C/23/2011	RAD040001	LET MD7	Letrozole	7	5	209	-	4
C/23/2011	RAD040001	LET MD7	Letrozole	7	6	212	-	4
C/23/2011	RAD040001	LET MD7	Letrozole	7	8	214	-	4

Appendix B Bioanalytical Results for Anastrozole in Human Plasma

Table B1 Concentration of Anastrozole in Human Plasma from AstraZeneca Study D2610C00011

Study ID	Subject	Treatment ID	Analyte	Day Nominal	Hour Nominal	Concentration (ng/mL)	Result Comment	Run ID
C/23/2011	RAD010002	ANA C1D7	Anastrozole	7	0	32.2	-	7
C/23/2011	RAD010002	ANA MD7	Anastrozole	7	3	53.1	-	7
C/23/2011	RAD010002	ANA MD7	Anastrozole	7	4	43.4	-	7
C/23/2011	RAD010002	ANA MD7	Anastrozole	7	5	38.1	-	7
C/23/2011	RAD010002	ANA MD7	Anastrozole	7	6	35	-	7
C/23/2011	RAD010002	ANA MD7	Anastrozole	7	8	36.4	-	7
C/23/2011	RAD010004	ANA C1D7	Anastrozole	7	0	28.6	-	7
C/23/2011	RAD010004	ANA C1D7	Anastrozole	7	0.5	34.4	-	7
C/23/2011	RAD010004	ANA C1D7	Anastrozole	7	1	36.3	-	7
C/23/2011	RAD010004	ANA C1D7	Anastrozole	7	2	41.7	-	7
C/23/2011	RAD010004	ANA C1D7	Anastrozole	7	3	39.4	-	7
C/23/2011	RAD010004	ANA C1D7	Anastrozole	7	4	34.9	-	7
C/23/2011	RAD010004	ANA C1D7	Anastrozole	7	5	34.1	-	7
C/23/2011	RAD010004	ANA C1D7	Anastrozole	7	6	35.1	-	7
C/23/2011	RAD010004	ANA C1D7	Anastrozole	7	8	35.6	-	7
C/23/2011	RAD010004	ANA MD7	Anastrozole	7	0	27.7	-	7
C/23/2011	RAD010004	ANA MD7	Anastrozole	7	0.5	33	-	7
C/23/2011	RAD010004	ANA MD7	Anastrozole	7	1	42.1	-	7
C/23/2011	RAD010004	ANA MD7	Anastrozole	7	2	38.5	-	7
C/23/2011	RAD010004	ANA MD7	Anastrozole	7	3	39.4	-	7
C/23/2011	RAD010004	ANA MD7	Anastrozole	7	4	34.4	-	7

Study ID	Subject	Treatment ID	Analyte	Day Nominal	Hour Nominal	Concentration (ng/mL)	Result Comment	Run ID
C/23/2011	RAD010004	ANA MD7	Anastrozole	7	5	35.3	-	7
C/23/2011	RAD010004	ANA MD7	Anastrozole	7	6	36.4	-	7
C/23/2011	RAD010004	ANA MD7	Anastrozole	7	8	34.2	-	7
C/23/2011	RAD010005	ANA C1D7	Anastrozole	7	0	39.7	-	7
C/23/2011	RAD010005	ANA C1D7	Anastrozole	7	0.5	35.8	-	7
C/23/2011	RAD010005	ANA C1D7	Anastrozole	7	1	45.6	-	7
C/23/2011	RAD010005	ANA C1D7	Anastrozole	7	2	41.5	-	7
C/23/2011	RAD010005	ANA C1D7	Anastrozole	7	3	42.5	-	7
C/23/2011	RAD010005	ANA C1D7	Anastrozole	7	4	39.1	-	7
C/23/2011	RAD010005	ANA C1D7	Anastrozole	7	5	36.6	-	7
C/23/2011	RAD010005	ANA C1D7	Anastrozole	7	6	34.7	-	7
C/23/2011	RAD010005	ANA C1D7	Anastrozole	7	8	38.2	-	7
C/23/2011	RAD010005	ANA MD7	Anastrozole	7	0	35.6	-	7
C/23/2011	RAD010005	ANA MD7	Anastrozole	7	0.5	45.9	-	7
C/23/2011	RAD010005	ANA MD7	Anastrozole	7	1	46.3	-	7
C/23/2011	RAD010005	ANA MD7	Anastrozole	7	2	35.6	-	7
C/23/2011	RAD010005	ANA MD7	Anastrozole	7	3	39.8	-	7
C/23/2011	RAD010005	ANA MD7	Anastrozole	7	4	41.1	-	7
C/23/2011	RAD010005	ANA MD7	Anastrozole	7	5	38.1	-	7
C/23/2011	RAD010005	ANA MD7	Anastrozole	7	6	38.7	-	7
C/23/2011	RAD010005	ANA MD7	Anastrozole	7	8	36.1	-	7

Supplementary Note 2

CLINICAL STUDY PROTOCOL

ICTU Adopted

**Study Title:**	A single arm phase IIa study (with combination safety run-in) to assess the safety and efficacy of AZD4547 in combination with either anastrozole or letrozole in ER positive breast cancer patients who have progressed on treatment with anastrozole or letrozole - **RADICAL**
**Protocol Number:**	C/23/2011
**Product:**	AZD4547 (FGFR inhibitor)
**Sponsor:**	Imperial College London
**EudraCT Number:**	2011-000454-32
**ISRCTN**:	80307982
**ClinicalTrials.gov ID:**	NCT01791985
**CRUK Ref:**	CRUKD/11/004
**AstraZeneca Ref:**	D2610C00011
**Development Phase:**	Ib/IIa
**Protocol Date:**	v8.0, 28 July 2016

Protocol amendment history: v7.0, 06 October 2015
v6.0, 13 April 2015
v5.0, 03 February 2015
v4.0, 12 December 2013
v3.0, 25 February 2013
v2.0, 20 February 2012
v1.0, 10 October 2011

Property of: Imperial Clinical Trials Unit-Section on Cancer (ICTU-Cancer)
May not be used, divulged or published without the consent of: ICTU-Cancer

CONTACT LIST

Chief Investigator

Professor Michael J Seckl
Imperial College London
1st Floor, Department of Medical Oncology
Charing Cross Hospital
Fulham Palace Road
London W6 8RF

Email: m.seckl@imperial.ac.uk
Tel: +44 (0)20 3311 1421
Fax: +44 (0)20 3313 5577

Sponsor

Imperial College London
Joint Research Compliance Office
Room 510C, 5th Floor, Lab Block
Charing Cross Hospital
Fulham Palace Road
London W6 8RF

Contact: Gary Roper
Head of Regulatory Compliance
Email: gary.roper@imperial.ac.uk
Tel: +44 (0)20 3311 0204
Fax: +44 (0)20 3311 0203

Funders

Cancer Research UK	AstraZeneca UK
Angel Building	Parklands
407 St John Street	Alderley Park
London	Macclesfield
EC1V 4AD	Cheshire
	SK10 4TG

ICTU-Cancer Study Coordinator

Mr Philip Badman
Cancer Research UK Imperial Centre: Clinical Trials Section
Imperial College London
Department of Surgery and Cancer
3rd Floor Radiotherapy Building
Hammersmith Hospital Campus
Du Cane Road
London, W12 0NN

Email: radical@imperial.ac.uk
Tel: +44 (0)20 3311 5203
Fax: +44 (0)20 3311 7443

Study Statistician

Dr Xinxue Liu
Statistician
Imperial Clinical Trials Unit-Cancer
Imperial College London
Email: xinxue.liu@imperial.ac.uk
Tel: +44 (0)207 594 1739

Protocol Development Group

Professor R.C. Coombes
Consultant Medical Oncologist
Imperial College Healthcare NHS Trust/
Imperial College London
Email: c.coombes@imperial.ac.uk
Tel: +44(0)208 383 5831

Professor E.R. Plummer
Professor of Experimental Cancer Medicine
Northern Institute for Cancer Research
Newcastle University
Email: ruth.plummer@ncl.ac.uk
Tel: +44(0)191 208 4300

Hanna Nicholas
CRUK Centre Senior Clinical Trials Manager
Cancer Research UK Imperial Centre: Clinical Trials Section
Department of Surgery and Cancer
Imperial College London
Email: h.nicholas@imperial.ac.uk
Tel: +44 (0)20 7594 2165

PK Laboratory (AZD4547)

PRA International
Early Development Services Bioanalytical Laboratory
Westerbrink 3
9405 BJ Assen
The Netherlands

Tel: +31(50) 402-2222
Fax: +31 (50) 402-2223

PK Laboratory (Anastrozole and Letrozole)

Covance Laboratories Inc.
3301 Kinsman Boulevard
Madison, Wisconsin 53704, USA

Brian Dayton
CBioA Program Manager,
Email: Brian.Dayton@covance.com
Tel: 001 608 245-7009

PD Laboratory

Quintiles Limited,
Global Central Laboratories
The Alba Campus
Rosebank, Livingston
West Lothian EH54 7EG
United Kingdom

Karen BrownProject Manager

Email: Karen.Brown@quintiles.com

Tel: +44 (0) 1506 814 501
Fax: + 44 (0)1506 816091

IMP Manufacturing Facilities

AstraZeneca R&D
Pharmaceutical Development
R&D Supply Chain
Silkroad Business Park
Macclesfield
Cheshire SK10 2NA
United Kingdom

AstraZeneca R&D Mölndal
Pepparedsleden 1
Mölndal
Sweden, SE-431 83
Juliette Brady
Supply Chain Programme Manager
Tel: +44(0)1625 513184
Fax: +44(0)1625 517468

IMP Distributor

Fisher Clinical Services
Langhurstwood Road
Horsham RH12 4QD
United Kingdom

Kerry Tingley Supply Chain Manager
Email: Kerry.Tingley@thermofisher.com
Tel: +44(0)1403 212623
Fax: +44(0)1403 212712

Central Radiological Review

Imperial College NHS Healthcare Trust /
Imperial College London

Dr Adrian Lim
Consultant Radiologist
Email: a.lim@imperial.ac.uk
Tel: +44(0)203311 1858
Fax: +44(0)203311 1861

TABLE OF CONTENTS

CONTACT LIST 2
TABLE OF CONTENTS 6
TRIAL SUMMARY 13
1. BACKGROUND AND RATIONALE 17
1.1. Breast Cancer 17
1.2. Investigational Agent 17
1.3. Non-clinical Studies 18
1.3.1. In Vitro Studies ... 18
1.3.2. In Vivo Studies ... 18
1.4. Clinical Studies 18
1.5. Aromatase Inhibitors 22
1.5.1. Pharmacokinetic Considerations of the Combination Therapy ... 22
1.6. Rationale 22
1.6.1. Rationale for use in Breast Cancer ... 23
1.6.2. Rationale for Revised Phase IIa Study Design ... 23
1.6.3. Rationale for AZD4547 Dose and Schedule (Phase IIa) ... 23
1.6.4. Risk – Benefit Assessment ... 24
2. STUDY OBJECTIVES AND ENDPOINTS 29
2.1. Primary Objectives 29
2.2. Secondary Objectives 29
2.3. Primary Endpoints 29
2.4. Secondary Endpoints 29
2.5. Exploratory Objectives / Endpoints 30
3. STUDY DESIGN 31
3.1. Overall Study Design 31
3.2. Treatment Regimens 31
3.3. Study Flow Charts 32
3.4. Follow-up 33
3.5. Study Termination 33
3.5.1. Treatment after Study Termination ... 33
4. PATIENT SELECTION AND RECRUITMENT 34
4.1. Screening and Enrolment 34
4.2. Subject Selection 34
4.2.1. Inclusion Criteria ... 34
4.2.2. Exclusion Criteria ... 36
5. STUDY PLAN AND PROCEDURES 38
5.1. Study Schedules 38
5.2. Procedures and Measurements 43
5.2.1. Demographic Data ... 43
5.2.2. Medical History / Concomitant Medical Conditions ... 43
5.2.3. Previous and Current Radiotherapy ... 43
5.2.4. Previous and Current Chemotherapy ... 43
5.2.5. Endocrine Therapy ... 43
5.2.6. Targeted Therapy ... 43
5.2.7. Surgical History ... 43
5.2.8. Medical History of Breast Cancer ... 43
5.2.9. Characteristics of the Primary or Metastatic Breast Tumour ... 43
5.2.10. Concomitant Medications ... 44
5.2.11. Prohibited Study Medication ... 44
5.2.12. Physical Examination ... 44
5.2.13. ECOG Performance Status ... 44
5.2.14. Vital Signs ... 44
5.2.15. ECG ... 44
5.2.16. Echocardiogram and/or MUGA Scan ... 45
5.2.17. Laboratory Evaluations ... 45
5.2.18. Ophthalmic Assessment ... 45

5.2.19. Treatment Compliance (Patient Diary) ... *46*
5.2.20. Principle Investigator Disease Status Assessments (Safety Run-In only) ... *46*
5.2.21. Tumour Assessments (Phase IIa only) ... *46*
5.2.22. Assessment of Archival Tissue Oncology Biomarkers (Phase IIa only) ... *47*
5.3. Pharmacokinetics (Safety run-in only) 47
5.3.1. Collection of Pharmacokinetic Samples ... *47*
5.3.2. Determination of drug concentration in PK samples ... *48*
5.3.3. PK Parameter Derivation ... *48*
5.4. Pharmacodynamics 49
5.4.1. Saftey Run-In ... *49*
5.4.2. PIIa ... *49*
5.5. Exploratory Research 49
5.5.1. Biomarker research ... *49*
5.5.2. Circulating Tumour Specific DNA (Phase IIa only) ... *50*
5.5.3. Pharmacogenetics ... *50*
5.6. Chain of Custody of Biological Samples 50
5.7. Total Blood Volumes 50
6. STUDY TREATMENT 53
6.1. Non Investigational Medicinal Products 53
6.2. Investigational Medicinal Product 53
6.2.1. Supply, Packaging and Labelling ... *53*
6.2.2. Storage and Dispensing ... *53*
6.2.3. Dosage and Duration ... *53*
6.2.4. Definition of Dose-Limiting Toxicity for assessment of safety and tolerability of AZD4547 in the safety run-in study ... *54*
6.2.5. Definition of Severe Toxicity for Assessment of Safety and Tolerability of AZD4547 in the PIIa study ... *55*
6.2.6. Definition of Evaluable Patient ... *55*
6.2.7. Safety Review Committee ... *55*
6.2.8. Dose Modifications for AZD4547 ... *55*
6.2.9. Accountability ... *60*
6.2.10. Compliance ... *60*
6.2.11. Drug Interactions/Precautions ... *60*
6.2.12. Overdose of IMP ... *61*
6.3.1. Permanent Discontinuation of Study Medication ... *61*
6.3.2. Withdrawal from Study ... *61*
6.3.3. Procedures for Withdrawal from Study ... *62*
7. PHARMACOVIGILANCE 63
7.1. Definition of an Adverse Event (AE) 63
7.1.1. Disease Progression ... *63*
7.1.2. New Cancers ... *63*
7.2. Recording of Adverse Events 63
7.3. Severity of Adverse Events 64
7.4. Causality of Adverse Events 64
7.5. Abnormal Laboratory Test Results 64
7.6. Definitions of Serious Adverse Events (SAE) 64
7.7. Reporting of SAEs 65
7.8. Definition of a Serious Adverse Reaction (SAR) 65
7.9. Definition of Suspected Unexpected Serious Adverse Reaction (SUSAR) 65
7.10. Reporting of SUSARs 65
7.11. Annual Reporting of Serious Adverse Reactions 65
8. STATISTICAL ANALYSES 66
8.1. Sample Size and Power Considerations 66
8.2. Data Analysis 66
8.2.1. Interim Analysis ... *66*
8.2.2. Preliminary final analysis ... *66*
8.2.3. Final Analysis ... *67*

8.2.4. Missing, Unused and Spurious Data ... 67
8.2.5. Deviations from the Statistical Plan ... 67
8.2.6. Efficacy Analysis ... 67
8.2.7. Primary Analysis ... 67
8.2.8. Secondary Analyses ... 67
8.2.9. Safety Analysis ... 68
9. REGULATORY, ETHICAL AND LEGAL ISSUES 69
9.1. Declaration of Helsinki 69
9.2. Good Clinical Practice 69
9.3. Independent Ethics Committee/Institutional Review Board Approval 69
9.3.1. Initial Approval ... 69
9.3.2. Approval of Amendments ... 69
9.3.3. Annual Safety Reports and End of Trial Notification ... 69
9.4. Regulatory Authority Approval 69
9.5. Insurance 69
9.6. Informed Consent 69
9.7. Contact with General Practitioner 70
9.8. Subject Confidentiality 70
9.9. Data Protection 70
9.10. End of Trial 70
9.11. Study Documentation and Data Storage 70
10. DATA AND STUDY MANAGEMENT 71
10.1. Source Data 71
10.2. Language 71
10.3. Data Collection 71
10.4. Electronic Recording of data 71
10.5. Data Management 71
10.6. Study Management Structure 71
10.6.1. Trial Steering Committee ... 71
10.6.2. Trial Management Group ... 72
10.6.3. Safety Review Committee (Safety run-in only) ... 72
10.6.4. Independent Data Monitoring Committee (Phase IIa only) ... 72
10.7. Monitoring 72
10.8. Quality Control and Quality Assurance 72
10.9. Disclosure of Data and Publication 72
11. REFERENCES 74
12. SIGNATURE PAGES 75
13. APPENDICES 79

LIST OF TABLES

Table 1: Study Plan (Safety run-in) ... 39
Table 2: Study Plan (Phase IIa) ... 41
Table 3: Pharmacokinetic Sampling Schedule for Safety run-in ... 48
Table 4: Volume of Blood to be Drawn from each Patient during Safety run-in ... 51
Table 5: Volume of Blood to be Drawn from each Patient During Phase IIa ... 52
Table 6: Dose Interventions ... 56

LIST OF FIGURES

Figure 1: Study Flow Chart – Safety run-in for both Anastrozole and Letrozole 32
Figure 2: Safety run-in - Dosing Algorithm Within a Cohort 32
Figure 3: Study flow chart Phase IIa 33
Figure 4: AZD4547 Dose Modifications for Toxicity 57
Figure 5: Management guidelines for patients with visual symptoms of ocular toxicity..................... 58
Figure 6: Toxicity management guidelines for patients with no visual symptoms of ocular toxicity... 59

LIST OF APPENDICES

Appendix A: Guidelines for Evaluation of Objective Tumour Response Using RECIST 1.1 (Response Evaluation Criteria in Solid Tumours)

Appendix B: Guidance on Potential Interactions with Concomitant Medications

ABBREVIATIONS AND DEFINITION OF TERMS

Abbreviation or term	Explanation
AE	Adverse event
AI	Aromatase inhibitor
ALT	Alanine aminotransferase
AST	Aspartate aminotransferase
$AUC_{(0-t)}$	Area under plasma concentration-time curve from zero to time t
AUC_{ss}	Area under plasma concentration-time curve during any dosing interval at steady state
AZ	AstraZeneca
bd	Bis in Die (twice daily)
C_{max}	Maximum plasma (peak) drug concentration after single dose administration
$C_{ss, max}$	Maximum (peak) steady state drug concentration in plasma during dosing interval
$C_{ss, min}$	Minimum (trough) steady state drug concentration in plasma during dosing interval
CI	Chief Investigator
CR	Complete response (RECIST)
CRF	Case report form
CSR	Clinical study report
CT	Computerised tomography
CTCAE	Common Terminology Criteria for Averse Events
ctDNA	circulating tumour specific DNA
CV	Coefficient of variation
CYP450	Cytochrome P450
DLT	Dose-limiting toxicity
DNA	Deoxyribonucleic acid
ECOG	Eastern Cooperative Oncology Group
eCRF	Electronic case report form
EDC	Electronic data capture
ECG	Electrocardiogram
ER+	Oestrogen receptor positive
FFPE	Formalin Fixed Paraffin Embedded

FGF	Fibroblast growth factor
FGF23	Fibroblast Growth Factor 23
FGFR	Fibroblast Growth Factor Receptor
FISH	Fluorescence in situ hybridization
GCP	Good Clinical Practice
GMP	Good Manufacturing Practice
IB	Investigator Brochure
IC_{50}	Concentration of a drug causing half maximal inhibitory effect
IDMC	Independent Data Monitoring Committee
ICTU	Imperial Clinical Trials Unit
IEC	Independent Ethics Committee
IMP	Investigational medicinal product
IRB	Institutional Review Board
ITTS	Intention to treat analysis set
LVEF	Left Ventricular Ejection Fraction
MedDRA	Medical Dictionary for Regulatory Activities
mL	Millilitre
mm	Millimetre
MRI	Magnetic resonance imaging
MTD	Maximum tolerated dose
MUGA	Multiple gated acquisition
NCCN	National Comprehensive Cancer Network
NE	Non-evaluable (RECIST)
NSAI	Non-steroidal aromatase inhibitor
NTL	Non-Target Lesion
OCT	Optical Coherence Tomography
ORR	Objective response rate
OS	Overall survival
PD	Progressive disease
PFS	Progression Free Survival
PI	Principle Investigator
PK	Pharmacokinetic
PD	Pharmacodynamic
PPS	Per protocol analysis set

PR	Partial response
QA	Quality assurance
QT	ECG interval measured from the onset of the QRS complex to the end of the T wave
RECIST	Response Evaluation Criteria in Solid Tumours
RNA	Ribonucleic acid
RPED	Retinal pigmented epithelium detachment
RVO	Retinal vein occlusion
SAE	Serious adverse event
SAP	Statistical Analysis Plan
SD	Stable disease (RECIST)
SOP	Standard Operating Procedure
SRC	Safety Review Committee
SRIS	Safety run-in analysis set
SSAR	Suspected Serious Adverse Reaction
SUSAR	Suspected Unexpected Serious Adverse Reaction
t_{max}	Time to reach peak or maximum concentration following drug administration
TMG	Trial Management Group
TSC	Trial Steering Committee
$t_{ss\ max}$	Time of maximum concentration at steady state
TL	Target Lesion
TSA	Tumour size assessment
ULN	Upper Limit of Normal

TRIAL SUMMARY

Title:

A single arm phase IIa study (with combination safety run-in) to assess the safety and efficacy of AZD4547 in combination with either anastrozole or letrozole in ER positive breast cancer patients who have progressed on treatment with anastrozole or letrozole – RADICAL

Primary Objectives:

Safety run-in:

- To assess the safety and tolerability and determine the dose of AZD4547 to be used in combination with a standard dose of anastrozole for the phase IIa part of the study
- To assess the safety and tolerability and determine the dose of AZD4547 to be used in combination with a standard dose of letrozole for the phase IIa part of the study

Phase IIa:

- To assess the efficacy of AZD4547 based on the change in tumour size at 12 weeks (or progression if prior to week 12), when used in combination with either anastrozole or letrozole in ER positive breast cancer patients who have progressed on treatment with either anastrozole or letrozole in any setting.

Secondary Objectives:

Safety run-in

- To assess the pharmacokinetics (PK) of anastrozole or letrozole when given alone compared to in combination with AZD4547
- To describe the PK of AZD4547 when given in combination with anastrozole or letrozole

Phase IIa

- To assess the efficacy of AZD4547 in combination with anastrozole or letrozole as measured by change in tumour size at 6 weeks, 20 weeks, then every 8 weeks, as per study plan.
- To assess the efficacy of AZD4547 in combination with anastrozole or letrozole as measured by tumour response (RECIST criteria) at 6 weeks, 12 weeks, then every 8 weeks, as per study plan.
- To assess the efficacy of the study treatment as measured by the objective response rate (ORR) at 6 weeks, 12 weeks, then every 8 weeks, as per study plan. To assess the efficacy of the study treatment as measured by progression-free survival (PFS)
- To assess the safety and tolerability of AZD4547 in combination with anastrozole or letrozole

Design:

Multi-centre, single arm, open label, phase IIa study (preceded by a safety run-in (Ib) phase)

Study Population:

Safety run-in

Post menopausal women with ER+ breast cancer who are progressing on treatment with either anastrozole or letrozole in the adjuvant or first-

line metastatic setting

Phase IIa
Post menopausal women with ER+ breast cancer who have progressed on treatment with either anastrozole or letrozole in any setting

Sample size: ***Safety run-in:*** 6-24 patients; ***Phase IIa***: 50 patients

Main Eligibility Criteria:

- Written informed consent and ability to comply with study protocol
- Aged ≥ 25 years of age
- Post menopausal women with histological confirmation of breast cancer with documented positive oestrogen receptor status (ER+) of primary or metastatic tumour tissue
- ECOG performance status 0-1 and minimum life expectancy of 12 weeks
- Fulfils criteria for previous treatment of breast cancer:
- **Safety run-in:** Relapse during a single regimen of adjuvant endocrine therapy with either anastrozole or letrozole
  or
- Progression during first line endocrine therapy with anastrozole or letrozole for advanced breast cancer
  Phase IIa: Progressing or progression at some point during breast cancer treatment on endocrine therapy with a non-steroidal AI*
  Co-administration of a targeted agent with the non-steroidal AI is permitted providing all toxicities have recovered to CTCAE Grade 1 or below.
- Prior chemotherapy in the advanced and adjuvant setting is permitted.
- Prior treatment with exemestane with or without everolimus is permitted.
  ***anastrozole or letrozole does not have to be the most recent therapy**
- **Safety run-in:** At least 1 lesion (measurable/non measurable) that can be accurately assessed by CT/MRI/plain x-ray at baseline and follow-up
  Phase IIa: At least 1 measurable lesion ≥ 10mm in longest diameter (or ≥ 15mm in the short axis for nodal disease) at baseline that can be accurately assessed by CT/MRI at baseline and follow up. Patients with bone only metastatic cancer must have a lytic or mixed lytic-blastic lesion that can be accurately assessed by CT or MRI.
- Adequate haematological, hepatic and renal function
- **Phase IIa**: Mandatory provision of tumour biopsy for assessment of oncology biomarkers
- **Safety run-in:** Study entry must be preceded by a minimum of 21 days of anastrozole or letrozole treatment

- **Phase IIa**: No restriction to duration of anastrozole or letrozole treatment prior to study entry.

Treatment: ***Safety run-ins***: Initially, patients will continue to receive the single agent treatment which they have progressed on: either anastrozole (1mg) or letrozole (2.5mg), orally, once daily for 7 days. N.B. this must be preceded by a minimum of 21 days of anastrozole or letrozole treatment prior to study entry.

Oral AZD4547 will then be added to this ongoing non-steroidal aromatase inhibitor (either anastrozole or letrozole) therapy twice daily but on an intermittent schedule of one week on/one week off.

Phase IIa: Patients will continue or restart the NSAI which they have progressed***** on: either anastrozole (1mg) or letrozole (2.5mg), orally, once daily but together with twice daily AZD4547 (80mg).
AZD4547 will be given on an intermittent schedule of one week on / one week off.

N.B. If 2 or more cases of severe toxicity (leading to permanent discontinuation of study drug) are observed in the first 6 patients, an alternative schedule of 80 mg twice daily, two weeks on / one week off will be considered but in context of emerging data from other AZD4547 studies which may suggest that this is a better tolerated schedule.

*****Prior to study entry, patients must have taken anastrozole or letrozole at some stage in their treatment to date for breast cancer; and shown evidence of resistance to this therapy. The NSAI does not have to be the most recent line of treatment.

Primary Endpoint:

Safety run-in:

- Safety and tolerability as assessed by Dose Limiting Toxicities (DLTs)

Phase IIa:

- Change in tumour size at 12 weeks (or progression if prior to week 12)

Secondary Endpoints:

Safety run-in:

- Pharmacokinetic (PK) parameters of anastrozole or letrozole when given alone and in combination with AZD4547
- PK parameters of AZD4547 when given in combination with anastrozole or letrozole.
- Safety and tolerability as assessed by adverse events (AEs)

Phase IIa:

- Change in tumour size at 6 weeks, 20 weeks, then every 8 weeks, as per study plan
- Tumour response RECIST criteria with 4 categories: complete response (CR), partial response (PR), stable disease (SD), progressive disease (PD)
- Objective response rate (ORR) with 2 categories: CR or PR, SD or PD
- Progression-free survival (PFS) is time from study entry to PD (RECIST)

Exploratory Endpoints:

- To collect and store plasma, serum and archival tumour samples or paired tumour biopsies, and analyse surplus blood or tissue, if available, for potential future exploratory research into factors that may influence development of cancer and/or

response to AZD4547 (where response is defined broadly to include efficacy, tolerability or safety)

- To investigate PK/PD relationships including serum FGF23, serum phosphate levels, serum FGF-2 (Safety run-in only)
- To analyse a range of oncology biomarkers, which may correlate with drug response
- To investigate the association between FGFR1 FISH score and the above primary and secondary efficacy endpoints (Safety run-in only)

1. BACKGROUND AND RATIONALE

1.1. Breast Cancer

Breast cancer is the second commonest cancer killer in women accounting for 12,000 deaths per year in the UK. The treatment of breast cancer is determined by the extent of the disease and a variety of other prognostic factors, including hormone receptor status. The most important factor determining response to hormonal manipulation is the presence of the oestrogen receptor (ER) in the target tissue[1]. The choice of treatment sequence is complex and dependent on a number of factors, including prior endocrine treatments received. The National Comprehensive Cancer Network (NCCN) advise that a sequence of up to 3 endocrine therapies may be appropriate before using cytotoxic chemotherapy for advanced disease[2]. The choice of first endocrine therapy is generally an anti-oestrogen such as tamoxifen or a non-steroidal aromatase inhibitor (AI) such as anastrozole or letrozole. Options for subsequent endocrine therapy include a steroidal AI such as exemestane or a selective oestrogen receptor down-regulator such as fulvestrant[3]. Irrespective of the treatment sequence, a number of patients will experience disease progression and therefore there remains a need to identify further treatment options for those patients who progress on endocrine therapy. One approach to this is to elucidate the causes of resistance to endocrine therapy so that agents can be developed which reverse endocrine resistance. Consequently, an improved understanding of the disease biology underlying this resistance is required.

Recent work has suggested that Fibroblast Growth Factor Receptor (FGFR) signalling triggered by fibroblast growth factors (FGFs) such as FGF-2 may be important. Indeed, addition of FGF-2 to breast cancer cell lines in vitro impairs the effects of non-steroidal AIs and tamoxifen whilst downregulation of FGFR1 by siRNAs sensitises breast cancer cells to these agents[4]. Moreover, FGFR1 over-expression in breast cancer is associated with poor prognosis[4]. The effects of FGF-2 in promoting resistance are not confined to anti-oestrogens or to breast cancer; it can also induce resistance to multiple cytotoxic drugs in breast and several other common cancers including lung cancer[5,6]. Our work shows that this growth factor activates MEK/Erk signalling and triggers phosphorylation and inactivation of the pro-apoptotic protein Bad. In addition, FGF-2 also upregulates the translation of several antiapoptotic proteins including Bcl-2, Bcl-XL, XIAP and cIAP1/2, in both cancer and model cell systems[6-8]. These pro-survival effects occur in MCF-7 cells that do not demonstrate FGFR amplification[4,6] (and our unpublished observations). However, about 8-20% of breast cancers display fluorescence in situ hybridisation (FISH) levels equal to 6 for FGFR1 amplification which correlates with early relapse and poor survival particularly in ER positive breast cancer[4]. This suggests that patients with ER positive breast cancer that have FGFR1 amplification might be particularly likely to benefit from FGFR inhibitor based therapies. Nevertheless, the fact that MCF7 cells that lack FGFR amplification are also sensitive to the effects of FGFR downregulation or inhibition[4,6,9] (and our unpublished observations) indicates that patients with less or no FGFR amplification may still benefit from such a therapeutic approach. Moreover, emerging data from other AZD4547 studies in a variety of tumour types have also shown that FISH6 FGFR1 amplification may not be necessary for response. Thus as we begin to study the potential efficacy of FGFR inhibitors in breast cancer, it would be important to determine whether FGFR1 amplification is really necessary as a determinant of disease response in ER positive disease.

1.2. Investigational Agent

AZD4547 is a potent and selective inhibitor of FGFR-1, 2 and 3 receptor tyrosine kinases (enzyme and cellular phosphorylation endpoints), and has a significantly lower potency for inhibition of IGF1R and KDR.

The FGFR family consists of four members each composed of an extracellular ligand binding domain, a trans-membrane domain and an intracellular cytoplasmic protein tyrosine kinase domain. Receptor activation leads to the recruitment and activation of specific downstream signalling partners that participate in the regulation of diverse processes such as cell growth, cell metabolism and cell survival. Dysregulation of the FGFR pathway via genetic modifications of FGFR-1, 2, 3 or 4, including amplification, translocation and mutations have been described in a range of tumour types including breast cancer, gastric cancer and multiple myeloma. Non-clinical data indicate the presence of such modifications confers sensitivity to FGFR inhibitors. Inhibition of FGFR mediated signalling can result in an anti-proliferative and/or pro-apoptotic activity, may have an anti-angiogenic effect, and may play a role in resistance to vascular endothelial growth factor (VEGF) inhibitor therapy. Therefore AZD4547 may have the potential to provide clinical benefit in a variety of advanced solid malignancies which have a FGFR-dependent mechanism.

1.3. Non-clinical Studies

1.3.1. In Vitro Studies

The MDA-MB-134 cell line is FGFR1 amplified (FISH=6) and the SUM52-PE cell-line is FGFR2 amplified (FISH=6) and in both lines this correlates with high expression at the protein level of the relevant FGFR. In both of these FGFR amplified cell-lines AZD4547 is a potent inhibitor of cell growth.

In addition, in low serum conditions, FGF-ligand can stimulate the growth of other breast cancer cell-lines, which do not carry an FGFR gene amplification and hence express significantly lower levels of FGF receptors, examples being MCF7, ZR-75-1 and HCC1187 cells and in these cell-lines AZD4547 inhibits FGF-stimulated proliferation (IC_{50} = 12nM, 36nM and 11 nM for inhibition of FGF-stimulated proliferation in MCF7, ZR-75-1 and HCC1187 respectively) as well as the stimulation of the downstream signalling markers phosho-Erk and phosho-FRS2.
AZD4547 did not produce any measureable in vitro inhibition of P-gp transport and as such would not be expected to cause any drug-drug interaction with substrates of this transporter in patients. (See Investigator Brochure (IB) for further details).

1.3.2. In Vivo Studies

There are no AZD4547 data for *in vivo* activity against breast cancer xenografts/models, but the *in vivo* single agent activity has been validated in several other tumour types.

1.4. Clinical Studies

Recruitment to Study D2610C00001, Study D2610C00002, Study D2610C00003, and Study D2610C00004 is complete.
Study D2610C00001, an open-label, multi-centre, dose escalation Phase I clinical study designed to assess the safety, tolerability, pharmacodynamics, PK and to determine the maximum tolerated dose (MTD) and / or recommended dose of AZD4547 in advanced cancer patients who have progressed following standard therapy or for whom no standard therapy exists, is in the reporting phase.

Study D2610C00003*****, a randomised double-blind Phase IIa study (with combination safety run-in) to assess the safety and efficacy of AZD4547 in combination with exemestane vs. exemestane alone in ER+ breast cancer patients with FGFR1 polysomy or gene amplification who have progressed following treatment with one prior endocrine therapy (adjuvant or first-line metastatic), is also in the reporting phase Enrolment to Part B of this study has also been terminated (27 March 2014) as recruitment was much slower than predicted, leading to concerns about the feasibility of completing enrolment in a realistic timeframe. This, combined with the limited evidence of clinical activity observed with AZD4547 monotherapy in FGFR-amplified gastric cancer and squamous non-

small cell lung cancer (Study D2610C00004 and Study D2610C00001), resulted in a business decision to terminate enrolment to this study.
***In response to emerging data, study D2610C00003 has since revised study design and switched from exemestane to fulvestrant as the comparator/combination agent.

Study D2610C00002 (same design as D2610C00001 but in Japanese patients) has completed recruitment and a study report is now available.

Study D2610C00004 (a randomised open-label phase IIa study in AZD4547 monotherapy versus paclitaxel in patients with advanced gastric cancer with tumours with FGFR2 polysomy or gene amplification) was terminated in June 2013; based on the results of an interim analysis. It was concluded that the study was unlikely to meet its primary objective of demonstrating superiority of AZD4547 monotherapy over paclitaxel in patients with gastric cancer with tumours that have *FGFR2* amplification (FISH score 6). A study report is now available.

In the AstraZeneca sponsored clinical development programme to date, a total of 208 patients had received at least a single dose of AZD4547 (either suspension formulation or tablet). From the phase I study, a dose of 80mg bd has been established for continuous dosing with AZD4547. All data presented here are preliminary and un-validated.

- 94 patients had received at least a single dose of AZD4547 in part A (dose escalation phase) of Study D2610C00001. 52 of these patients had received AZD4547 at a dose of 80mg .In Part A, 43 patients received a single dose of AZD4547. Cohorts were dosed with the suspension formulation as follows: 20 mg bd (n=3), 40 mg bd (n=5), 80 mg bd (n=6), 150 mg bd (n=7), 200 mg bd (n=6). Cohorts were dosed with the tablet formulation as follows: 200 mg bd (n=4), 160 mg bd (n=6),120 mg bd (n=6).
- Seven dose limiting toxicities (DLTs) have been reported: increased liver transaminases (80 mg bd), mucositis (120 bd mg) stomatitis (150 mg bd),uncontrolled phosphate (160 mg bd), acute renal failure (160 mg bd, 200 mg bd),increased ALT in association with CTCAE Grade 2 increases in phosphate and calcium:phosphate product (200 mg bd).
- The 160 mg bd dose was declared non-tolerated as 2/6 patients experienced DLTs. The Safety Review Committee decided that the 120 mg bd dose was not sufficiently tolerated to support chronic dosing, although it did not achieve the protocol defined definition of a non-tolerated dose. The MTD for AZD4547 has not been formally defined in this study.
- Part B (safety and tolerability expansion phase) of the study has been completed and 6 patients have been dosed at 80 mg bd continuous dose. No patient experienced a DLT in Part B.
- Part C (safety, tolerability and efficacy expansion phase), exploring the safety, tolerability, PK and preliminary anti-tumour activity of AZD4547 in patients with FGFR1 and/or FGFR2 gene amplified tumours, has now completed. There are 3 cohorts for Part C: Cohort 1 – 20 patients with any solid tumour with FGFR1 or FGFR2 amplification (FISH score 6), Cohort 2 – 15 patients with squamous NSCLC that have tumours with FGFR1 amplification (FISH score 6) and Cohort 3 (enrolment terminated)– 10 patients with advanced gastric adenocarcinoma (including adenocarcinoma of the lower third of the oesophagus or the gastro-oesophageal junction) with tumours that have FGFR2 amplification (FISH score 6). A total of 45 patients have been dosed at 80 mg bd (continuous dose). No patient had a DLT in Part C. Enrolment to Cohort 3 in Part C was terminated early due to review of the data from Study D2610C00004.

- 34 out of 40 patients in Part C had discontinued from the study, 22 patients due to disease progression, 8 patients due to an AE, 3 patients due to patient decision, and 1 patient due to "Other" (death).

To date, the majority of adverse events experienced by patients receiving AZD4547 have been CTCAE Grade 1 or 2 in intensity. The most commonly reported AEs (overall; all doses in all parts of the study) were constipation (43 patients); xerostomia (40 patients); stomatitis (39 patients); diarrhoea (33 patients); alopecia (32 patients); vomiting (31 patients) and decreased appetite (31 patients).

- There have been 49 SAEs reported by 25 patients; this includes 1 SAE (respiratory distress) in 1 patient who was ongoing in Part C after the date of database lock for the study. Nineteen SAEs in 12 patients were considered by the reporting investigator to be related to treatment with AZD4547. Asthenia, blood creatinine increased, chorioretinopathy, dehydration, dyspnoea, general physical health deterioration, renal failure, sepsis, and vomiting are SAE terms reported on more than one occasion.
- Four deaths related to an AE had been reported: unknown cause; euthanasia and respiratory distress, none were considered related to AZD4547 and sepsis with respiratory failure' which the reporting investigator considered was related to treatment with AZD4547. The patient had paracentesis for ascites 16 days prior to the events that may have triggered the sepsis with respiratory failure, pericardial effusion and pleural effusion. The patient's underlying metastatic gastric carcinoma with ascites also provides an alternative explanation for the occurrence of the events.

A total of 20 from 82 eligible patients have had a best response of partial response (1 patient) or prolonged stable disease (stable disease ≥7 weeks in Part A and Part B [8 patients] and stable disease ≥6 weeks in Part C [11]; total 19 patients) based on tumour assessment by RECIST 1.1. Note that Part C cluster patients are not included in the efficacy analyses.

At study completion, a total of 34 patients had received at least a single dose of AZD4547 in the Japan dose escalation study (D2610C0002).

- Cohorts were dosed with the tablet formulation as follows: 40 mg bd (n=3), 80 mg bd (n=10), 120 mg bd (n=6) and 160 mg qd (n=15).
- No DLTs have been reported to date, and the MTD has not yet been defined.
- A total of 10 patients received at least a single dose of AZD4547 at 80 mg bd.
- Thirty four patients had discontinued from the study, 19 patients due to disease progression, 9 patients due to AEs and 6 patients due to patient decision.
- All but 1 patient had reported at least one AE at the data cut-off. To date, the majority of AEs experienced by patients receiving AZD4547 have been CTCAE Grade 1 or 2 in intensity.
- The most commonly reported AEs at the AZD4547 80 mg bd dose from Part A and Part B were stomatitis (6 patients), xerostomia (5 patients), dysgeusia (5 patients), nausea (4 patients), decreased appetite (3 patients), diarrhoea (3 patients), epistaxis (3 patients), hyperphosphataemia (3 patients), malaise (3 patients), neutropenia (3 patients), and vomiting (3 patients).
- Four SAEs were reported by 3 patients: stomatitis (1 patient); nausea (1 patient); decreased appetite (2 patients). No deaths due to an AE have been reported during the study treatment.

- The best objective response observed (in the opinion of the investigator) was prolonged stable disease (stable disease≥4weeks), recorded in 22/34 patients.

Study D2610C00003 had 2 parts; a safety run-in (Part A: AZD4547 in combination with exmestane) with 4 cohorts and a randomised Phase IIa study (Part B), using AZD4547 in combination with fulvestrant.

A total of 31 patients with ER+ breast cancer received at least a single dose of AZD4547 in combination with exemestane 25 mg in the safety run-in period. In all cohorts of the safety run-in period patients have received exemestane 25 mg for 7 days prior to co-administration of exemestane 25 mg with AZD4547 bd.

- In the first cohort (AZD4547 80 mg bd continuous), all 5 patients had discontinued from AZD4547. Three due to disease progression and 2 due to an AE (nail discolouration and oedema peripheral in 1 patient; depression and neuralgia in 1 patient). No DLTs had been reported to date.
- Although the 80 mg bd AZD4547 cohort did not fulfil the protocol definition of a non-tolerated dose (2/6 patients experiencing a DLT within the 21-day combination evaluation period), the Safety Review Committee decided that the 80 mg bd dose was not appropriate for chronic dosing in this patient population due to the emerging tolerability profile.
- In the second cohort (AZD4547 40 mg bd continuous) , all 5 patients had discontinued the study (4 patients have discontinued due to disease progression and 1 patient due to an AE (chorioretinopathy)
- Twelve patients had been recruited in cohort 3 (80 mg bd intermittent schedule 1 week on AZD4547 and 1 week off treatment). All patients had discontinued the study; 6 patients due to disease progression, 5 due to AEs (dizziness, renal failure; retinal detachment and detachment of retinal pigment epithelium [2 cases]), and 1 due to patient decision.
- Nine patients had been recruited to Cohort 4 (80 mg bd intermittent schedule 2 weeks on AZD4547 and 1 week off treatment). Seven of the 9 patients have discontinued the study (3 patients due to disease progression and 2 due to AEs [detachment of retinal pigment epithelium and lethargy]) and 2 patients are ongoing.
- Part B, nine patients with ER+ breast cancer have received at least a single dose of AZD4547 (80 mg bd continuous dosing) in combination with fulvestrant 500 mg.
- To date, the majority of AEs had been CTCAE Grade 1 or 2 in intensity.
- In Part A the most commonly reported AEs were alopecia (19 patients), xerostomia (17 patients), dysgeusia (16 patients), constipation (12 patients), diarrhoea (11 patients), dry skin (11 patients) and nausea (11 patients). Data from Part B not available at this time.
- The recommended dose for the Phase IIa Period (AZD4547 80 mg bd 2 weeks on/ 1 week off) was based on an analysis of data from the safety run-in period. The most commonly reported AEs in the 9 patients receiving this treatment dose and schedule have been: dysgeusia (6 patients), alopecia (5 patients), nausea (5 patients), decreased appetite (4 patients), xerostomia (4 patients), and nail disorder (4 patients).
- There had been 20 on treatment SAEs reported from 11 patients. In Part A, these were neutropenic sepsis; anaemia; pleural effusion; device deposit issue; renal failure; asthma; dyspnoea; jugular vein thrombosis; VIIth nerve paralysis; pyelonephritis; dizziness; troponin increased; lower respiratory tract infection;; oesophageal achalasia, stomatitis and diarrhoea. Seven of these SAEs (device deposit issue, diarrhoea, dizziness, oesophageal achalasia, renal failure, stomatitis, troponin increased) were considered by the reporting investigator to be related to treatment with AZD4547).

- In Part B, theses were dizziness, inflammation, psoriatic arthropathy, and gait disturbance. None were considered by the reporting investigator to be related to treatment with AZD4547.
- No deaths due to an AE have been reported during the study treatment.

At the time of study closure, a total of 40 patients with advanced gastric cancer had received study treatment of AZD4547 (80 mg bd 2 weeks on and 1week off schedule) in the D2610C00004 study

- Thirty nine patients had discontinued the study treatment (31 due to disease progression, 3 due to AE, 2 due to patient decision, 2 due to death and 1 due to performance score of 3) and 1 patient was ongoing.
- To date, the majority of AEs experienced by patients receiving AZD4547 have been CTCAE Grade 1 or 2 in intensity. Most commonly reported AEs (of any grade) reported for patients receiving AZD4547 were: decreased appetite (16 patients), asthenia (11 patients), nausea (10 patients), constipation (10 patients), stomatitis (10 patients), abdominal pain (9 patients), abdominal pain upper (9 patients), xerostomia (9 patients), and vomiting (8 patients).
- Twelve on-treatment or post-treatment SAEs had been reported for 8 patients receiving AZD4547. One AZD4547 treatment-related SAE was reported, stomatitis in 1 patient.

Two deaths related to an AE had been reported (intestinal haemorrhage and arterial disorder), neither considered related to AZD4547.The adverse events considered to be associated with the administration of AZD4547 can be found in section 5.4 of the current IB.

1.5. Aromatase Inhibitors

1.5.1. Pharmacokinetic Considerations of the Combination Therapy

The pharmacokinetics and metabolism of letrozole and anastrozole are well understood [10]. A key question for us is whether we anticipate any interactions with AZD4547. Letrozole with continuous oral dosing reaches a steady state at 2-6 weeks. The drug is cleared through hepatic metabolism via CYP3A4 and CYP2A6 and subsequent urine clearance. Anastrozole reaches a steady state after 7 days and is 40% plasma protein bound. It is also metabolised in the liver and cleared via the kidneys. It has no effect on CYP2A6 but can inhibit CYP1A2 and CYP2C8 albeit at higher concentrations (2 logs) than clinically used. AZD4547 binding to human serum albumin and to human α1-acid glycoprotein was 93.2% and 69.6% respectively. One of the principal metabolites formed by human hepatocytes was found in the rat, but other human metabolites were not formed in significant amounts in any non-clinical species. CYP3A4, CYP3A5 and CYP2D6 are likely to be responsible for the metabolism of AZD4547 in vivo, although CYP1A1 turnover may be important in smokers. AZD4547 produced competitive inhibition of CYP3A4/5 using testosterone as the probe substrate, but not with midazolam, and it was also shown to be a time dependent inhibitor of the same isozymes.

From the above, we do not anticipate any problems with the combination of anastrozole and AZD4547. However, there is a small possibility that letrozole and AZD4547 may interact through one of their common metabolising enzymes CYP3A4. Nevertheless, Astrazeneca have in house data showing that other drugs using this pathway are unaffected by AZD4547 suggesting that this issue may not be clinically relevant. On this basis, the safety run-in part of the trial has been designed to start using the pharmacologically active, tolerated, dose defined from the currently running Phase I AZD4547 trial with the usual clinical dosing used for either anastrozole or letrozole.

1.6. Rationale

There is an increasing body of evidence implicating FGFs such as FGF-2 and their receptors in cancer biology, whereby these growth factors /receptors drive cancer cell proliferation, invasion, and

survival as well as facilitating neoangiogenesis. More recently, it has become clear that FGF-2 and its receptors FGFR1 and FGFR2 can also induce resistance to multiple chemotherapeutic agents in several cancer types. Consequently, there has been considerable interest in developing selective FGFR inhibitors. The most widely used FGFR inhibitor in preclinical studies has been PD173074. We have shown this to be selective (like AZD4547) and active in blocking the growth of multiple lung cancer cell lines in vitro as well as able to block SCLC xenograft growth in vivo[11] (and data not shown). Moreover, PD173074 blocks chemoresistance in vitro and enhances the effects of chemotherapy in vivo. There is no reason to believe that AZD4547 would behave differently and our preliminary data confirms this in lung cancer cells.

1.6.1. Rationale for use in Breast Cancer

Ectopic over expression/activation of FGFR1 in mammary cells leads to increased proliferation and invasiveness, reduced survival and cell transformation[12] and expression of an FGFR1 transgene in mice caused mammary alveolar hyperplasia and invasive lesions[13]. In the SUM44, MDA-MB-134 and CAL120 cell-lines FGFR1 gene amplification correlates with high levels of FGFR expression and activation of downstream signaling pathways including pErk and pAkt pathways[4,14]. The MDA-MB-134 cell-line is highly dependent upon FGF-ligand for growth and survival and very sensitive to growth inhibition by AZD4547 (IC50 =15nM). Exposure of SUM44 cells to FGF induces resistance to the growth inhibitory effects of tamoxifen and in both this cell-line and CAL120 down regulation of FGFR1 sensitizes to the growth inhibitory effects of tamoxifen treatment[4]. These data suggest that inhibition of FGFR1 can arrest the growth of mammary cells by both endocrine-dependent and endocrine-independent mechanisms. This together with the data presented previously provides a rationale for why AZD4547 should be tested in combination with AIs such as anastrozole and letrozole to see whether it can reverse resistance to these AIs. In addition, one of the key questions as FGFR inhibitors like AZD4547 are developed, is whether amplification of FGFR1 is really necessary to see benefit from this class of agent. Our pre-clinical data suggests that such amplification may not be necessary and this study will therefore also test this hypothesis.

1.6.2. Rationale for Revised Phase IIa Study Design

When this study was originally conceived, the standard of care for post-menopausal women with ER positive advanced breast cancer included letrozole or anastrozole therapy. Once this therapy failed, many women were then given exemestane alone before progressing to chemotherapy. However, the recent results of the Bolero trial[15] and other studies have changed the landscape and currently, many women are now being offered exemestane together with the rapalogue everolimus. This is clearly efficacious but also considerably more toxic than exemestane alone. Consequently, whether this will remain the next line of therapy following resistance to letrozole and anastrozole is very unclear particularly since a number of other novel agents are being studied in this disease setting. Therefore, in discussion with the UK breast community, the Trial Steering Committee (TSC) and Trial Management Group (TMG) felt that the most appropriate design was a simple non-randomised single arm design in which the central hypotheses of whether AZD4547 can re-establish response to either letrozole or anastrozole will be tested and whether FISH6 FGFR1 amplification is necessary for this benefit; there will also be an ongoing appraisal of safety and tolerabilty.

1.6.3. Rationale for AZD4547 Dose and Schedule (Phase IIa)

On completion of the DLT review period, the Safety Review Committee (SRC) reviewed all available safety data from both cohorts in the safety run-in part of the study and made the recommendation that 80mg bd AZD4547 one week on/one week off + NSAI combination will be carried forward to the Phase IIa part of the study.

1.6.4. Risk – Benefit Assessment

Potential Benefits

Dysregulation of the FGFR pathway is observed in a variety of cancers, due to gene amplification, translocations or mutations. Non-clinical data suggests that inhibition of FGFR mediated signalling can result in an anti-proliferative and/or proapoptotic activity and may also have an anti-angiogenic effect.

Potential Risks and their Management

Ocular Toxicity

In the clinical studies conducted to date the adverse events reported regarding the anterior aspect of the eye (dry eyes, blurred vision, conjunctivitis and keratitis) are consistent with the pathological changes that were seen pre-clinically. It is anticipated that patients will report any visual disturbances or discomfort relating to the eye in advance of any significant pathology such as ulceration occurring. The decision to continue on study treatment if mild corneal changes in the eye examination are observed will be left to the Investigator's discretion, since a patient may indicate a wish to tolerate minor discomfort if there is perceived clinical benefit from the therapy. A patient should be immediately withdrawn from study treatment if corneal ulceration occurs, and appropriate expert ophthalmologic consultation should be initiated.

Retinal pigmented epithelium detachment (RPED) has been identified in clinical studies with AZD4547 (45 occurrences as of 04 June 2014). Patients with conditions pre-disposing to the development or re-occurrence of RPED will be excluded from participation in the study. In order to detect this, patients will have a baseline ophthalmologic examination (including OCT scan) prior to initiation of study treatment and approximately monthly for the first 3 months. Thereafter, patients continuing the study treatment will have a full ophthalmological review every 8 weeks (+/- 1 week) until permanent discontinuation of AZD4547. At any other time, abnormal visual symptoms or signs will trigger a full ophthalmological review.

Mineralisation, Particularly in the Heart

The cardiac mineralisation identified in both non-clinical species is thought to be as a direct consequence of elevated serum phosphate levels. The increase in phosphate levels are thought to be pharmacological as a consequence of inhibition of FGF23 modulated phosphate homeostasis in the kidney. In the dog, the increase in phosphate level occurred prior to mineralisation, and at lower doses where no mineralisation occurred. Mineralisation was of low incidence, and was not present following 4 weeks off dose. The clinical studies to date have confirmed the pre-clinical finding of increases in serum phosphate and following review of the data hyperphosphataemia is considered to be an expected event in patients treated with AZD4547. There have been no reports, and no evidence of any soft tissue, including cardiac, mineralisation clinically. However based upon the presumption that increases in phosphate precede mineralisation, patients will be excluded from the study if they have phosphate or calcium levels above the upper limit of normal prior at time of entry. Serum phosphate and calcium are included in the standard clinical chemistry safety bloods which will be assessed on a regular basis. Any patient who experiences a doubling of phosphate from baseline or a corrected calcium:phosphate product >4.5 mmol/L should have phosphate chelation therapy initiated with a non-calcium containing agent, and weekly clinical chemistry assessments performed until resolution of the parameter to below the intervention limit. Investigators must seek appropriate specialist medical consultation (renal or metabolic) to advise on the prescription and titration of phosphate chelation agents, and to raise the patients' awareness of low phosphate diets.

It is likely that mineralisation occurring within the heart will result in functional changes prior to any gross structural changes being apparent by specific imaging technology. Therefore patients will have regular troponin I or troponin T measurements at the same time as the clinical chemistry safety blood measurements. In addition ECGs and MUGA/echocardiograms will be assessed regularly as detailed in the study plan while the patient is on study treatment in order to identify functional changes. The protocol includes standard exclusion criteria for unstable cardiac conditions and risk factors for QTc prolongation.

Renal Toxicity

In clinical studies of AZD4547 in advanced cancer populations there have been 8 renal SAEs in 7 patients. Each case may have an alternative explanation for the events, such as presence of a renal tumour, adrenal metastases, advanced cancer, and a history of listhesis. Analysis of the laboratory values for serum creatinine showed that 30% of patients have had an abnormal serum creatinine value while taking AZD4547 or during the follow-up period. Of these, 19 patients had an improvement in the serum creatinine level when AZD4547 was stopped either temporarily or permanently.

Although there are multiple alternative explanations for the observed elevated creatinine levels (such as advanced cancer patients with disease progression, nephrotoxic concomitant medications, or a history of renal failure) there appears to be a pattern in many patients where the phosphate rises in tandem with serum creatinine. The magnitude of change is not the same but the general trend upwards or downwards is matched. Intermittent dosing schedules show the effect more clearly, and the pattern can be seen in patients with normal serum creatinine values, as well as those whose creatinine rises above the upper limit of normal.

All patients should be closely monitored for any signs of impaired renal function. Patients should be excluded from the study if they have serum creatinine >1.5 times the upper limit of normal concurrent with creatinine clearance <50 mL/min (measured or estimated by Cockcroft and Gault formula). Serum creatinine and blood urea nitrogen will be included in the standard clinical chemistry safety bloods and will be assessed on a regular basis.

Bone Turnover

Histopathological changes in bone structure have been identified in the rat but not the dog. Similar bone changes have been reported in the literature following administration of another FGFR inhibitor to rats, and have been considered due to a pharmacological effect on growing bones. Patients born with mutations in FGFR genes develop a range of skeletal disorders during childhood such as osteoglyphonic dysplasia, Apert syndrome and hypochondroplastic dwarfism. Only patients over the age of 25 will be permitted to enter the study, in order to exclude individuals who have not completed maturation of their skeleton. Bone adverse events will be reviewed on a case-by-case basis as it is not possible to provide specific stopping criteria given the background of extensive metastatic disease seen with the advanced cancer patient population, which might result itself in pathological fractures and bone pain.

Mouth-related conditions

Events of ageusia, dysguesia, stomatitis/oral mucositis and xerostomia have been reported in the clinical studies to date. In cases of stomatitis particular attention should be given to prophylaxis, maintaining a high standard of oral hygiene with the regular use of antibacterial mouthwashes during the study. Saline nasal sprays may help nasal mucosal dryness and so reduce the incidence of epistaxis.

Dermatological Toxicity

There have been a number of events reported in patients receiving treatment with AZD4547 involving the skin and associated appendages. These include events of dry skin, alopecia, hair changes, trichomegaly and changes to the nails and nail beds.

Diarrhoea

Diarrhoea has been commonly reported in patients receiving AZD4547 across all studies, the majority of which have been non-serious and CTCAE Grade 1. Diarrhoea is therefore considered as an expected event in patients treated with AZD4547. Patients have responded to symptomatic treatment, for example with loperamide.

Neutropenia

Neutropenia and febrile neutropenia are common risk factors for cancer patients receiving chemotherapy treatment. A review of data for patients enrolled in AZD4547 clinical studies has not identified any dose relationship with neutropenia and indicates most events are CTCAE Grade 1 or Grade 2. However, events of CTCAE Grade 4 neutropenia have been reported and were associated with febrile events with the possibility of infection, treatment with antibiotics was given. One patient who died had febrile neutropenia but the investigator considered the patient died as a result of disease progression. Two CTCAE Grade 1 AEs were considered by the investigator to be related to treatment with AZD4547. Two SAEs with element of neutropenia have been reported (1 in Study D2610C00003 and 1 in an Investigator-Sponsored Study); however, these were not considered to be related to AZD4547 treatment.

Any AE of neutropenia should be managed as deemed appropriate by the investigator with close follow up and interruption of study drug if CTCAE Grade 3 or worse neutropenia occurs. If a patient develops febrile neutropenia, study treatment should be stopped and appropriate management including granulocyte-colony stimulating factor should be given according to local hospital guidelines.

Increases in Transaminases

Increases in transaminases have been reported in the clinical studies to date and following review of the data are considered to be expected events in patients treated with AZD4547 Most of these increases were CTCAE Grade 1 or Grade 2. There was no clear relationship between the dose of AZD4547 and the incidence or severity of the increase in transaminases.

Intermittent dosing schedules have allowed examination of the effect of dechallenge and rechallenge with AZD4547, and while there was some evidence of an effect with AZD4547 in a few patients, overall, there was little difference in the data obtained from dosing with continuous or intermittent schedules. Liver function test abnormalities were most common in patients with liver metastases and in patients with progression of their underlying cancer; multiple concomitant medications in these patients is also a confounding factor.

Studies of AZD4547 include regular measurements of ALT, AST and other hepatic biochemistry parameters. All patients should be closely monitored for any signs of of liver toxicity.

Asthenic Conditions

Literature for multi-targeted kinases suggests there may be an association between asthenic events and the use of FGFR and VEGF inhibitors; however, it is difficult to suggest there is an association directly with FGFR inhibitors.

There is also a suggestion from pre-clinical findings that FGF may modulate some of the metabolic processes such as FGF-19 (ligand for FGFR4) and FGF21 (ligand for FGFR 1, 2, 3, and 4) that can

regulate glucose, lipid, and energy metabolism and cause changes in energy expenditure; this may provide a mechanism for why asthenic events occur with FGFR inhibitors.

Preliminary findings from the data analysed within the AZD4547 clinical studies shows an incidence of asthenic reported terms of approximately 50% within the most frequently prescribed schedule of 80 mg bd continuous cohorts. There is not a dose response for asthenic AEs occurring at higher doses in the continuous schedule. Findings are inconsistent, the proportion of AEs are less severe and frequent in the 120 mg and 200 mg cohorts in comparison with those found in the 20 mg to 160 mg cohorts.

The data for asthenic events for intermittent schedules of 80 mg bd (1 week on/1 week off and 2 weeks on/1 week off) shows an incidence of approximately 45% to 50% and approximately 50% for patients receiving 80 mg continuously. There is also >50% incidence of asthenic conditions reported in the higher dosing cohorts in Study D2610C00001 and D2610C00002.

Across all the studies the time to onset for asthenic conditions overlaps amongst the varying dosing cohorts. This makes it difficult to suggest a causal relationship as the time to onset is not reduced in the higher dosing cohorts and intermittent schedules where patients are off drug for 1 week. Confounding factors such as disease progression, low haemoglobin values, comorbidities such as hypothyroidism, chronic obstructive pulmonary disease, infections and multiple concomitant medications (opiates, antihistamines, anti-hypertensive, anti-nausea) are alternative explanations for asthenic events, but no single factor can explain all cases. Common treatable causes of asthenic conditions (e.g., iron, vitamin B12 or folate deficiencies and hypothyroidism) should be investigated in patients with asthenia, fatigue or malaise.

Reproductive Organs

No reproductive toxicology or teratogenic studies have been conducted with AZD4547 to date, and it is unknown whether the drug is excreted in human milk. This study is being conducted in postmenopausal women only and so this is not considered a relevant risk in this study.

Possible Drug Interactions

AZD4547 is a substrate of CYP3A4 and CYP2D6 therefore use of inhibitors/inducers of these isoforms will be excluded from 2 weeks prior to the first dose of AZD4547 and for the duration of study treatment. CYP1A1, an isoform highly inducible by cigarette smoking, is also capable of metabolism of AZD4547 and may lead to lower exposures in smokers, therefore smoking status will be recorded as part of the demographic information for all participating patients. AZD4547 shows weak competitive inhibition of CYP3A4 and is also a time-dependent inhibitor of this isoform. This may lead to reduced metabolism (and increased exposure) of any co-administered drugs that are significantly cleared via this pathway. Concomitant use of medicines significantly metabolised by CYP3A4 will be contraindicated during the course of the study. Use of other agents less significantly metabolised will be permitted with caution if considered clinically indicated for the welfare of the patients, and patients will be closely monitored for possible drug interactions.

Overall Benefit-Risk Assessment

In the advanced cancer setting that has been chosen for this study, prolonged survival rates are very low and there is a large unmet clinical need for novel therapeutic agents.

Although there can be no certainty of clinical benefit to patients, non-clinical data with AZD4547 support the hypothesis that FGFR inhibition may be a valid target for the treatment of tumours driven via this pathway. The non-clinical safety profile has not identified any risks that would preclude

investigation in this setting, and monitoring is in place for those risks deemed to be most likely or serious.

Investigation of AZD4547 in this patient population is justified, based upon the non-clinical safety profile, the limited life expectancy due to malignant disease, and the strength of the scientific hypothesis under evaluation. Thus the benefit/risk assessment for this study supports the oral administration of AZD4547 together with anastrozole or letrozole to patients with advanced cancer, according to the proposed study design.

2. STUDY OBJECTIVES AND ENDPOINTS

2.1. Primary Objectives

Safety run-in

- To assess the safety and tolerability and determine the dose of AZD4547 to be used in combination with a standard dose of anastrozole for the phase IIa part of the study
- To assess the safety and tolerability and determine the dose of AZD4547 to be used in combination with a standard dose of letrozole for the phase IIa part of the study

Phase IIa

- To assess the efficacy of AZD4547, based on the change in tumour size at 12 weeks (or progression if prior to week 12), when used in combination with either anastrozole or letrozole in ER positive breast cancer patients who have progressed on treatment with either anastrozole or letrozole in any setting.

2.2. Secondary Objectives

Safety run-in

- To assess the pharmacokinetics (PK) of anastrozole or letrozole when given alone compared to in combination with AZD4547
- To describe the PK of AZD4547 when given in combination with anastrozole or letrozole

Phase IIa

- To assess the efficacy of AZD4547 in combination with anastrozole or letrozole as measured by change in tumour size at 6 weeks, 20 weeks, then every 8 weeks, as per study plan.
- To assess the efficacy of AZD4547 in combination with anastrozole or letrozole as measured by the tumour response (RECIST criteria) at 6 weeks, 12 weeks, then every 8 weeks, as per study plan.
- To assess the efficacy of the study treatment as measured by the objective response rate (ORR) at 6 weeks, 12 weeks, then every 8 weeks, as per study plan.
- To assess the efficacy of the study treatment as measured by progression-free survival (PFS)
- To assess the safety and tolerability of AZD4547 in combination with anastrozole or letrozole

2.3. Primary Endpoints

Safety run-in

- Safety and tolerability as assessed by DLTs

Phase IIa

- Change in tumour size at 12 weeks (or progression if prior to week 12)

2.4. Secondary Endpoints

Safety run-in

- PK parameters of anastrozole or letrozole when given alone and in combination with AZD4547
- PK parameters of AZD4547 when given in combination with anastrozole or letrozole
- Safety and tolerability as assessed by AE

Phase IIa

- Change in tumour size at 6 weeks, 20 weeks, then every 8 weeks, as per study plan
- Tumour response RECIST criteria with 4 categories: complete response (CR), partial response (PR), stable disease (SD), progressive disease (PD)
- Objective response rate (ORR) with 2 categories: CR, PR, SD versus PD.
- Progression-free survival (PFS) is time from study entry to PD (RECIST)

2.5. Exploratory Objectives / Endpoints

- To collect and store plasma, serum and archival tumour samples or paired tumour biopsies and analyse surplus blood or tissue, if available, for potential future exploratory research into factors that may influence development of cancer and/or response to AZD4547 (where response is defined broadly to include efficacy, tolerability or safety)
- To investigate PK/PD relationships including serum FGF23, serum phosphate levels, serum FGF-2 (safety run-in only)
- To analyse a range of oncology biomarkers, which may correlate with drug response
- To investigate the association between FGFR1 FISH score and the above primary and secondary efficacy endpoints (safety run-in only)

3. STUDY DESIGN

This study protocol has undergone peer review as part of the process of endorsement by the Cancer Research UK New Agents Committee

3.1. Overall Study Design

This is a phase IIa (with safety run-in), multi-centre, open label, single arm study of AZD4547 administered orally together with anastrozole or letrozole in ER+ breast cancer patients who have progressed on treatment with anastrozole or letrozole; in adjuvant or first line metastatic (safety run-in only) or any setting (phase IIa only). There are two parts to this study: an initial safety run-in followed by a phase IIa study.

Safety run-in: Two safety run-ins will be conducted to assess the safety and tolerability of AZD4547 in combination with anastrozole and in combination with letrozole. Between 3 and 12 patients will be enrolled into each of the safety run-in parts of the study (i.e. 6 to 24 in total). In both parts, the first cohort will receive 80 mg bd AZD4547. The total number of patients will depend upon the number of cohorts necessary (see Figure 1). A cohort will have a minimum of 3 and maximum of 6 patients (see

Figure 2). The Safety Review Committee (SRC) will determine the dose de-escalation scheme, whether further cohorts are required, and ultimately select the safe and tolerated dose of AZD4547 to be used in the phase IIa study. A different dose level of AZD4547 may be selected for combination with anastrozole than for letrozole.

Phase IIa: 50 patients will be recruited to receive study treatment (see Figure 3).

3.2. Treatment Regimens

Safety run-in:

Initially, patients will continue to receive the single agent treatment which they have progressed on: either anastrozole (1mg) or letrozole (2.5mg), orally, once daily for 7 days. N.B. this must be preceded by a minimum of 21 days of anastrozole or letrozole treatment prior to study entry.

Oral AZD4547 will then be added to this ongoing non-steroidal aromatase inhibitor therapy twice daily but on an intermittent schedule of one week on/one week off.

Phase IIa:

Patients will continue or restart the NSAI which they have progressed***** on: either anastrozole (1mg) or letrozole (2.5mg), orally, once daily but together with twice daily AZD4547 (80mg); the confirmed dose level for AZD4547 determined during the safety run-in part of the study.

*****Prior to study entry, patients must have taken anastrozole or letrozole at some stage in their treatment to date for breast cancer; and shown evidence of resistance to this therapy. The NSAI does not have to be the most recent line of treatment.

AZD4547 will be given on an intermittent schedule of one week on / one week off. This schedule has been well tolerated in the preceding safety run-in part of the study. However, if 2 or more cases of severe toxicity (leading to permanent discontinuation of study drug) are observed in the first 6 patients, the Independent Data Monitoring Committee (IDMC) will consider an alternative schedule of two weeks on / one week off, if emerging data from other AZD4547 studies suggest that this is a better tolerated schedule.

3.3. Study Flow Charts

Figure 1: Study Flow Chart – Safety run-in for both Anastrozole and Letrozole

Cohort 1

7 day NSAI[1] run-in	80mg bd AZD4547 one week on/one week off + NSAI[1] combination

Cohort 2

7 day NSAI[1] run-in	< 80mg bd AZD4547 one week on/one week off + NSAI[1] combination

[1]anastrozole or letrozole

Figure 2: Safety run-in - Dosing Algorithm Within a Cohort

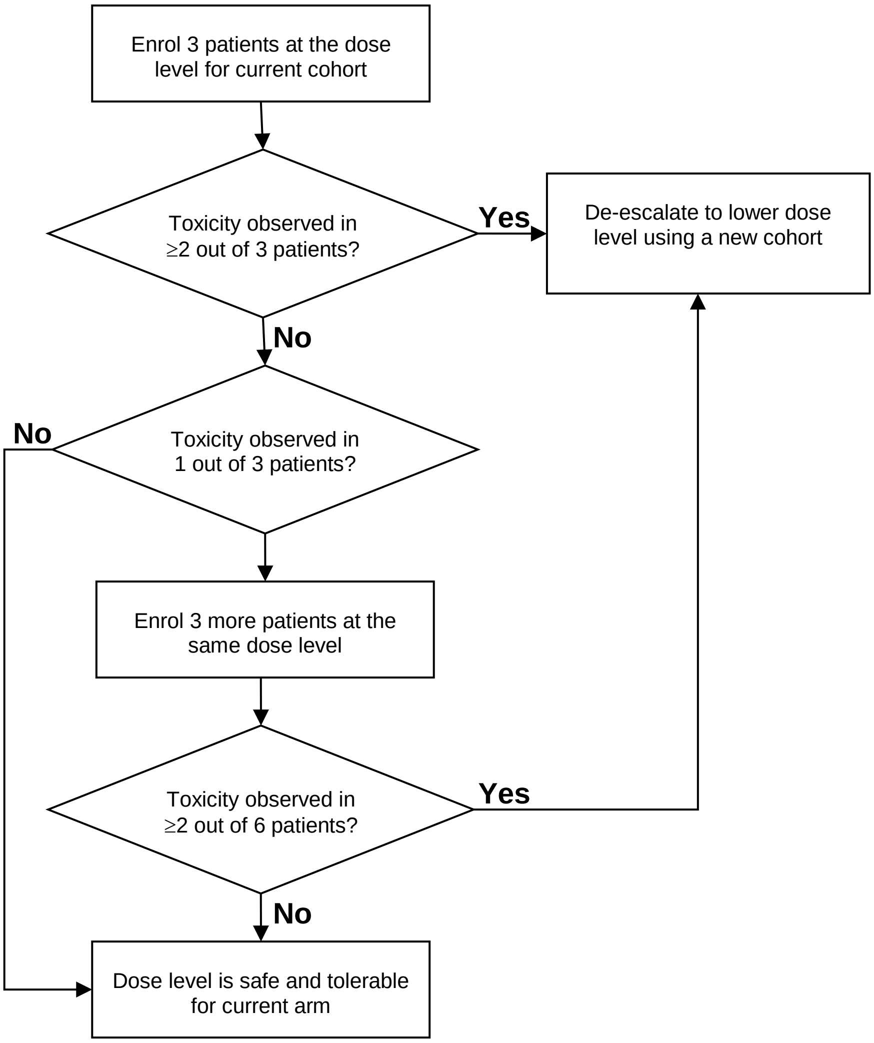

Figure 3: Study flow chart Phase IIa

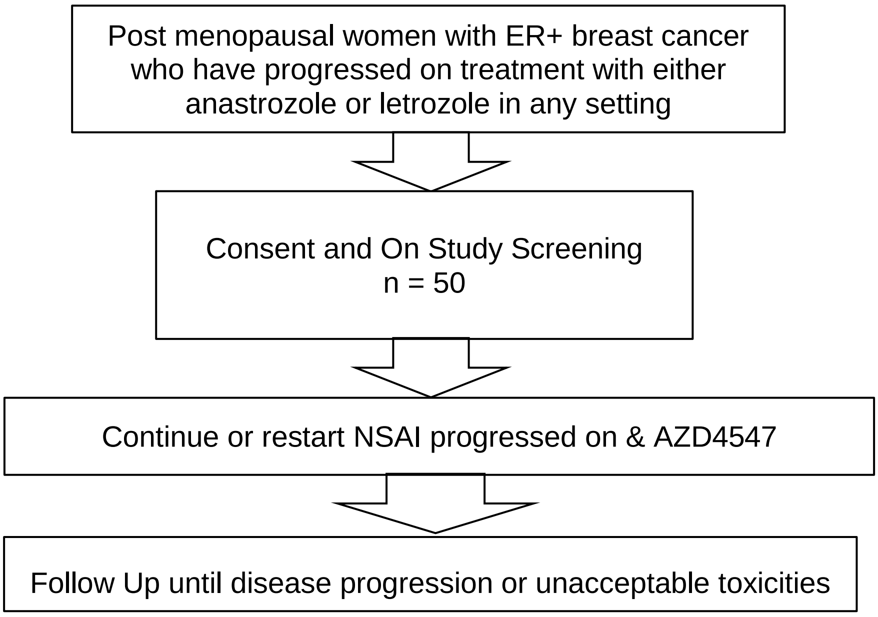

3.4. Follow-up

All patients on AZD4547 and either anastrozole or letrozole should be followed for 28 days after the last dose of study treatment for complete safety data.

3.5. Study Termination

The study will be terminated when:

- All patients on AZD4547 and either anastrozole or letrozole combination therapy have permanently discontinued study medication due to progression or unacceptable toxicity, and completed 28 days follow-up for collection of safety data

3.5.1. Treatment after Study Termination

Following participation in the study, patient care will be decided by their local doctor according to usual practice.

4. PATIENT SELECTION AND RECRUITMENT

4.1. Screening and Enrolment

Each patient will undergo screening during the 28 days prior to admission to confirm eligibility. Tumour assessments and other clinical data obtained as standard of care prior to consent may be used for the study provided they comply with the protocol specified timelines. Written informed consent will be obtained before the subject undergoes any study specific procedures.

Each potential patient will be assigned a unique identifier number for use during the trial. A complete record of all patients who enter screening for the study, and also those who go on to be enrolled, must be maintained at each site. The local investigator is responsible for ensuring that this record includes the allocated trial ID as well as the patient identifiable data including name, hospital number and date of birth.

Eligible patients who take part in the study must meet all of the listed inclusion criteria and none of the exclusion criteria. NB. Some criteria are relevant for the safety run-in or phase IIa parts of the study only; these are marked accordingly.

4.2. Subject Selection

4.2.1. Inclusion Criteria

Patients must fulfil all of the following criteria.

1. Written (signed and dated) informed consent and be capable of co-operating with treatment and follow-up
2. Aged ≥ 25 years of age (N.B. in line with other studies with AZD4574 and due to concerns of possible effects on the immature skeleton)
3. Post menopausal women. Women will be considered postmenopausal if they have had a bilateral oophorectomy or the following specific requirements apply:

   Safety run-in:
   - Women under 50 years old would be considered post-menopausal if they have been amenorrhoeic for 24 months and have follicle-stimulating hormone (FSH) and oestradiol levels in the post-menopausal range. Patients with prior exposure to depot LHRH analogues must be 24 months or more following the last administration
   - Women aged 50 years and older would be considered post-menopausal if they have been amenorrhoeic for 12 months and patients with prior exposure to depot LHRH analogues must be 12 months or more following the last administration
   - Women rendered amenorrhoeic by adjuvant chemotherapy, who were premenopausal or perimenopausal prior to chemotherapy, must have been amenorrhoeic for at least 24 months

   Phase IIa:
   - Women under 50 years old would be considered post-menopausal if they have been amenorrhoeic for 24 months and have follicle-stimulating hormone (FSH) and oestradiol levels in the post-menopausal range.
   - Women aged 50 years and older would be considered post-menopausal if they have been amenorrhoeic for 12 months
   - Women rendered amenorrhoeic by adjuvant chemotherapy, who were premenopausal or perimenopausal prior to chemotherapy, must have been amenorrhoeic for at least 24 months
   - Perimenopausal women rendered amenorrhoeic from exposure to depot LHRH analogues*

   *Patients must have taken LHRH analogues for at least 6 months

4. Eastern Cooperative Oncology Group (ECOG) performance status 0-1 with no deterioration over the previous 2 weeks and minimum life expectancy of 12 weeks
5. Histological confirmation of breast cancer with documented positive oestrogen receptor status (ER+) of primary or metastatic tumour tissue according to local laboratory parameters
6. **Phase IIa**: Mandatory provision of tumour biopsy for assessment of oncology biomarkers
7. Fulfils criteria for previous treatment of breast cancer*:

   Safety run-in:
   - Relapse during a single regimen of adjuvant endocrine therapy with either anastrozole or letrozole
     or
   - Progression during first line endocrine therapy with a non-steroidal AI for advanced breast cancer**. Co-administration of a targeted agent with the non-steroidal AI is permitted providing all toxicities have recovered to CTCAE Grade 1 or below

     1 prior regimen of chemotherapy in the advanced setting is permitted. Chemotherapy administered in the adjuvant setting is permitted

   Phase IIa:
   - Progressing or progression at some point during breast cancer treatment on endocrine therapy with a non-steroidal AI.*** Co-administration of a targeted agent with the non-steroidal AI is permitted providing all toxicities have recovered to CTCAE Grade 1 or below.
   - Prior chemotherapy in the advanced and adjuvant setting is permitted.
   - Prior treatment with exemestane with or without everolimus is permitted.

   *HER2-positive breast cancer patients should have been offered at least one prior line of HER2 directed therapy
   **Advanced breast cancer: metastatic disease or locally advanced disease which is not amenable to treatment with curative intent
   ***anastrozole or letrozole does not have to be the most recent therapy

8. **Safety run-in:** At least one lesion (measurable and/or non-measurable) that can be accurately assessed by CT/MRI/plain x-ray at baseline and follow up visits

   Phase IIa: At least one lesion ≥ 10mm in the longest diameter at baseline (or ≥ 15mm in the short axis for nodal disease) that can be accurately measured with CT/MRI at baseline and is suitable for accurate repeated measurements. Patients with bone only metastatic cancer must have a lytic or mixed lytic-blastic lesion that can be accurately assessed by CT or MRI.
9. **Safety run-in:** Study entry must be preceded by a minimum of 21 days of anastrozole or letrozole treatment

   Phase IIa: No set duration of anastrozole or letrozole treatment prior to study entry.

4.2.2. Exclusion Criteria

1. Treatment with any of the following:
   a. **Safety run-in:** more than 1 regimen of endocrine therapy for advanced breast cancer
   b. previous exposure to any FGFR inhibitor
   c. **Safety run in**: more than 1 prior regimen of chemotherapy for advanced breast cancer.
   d. potent inhibitors or inducers of CYP3A4 or CYP2D6, or substrates of CYP3A4 within 2 weeks prior to first dose of study treatment (3 weeks for St John's Wort)
   e. major surgery within 4 weeks prior to first dose of study treatment
   f. radiotherapy with a wide field of radiation within 4 weeks prior to first dose of study treatment; or radiotherapy with a limited field of radiation for palliation within 2 weeks before the first dose of study treatment
2. With the exception of alopecia, any unresolved toxicities from prior therapy greater than CTCAE grade 1 at time of starting study
3. Spinal cord compression or brain metastases unless asymptomatic, treated and stable and not requiring steroids for at least 4 weeks prior to start of study treatment
4. Any evidence of severe or uncontrolled systemic diseases or active infection
5. Any of the following cardiac criteria:
   a. Resting corrected QT interval (QTc) >470 ms
   b. Any clinically important abnormalities in rhythm, conduction or morphology of resting ECG e.g. complete left bundle branch block, third degree heart block
   c. Any factors that increase the risk of QTc prolongation or risk of arrhythmic events such as heart failure, hypokalaemia, congenital long QT syndrome, family history of long QT syndrome or unexplained sudden death under 40 years of age or any concomitant medication known to prolong the QT interval
6. Inadequate bone marrow reserve or organ function as defined by any one of the following parameters:

   Haemoglobin < 9.0 g/dL (<90.0 g/L)
   Absolute neutrophil count (ANC) < 1.5 x 10^9 /L
   Platelet count < 100 x 10^9 /L
   Alanine aminotransferase > 2.5 x ULN if no demonstrable liver metastases or > 5 x ULN in the presence of liver metastases
   Aspartate aminotransferase > 2.5 x ULN if no demonstrable liver metastases or > 5 x ULN in the presence of liver metastases
   Total bilirubin > 1.5 x ULN if no demonstrable liver metastases or > 3 x ULN in the presence of liver metastases
   Creatinine > 1.5 times ULN or creatinine clearance <50ml/min
   Corrected calcium > ULN
   Phosphate > ULN

7. Refractory nausea and vomiting, chronic gastrointestinal diseases, inability to swallow the formulated IMP or previous significant bowel resection that would preclude absorption of AZD4547 or anastrozole or letrozole
8. History of hypersensitivity to anastrozole or letrozole
9. History of another malignancy within 5 yrs prior to starting study treatment, except adequately treated basal or squamous cell carcinoma of the skin, carcinoma of the cervix and the disease under study

10. Any of the following ophthalmological criteria:*
    - Current evidence or previous history of retinal pigmented epithelium detachment (RPED)
    - Previous laser treatment or intra-ocular injection for treatment of macular degeneration
    - Current evidence or previous history of soft drusen, drusenoid RPE detachment and wet macular degeneration.
    - Current evidence or previous history of retinal vein occlusion (RVO)
    - Current evidence or previous history of retinal degenerative diseases (e.g. hereditary)

    *Patients with uncontrolled glaucoma or intra-ocular pressure >21 mmHg at screening should be referred for ophthalmological management and the condition controlled prior to first dose of study treatment.

11. Concurrent treatment with another investigational agent or use of another investigational agent within 30 days or 5 half lives, whichever is longer, preceding the first dose of study treatment
12. Concurrent treatment with prohibited medications and wash out period for that drug will not have been completed before starting study medication (see Appendix B)

5. STUDY PLAN AND PROCEDURES

5.1. Study Schedules

Scheduled visits are uniquely identified by study period, and day within the period. The combination treatment period of the safety run-in study and the phase IIa study are further partitioned into consecutively numbered treatment cycles of 28 days duration. The period and day when scheduled visits occur, as well as, observations and assessments required during the study are summarised in Table 1 for the safety run-in and Table 2 for phase IIa.

In the phase IIa study, unless indicated otherwise, scheduled assessments may take place within ±1 day of the scheduled day, e.g, laboratory evaluations scheduled for cycle 1 day 7 may take place on cycle 1 day 6 or day 8. Assessment days will all be relative to the start of study treatment, i.e.Cycle 1 Day 1.

If a patient has AZD4547 treatment breaks, please contact the Study Team for advice regarding appropriate timing of PD biomarker assessments. All other assessments, including laboratory safety assessments, vital signs and RECIST should continue to be performed as described in the appropriate sections of the protocol, relative to the baseline assessments

Table 1: Study Plan (Safety run-in)

Study Period	Screening	NSAI[1] monotherapy		AZD4547 + NSAI[1] combination								AZD4547 / NSAI[1] Discont.	28-day follow-up
Cycle	n/a	n/a		Cycle 1				Cycle 2		Cycle 3	Cycle 4 (onwards)		
Activity / Day	-28 to -1	1	7	1	7	15	21	1	15	1	1		
Inclusion/exclusion criteria & informed consent	X												
Demographics	X												
Medical history / Concomitant medical conditions	X	X	X	X	X	X	X	X		X	X	X	
Radiotherapy (previous/current) [2]	X	X	X	X	X	X	X	X		X	X	X	X
Chemotherapy (previous/current)	X												X
Surgical History	X												
Disease Extent/Tumour Characteristics	X												
Concomitant medication	X	X	X	X	X	X	X	X		X	X	X	X
Prohibited medication	X												
Physical examination	X	X	X					X		X	X	X	
ECOG performance status	X	X	X		X	X	X	X		X	X	X	X
Vital signs	X		X					X		X	X	X	X
ECG	X				X			X		X	X	X	
Echo / MUGA scan	X							X[3]				X	
Laboratory evaluations (blood/urine)	X		X		X	X	X	X		X	X	X	X

Study Period	Screening	NSAI[1] monotherapy		AZD4547 + NSAI[1] combination								AZD4547 / NSAI[1] Discont.	28-day follow-up
**Cycle**	**n/a**	**n/a**		**Cycle 1**				**Cycle 2**		**Cycle 3**	**Cycle 4 (onwards)**		
**Activity / Day**	**-28 to -1**	**1**	**7**	**1**	**7**	**15**	**21**	**1**	**15**	**1**	**1**		
Ophthalmic assessment	X							X		X	X[4]	X	
Treatment compliance			X	X	X	X	X	X		X	X	X	
PI disease status assessments	X								X		X		
Pharmacokinetic sampling			X		X								
Blood sample PD biomarkers	X		X		X	X	X					X	
Treatment dosing		Treatment will be administered according to details in Section 6.2.3.											
DLT assessment				X	X	X	X	X					
Adverse events		X	X	X	X	X	X	X		X	X	X	X
Archival tumour (optional)	X												
Paired tumour biopsy (optional)	X							X[5]					
Pharmacogenetic sample (optional)		X											

[1] Either anastrozole or letrozole
[2] **ONLY** radiation for palliation at focal sites is permitted whilst the patient is on study medication
[3] After the cycle 2, day 1 assessment, MUGA/ECHO should be performed every three months i.e. at the end of cycles 4, 7 etc. and at discontinuation of AZD4547.
[4] After first 3 months of AZD4547, ophthalmological review should be every 8 weeks (+/- 1 week) until permanent discontinuation of AZD4547
[5] The second biopsy should be collected during cycle 2, but as close to completion of 1st cycle of study treatment as possible

Notes: Activities in italic are unique to Safety run-in study.

Table 2: Study Plan (Phase IIa)

Study period	Screening	AZD4547 + NSAI[1] combination									AZD4547 / NSAI[1] Discont.	28-day follow-up
Cycle	n/a	Cycle 1				Cycle 2		Cycle 3	Cycle 4	Cycle 5 (onwards)		
**Activity / Day**	**-28 to -1**	**1**	**8**	**15**	**22**	**1**	**15**	**1**	**1**	**1**		
Inclusion/exclusion criteria & informed consent	X											
Archival tumour[2] sample (mandatory) / exploratory (optional)	X											
Demographics	X											
Medical history / Concomitant medical conditions	X	X	X	X	X	X	X	X	X	X	X	
Radiotherapy (previous/current) [3]	X	X	X	X	X	X	X	X	X	X	X	X
Chemotherapy (previous/current)	X											X
Endocrine Therapy (previous)	X											
Targeted Therapy (previous)	X											
Surgical History	X											
Disease Extent/Tumour Characteristics	X											
Concomitant medication	X	X	X	X	X	X	X	X	X	X	X	X
Prohibited medication	X											
Physical examination	X	X		X		X		X	X	X	X	
ECOG performance status	X	X	X	X	X	X	X	X	X	X	X	X
Vital signs	X	X		X		X		X	X	X	X	X
ECG	X		X			X		X	X	X	X	
Echo / MUGA scan	X							X[4]			X	

**Study period**	**Screening**	**AZD4547 + NSAI[1] combination**									**AZD4547 / NSAI[1] Discont.**	**28-day follow-up**
**Cycle**	**n/a**	**Cycle 1**				**Cycle 2**		**Cycle 3**	**Cycle 4**	**Cycle 5 (onwards)**		
**Activity / Day**	**-28 to -1**	**1**	**8**	**15**	**22**	**1**	**15**	**1**	**1**	**1**		
Laboratory evaluations (blood/urine)	X	X	X	X	X	X	X	X	X	X	X	X
Ophthalmic assessment	X					X		X	X[5]		X	
Treatment compliance			X	X	X	X		X	X	X	X	
Tumour assessments as per RECIST 1.1	X						X		X[6]		X	
Blood sample PD biomarkers[7]	X		X	X	X	X		X	X	X	X	
Treatment dosing		Treatment will be administered according to details in Section 6.2.3.										
Adverse events	X[8]	X	X	X	X	X	X	X	X	X	X	X
Paired tumour biopsy (optional)	X[9]				X[10]							
Pharmacogenetics sample (optional)		X										
Circulating tumour specific DNA (ctDNA) (optional)	X[11]	X	X	X	X	X	X	X	X	X	X	X

[1] Either anastrozole or letrozole
[2] If an archival sample is not available, a fresh tumour biopsy sample must be taken.
[3]**ONLY** radiation for palliation at focal sites is permitted whilst the patient is on study medication
[4] After the cycle 3, day 1 assessment, MUGA/ECHO should be performed every three months i.e. at the end of cycles 5, 8 etc. and at discontinuation of AZD4547.
[5] After first 3 months of AZD4547, ophthalmological review should be every 8 weeks (+/- 1 week) i.e. at the end of cycles 5, 7 etc. and at discontinuation of AZD4547
[6] Tumour assessments to be carried out at baseline, week 6, week 12, then every 8 weeks until disease progression or permanent discontinuation of study treatment and finally at AZD4547 discontinuation visit.
[7] If a patient has AZD4547 treatment delays, please contact the Study Team for advice regarding appropriate timing of PD biomarker assessments
[8] AEs collected from the point the patient has been confirmed to be eligible by the RADICAL study team
[9] The first biopsy must only be taken once the patient has been confirmed to be eligible by the RADICAL study team.
[10] The second biopsy **must be taken within 18 hours** of administration of previous AZD4547 dose
[11] Sample to be taken within 1 week of Cycle 1 day 1

5.2. Procedures and Measurements

5.2.1. Demographic Data

Subject date of birth, race / ethnicity and smoking status (smokes or not / habitual or occasional) will be collected at screening.

5.2.2. Medical History / Concomitant Medical Conditions

Both past medical history and concomitant medical conditions will be collected. However, information on radiotherapy, chemotherapy, endocrine therapy, targeted therapy, surgery and medical history of breast cancer including characteristics of the primary and / or metastatic tumour(s), will be documented separately (see below). Concurrent diseases i.e. other medical conditions that are ongoing from the start of the study will be documented in Adverse Events if they worsen.

5.2.3. Previous and Current Radiotherapy

Radiotherapy includes all treatments prior to study entry, whilst on the study, or post permanent discontinuation of study treatment. The following will be collected: site or region (breast, local lymph nodes, distant lymph nodes, bone, brain, or other), setting (neoadjuvant; adjuvant; metastatic), range of field given (wide or limited) and start and end dates of treatment.

5.2.4. Previous and Current Chemotherapy

Chemotherapy includes all treatments prior to study entry or post permanent discontinuation of study treatment. The following will be collected: agent / regimen, site (breast, lung, liver, bone, brain, other) route of administration (IV or tablet), setting (neoadjuvant; adjuvant; metastatic) and start and end dates of treatment.

5.2.5. Endocrine Therapy

Endocrine therapy includes all treatments prior to study entry. The following will be collected: setting (neoadjuvant; adjuvant; metastatic), treatment name (tamoxifen; anastrozole; letrozole; exemestane; other) and start and end dates of treatment.

5.2.6. Targeted Therapy

Targeted therapy includes all treatments prior to study entry. The following will be collected: setting (neoadjuvant; adjuvant; metastatic), treatment name and start and end dates of treatment.

5.2.7. Surgical History

Details of any past surgery that the patient has undergone as part of their treatment of cancer will be collected, i.e. surgical event, reason for surgery and date of surgery.

5.2.8. Medical History of Breast Cancer

Details will be collected on the extent of the patient's disease. The following details will be collected: date of original breast cancer diagnosis, indication of all known sites of locally advanced and metastatic disease, date of most recent progression / recurrence.

5.2.9. Characteristics of the Primary or Metastatic Breast Tumour

The following details will be collected on the characteristics of the tumour: whether tumour was primary or metastatic, location of tumour (if metastatic), histological confirmation of breast cancer tumour (if metastatic), histology of tumour grade, TNM classification of tumour, cancer stage of tumour and receptor status of tumour.

5.2.10. Concomitant Medications

All medications, with the exception of prohibited medications, that are being taken in the 4 weeks prior to starting study treatment (including those during screening) and those taken whilst on study will be documented as a concomitant medication;. The following details will be collected: drug name, reason for therapy, therapy dosage / units, frequency of therapy, route of administration, start and end date of therapy

5.2.11. Prohibited Study Medication

Medications defined as prohibited for use during the study according to Appendix B, “Guidance on Potential Interactions with Concomitant Medications” will be documented during screening. Patients must stop taking any prohibited medications and have completed the required “washout” period prior to starting AZD4547, as per exclusion criterion 12.

5.2.12. Physical Examination

A complete physical examination will be performed, as per local practice at the scheduled visits indicated in the Study Plan. The following examinations should be undertaken: general appearance, skin, head and neck, lymph nodes, thyroid, musculoskeletal/extremities, cardiovascular, respiratory, abdomen and neurological. The outcome of the examinations will be assessed as normal or abnormal, and whether clinically significant.

5.2.13. ECOG Performance Status

Performance status will be assessed at the scheduled visits indicated in the Study Plan according to ECOG criteria as follows:

0 = Fully active, able to carry on all predisease activities without restriction

1 = Restricted in physically strenuous activity but ambulatory and able to carry out work of a light or sedentary nature. For example, light housework, office work

2 = Ambulatory and capable of all self care but unable to carry out any work activities. Up and about more than 50% of waking hours

3 = Capable of only limited self-care, confined to bed or chair 50% or more of waking hours

4 = Completely disabled. Cannot carry on any selfcare. Totally confined to bed or chair

5 = Death

5.2.14. Vital Signs

Vital signs including weight, pulse and blood pressure will be measured at the scheduled visits indicated in the Study Plan. Vital signs may be assessed at any time during the visit; however, supine blood pressure and pulse should be measured after 10 minutes rest. Height will be measured at initial screening visit only.

5.2.15. ECG

A standard 12-lead ECG will be performed as indicated in the Study Plan. The patient should be examined with the same machine throughout the study.

ECGs will be recorded at the following time points:

- Screening
- Cycle 1 day 8: 2 hours post morning dose of study medication (AZD4547 + either anastrozole or letrozole)
- Cycle 2 day 1 (and each subsequent cycle): any time on the day of assessment

- At discontinuation of AZD4547 + either anastrozole or letrozole therapy

Details of rhythm, ECG intervals and an overall evaluation will be collected.

5.2.16. Echocardiogram and/or MUGA Scan

An echocardiogram and / or MUGA scan to assess left ventricular ejection fraction (LVEF) will be performed at screening, Cycle 3 day 1 (± 1 week), then every 3 months (± 1 week) and finally at AZD4547 discontinuation visit.

5.2.17. Laboratory Evaluations

Blood and urine samples for haematology, clinical chemistry and urinalysis will be taken at scheduled visits and analysed at the local laboratory using standard methods for routine tests. NB. ***On dosing days for all cycles, samples must be taken pre-dose***; as on these visit days, samples must be processed, and results available, prior to administration of the first daily dose of AZD4547 to ensure an assessment of patient suitability to resume dosing is undertaken.

The following variables will be measured:

Clinical Chemistry: ALT, AST, Alkaline phosphatase, Bilirubin (total), Corrected Calcium (total), Creatinine (total), Random glucose, Magnesium, Phosphate, Potassium, Sodium, Troponin I or Troponin T, Urea nitrogen, Albumin

Haematology: Haemoglobin, Leukocyte, Neutrophils, Lymphocytes, Platelets

Urinalysis: Glucose, Protein, Blood

Urinalysis parameters will be measured using a dipstick test. If there are any abnormalities found the urine sample will be sent for Micro Culture Sensitivity Testing

N.B. Management of patients will be according to the corrected calcium result and corrected calcium:phosphate product. Where corrected calcium is not provided by the site laboratory the following formula should be used:
Corrected Calcium = Total Calcium (mmol/L) + ([40 – Albumin (G/L)] x 0.02)

The corrected calcium phosphate product ($Ca:PO_4$) is calculated by multiplying the corrected calcium result by phosphate result:
$Ca:PO_4$ = Phosphate (mmol/L) x ((Total Calcium (mmol/L) + ([40 – Albumin (G/L)] x 0.02))

Laboratory values that have changed significantly from baseline and are considered to be of clinical concern must be recorded as an adverse event and followed up as appropriate.

5.2.18. Ophthalmic Assessment

An ophthalmic assessment will be performed by an ophthalmic expert at screening, Cycle 2 day 1, Cycle 3 day 1 and Cycle 4 day 1 (+/- 3 days), as shown in the study plan (Table 1 and Table 2). Thereafter, patients continuing the study treatment should have a full ophthalmological review every 8 weeks (+/- 1 week) and finally at AZD4547 discontinuation visit. At any other time, abnormal visual symptoms or signs will trigger a full ophthalmological review. Algorithms for further management are provided in Figure 5 and Figure 6 in Section 6.2.8 “Dose Modifications for AZD4547”.

The ophthalmic assessment should be performed on each occasion by the same ophthalmic expert where possible.

The following assessments will be performed in the order stated:

(i) Visual acuity (best corrected) including near and distance vision for each eye separately

(ii) Amsler grid

(iii) Schirmer's test without anaesthesia – read after 5 minutes (this test should be done before instillation of stains or dilatory agents)

(iv) Slit lamp examination:
- Apply 1 drop of 2% fluorescein followed by 1 drop of normal saline
- Measure intra-ocular pressure
- Photograph any abnormalities

(v) Fundoscopy and lens examination following pupil dilatation should be performed using binocular equipment and a 78 dioptre lens (or nearest available equivalent lens)

(vi) OCT scans of the macula area of both eyes should be performed at screening and monthly for the first 3 months on study treatment. After this time, an OCT scan should be performed on the occurrence of clinical symptoms or signs suggestive of RPED. OCT is the preferred methodology for diagnosis of RPED. If OCT is not available as part of local clinical practice, an equivalent alternative diagnostic methodology to screen for RPED should be used.

Clinically significant abnormalities detected during ophthalmic assessments should be reported as AEs. The patient should be managed under the care of a competent ophthalmologist with appropriate medication and followed up until the condition has resolved.

A central review of ophthalmology reports may be undertaken throughout the study.

5.2.19. Treatment Compliance (Patient Diary)

Patients will keep a detailed record of all study medication that they take in their patient diaries. Date and time of administration, drug name and drug dose will be collected.

5.2.20. Principle Investigator Disease Status Assessments (Safety Run-In only)

Principle Investigator (PI) disease status assessments will be performed using CT, MRI or plain x-ray to assess any progression of the patient's disease. The clinician must document: date of assessment, imaging method used (X-ray, CT or MRI), site (breast, lung, liver, pleural effusion, local lymph nodes, distant lymph nodes, bone, brain or other) and outcome of assessment. Disease status assessments will be performed at screening, Cycle 2 day 15 and Cycle 4 day 1.

5.2.21. Tumour Assessments (Phase IIa only)

Tumour assessments will be performed using CT or MRI scans of the chest, abdomen and pelvis. The same method used for assessment at baseline must be used at all subsequent time points.

Tumour assessment will include: if disease is measurable or non-measurable (at least one lesion must be measurable), date of assessment, imaging method used, site (breast, lung, liver, pleural effusion, local lymph nodes, distant lymph nodes, bone, brain or other site), longest diameter of each target lesion and sum of longest diameters for all target lesions.

Patient response to treatment will be assessed using RECIST v1.1 criteria. Tumour size, Progression Free Survival and Objective Response Rate will all be determined.

Tumour Size, will be assessed at baseline and on scans at subsequent time points and recorded as the sum of the longest diameters of the target lesions.

Progression Free Survival (PFS) is defined by the progression criteria of RECIST.

Objective Response Rate (ORR) is defined as the percentage of patients who have at least one visit response of CR or PR prior to any evidence of progression (as defined by RECIST v1.1)

The RECIST v1.1 (January 2009) guidelines for measurable, non-measurable, target and non-target lesions and the objective tumour response criteria (complete response, partial response, stable disease or progression of disease) are detailed in Appendix A

Baseline assessment should be performed no more than 28 days before the start of study treatment and ideally as close as possible to the start of study treatment; it should include all areas known for possible breast cancer metastases.

Subsequent tumour assessments will be conducted at week 6, week 12 (primary endpoint) and then every 8 weeks until objective disease progression or cessation of study treatment and finally at AZD4547 discontinuation visit. The window for each assessment is ± 1 week.

Duplicates will be made of all CT/MRI scans. These duplicate scans will be collected to enable an independent review of progression.

5.2.22. Assessment of Archival Tissue Oncology Biomarkers (Phase IIa only)

An archival tissue sample (either from the diagnostic tumour or a metastatic site) in the form of formalin fixed paraffin embedded (FFPE) tumour block will be collected from each patient. If it is not possible to obtain the entire tumour block, 10-20 slides of unstained 5 micron sections may be provided instead.

Provision of a tumour sample is mandatory. If an archival sample is not available, a fresh tumour biopsy sample must be taken. Tumour samples will be tested for oncology biomarkers.

Further details on sample processing, handling and shipment are provided in the Laboratory Manual.

The following details will be collected: whether archival or fresh tumour, sample tissue type (primary or metastatic), whether representative tumour tissue is present, biopsy site, biopsy type, and histology sample ID.

Archival tumour blocks will be returned to source at the end of the study or earlier, upon request, if required.

5.3. Pharmacokinetics (Safety run-in only)

5.3.1. Collection of Pharmacokinetic Samples

Venous blood samples (2 x 2.7 mL) for determination of concentrations of AZD4547, anastrozole and letrozole in plasma will be taken at the times presented in Table 3 on both day 7 of the NSAI monotherapy and day 7 of cycle 1 of the AZD4547 + anastrozole or letrozole combination therapy. These plasma samples will also be analysed for phosphate and the data used with the AZD4547 PK data to investigate any PK / PD relationship. The date and time of collection of each sample will be recorded.

Table 3: Pharmacokinetic Sampling Schedule for Safety run-in

NSAI[1] monotherapy day 7 and cycle 1 day 7 (AZD4547 + NSAI[1])	
**Time relative to dose (hours)**	**Time window**
Pre-dose	N/a
0.5	±10min
1	±10min
2	±15min
3	±15min
4	±15min
5	±15min
6	±30min
8	±30min

[1] Either anastrozole or letrozole

The timing of the PK samples may be adjusted during the study, dependent on emerging data, in order to ensure appropriate characterisation of the plasma concentration-time profiles. The total number of samples and the total volume of blood taken from each patient will not exceed that presented in Table 4. Samples will be collected, labelled, stored and shipped as detailed in the Laboratory Manual.

5.3.2. Determination of drug concentration in PK samples

Samples for determination of AZD4547 concentrations in plasma will be analysed at PRA International, The Netherlands. Samples for determination of anastrozole and letrozole concentrations in plasma will be analysed at Covance, UK, using appropriate bioanalytical methods.

5.3.3. PK Parameter Derivation

The actual sampling times will be used in the parameter calculations and PK parameters will be derived using standard non-compartmental methods.

Where possible the following PK parameters will be reported:

Anastrozole

Last day of NSAI monotherapy: $C_{ss,max}$, $t_{max,ss}$, $C_{ss,min}$, $AUC_{(0-8)}$, $AUC_{(0-t)}$, t, AUC_{ss}, CL_{ss}/F, V_{ss}/F, metabolite:parent ratio

Cycle 1 Day 7: $C_{ss,max}$, $t_{max,ss}$, $C_{ss,min}$, $AUC_{(0-8)}$, $AUC_{(0-t)}$, t, AUC_{ss}, CL_{ss}/F, V_{ss}/F, metabolite:parent ratio, ratio of C_{max} Cycle 1 Day 7 / C_{max} last day of NSAI monotherapy, ratio of AUC_{ss} Cycle 1 Day 15 / AUC_{ss} Last day of NSAI monotherapy

Letrozole

Last day of NSAI monotherapy: $C_{ss,max}$, $t_{max,ss}$, $C_{ss,min}$, $AUC_{(0-8)}$, $AUC_{(0-t)}$, t, AUC_{ss}, CL_{ss}/F, V_{ss}/F, metabolite:parent ratio

Cycle 1 Day 7: $C_{ss,max}$, $t_{max,ss}$, $C_{ss,min}$, $AUC_{(0-8)}$, $AUC_{(0-t)}$, t, AUC_{ss}, CL_{ss}/F, V_{ss}/F, metabolite:parent ratio, ratio of C_{max} Cycle 1 Day 15 / C_{max} Last day of NSAI monotherapy, ratio of AUC_{ss} Cycle 1 Day 15 / AUC_{ss} Last day of NSAI monotherapy

AZD4547

Cycle 1 Day 7: $C_{ss,max}$, $t_{max,ss}$, $C_{ss,min}$, $AUC_{(0-8)}$, $AUC_{(0-12)}$, $AUC_{(0-t)}$, t, AUC_{ss}, CL_{ss}/F, V_{ss}/F

5.4. Pharmacodynamics

5.4.1. Saftey Run-In

Blood samples (3 x 5ml) will be collected to provide two samples of plasma and one sample of serum per time point, as indicated in Table 1. These will be analysed for a range of oncology biomarkers including FGF23 and FGF2, which may correlate with drug response. The date of collection and sample IDs will be recorded on the eCRF.

5.4.2. PIIa

Blood samples (2 x 5ml) will be collected to provide one sample of plasma and one sample of serum per time point, as indicated in Table 2. These will be analysed for a range of oncology biomarkers, which may correlate with drug response. The date of collection and sample IDs will be recorded on the eCRF.

5.5. Exploratory Research

5.5.1. Biomarker research

Where a patient agrees to take part in the exploratory research aspect of the study, biological samples e.g. archived and study-specific tumour samples will be collected and may be analysed for biomarkers to investigate possible relationships with disease status, efficacy of study drug and outcome. These results may be reported separately from the clinical study report (CSR).

5.5.1.1. Collection of Archival Tumour Tissue Samples (Safety run-in)

This part of the study is optional. Where patients consent to take part, an archival tissue sample (either from the diagnostic tumour or a metastatic site) in the form of formalin fixed paraffin embedded (FFPE) tumour block will be collected. If it is not possible to obtain the entire tumour block, 10-20 slides of unstained 5 micron sections may be provided instead.

The following details will be collected: tissue obtained (yes / no), sample tissue type (primary or metastatic), whether representative tumour tissue is present, biopsy site, biopsy type, histology sample ID, whether FGFR FISH assessment was undertaken, date of assessment, FGFR1 FISH score.

5.5.1.2. Exploratory Research on Archival Tumour Tissue Samples (Phase IIa)

This part of the study is optional. Where patients consent to take part, 5-10 slides of unstained 5 micron sections will be taken from the archival tumour sample for exploratory research.

If archival tumour tissue was not available for mandatory assessment of oncology biomarkers, and a fresh tumour biopsy was obtained for this purpose (see 5.2.22), then slides form this new FFPE block may be used instead.

Further details on sample processing, handling and shipment are provided in the Study Manual.

Archival tumour blocks will be returned to source at the end of the study or, upon request, earlier if required. These samples are classified as research samples and will be registered with the Imperial College Healthcare NHS Tissue Bank (ICHTB).

The following details will be collected: whether slides taken; date slides taken and histology sample ID.

5.5.1.3. Collection of Paired Tumour Biopsies

This part of the study is optional. Where patients consent to take part, tumour biopsies should be collected prior to initiation of treatment (i.e. once the patient has been confirmed to be eligible by the RADICAL study team) and on cycle 1 day 22 (within 18 hours of administration of previous AZD4547 dose). It is strongly encouraged that at least the first biopsy is taken.

The following details will be collected: date of biopsy collection, biopsy site, biopsy type and histology sample ID.

5.5.2. Circulating Tumour Specific DNA (Phase IIa only)

Where a patient agrees to take part in the optional ctDNA study, 10 ml of blood will be collected at each of the time-points indicated in Table 2 to provide a plasma sample for ctDNA analysis. The date of collection and sample ID will be recorded on the eCRF.

The results of this research will not form part of the CSR.

5.5.3. Pharmacogenetics

Where the patient agrees to take part in the optional pharmacogenetics study, a single blood sample (1 x 5ml) will be collected to provide a plasma sample for genetic analysis. This should be obtained immediately prior to starting study treatment. The date of collection and sample ID will be recorded on the eCRF.

The results of this research will not form part of the CSR.

5.6. Chain of Custody of Biological Samples

In all cases, patients will be consented for the collection and use of their biological samples and a full chain of custody will be maintained for all samples throughout their lifecycle.

The investigator at each site is responsible for maintaining a record of full traceability of biological samples collected from patients while these are in storage at the site, either until shipment or disposal.

Any person(s) responsible for temporarily holding samples, e.g. sub-contracted service provider keeps full traceability of samples from initial receipt of sample to further shipment or disposal (as appropriate).

Imperial College keeps overall oversight of the entire lifecycle through internal procedures and monitoring of study sites

Samples retained for further use will be registered with the Imperial College Healthcare NHS Tissue Bank (ICHTB).

5.7. Total Blood Volumes

The total volume of blood that will be drawn from each patient in this study is shown in Table 4 for the safety run-in and in Table 5 for phase IIa. The number of samples taken, and the volume required for analysis, may change during the course of the study as new data becomes available.

Table 4: Volume of Blood to be Drawn from each Patient during Safety run-in

	Screening, NSAI[c] monotherapy and Cycle 1			Cycle 2 (and each subsequent cycle[b])			Treatment discontinuation / 28 day Follow-up		
	Sample volume (mL)	Number of samples	Total volume (mL)	Sample volume (mL)	Number of samples	Total volume (mL)	Sample volume (mL)	Number of samples	Total volume (mL)
Clinical chemistry[a]	6	5	30	6	1	6	6	2	12
Haematology[a]	9	5	45	9	1	9	9	2	18
Pharmaco-kinetics	5.4	18	97.2	-	-	-	-	-	-
PD biomarkers	15	5	75	-	-	-	15	1	15
Pharmaco-genetics	5	1	5	-	-	-	-	-	-
**Total**			**252.2**			**15**			**45**

[a] exact volume of blood for clinical chemistry and haematology may vary depending on local practice
[b] total volume in subsequent cycles may vary, but volume in any one cycle will not exceed the volume required at Cycle 2
[c] Either anastrozole or letrozole

Table 5: Volume of Blood to be Drawn from each Patient During Phase IIa

	Screening and Cycle 1			Cycle 2			Cycle 3 (and each subsequent cycle[b])			Treatment discontinuation / 28 day Follow-up		
	Sample volume (mL)	Number of samples	Total volume (mL)	Sample volume (mL)	Number of samples	Total volume (mL)	Sample volume (mL)	Number of samples	Total volume (mL)	Sample volume (mL)	Number of samples	Total volume (mL)
Clinical chemistry [a]	6	5	30	6	2	12	6	1	6	6	2	12
Haematology [a]	9	5	45	9	2	18	9	1	9	9	2	18
PD biomarkers	10	4	40	10	1	10	10	1	10	10	1	10
Pharmaco-genetics	5	1	5	-	-	-	-	-	-	-	-	-
ctDNA analysis	10	5	50	10	2	20	10	1	10	10	2	20
**Total**			**170**			**60**			**35**			**60**

[a] exact volume of blood for clinical chemistry and haematology may vary depending on local practice
[b] total volume in cycle 3 and subsequent cycles may vary, but volume in any one cycle will not exceed the volume required at Cycle 2

6. STUDY TREATMENT

6.1. Non Investigational Medicinal Products

Anastrozole (1mg once daily, orally) and letrozole (2.5mg once daily, orally) will be prescribed according to usual practice and dispensed from either site specific hospital stock or via the General Practitioner. Compliance to these treatments will be recorded using Patient Diary cards and all data transferred to the eCRFs.

6.2. Investigational Medicinal Product

Safety run-in: AZD4547 is manufactured by Pharmaceutical Development R&D, AstraZeneca UK according to Good Manufacturing Practice.

The investigational product is provided as, 20mg white, film-coated tablets containing AZD4547.

Phase IIa: AZD4547 is manufactured by Pharmaceutical Development R&D, AstraZeneca UK and AstraZeneca R&D Mölndal, according to Good Manufacturing Practice.

The investigational product is provided as, 20mg beige; film-coated tablets containing AZD4547.

6.2.1. Supply, Packaging and Labelling

AZD4547 will be packaged, labelled and distributed to sites by Fisher Clinical Services.
Labels will be prepared in accordance with Good Manufacturing Practice Annexe 13 requirements and local regulatory guidelines.

AZD4547 will only be dispatched to sites after receipt of confirmation that the regulatory checklist is complete.

6.2.2. Storage and Dispensing

The investigational products must be stored in a secure area with access limited to the Investigator and authorised site staff. AZD4547 film-coated tablets should be stored below 30°C in the original pack until use. Maintenance of a temperature log (manual or automated) is required. For further information investigators should refer to the investigator brochure.

The investigational product should only be dispensed and administered as directed in the protocol, and only by site staff authorised to do so i.e. pharmacist / trials technician. Only subjects enrolled in the trial may receive investigational product. AZD4547 will be dispensed at the beginning of each cycle of combination treatment.

6.2.3. Dosage and Duration

AZD4547 will be administered orally twice daily in a tablet formulation. A cycle of treatment will be defined as 28 days: one week of AZD4547 twice daily in combination with either anastrozole or letrozole once daily, followed by one week of either anastrozole or letrozole once daily alone, and the intermittent schedule repeated once more to complete one cycle.

For procedures relating to dose modification, dosing interruption and restarting of AZD4547 for subjects in both the safety run-in and phase IIa study, see 6.2.8.

6.2.3.1. Safety run-in

Dosing will begin with AZD4547 at 80 mg, twice daily given on an intermittent schedule of one week on/one week off.

Patients will be enrolled to ensure a minimum of 3 and a maximum of 6 evaluable patients per cohort. Dose de-escalation will occur according to the following conditions:

If one patient experiences a DLT in a group of 3 or more evaluable patients, then the cohort will be expanded to include 6 evaluable patients. If no more than one DLT is observed in the complete cohort of 6 evaluable patients then, with SRC review, this dose will be taken forward for the phase IIa part of the study.

If 2 or more patients experience a DLT in a group of up to 6 patients, irrespective of the number of patients enrolled, the combination dose will be considered not tolerated and recruitment to the cohort will cease. Instead, a lower intermediary combination dose (de-escalation) may be considered in order to better define the combination MTD.

There will be no intra-patient dose escalations.

The dose for subsequent cohorts or a decision to stop recruitment to the safety run-in part of the study will be agreed by the Safety Review Committee (SRC) after review of the data from each cohort. A different dose level of AZD4547 may be selected for combination with anastrozole than letrozole.

6.2.3.2. Phase IIa

The dose of AZD4547 to be used in combination with anastrozole and letrozole in the phase IIa part of the study has been determined in the safety run-in, i.e. 80 mg, twice daily given on an intermittent schedule of one week on/one week off.

N.B. If 2 or more cases of severe toxicity (leading to permanent discontinuation of study drug) are observed in the first 6 patients, an alternative schedule of two weeks on / one week off will be considered, if emerging data from other AZD4547 studies suggest that this is a better tolerated schedule. A cycle of treatment will be defined as 28 days: e.g. Cycle 1, two weeks of AZD4547 twice daily in combination with either anastrozole or letrozole once daily, followed by one week of either anastrozole or letrozole once daily alone, then one week of AZD4547 twice daily in combination with either anastrozole or letrozole once daily to complete the cycle.

6.2.4. Definition of Dose-Limiting Toxicity for assessment of safety and tolerability of AZD4547 in the safety run-in study

A DLT is defined as any toxicity not attributable to the disease or disease-related processes under investigation, which occurs after the first dose of AZD4547 at start of cycle 1 and before the end of cycle 1 (the DLT assessment window) and includes:

1. Haematological toxicity = CTCAE grade 4 present for more than 4 days
2. Non-haematological toxicity ≥ CTCAE grade 3 including:

- Infection including febrile neutropenia (Grade 3 with temperature ≥38.5°C or Grade 4 with temperature ≥38°C)
- QTc prolongation (> 500 msec) or QTc increase >60 msec from baseline

3. Any other toxicity that is greater than that at baseline, is clinically significant and/or unacceptable, does not respond to supportive care and results in a disruption of dosing schedule of more than 14 days
4. Any event, including significant dose reductions or omissions, judged to be a DLT by the SRC

A DLT excludes:

1. Alopecia of any grade
2. Isolated laboratory changes of any grade without clinical sequelae or clinical significance

6.2.5. Definition of Severe Toxicity for Assessment of Safety and Tolerability of AZD4547 in the PIIa study

For the purposes of the PIIa study, severe toxicity is defined as indicated above (6.2.4), but occurring after the first dose of AZD4547 at start of cycle 1 until study treatment discontinuation.

6.2.6. Definition of Evaluable Patient

6.2.6.1. Safety run-in

The safety run-in analysis set (SRIS) will be used for decisions on dose de-escalation and definition of dose to take forward into the phase IIa part of the study. The SRIS includes any patient that has received AZD4547 <u>and</u> either:

has completed minimum safety evaluation requirements and has received at least 80% of the specified dose (both AZD4547 and anastrozole / letrozole) during the first 28 day cycle

or

has experienced a DLT during the first 28 day cycle

In the safety run-in, patients that are withdrawn from the study but are deemed evaluable will not be replaced. Any patient that is withdrawn and not evaluable will be replaced to ensure the minimum number of evaluable patients is achieved.

6.2.6.2. Phase IIa

The intention-to-treat analysis set (ITTS) will be used for the efficacy analysis. The ITTS includes all study patients irrespective of drug compliance to study medication.

6.2.7. Safety Review Committee

In the safety run-in, once there are at least 3 evaluable patients at a dose level the SRC will review and assess all available safety data from the cohort together with available PK and pharmacodynamic data to make a decision on the dose for the next cohort of patients. Any dose interruptions and reductions will be taken into account. If there are still other patients that are ongoing at the time of the review, the SRC may decide to defer their decision until these further patients become evaluable.

6.2.8. Dose Modifications for AZD4547

If a patient experiences a clinically significant and/or unacceptable toxicity including a DLT not attributable to the disease or disease-related processes under investigation, where the Investigator considers the AE of concern to be specifically associated with AZD4547, dosing with AZD4547 will

be interrupted or the dose reduced and supportive therapy administered as required (see Figure 4 and Table 6).

If the toxicity resolves or reverts to a clinically acceptable level (at least ≤ CTCAE grade 2) within 14 days of onset and the patient is showing clinical benefit, study medication may be restarted using the rules below for dose modifications (see Figure 4) and with discussion and agreement with the Sponsor Study Team as needed.

If the toxicity does not resolve to a clinically acceptable level (at least ≤ CTCAE grade 2) after 14 days, then the patient should have study medication permanently discontinued and observed until resolution of the toxicity.

If a patient experiences a doubling of phosphate from baseline or a corrected calcium:phosphate product > 4.5mmol/L then the patient may remain on study treatment but phosphate chelation therapy (non-calcium containing agent) must be initiated, and clinical chemistry monitored weekly until resolution of the parameter to below the intervention limit. Investigators must seek appropriate specialist medical consultation (renal or metabolic) to advise on the prescription and titration of phosphate chelation agents, and to raise the patients awareness of low phosphate diets.

If patients experience toxicities regarding the anterior aspect of the eye (dry eyes, punctuate keratopathy and keratitis) such events must be clinically managed to prevent secondary consequences e.g. secondary infections following corneal abrasions. Lubricating eye drops/replacement tears should be used; if there is any indication of extra eyelash growth or eyelashes rubbing on the cornea then these eyelashes should be removed. It is anticipated that patients will report any visual disturbances or discomfort relating to the eye in advance of any significant pathology such as ulceration occurring. The decision to continue on study treatment if mild corneal changes in the eye examination are observed will be left to the Investigator's discretion, since a patient may indicate a wish to tolerate minor discomfort if there is perceived clinical benefit from the therapy. A patient should also be permanently discontinued from AZD4547 if corneal ulceration occurs, and appropriate expert ophthalmologic consultation should be initiated.

RPED has been identified in clinical studies with AZD4547 (45 occurrences as of 04 June 2014).

The prognosis is generally good if there is no actual haemorrhage from the capillaries and no evidence of any fibrovascular growth in the sub-RPE space.

An ophthalmological assessment is required if there are any of the following at any time:

- Abnormalities in the Amsler grid test
- Changes in near vision acuity
- Blurred vision
- Distortion of central vision

Subsequent management should be according to the algorithm included in Figure 5 and Figure 6. Any patients with an optical coherence tomography (OCT) scan diagnostic of RPED should be managed according to the same algorithm.

Table 6: Dose Interventions

Starting Dose	X mg bd
Reduced dose -1	X/2 mg bd
Reduced dose -2	(Reduced dose -1)/2 mg bd

Figure 4: AZD4547 Dose Modifications for Toxicity

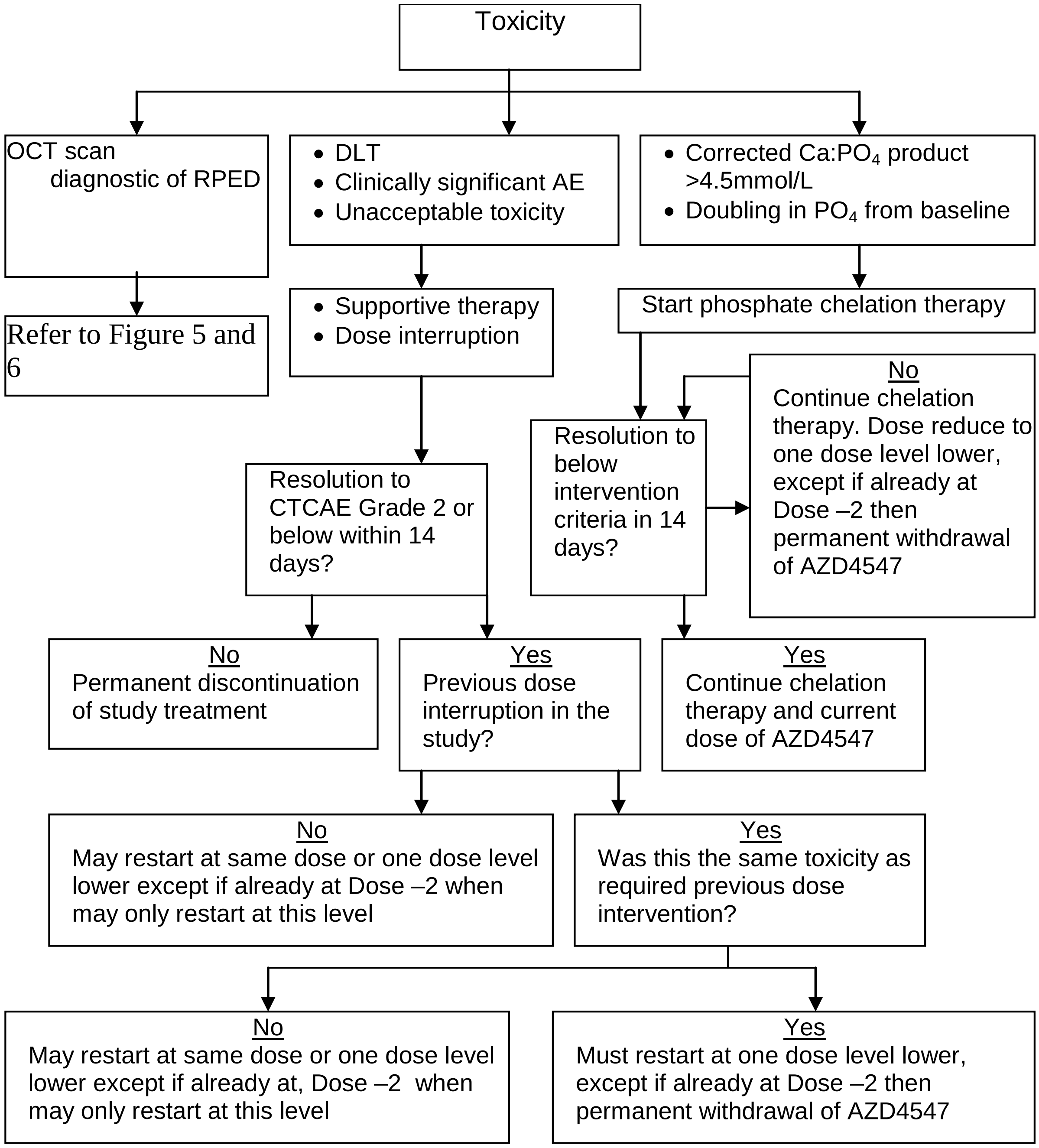

Figure 5: Management guidelines for patients with visual symptoms of ocular toxicity

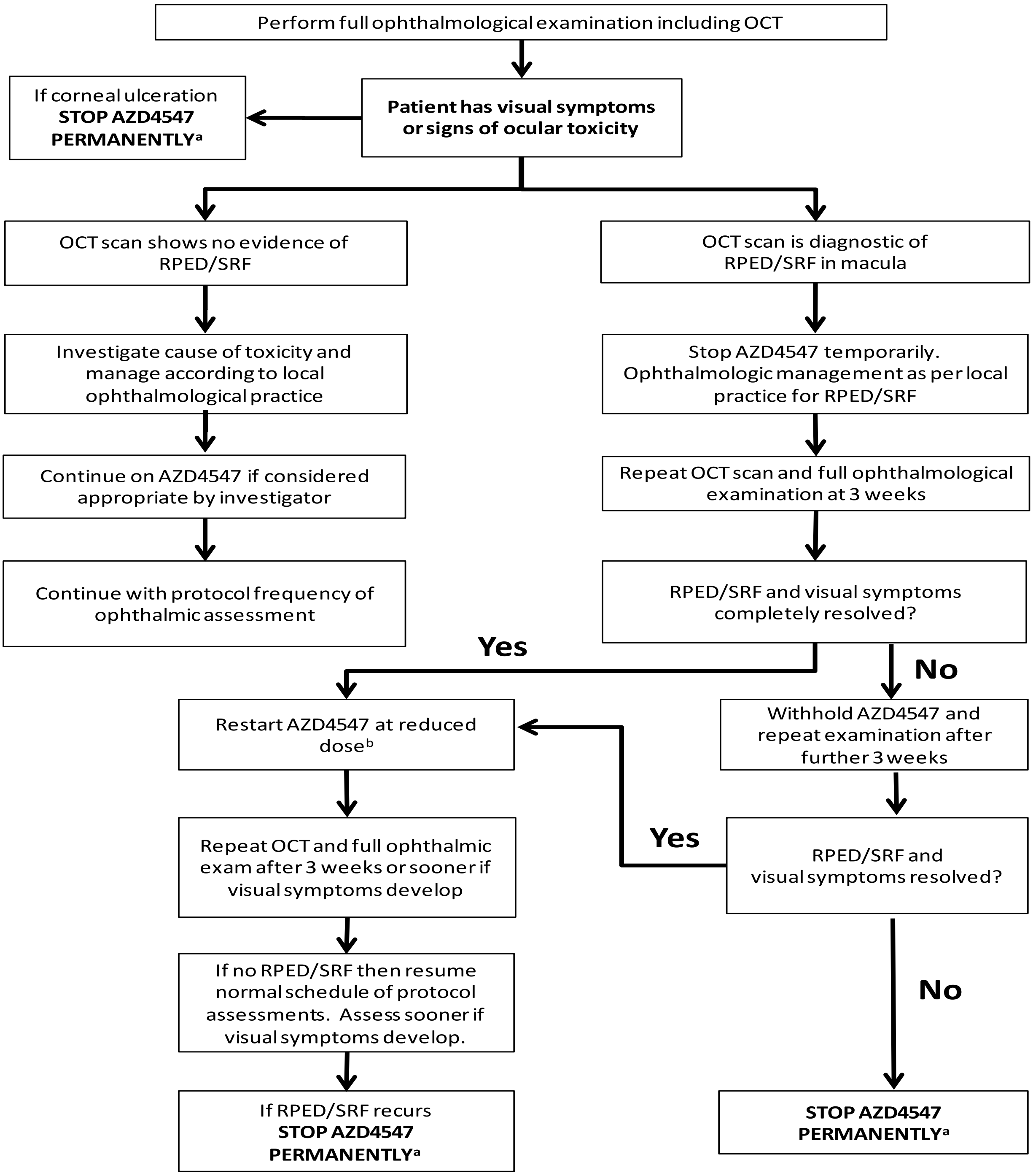

a: After permanent discontinuation of AZD4547 due to ocular toxicity, patients should be managed according to local clinical practice

b:Only 1 dose reduction allowed for management of RPED or SRF.

MedDRA Medical Dictionary for Regulatory Activities; OCT Optical-coherence-tomography; RPED or SRF This grouped term includes RPED (MedDRA preferred terms of detachment of retinal pigment epithelium and detachment of macular retinal pigment epithelium), MedDRA preferred term subretinal fluid, MedDRA preferred term serous detachment, MedDRA preferred term retinal detachment (MedDRA lower level term: serous retinal detachment); SRF Subretinal fluid

Figure 6: Toxicity management guidelines for patients with no visual symptoms of ocular toxicity

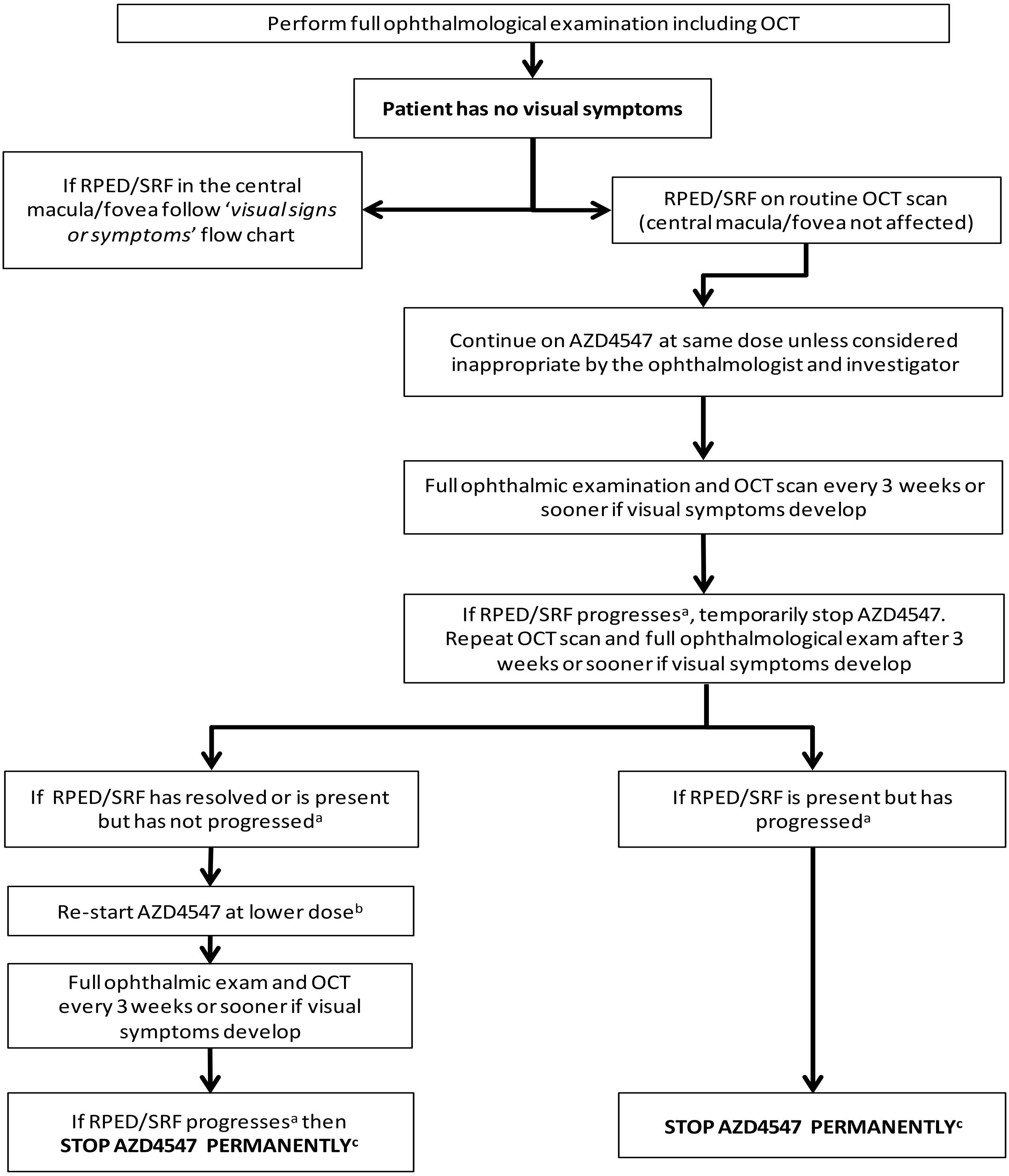

a: Progression of RPED or SRF is defined as development of visual symptoms, extension from para-macular to macula, or increase in the number of lesions.

b: Only 1 dose reduction allowed for management of RPED or SRF.

c: After permanent discontinuation of AZD4547 due to ocular toxicity, patients should be managed according to local clinical practice.

MedDRA Medical Dictionary for Regulatory Activities; OCT Optical-coherence-tomography; RPED or SRF This grouped term includes RPED (MedDRA preferred terms of detachment of retinal pigment epithelium and detachment of macular retinal pigment epithelium), MedDRA preferred term subretinal fluid, MedDRA preferred term serous detachment, MedDRA preferred term retinal detachment (MedDRA lower level term: serous retinal detachment); SRF Subretinal fluid.

6.2.9. Accountability

Patients should return all unused study medication and empty packaging to the Investigator.

A drug accountability record of the number of tablets dispensed to and returned by each subject will be maintained by the study pharmacist. This form is not part of the CRF but is maintained in the study TMF.

In accordance with local regulatory requirements, the investigator / designated site staff will document the amount of study drug received from Fisher Clinical Services, the amount (with date) dispensed to study subjects, the amount (with date) returned by study subjects and the amount destroyed locally.

Product accountability records will be maintained throughout the course of the study and filed with delivery documentation. Destruction will be documented as per local policy.

6.2.10. Compliance

The compliance of the patient will be assessed at each visit using the patient diary and the accuracy of the patient diaries will be cross checked with drug accountability records. At each visit, the cause of any missed doses should be discussed. Any AE(s) associated with missed doses must be recorded in the CRF. Subjects should be instructed on the importance of compliance to study treatments.

6.2.11. Drug Interactions/Precautions

6.2.11.1. Restrictions

The following restrictions apply while the patient is receiving study treatment and for the specified times before and after:

1. All patients should avoid concomitant use of drugs, herbal supplements and/or ingestion of foods known to modulate CYP3A4 or CYP2D6 enzyme activity and drugs that are known to be CYP3A4 substrates from the time they enter the screening period until 2 weeks after the last dose of study treatment. Please refer to Appendix B for further details.

2. Patients must not use oestrogen-containing agents such as hormone replacement therapy.

3. Patients with uncontrolled glaucoma or intra-ocular pressure ≥ 21mm Hg at screening should be referred for ophthalmological management and the condition controlled prior to first dose.

6.2.11.2. Concomitant Treatments

Information on any treatment in the 4 weeks prior to starting study treatment and all concomitant treatments given during the study, with reasons for the treatment, will be recorded in the CRF. If medically feasible, patients taking regular medication, with the exception of potent inhibitors or inducers of CYP3A4 or CYP2D6 or substrates of CYP3A4, should be maintained on it throughout the study period.

Patients should not be administered calcium-containing phosphate chelation agents, such as calcium acetate, for the management of hyperphosphataemia whilst receiving study treatment due to the increased risk of precipitating ectopic tissue mineralisation.

Other anticancer agents, investigational agents and radiotherapy should not be given while the patient is on study treatment although radiation for palliation at focal sites is permitted.

Patients may receive treatment with corticosteroids and/or bisphosphonates for the treatment of bone metastases.

Patients may take warfarin or a coumarin preparation but it is recommended that they should have their anticoagulation monitored carefully and dose adjusted accordingly.

Supportive care and other medications that are considered necessary for the patient's well-being may be given at the discretion of the Investigator.

6.2.12. Overdose of IMP

There is no known antidote to AZD4547. Investigators should be advised that any patient who receives a higher dose of AZD4547 than that intended should be monitored closely, managed with appropriate supportive care and followed up expectantly.

If an overdose of AZD4547 occurs in the course of the study, then Investigators or other site personnel should inform the Sponsor **within one day** i.e. immediately and no later than **the end of the next business day** of when he or she becomes aware of it.

For overdoses associated with a SAE, standard reporting timelines apply. For other overdoses, reporting should be done within 30 days.

For treatment of overdose with anastrozole / letrozole please refer to the local prescribing information. Overdose of anastrozole or letrozole with associated AEs / SAEs should be recorded in the relevant AE / SAE module of the eCRF and reported according to the standard timelines.

6.3. Permanent Discontinuation of Study Medication and Withdrawal from Study

6.3.1. Permanent Discontinuation of Study Medication

A patient may be permanently discontinued from study medication for the following reasons:

- Patient decision
- Significant adverse events or unacceptable toxicities
- Severe non-compliance to this protocol as judged by the Investigator
- Confirmed disease progression
- Allergic reaction to study medication
- If the investigator considers that a subject's health will be compromised due to adverse events or concomitant illnesses that develop after entering the study.

Date of permanent discontinuation and the reason will be recorded.

Once study medication is permanently discontinued it cannot be restarted.

6.3.2. Withdrawal from Study

Withdrawal from the study refers to discontinuation of both study medication and study participation; this can occur at any time according to the following reasons:

- Patient decision
- Lost to follow-up
- Death
- Investigator decision

If a patient dies whilst participating in the study a “Statement of Death” CRF must be completed. The following details will be collected: date of death, whether autopsy performed, whether death was related to the disease under investigation, primary cause of death, secondary cause of death, and any other details.

6.3.3. Procedures for Withdrawal from Study

If the patient is withdrawn from the study the date of withdrawal and the reason must be recorded. If possible, the investigator should arrange for the end of study assessments to be completed. Where the patient has withdrawn due to an AE, the investigator should follow the procedures in section 7.0.

7. PHARMACOVIGILANCE

7.1. Definition of an Adverse Event (AE)

An AE is any untoward medical occurrence (including deterioration of a pre-existing medical condition) in a patient or clinical trial subject administered a medicinal product, and which does not necessarily have a causal relationship with this treatment. An AE can therefore be any unfavourable and unintended sign including abnormal results of an investigation (e.g. laboratory finding, electrocardiogram), symptom(s) (e.g. nausea, chest pain), signs (e.g. tachycardia, enlarged liver) or disease temporally associated with the use of the trial medication.

7.1.1. Disease Progression

Disease progression is a worsening of a patient's condition attributable to the disease for which the study medication is being given. This may be an increase in severity of the disease or increase in the symptoms of the disease. The development of new, or progression of existing metastasis to the primary cancer under study should be considered as disease progression and not an AE. **Events that are unequivocally due to disease progression should not be reported as AEs during the study**.

7.1.2. New Cancers

The development of a new cancer should be regarded as an AE and reported accordingly. Generally, it will also meet at least one of the serious criteria.

7.2. Recording of Adverse Events

AEs will be collected throughout the study, from the point that the RADICAL team have confirmed patient eligibility, via e-mail (and the study entry eCRF has been completed by the RADICAL team) until the end of follow-up; they will be followed up according to local practice until the event has stabilised or resolved, or the Follow-up Visit, whichever is the sooner*. Serious Adverse Events (SAEs) will also be recorded throughout the study.

Any AEs which remain unresolved at the patient's last visit in the study should be followed up by the Investigator for as long as medically indicated, but without further recording in the CRF.*

***N.B. Any ophthalmic AEs will be followed up until event resolution or stabilisation (or until the trial closes if event is on-going at the end of trial) and should be recorded in the eCRF.**

If an Investigator learns of any SAEs, including death, at any time after a patient has completed the study and he/she considers there is a reasonable possibility that the event is related to AZD4547, the Investigator should notify the trials unit.

The following details will be collected in the CRF for each AE:

- AE description / diagnosis
- Date of onset and date of resolution
- CTCAE grade maximum intensity
- Seriousness
- Investigator causality rating against the study medication (yes or no)
- Action taken with regard to study medication
- Outcome

7.3. Severity of Adverse Events

Severity is a measure of intensity whereas seriousness is defined by the criteria in section 7.6. Severity will be assessed using the grading scales found in the National Cancer Institute CTCAE version 4.02 (September 2009) for all adverse events with an assigned CTCAE term. For those events without assigned CTCAE grades, the recommendation on page 1 of the CTCAE that converts mild, moderate and severe into CTCAE grades should be used. A copy of the CTCAE version 4.02 can be downloaded from the Cancer Therapy Evaluation Program website (http://ctep.cancer.gov).

7.4. Causality of Adverse Events

The Investigator will assess causal relationship between the investigational product and the combination treatment and each AE.

Unassessable: There is insufficient or incomplete evidence to make a clinical judgement of the causal relationship

Unrelated: No evidence of any causal relationship

Unlikely: There is little evidence to suggest there is a causal relationship (e.g. the event did not occur within a reasonable time after administration of the trial medication). There is another reasonable explanation for the event (e.g. the patient's clinical condition, other concomitant treatment).

Possible: There is some evidence to suggest a causal relationship (e.g. because the event occurs within a reasonable time after administration of the trial medication). However, the influence of other factors may have contributed to the event (e.g. the patient's clinical condition, other concomitant treatments).

Probable: There is evidence to suggest a causal relationship and the influence of other factors is unlikely.

Definite: There is clear evidence to suggest a causal relationship and other possible contributing factors can be ruled out.

7.5. Abnormal Laboratory Test Results

All clinically important abnormal laboratory test results occurring during the study will be recorded as adverse events. The clinically important abnormal laboratory tests will be repeated at appropriate intervals until they return either to baseline or to a level deemed acceptable by the investigator and the clinical monitor, or until a diagnosis that explains them is made.

7.6. Definitions of Serious Adverse Events (SAE)

An SAE is an AE occurring during any part of the study that meets one or more of the following criteria:

- Results in death;
- Is life-threatening*;
- Requires hospitalisation or prolongation of existing inpatient's hospitalisation**;
- Results in persistent or significant disability or incapacity;
- Is a congenital abnormality or birth defect;

* "Life-threatening" in the definition of "serious" refers to an event in which the subject was at risk of death at the time of the event; it does not refer to an event which hypothetically might have caused death if it were more severe.

** “Hospitalisation” means any unexpected admission to a hospital department. It does not usually apply to scheduled admissions that were planned before study inclusion or visits to casualty (without admission).

Medical judgement should be exercised in deciding whether an adverse event/reaction is serious in other situations. Important adverse events/reactions that are not immediately life-threatening, or do not result in death or hospitalisation but may jeopardise a subject, or may require intervention to prevent one of the other outcomes listed in the definition above should also be considered serious.

7.7. Reporting of SAEs

Rapid reporting, within 24 hours of the Principal Investigator or designee becoming aware of the event, of all SAEs occurring during the study or within 28 days following the completion of the study treatment by the subject, must be performed as detailed in the “SAE reporting instructions”. If the investigator becomes aware of safety information that appears to be drug related, involving a subject who participated in the study, even after an individual subject has completed the study, this should also be reported to the Sponsor.

The SAE should be reported electronically to the study team at the Imperial Clinical Trials Unit – Section on Cancer via the RADICAL database as detailed in the Pharmacovigilance study manual.

All SAEs will be reviewed by the Chief Investigator or designated representative to confirm relatedness and expectedness.

Following documented assessment by the CI, the completed SAE form will be sent by e-mail to AstraZeneca and the Sponsor by the study team at ICTU-Ca within the pre-specified timelines.

7.8. Definition of a Serious Adverse Reaction (SAR)

A SAR is defined as a SAE that is judged to be related to any dose of study drug administered to the subject.

7.9. Definition of Suspected Unexpected Serious Adverse Reaction (SUSAR)

Any SAR that is NOT consistent with the applicable product information as set out in the Investigator Brochure (IB) or Summary of Product Characteristics (SPC).

7.10. Reporting of SUSARs

SUSARs will be notified to the appropriate regulatory authority, the relevant Independent Ethics Committee (IEC) / Institutional review board (IRB), AstraZeneca, the Sponsor and the participating Principal Investigators by ICTU-Ca in accordance with regulatory requirements.

Follow up of patients who have experienced a SUSAR should continue until recovery is complete or the condition has stabilised.

7.11. Annual Reporting of Serious Adverse Reactions

Annual reports will be submitted to the MHRA and main REC by ICTU-Ca according to current requirements.

8. STATISTICAL ANALYSES

8.1. Sample Size and Power Considerations

For the primary analysis, a 95% two-sided confidence interval will be calculated for change in tumour size at 12 weeks. With 50 patients, assuming a standard deviation (SD) of 0.30* for the change in log transformed tumour size, the confidence interval for the mean on the log scale will extend 0.083 from the observed mean in either direction. On the natural scale, the 95% confidence interval for the observed geometric mean will extend 1.09 in either direction. This sample size should also be sufficient for the secondary and exploratory analyses to be performed.

*Our estimate of the SD is based on previously collected data, which showed that the standard deviation of logarithmically transformed tumour size at 12 weeks is 0.30. Since we would expect the SD of change in logarithmically transformed tumours size to be somewhat smaller than the SD of log transformed tumour size at 12 weeks, we feel that the above estimate of the width of the confidence interval is very conservative.

8.2. Data Analysis

8.2.1. Interim Analysis

An interim analysis will occur after 20 patients are recruited into the study (including patients in the safety run in phase) and have completed their 12-week follow up. Following recommendations from the Independent Data Monitoring Committee (IDMC) data will also be reviewed once 20 patients (including patients in the safety run in phase) have completed their 20-week follow up. Recruitment into the study will continue whilst the analysis is being carried out.

≥30% will be considered as the 12/20-week clinical benefit rate (stable disease, partial response, complete response) of interest and ≤5% as non-significant 12/20-week clinical benefit rate. With power of 85% and type I error of one-sided 0.05, the study will continue if 4 or more patients out of 20 show clinical benefit. If less than 4 patients show clinical benefit the study will only continue if a particular biomarker has been identified.

8.2.2. Preliminary Final Analysis

Since the study has follow up of efficacy and safety endpoints beyond 12 weeks, the preliminary final analysis may be conducted as soon as the database can be soft locked for the primary endpoint data accumulated up to 12 weeks after the last subject enters the study.

The analysis of primary endpoint, i.e. change in tumour size at 12 weeks (or progression if prior to week 12), will be performed on completed cases only. To assess the effect of early drop out (due to death or withdrawal before 12 week follow up scan or progression, for example) change in tumour size at 6 weeks will also be calculated and the results compared to the primary analysis. Subjects with only baseline measures will be excluded from the analysis.

The study data will be summarised using standard descriptive methods. Histograms and box-plots will be used to assess the distributional assumptions and to check for possible outliers. Mathematical transformations will be applied, where appropriate, in order to render variables normally distributed. Continuous variables that follow an approximately normal distribution will be summarised using the mean and standard deviation. Skewed continuous variables will be summarised using the median and inter-quartile range. Categorical variables (binary, ordered and multinomial) will be presented in terms of frequencies and percentages. Where possible, the relationship between the outcomes and other variables will be explored graphically, using scatter plots and box-plots prior to model fitting. Change in tumour size at 12 weeks for each patient will

be shown graphically in a “waterfall plot” with subgroups based on tumour response and objective response highlighted in different colours to identify possible patterns.

The comprehensive statistical analysis plan (SAP) will be finalised prior to preliminary final analysis.

8.2.3. Final Analysis

The final analysis will be conducted after the collection of the last data point for the last patient (i.e. study end). The final analysis will be an addendum of the preliminary final analysis for any data collected since the time of the previous analysis.

8.2.4. Missing, Unused and Spurious Data

Safety run-in

There will be no data imputation for missing data in the primary endpoint (DLTs). Procedures for accounting for missing, unused and spurious PK concentration-time data will be determined by the PK parameter derivation rules of the PK scientist. The PK parameter derivation rules will not be documented in the SAP.

Phase IIa

For the primary analysis, there will be no data imputation for missing data in the primary endpoint.

Imputation methods may be proposed for purposes of sensitivity analysis. Imputation methods for missing data in the primary endpoint and secondary endpoints will be fully documented in the SAP, if any.

8.2.5. Deviations from the Statistical Plan

Any deviation(s) from the final statistical plan in the final analysis will be described and justification given in the final report.

8.2.6. Efficacy Analysis

All patients will be included in the efficacy analysis.

To be considered ‘evaluable’ for the primary efficacy analysis, a patient must have a week 12 (± 1 week) tumour measurement or evidence of progression prior to week 12.

8.2.7. Primary Analysis

The primary endpoint of change in tumour size at week 12 (or progression if prior to week 12) will be assessed in all patients. The data cut-off for the primary endpoint will occur 12 weeks after the last subject has entered the study.

8.2.8. Secondary Analyses

Frequency tabulations of the tumour response RECIST criteria and 2-category ORR will be presented.

As median time to progression is expected to be 6 months and the recruitment period to be 24 months, the analysis of PFS will take place at 30 months (when each subject has been followed up to progression or for a minimum of 6 months). At this time we anticipate that 95% of subjects will have progressed on study treatment.

8.2.9. Safety Analysis

All patients who receive at least one dose of study treatment will be included in the safety analysis set. Safety data will not be formally analysed. Safety and tolerability data will be presented by treatment received.

9. REGULATORY, ETHICAL AND LEGAL ISSUES

9.1. Declaration of Helsinki

The investigator will ensure that this study is conducted in full conformity with the principles of the 1964 Declaration of Helsinki and any subsequent revisions.

9.2. Good Clinical Practice

The study will be conducted in accordance with the guidelines laid down by the International Conference on Harmonisation for Good Clinical Practice (ICH GCP E6 guidelines).

9.3. Independent Ethics Committee/Institutional Review Board Approval

9.3.1. Initial Approval

Prior to the shipment of IMP and the enrolment of subjects, the IEC/IRB must provide written approval of: the conduct of the study at named sites, the protocol and any amendments, the Subject Information Sheet and Consent Form, any other written information that will be provided to the subjects, any advertisements that will be used and details of any subject compensation.

9.3.2. Approval of Amendments

Proposed amendments to the protocol and aforementioned documents must be submitted to the IEC/IRB for approval. Amendments requiring IEC/IRB approval may be implemented only after a copy of the IEC/IRB's approval letter has been obtained.

Amendments that are intended to eliminate an apparent immediate hazard to subjects may be implemented prior to receiving Sponsor or IEC/IRB approval. However, in this case, approval must be obtained as soon as possible after implementation.

9.3.3. Annual Safety Reports and End of Trial Notification

The IEC/IRB will be sent annual safety updates in order to facilitate their continuing review of the study (reference. ICH GCP E6 Section 3.1.4) and will also be informed about the end of the trial, within the required timelines.

9.4. Regulatory Authority Approval

The study will be performed in compliance with the regulatory requirements of the United Kingdom. Clinical Trial Authorisation from the appropriate Regulatory Authority must be sought/obtained prior to the start of the study. In addition, the Regulatory Authority must approve amendments (as instructed by the Sponsor), receive SUSAR reports and annual safety updates, and be notified of the end of the trial.

9.5. Insurance

The Sponsor has civil liability insurance, which covers this study in the United Kingdom.

9.6. Informed Consent

The Principal Investigator at each site will:

- Ensure that each patient is given full and adequate oral and written information about the study including the background, purpose and risks/benefits of participation
- Ensure that each patient is notified that they are free to withdraw from the study at any time

- Ensure that each patient is given the opportunity to ask questions and allowed sufficient time to read and understand the information sheet
- Ensure each patient provides signed, dated informed consent before undergoing any study specific procedure
- Ensure the original copy of the signed, dated Informed Consent Form is stored in the patient's medical records and a copy is also filed in the Investigator site file
- Ensure that each patient receives a copy of the signed, dated Informed Consent Form

9.7. Contact with General Practitioner

It is the investigator's responsibility to inform the subject's General Practitioner (where applicable) by letter that the subject is taking part in the study provided the subject agrees to this, and information to this effect is included in the Subject Information Sheet and Informed Consent. A copy of the letter should be filed in the Investigator Site File.

9.8. Subject Confidentiality

The investigator must ensure that the subject's privacy is maintained. On the CRF or other documents submitted to the Sponsors, subjects will be identified by a trial ID number only. Documents that are not submitted to the Sponsor (e.g. signed informed consent form) should be kept in a strictly confidential file by the investigator.

The investigator shall permit direct access to subjects' records and source document for the purposes of monitoring, auditing, or inspection by the Sponsor, authorised representatives of the Sponsor, Regulatory Authorities and IECs / IRBs.

9.9. Data Protection

Precautions will be taken to ensure that patient confidentiality is preserved at all times. The Patient Consent form will identify those individuals who will require access to patient data and identifiable details and obtain appropriate permission from the consenting patient.

9.10. End of Trial

The end of the trial is defined as collection of the last data point for the last patient.

9.11. Study Documentation and Data Storage

The investigator must retain essential documents until notified by the Sponsor (Imperial College London), and at least for ten years after study completion, as per Imperial College London policy. Subject files and other source data (including copies of protocols, CRFs, original reports of test results, IMP dispensing logs, correspondence, records of informed consent, and other documents pertaining to the conduct of the study) must be kept for the maximum period of time permitted by the institution. Documents should be stored in such a way that they can be accessed/data retrieved at a later date. Consideration should be given to security and environmental risks.

No study document will be destroyed without prior written agreement between the Sponsor and the investigator. Should the investigator wish to assign the study records to another party or move them to another location, written agreement must be obtained from the Sponsor.

10. DATA AND STUDY MANAGEMENT

10.1. Source Data

All original records and certified copies of original records of clinical findings, observations, or other activities necessary for the reconstruction and evaluation of the trial are classified as source data. Source data are contained in source documents; these are defined as: original documents, data, and records e.g., hospital records, clinical and office charts, laboratory notes, memoranda, subjects' diaries or evaluation checklists, pharmacy dispensing records, recorded data from automated instruments, copies or transcriptions certified after verification as being accurate copies, microfiches, photographic negatives, microfilm or magnetic media, x-rays, subject files, and records kept at the pharmacy, at the laboratories and at medico-technical departments involved in the clinical trial.

10.2. Language

CRFs will be in English. Generic names for concomitant medications should be recorded in the CRF wherever possible. All written material to be used by subjects must use vocabulary that is clearly understood, and be in the language appropriate for the study site.

10.3. Data Collection

In compliance with Good Clinical Practice (GCP), the medical records/medical notes should be clearly marked and allow easy identification of a patient's participation in the clinical trial

The Investigator (or delegated member of the site study team) must record all data relating to protocol procedures, IMP administration, laboratory data, safety data and efficacy data into the trial InForm electronic data collection (EDC) system.

10.4. Electronic Recording of data

Full details for procedures for completion of eCRFs will be provided in the study manual.

10.5. Data Management

Data management will be performed by the Imperial Clinical Trials Unit – Section on Cancer using the InForm electronic data capture (EDC) and management system. The system allows for real time oversight of trial activity including adverse event reporting, rapid data validation and data aggregation.

AE data will be coded using the Medical Dictionary for Regulatory Activities (MedDRA) system organ class and preferred term, and CTCAE grade.

Data queries will be raised for inconsistent, impossible or missing data. All entries to the study database will be available in an audit trial.

10.6. Study Management Structure

10.6.1. Trial Steering Committee

The Trial Steering Committee (TSC) convened for the safety run-in part of the study, will continue their role in the phase IIa part of the study. The TSC includes: an independent Chair; two independent clinicians; a patient representative; the Chief Investigator and Trial Coordinator. The role of the TSC will be to provide overall supervision of the trial including monitoring progress, adherence to the protocol and patient safety. It will also consider new information relevant to the

research question as it becomes available and as necessary, advise the TMG on operational issues.

Where possible, membership will also include a lay/consumer representative

10.6.2. Trial Management Group

The Trial Management Group (TMG) convened for the safety run-in part of the study, will continue their role in the phase IIa part of the study. The TMG includes: the Chief Investigator, co-investigators and identified key collaborators, the trial statistician and trial co-ordinator. Principle Investigators and key study personnel may be invited to join the TMG as appropriate to ensure representation from a range of sites and professional groups.

Notwithstanding the legal obligations of the Sponsor and Chief Investigator, the TMG will have operational responsibility for the day to day conduct of the trial.

10.6.3. Safety Review Committee (Safety run-in only)

The Safety Review Committee (SRC) will consist of the Chief Investigator, the Trial Coordinator and local investigators or delegates from each actively recruiting site. An AstraZeneca Medical Science Director and a Medical Advisor from Cancer Research UK's Drug Development Office will also be invited to attend. The ICTU-Ca Senior Trials Manager and Study Statistician may attend as appropriate. The committee will evaluate safety data acquired during the safety run-in part of the study only, and make recommendations on dose de-escalation / modification decisions in the safety run-in phase and confirm the dose to take forward to the phase IIa part of the study.

10.6.4. Independent Data Monitoring Committee (Phase IIa only)

An Independent Data Monitoring Committee (IDMC) will be convened to monitor data collected during the phase IIa part of the study only, and make recommendations to the TSC on whether there are any ethical or safety reasons as to why the trial should not continue. It will consist of an independent Chair, an independent statistician and an independent clinician.

10.7. Monitoring

The study will be monitored periodically by monitors in the UK to assess the progress of the study, verify adherence to the protocol, ICH GCP E6 guidelines and other national/international requirements and to review the completeness, accuracy and consistency of the data.

Monitoring procedures and requirements will be documented in a Monitoring Plan. Monitoring will be proportionate to the objective, purpose, design, size, complexity, blinding, endpoints and risks associated with the clinical trial. The appropriate level and nature of monitoring required for the clinical trial will be assessed by undertaking a formal risk assessment analysis of the study.

10.8. Quality Control and Quality Assurance

Quality Control will be performed according to ICTU internal procedures. The study may be audited by a Quality Assurance representative of the Sponsor. All necessary data and documents will be made available for inspection.

10.9. Disclosure of Data and Publication

Information concerning the study, patent applications, processes, scientific data or other pertinent information is confidential and remains the property of the Sponsor. The investigator may use this information for the purposes of the study only.

It is understood by the investigator that the Sponsor will use information developed in this clinical study in connection with the development of the IMP and, therefore, may disclose it as required to other clinical investigators and to Regulatory Authorities. In order to allow the use of the information derived from this clinical study, the investigator understands that he/she has an obligation to provide complete test results and all data developed during this study to the Sponsor.

Verbal or written discussion of results prior to study completion and full reporting, should only be undertaken with written consent from the Sponsor.

Therefore all information obtained as a result of the study will be regarded as CONFIDENTIAL, at least until appropriate analysis and review by the investigator(s) is completed.

Investigators may only present data separately to the total data available, with the permission of the TMG, and not less than 6 months after the publication of the main results.

AstraZeneca has the right to review all abstracts, papers or other research communications prior to their submission to journals, meetings or conferences.

11. REFERENCES

1. Fisher B, Anderson S, Tan-Chiu E, et al: Tamoxifen and chemotherapy for axillary node-negative, estrogen receptor-negative breast cancer: findings from National Surgical Adjuvant Breast and Bowel Project B-23. J Clin Oncol 19:931-42, 2001

2. NCCN: National Comprehensive Cancer Network Clinical Practice Guidelines in Oncology (NCCN Guidelines™): Breast Cancer V2. 2010. 2010

3. Cardoso F, Castiglione M: Locally recurrent or metastatic breast cancer: ESMO clinical recommendations for diagnosis, treatment and follow-up. Ann Oncol 20 Suppl 4:15-8, 2009

4. Turner N, Pearson A, Sharpe R, et al: FGFR1 amplification drives endocrine therapy resistance and is a therapeutic target in breast cancer. Cancer Res 70:2085-94, 2010

5. Song S, Wientjes MG, Gan Y, et al: Fibroblast growth factors: an epigenetic mechanism of broad spectrum resistance to anticancer drugs. Proc Natl Acad Sci USA 97:8658-8663, 2000

6. Pardo OE, Wellbrock C, Khanzada UK, et al: FGF-2 protects small cell lung cancer cells from apoptosis through a complex involving PKCepsilon, B-Raf and S6K2. Embo J 25:3078-88, 2006

7. Pardo OE, Arcaro A, Salerno G, et al: Fibroblast growth factor-2 induces translational regulation of Bcl-XL and Bcl-2 via a MEK-dependent pathway: correlation with resistance to etoposide-induced apoptosis. J Biol Chem 277:12040-6, 2002

8. Pardo OE, Lesay A, Arcaro A, et al: Fibroblast growth factor 2-mediated translational control of IAPs blocks mitochondrial release of Smac/DIABLO and apoptosis in small cell lung cancer cells. Mol Cell Biol 23:7600-10, 2003

9. Aguilar H, Sole X, Bonifaci N, et al: Biological reprogramming in acquired resistance to endocrine therapy of breast cancer. Oncogene, 2010

10. Lonning P, Pfister C, Martoni A, et al: Pharmacokinetics of third-generation aromatase inhibitors. Semin Oncol 30:23-32, 2003

11. Pardo OE, Latigo J, Jeffery RE, et al: The fibroblast growth factor receptor inhibitor PD173074 blocks small cell lung cancer growth in vitro and in vivo. Cancer Res 69:8645-51, 2009

12. Xian W, Pappas L, Pandya D, et al: Fibroblast growth factor receptor 1-transformed mammary epithelial cells are dependent on RSK activity for growth and survival. Cancer Res 69:2244-51, 2009

13. Welm BE, Freeman KW, Chen M, et al: Inducible dimerization of FGFR1: development of a mouse model to analyze progressive transformation of the mammary gland. J Cell Biol 157:703-14, 2002

14. Reis-Filho JS, Simpson PT, Turner NC, et al: FGFR1 emerges as a potential therapeutic target for lobular breast carcinomas. Clin Cancer Res 12:6652-62, 2006

15. Baselga J, Campone M, Piccart M, Burris III HA, Rugo HS, Sahmoud T et al. Everolimus in postmenopausal hormone-receptor-positive advanced breast cancer. N Engl J Med 366:520-529, 2012

12. SIGNATURE PAGES

SIGNATURE PAGE 1 (Chief Investigator)

The signature below constitutes approval of this protocol by the signatory.

I agree to the terms of this study protocol. I will conduct the study according to all stipulations of the protocol including all statements regarding confidentiality, and according to the principles of Good Clinical Practice (GCP) and local regulations.

Study Title: A single arm phase IIa study (with combination safety run-in) to assess the safety and efficacy of AZD4547 in combination with either anastrozole or letrozole in ER positive breast cancer patients who have progressed on treatment with anastrozole or letrozole - **RADICAL**

Protocol Number: C/23/2011

Signed: ____

Michael J Seckl
Professor of Molecular Cancer Medicine

Date: ______________________

SIGNATURE PAGE 2 (Sponsor)

The signature below constitutes approval of this protocol by the signatory.

Study Title: A single arm phase IIa study (with combination safety run-in) to assess the safety and efficacy of AZD4547 in combination with either anastrozole or letrozole in ER positive breast cancer patients who have progressed on treatment with anastrozole or letrozole - **RADICAL**

Protocol Number: C/23/2011

Signed: ______________________________

Gary Roper
Head of Regulatory Compliance
Imperial College London

Date: ______________

SIGNATURE PAGE 3 (STUDY STATISTICIAN)

The signature below constitutes approval of this protocol by the signatory.

Study Title: A single arm phase IIa study (with combination safety run-in) to assess the safety and efficacy of AZD4547 in combination with either anastrozole or letrozole in ER positive breast cancer patients who have progressed on treatment with anastrozole or letrozole - **RADICAL**

Protocol Number: C/23/2011

Signed: ______________________________

Xinxue Liu
Study Statistician
Imperial College London

Date: ____________________

SIGNATURE PAGE 4 (INVESTIGATOR)

The signature of the below constitutes agreement of this protocol by the signatory and provides the necessary assurance that this study will be conducted at his/her investigational site according to all stipulations of the protocol including all statements regarding confidentiality.

Study Title: A single arm phase IIa study (with combination safety run-in) to assess the safety and efficacy of AZD4547 in combination with either anastrozole or letrozole in ER positive breast cancer patients who have progressed on treatment with anastrozole or letrozole - **RADICAL**

Protocol Number: C/23/2011

Address of Institution: ____

____

____

Signed: ____

Print Name and Title: ____

Date: ______________________

13. APPENDICES

Appendix A: Guidelines for Evaluation of Objective Tumour Response Using RECIST 1.1 (Response Evaluation Criteria in Solid Tumours)

(ICTU-Cancer guidance document v2.0 dated 17 November2014)

Response and progression will be evaluated in this study using the new international criteria proposed by the revised Response Evaluation Criteria in Solid Tumours (RECIST) guideline (version 1.1) [*Eur J Ca* 45:228-247, 2009]. Changes in the largest diameter (unidimensional measurement) of the tumour lesions and the shortest diameter in the case of malignant lymph nodes are used in the RECIST criteria.

Definition of Disease Parameters

<u>Measurable disease</u> Must be accurately measured in a least one dimension (longest diameter in the plane of measurement is to be recorded) with a minimum size of:

- 10mm by CT scan (CT scan slice thickness no greater than 5mm; when CT scans have slice thickness >5mm, the minimum size should be twice the slice thickness).
- 10mm caliper measurement by clinical exam (lesions which cannot be accurately measured with calipers should be recorded as non-measureable).
- 20mm by chest X-ray.

Note: Tumour lesions situated in a previously irradiated area or in an area subjected to other loco-regional therapy are usually not considered measureable unless there has been demonstrated progression in the lesion. Study protocols should detail the conditions under which such lesions would be considered measurable.

<u>Malignant lymph nodes</u> Criteria for lymph nodes given as ≥15mm short axis for target lesions and 10mm to <15mm for non-target lesions. Nodes under 10mm to be considered non-pathological.

<u>Non-measurable disease</u> All other lesions, including small lesions (longest diameter <10 mm or pathological lymph nodes with 10 to <15 mm short axis), as well as truly non-measurable lesions. Lesions considered truly non-measurable include; leptomeningeal disease, ascites, pleural/pericardial effusions, inflammatory breast disease, lymphangitic involvement of skin or lung, abdominal masses/abdominal organomegaly identified by physical exam that is not measureable by reproducible imaging techniques.

Note: Lytic bone lesions or mixed lytic-blastic lesions with identifiable soft tissue components that can be evaluated by cross-sectional imaging techniques such as CT or MRI can be considered measurable if the soft tissue component meets the definition of measurability described above.

'Cystic lesions' thought to represent cystic metastases can be considered measurable if they meet the definition of measurability described above. However, if non-cystic lesions are present in the same patient, these are preferred for selection as target lesions.

<u>Target lesions</u> All measurable lesions up to a maximum of 2 lesions per organ and 5 lesions in total, representative of all involved organs, should be identified as target lesions and recorded and measured at baseline. Target lesions should be selected on the basis of their size (lesions with the longest diameter) and be representative of all involved organs, as well as their suitability for reproducible repeated measurements. All measurements should be recorded in metric notation

using calipers if clinically assessed. A sum of the diameters (longest for non-nodal lesions, short axis for nodal lesions) for all target lesions will be calculated and reported as the baseline sum diameters, which will be used as reference to further characterize any objective tumour regression in the measurable dimension of the disease. If lymph nodes are to be included in the sum, only the short axis will contribute.

Non-target lesions All lesions (or sites of disease) not identified as target lesions, including pathological lymph nodes and all non-measurable lesions, should be identified as non-target lesions and be recorded at baseline. Measurements of these lesions are not required and they should be followed as 'present', 'absent' or in rare cases, 'unequivocal progression'.

Methods of Measurement

All measurements should be taken and recorded in metric notation using a ruler or calipers. All baseline evaluations should be performed as closely as possible to the beginning of treatment and never more than 4 weeks before the beginning of the treatment.

The same method of assessment and the same technique should be used to characterize each identified and reported lesion at baseline and during follow-up.

CT/MRI: CT is the best currently available and reproducible method to measure lesions selected for response assessment. MRI is also acceptable in certain situations (e.g. for body scans but not for lung).

Chest x-ray Lesions on chest x-ray may be considered measurable lesions if they are clearly defined and surrounded by aerated lung. However, CT is preferable.

Clinical lesions Clinical lesions will only be considered measurable when they are superficial and ≥10mm in diameter as assessed using calipers. For the case of skin lesions, documentation by colour photography, including a ruler to estimate the size of the lesion, is recommended.

Ultrasound (US) should not be used to measure tumour lesions.

Tumour markers Tumour markers alone cannot be used to assess response. If markers are initially above the upper normal limit, they must normalize for a patient to be considered in complete clinical response. Specific guidelines for both CA-125 response (in recurrent ovarian cancer) and PSA response (in recurrent prostate cancer) have been published [*JNCI* 96:487-488, 2004; *J Clin Oncol* 17, 3461-3467, 1999; *J Clin Oncol* 26:1148-1159, 2008]. In addition, the Gynecologic Cancer Intergroup has developed CA-125 progression criteria which are to be integrated with objective tumour assessment for use in first-line trials in ovarian cancer [*JNCI* 92:1534-1535, 2000].

Cytology, Histology Can be used in rare cases (e.g. for evaluation of residual masses to differentiate between Partial Response and Complete Response or evaluation of new or enlarging effusions to differentiate between Progressive Disease and Response/Stable Disease).

Endoscopy, Laparoscopy Use of endoscopy and laparoscopy is not advised. However, they can be used to confirm complete pathological response.

New Lesions

It is sometimes reasonable to incorporate the use of FDG-PET scanning to complement CT in assessment of progression (particularly possible 'new' disease). New lesions on the basis of FDG-PET imaging can be identified according to the following algorithm:

Negative FDG-PET at baseline, with a positive FDG-PET at follow-up is PD based on a new lesion.

No FDG-PET at baseline and a positive FDG-PET at follow up:

- If the positive FDG-PET at follow-up corresponds to a new site of disease confirmed by CT, this is PD.
- If the positive FDG-PET at follow-up is not confirmed as a new site of disease on CT, additional follow-up CT scans are needed to determine if there is truly progression occurring at that site (if so, the date of PD will be the date of the initial abnormal FDG-PET scan).
- If the positive FDG-PET at follow-up corresponds to a pre-existing site of disease on CT that is not progressing on the basis of the anatomic images, this is no PD.

Response Criteria

Evaluation of Target Lesions

Complete Response (CR): Disappearance of all target lesions. Any pathological lymph nodes (whether target or non-target) must have reduction in short axis to <10 mm.

Partial Response (PR): At least a 30% decrease in the sum of the diameters of target lesions, taking as reference the baseline sum diameters

Progressive Disease (PD): At least a 20% increase in the sum of the diameters of target lesions, taking as reference the smallest sum on study (this may include the baseline sum). The sum must also demonstrate an absolute increase of at least 5mm.

Stable Disease (SD): Neither sufficient shrinkage to qualify for PR nor sufficient increase to qualify for PD.

Evaluation of Non-Target Lesions

Complete Response (CR): Disappearance of all non-target lesions and normalization of tumour marker levels. All lymph nodes must be non-pathological in size (<10 mm short axis)

Non-CR/Non-PD: Persistence of one or more non-target lesion(s) and/or maintenance of tumour marker level above the normal limits

Progressive Disease (PD): Unequivocal progression of existing non-target lesions.

- When patient has measurable disease – to achieve 'unequivocal progression' on the basis of the non-target disease, there must be an overall level of substantial worsening in non-

target disease such that, even in presence of SD or PR in target disease, the overall tumour burden has increased sufficiently to merit discontinuation of therapy. A modest 'increase' in the size of one or more non-target lesions is usually not sufficient to qualify for unequivocal progression status.

- When patient has none-measurable disease – there is no measurable disease assessment to factor into the interpretation of an increase in non-measurable disease burden. Because worsening in non-target disease cannot be easily quantified, a useful test that can be applied is to consider if the increase in overall disease burden based on change in non-measurable disease is comparable in magnitude to the increase that would be required to declare PD for measurable disease. Examples include an increase in a pleural effusion from 'trace' to 'large' or an increase in lymphangitic disease from localised to widespread.

Evaluation of Best Overall Response

Summary of overall response status calculation at each time point, for patients who have measurable disease at baseline.

Target Lesions	Non-Target Lesions	New Lesions	Overall Response	Best Overall Response when Confirmation is Required*
CR	CR	No	CR	≥4 wks. Confirmation**
CR	Non-CR/Non-PD	No	PR	≥4 wks. Confirmation**
CR	Not evaluated	No	PR	
PR	Non-CR/Non-PD/not evaluated	No	PR	
SD	Non-CR/Non-PD/not evaluated	No	SD	documented at least once ≥4 wks. from baseline**
Not all evaluated	Non-PD	No	Not evaluated	
PD	Any	Yes or No	PD	no prior SD, PR or CR
Any	PD***	Yes or No	PD	
Any	Any	Yes	PD	

* See RECIST 1.1 manuscript for further details on what is evidence of a new lesion.

** Only for non-randomized trials with response as primary endpoint.

*** In exceptional circumstances, unequivocal progression in non-target lesions may be accepted as disease progression.

Note: Patients with a global deterioration of health status requiring discontinuation of treatment without objective evidence of disease progression at that time should be reported as "*symptomatic deterioration.*" Every effort should be made to document the objective progression even after discontinuation of treatment.

For Patients with Non-Measurable Disease (i.e., Non-Target Disease)

Non-Target Lesions	New Lesions	Overall Response
CR	No	CR
Non-CR/non-PD	No	Non-CR/non-PD*
Not all evaluated	No	not evaluated
Unequivocal PD	Yes or No	PD
Any	Yes	PD
* 'Non-CR/non-PD' is preferred over 'stable disease' for non-target disease since SD is increasingly used as an endpoint for assessment of efficacy in some trials so to assign this category when no lesions can be measured is not advised		

Duration of Response

Duration of overall response: The duration of overall response is measured from the time measurement criteria are met for CR/PR (whichever status is recorded first) until the first date that recurrence or PD is objectively documented, (taking as reference for PD the smallest measurements recorded on study).

Duration of stable disease: SD is measured from the start of the treatment (in randomised trials, from the date of randomization) until the criteria for disease progression are met, taking as reference the smallest sum on study (if baseline sum is the smallest, this is the reference for calculation of PD).The clinical relevance of the duration of SD varies for different studies and diseases. This time interval should take into account the expected clinical benefit that such a status may bring to the population under study. If the proportion of patients achieving stable disease for a minimum period of time is an endpoint of importance in a particular trial, the protocol should specify the minimal time interval required between two measurements for determination of stable disease.

Response Review

For trials where the objective response (CR and PR) is the primary endpoint it is recommended that all responses be reviewed by an expert(s) independent of the study. Simultaneous review of the patients' files and radiological images is the best approach.

Appendix B:

Guidance on Potential Interactions with Concomitant Medications (dated 9th July 2010)*

***The appendix should not be taken as a definitive list. All conmeds should be checked locally for Drug Drug Interactions by checking the Summary of Product Characteristics for each drug. This information is publically available online (http://www.medicines.org.uk).**

1. Drugs affecting CYP3A4 or CYP2D6 metabolism that AstraZeneca strongly recommend are not combined with AZD4547

Table 1: Potent CYP3A4 or CYP2D6 inhibitors may increase exposure to AZD4547 more than 5-fold

Fluoxetine	Minimum of 35 days washout prior to AZD4547 administration and for 14 days following discontinuation of AZD4547
Ketoconazole<br>Ritonavir<br>Saquinavir<br>Indanavir<br>Nefazodone<br>Nelfinavir<br>Paroxetine<br>Quinidine	Minimum of 2 weeks washout prior to AZD4547 administration and for 2 weeks following discontinuation of AZD4547
Itraconazole<br>Clarithromycin (250mg or 500mg bd)	Minimum of 1 week washout prior to AZD4547 administration and for 2 weeks following discontinuation of AZD4547

Table 2: Potent Inducers of CYP3A4 may reduce exposure to AZD4547 by more than 3-fold

Barbiturates<br>Carbamazepine<br>Phenytoin<br>Rifampicin, Rifabutin<br>Troglitazone	Minimum of 2 weeks washout prior to AZD4547 administration and for 2 weeks following discontinuation of AZD4547
St John's Wort	Minimum of 3 weeks washout prior to AZD4547 administration and for 2 weeks following discontinuation of AZD4547

There are currently no data confirming that there is a pharmacokinetic (PK) interaction between these agents and AZD4547; a potential interaction is considered on the basis of preclinical data only. This list is not intended to be exhaustive, and a similar restriction will apply to other agents that are known to strongly modulate CYP3A4 or CYP2D6 activity. Appropriate medical judgment is required. Please contact the study coordinator at ICTU-Cancer with any queries you have on this issue.

2. Drugs affecting CYP3A4 metabolism that AstraZeneca considers may be allowed with caution

Table 3: Moderate Inhibitors of CYP3A4 or CYP2D6 may increase exposure to AZD4547

Warning of possible interaction	
Aprepitant<br>Diltiazem<br>Duloxetine<br>Erythromycin<br>Fluconazole<br>Sertraline<br>Terbinafine<br>Verapamil	Drugs are permitted but caution should be exercised and patients monitored closely for possible drug interactions. Please refer to full prescribing information for all drugs prior to co-administration with AZD4547.
Grapefruit juice<br>Seville oranges (and other products containing Seville oranges)	Patients should abstain from eating large amounts of grapefruit and Seville oranges (and other products containing these fruits e.g., grapefruit juice or marmalade) during the study (i.e. no more than a small glass of grapefruit juice (120 mL) or half a grapefruit or 1-2 teaspoons (15 g) of Seville orange marmalade daily).

3. Medicines that are significantly metabolised by CYP3A4 that AstraZeneca strongly recommend are not combined with AZD4547

Table 4: Exposure, pharmacological action and toxicity may be increased by inhibition of CYP3A4 by AZD4547

Alfentanil<br>Cyclosporin<br>Tacrolimus<br>Atorvastatin<br>Lovastatin<br>Simvastatin	Minimum of 1 week washout prior to AZD4547 administration and for 2 weeks following discontinuation of AZD4547
Carbamazepine	Minimum of 2 weeks washout prior to AZD4547 administration and for 2 weeks following discontinuation of AZD4547.

There are currently no data confirming that there is a pharmacokinetic (PK) interaction between these agents and AZD4547; a potential interaction is considered on the basis of preclinical data only. This list is not intended to be exhaustive, and a similar restriction will apply to other agents with narrow therapeutic windows that are known to depend on CYP3A4 for metabolism. Appropriate medical judgment is required. Please contact AstraZeneca with any queries you have on this issue.

4. MEDICINES THAT ARE SIGNIFICANTLY METABOLISED BY CYP3A4 THAT STRAZENECA CONSIDERS MAY BE ALLOWED WITH CAUTION

Table 5: Exposure, pharmacological action and toxicity may be increased by inhibition of CYP3A4 by AZD4547

Warning of possible interaction:	
Alprazolam<br>Erythromycin<br>Felodipine<br>Isradipine<br>Midazolam<br>Nifedipine<br>Tamoxifen<br>Trazodone<br>Triazolam<br>And possibly other calcium antagonists<br>Methylprednisolone<br>Pimozide<br>Quinidine	Drugs are permitted but caution should be exercised and patients monitored closely for possible drug interactions. Please refer to full prescribing information for all drugs prior to co-administration with AZD4547.

5. Drugs that may prolong QT interval

The drugs listed in this section are taken from information provided by The Arizona Center for Education and Research on Therapeutics and The Critical Path Institute, Tucson, Arizona and Rockville, Maryland.

Ref: http://www.arizonacert.org/medical-pros/drug-lists/drug-lists.htm

5.1 Drugs known to prolong QT interval

The following drugs are known to prolong QT interval or induce Torsades de Pointes and should not be combined with AZD4547. Recommended washout periods following cessation of treatment with these agents are provided in the following table.

**Contraindicated drug**	**Washout period prior to AZD4547 start**
Droperidol	2 days
Erythromycin	
Procainamide	
Cisapride	7days
Clarithromycin	
Disopyramide	
Dofetilide	
Domperidone*	

Contraindicated drug	Washout period prior to AZD4547 start
Ibutilide	
Quinidine	
Sotalol	
Sparfloxacin	
Thioridazine	
Bepridil	14 days
Chlorpromazine	
Halofantrine	
Haloperidol	
Mesoridazine	
Levomethadyl	4 weeks
Methadone	
Pimozide	
Arsenic trioxide	6 weeks*
Pentamidine	8 weeks
Amiodarone	1 year
Chloroquine	

* Estimated value as pharmacokinetics of arsenic trioxide has not been studied

5.2 Drugs that may possibly prolong QT interval

The use of the following drugs is permitted (notwithstanding other exclusions and restrictions) provided the patient has been stable on therapy for the periods indicated.

Warning of possible interaction	
**Drug**	**Minimum treatment period on medication prior to AZD4547 start**
Alfuzosin	2 days
Chloral hydrate	
Ciprofloxacin	
Dolasetron	
Foscarnet	
Galantamine	
Gemifloxacin	
Isradipine	
Ketoconazole	
Levofloxacin	
Mexiletine	
Nicardipine	

Warning of possible interaction	
**Drug**	**Minimum treatment period on medication prior to AZD4547 start**
Octreotide	
Ofloxacin	
Ondansetron	
Quetiapine	
Ranolazine	
Telithromycin	
Tizanidine	
Vardenafil	
Venlafaxine	
Ziprasidone	
Amantadine	7 days
Amitriptyline	
Amoxapine	
Clozapine	
Doxepin	
Felbamate	
Flecainide	
Fluconazole	
Fosphenytoin	
Gatifloxacin	
Granisetron	
Imipramine	
Indapamide	
Lithium	
Moexipril/HCTZ	
Moxifloxacin	
Risperidone	
Roxithromycin	
Sertraline	
Trimethoprim-Sulfa	
Trimipramine	
Voriconazole	
Azithromycin	14 days
Citalopram	
Clomipramine	

Warning of possible interaction	
**Drug**	**Minimum treatment period on medication prior to AZD4547 start**
Itraconazole	
Nortriptyline	
Paroxetine	
Solifenacin	
Tacrolimus	
Fluoxetine	5 weeks
Protriptyline	6 weeks
Tamoxifen	8 weeks
