# Supplementary material for: Results of the phase IIa RADICAL trial of the FGFR inhibitor AZD4547 in endocrine resistant breast cancer
Source: Nat Commun. 2022 Jun 10;13:3246. doi: 10.1038/s41467-022-30666-0 (PMC9187670; doi:10.1038/s41467-022-30666-0)
Supplement: Supplementary file 3 — Reporting Summary [file 41467_2022_30666_MOESM3_ESM.pdf]

nature portfolio

Corresponding author(s): RC Coombes, PD Badman, JP Lozano-Kuehne, X Liu, IR Macpherson, I Zubairi, RD Baird, N Rosenfeld, J Garcia-Corbacho, N Cresti, ER Plummer, A Armstrong, R Allerton, D Landers, H Nicholas, L McLellan, A Lim, F Mouliere, OE Pardo, MJ Seckl

Last updated by author(s): 2022/04/12

Reporting Summary

Nature Portfolio wishes to improve the reproducibility of the work that we publish. This form provides structure for consistency and transparency in reporting. For further information on Nature Portfolio policies, see our Editorial Policies and the Editorial Policy Checklist.

Statistics

For all statistical analyses, confirm that the following items are present in the figure legend, table legend, main text, or Methods section.

n/a	Confirmed	
☐	☒	The exact sample size (n) for each experimental group/condition, given as a discrete number and unit of measurement
☐	☒	A statement on whether measurements were taken from distinct samples or whether the same sample was measured repeatedly
☐	☒	The statistical test(s) used AND whether they are one- or two-sided<br>*Only common tests should be described solely by name; describe more complex techniques in the Methods section.*
☐	☒	A description of all covariates tested
☐	☒	A description of any assumptions or corrections, such as tests of normality and adjustment for multiple comparisons
☐	☒	A full description of the statistical parameters including central tendency (e.g. means) or other basic estimates (e.g. regression coefficient) AND variation (e.g. standard deviation) or associated estimates of uncertainty (e.g. confidence intervals)
☐	☒	For null hypothesis testing, the test statistic (e.g. F, t, r) with confidence intervals, effect sizes, degrees of freedom and P value noted<br>*Give P values as exact values whenever suitable.*
☒	☐	For Bayesian analysis, information on the choice of priors and Markov chain Monte Carlo settings
☒	☐	For hierarchical and complex designs, identification of the appropriate level for tests and full reporting of outcomes
☐	☒	Estimates of effect sizes (e.g. Cohen's d, Pearson's r), indicating how they were calculated

Our web collection on statistics for biologists contains articles on many of the points above.

Software and code

Policy information about availability of computer code

Data collection	Data were recorded using the InForm (version 4.6) electronic data capture (EDC) and management system.
Data analysis	Data analysis were performed using Stata 16.1 (StataCorp. 2019. Stata Statistical Software: Release 16. College Station, TX: StataCorp LLC) and packages in R 4.0.0 (R Core Team (2020). R: A language and environment for statistical computing. R Foundation for Statistical Computing, Vienna, Austria. URL https://www.R-project.org/)

For manuscripts utilizing custom algorithms or software that are central to the research but not yet described in published literature, software must be made available to editors and reviewers. We strongly encourage code deposition in a community repository (e.g. GitHub). See the Nature Portfolio guidelines for submitting code & software for further information.

Data

Policy information about availability of data

All manuscripts must include a data availability statement. This statement should provide the following information, where applicable:

- Accession codes, unique identifiers, or web links for publicly available datasets
- A description of any restrictions on data availability
- For clinical datasets or third party data, please ensure that the statement adheres to our policy

The full study protocol is available as Supplementary Note 2 in the Supplementary Information file. The gene expression data generated in this study have been deposited in the Gene Expression Omnibus (GEO) database under accession code GSE198650. The link to access the data is https://www.ncbi.nlm.nih.gov/geo/query/acc.cgi?acc=GSE198650. The raw and processed data will be freely and publicly available from 18.03.2022 and can be used for further analysis by other

investigators.
Source data for graphs are provided in the Supplementary Information/Source Data file. The datasets generated during and/or analysed in the study which are not made publicly available due to data privacy laws can be made available upon reasonable request. Any requests for additional clinical data will be reviewed by the Imperial Clinical Trials Unit (Study Chief Investigator, Study Operations Manager, Head of Statistics, Q.A. Manager and Director of Operations). Any data to be shared will need a Data Sharing Agreement in place. All data shared will be de-identified. Any requests for clinical data should be addressed to Raoul Charles Coombes (c.coombes@imperial.ac.uk) and Michael Seckl (m.seckl@imperial.ac.uk)

Field-specific reporting

Please select the one below that is the best fit for your research. If you are not sure, read the appropriate sections before making your selection.

- [x] Life sciences
- [ ] Behavioural & social sciences
- [ ] Ecological, evolutionary & environmental sciences

For a reference copy of the document with all sections, see nature.com/documents/nr-reporting-summary-flat.pdf

Life sciences study design

All studies must disclose on these points even when the disclosure is negative.

Sample size
Justification of sample size: With a power of 85% and type I error of one-sided 0.05, 4 or more patients with clinical benefit out of 20 patients was the calculated criteria for the study to safely continue. The required total sample size for phase IIa assuming a standard deviation of 0.30 for the change in tumour size was 50 patients. The 95% confidence interval for the observed geometric mean will extend 1.09 in either direction with the total sample size.

Data exclusions
No data were excluded from all patients enrolled in the study.

Replication
A central review of subject's CT scans by RECIST was undertaken by an independent radiologist: Dr Adrian Lim, Imperial College Healthcare NHS Trust. Patient response to treatment was assessed at participating sites and by the Central Independent Reviewer using RECIST v1.1 criteria. The Guidelines for Evaluation of Objective Tumour Response using RECIST 1.1 (Response Evaluation Criteria in Solid Tumours) were included as an Appendix in the study protocol.
There were no significant discrepancies and any minor discrepancies were resolved on consensus re-review of the scans.

Randomization
The study is a single-arm study.

Blinding
Blinding is not relevant to the study. The study is a one arm study. All participants received the treatment.

Reporting for specific materials, systems and methods

We require information from authors about some types of materials, experimental systems and methods used in many studies. Here, indicate whether each material, system or method listed is relevant to your study. If you are not sure if a list item applies to your research, read the appropriate section before selecting a response.

Materials & experimental systems

n/a	Involved in the study	
x		Antibodies
x		Eukaryotic cell lines
x		Palaeontology and archaeology
x		Animals and other organisms
	x	Human research participants
	x	Clinical data
x		Dual use research of concern

Methods

n/a	Involved in the study	
x		ChIP-seq
x		Flow cytometry
x		MRI-based neuroimaging

Human research participants

Policy information about studies involving human research participants

Population characteristics	Eligible patients were those with metastatic breast cancer whose cancers had progressed on treatment with anastrozole or letrozole, either in the adjuvant or first line metastatic (safety run-in only) or any setting (phase IIa only). The NSAI did not have to be the most recent line of treatment. Inclusion criteria included: written informed consent, ECOG performance status 0-1; ≥ 25 years of age, post-menopausal with histologically-confirmed ER+ breast cancer (primary or metastatic tumour tissue) with at least 1 lesion that could be accurately assessed by CT/MRI/x-ray at baseline.
Recruitment	Patients with metastatic breast cancer who had progressed on treatment with anastrozole or letrozole, who fitted the protocol eligibility criteria were approached and consented at the clinics in our 7 recruiting sites. Any patients who fitted the eligibility criteria in the study protocol were approached by their Healthcare team who discussed the study with them. Interested patients were then given the PIS to read and were given the chance to discuss and ask any questions with their Healthcare team. Interested patients were then consented (informed written consent) to the study (treatment and samples).<br><br>There were no self-selection bias or other biases identified in this patient group.
Ethics oversight	The study was approved by NRES Committee East Midlands - Derby 1 (Ref: 11/EM/0393) on 23.12.2011. The favorable opinion applied to all NHS sites participating in the study, subject to permission being obtained from each NHS site R&D office prior to them starting in the study. The other condition of the Ethics approval was that a Clinical Trial Authorisation must be obtained from the MHRA before the study could begin. A favorable approval from the MHRA was given on 17/01/12.<br><br>The Research and Development (R&D) Department at each participating institution are as follows:<br>Charing Cross Hospital - Imperial College Healthcare NHS Trust Joint Research Compliance Office<br>Beatson West of Scotland Cancer Centre - NHS Greater Glasgow and Clyde R&D Central Office<br>Cancer Research UK Cambridge Centre, Addenbrooke's Hospital – Cambridge University Hospitals NHS Foundation Trust R&D Department<br>Northern Centre for Cancer Care, Freeman Hospital – Newcastle upon Tyne Hospitals NHS Foundation Trust Joint Research Office<br>Christie Hospital, Manchester – The Christie NHS Foundation Trust, The R&D Division<br>Russells Hall Hospital – The Dudley Group NHS Foundation Trust R&D Directorate<br>Queen's Hospital Burton – Burton Hospitals NHS Foundation Trust R&D Department

Note that full information on the approval of the study protocol must also be provided in the manuscript.

Clinical data

Policy information about clinical studies

All manuscripts should comply with the ICMJEguidelines for publication of clinical research and a completedCONSORT checklist must be included with all submissions.

Clinical trial registration	The study was registered with The European Union Clinical Trials Register on 5/1/2012 (2011-000454-32) and on the ISRCTN Register (80307982)on 27/04/2012. The study was also registered on ClinicalTrials.gov (NCT01791985)
Study protocol	A copy of the protocol has been sent as part of this application.<br>The protocol is also available at http://www.imperialclinicaltrialsunit.org/trials/
Data collection	The data was collected from 7sites: Imperial College London NHS Trust; Beatson West of Scotland Cancer Centre, Glasgow; Addenbrookes Hospital, Cambridge; Northern Centre for Cancer Care, Freeman Hospital, Newcastle; Christie Hospital, Manchester; Russells Hall Hospital; Queen's Hospital, Burton. Recruitment for the Safety Run-In occurred from 23/10/12 to 07/08/13 and recruitment for the PIIa part of the study occurred between 24/04/14 and 11/12/15. Data was collected from October 2012 to August 2018.
Outcomes	Primary Outcomes:<br>Safety run-in:<br>* Safety and tolerability and the dose of AZD4547 to be used in combination with a standard dose of anastrozole/letrozole for the phase IIa part of the study<br>Phase IIa:<br>* Efficacy of AZD4547 based on the change in tumour size at 12 weeks (or progression if prior to week 12), when used in combination with either anastrozole or letrozole in ER positive breast cancer patients who have progressed on treatment with either anastrozole or letrozole in any setting<br><br>Secondary Outcomes:<br>Safety run-in<br>* Pharmacokinetics (PK) of anastrozole or letrozole when given alone compared to in combination with AZD4547<br>Phase IIa<br>* Efficacy of AZD4547 in combination with anastrozole or letrozole as measured by:<br>- change in tumour size at 6 weeks, 20 weeks, then every 8 weeks, as per study plan

- tumour response (RECIST criteria) at 6 weeks, 12 weeks, then every 8 weeks, as per study plan
- objective response rate (ORR) at 6 weeks, 12 weeks, then every 8 weeks, as per study plan
- progression-free survival (PFS)

* Safety and tolerability of AZD4547 in combination with anastrozole or letrozole
